# Longitudinal study of a rodent invasion reveals shifts in host pathogen interactions

**DOI:** 10.1371/journal.ppat.1014349

**Published:** 2026-07-23

**Authors:** Andrew McManus, Maxime Galan, Nathalie Charbonnel, Celia Holland, Vincent Sluydts, Annetta Zintl, Peter Stuart

**Affiliations:** 1 Department of Biological and Pharmaceutical Sciences, Munster Technological University, Tralee, Ireland; 2 CBGP, INRAE, CIRAD, Institut Agro, IRD, University of Montpellier, Montpellier, France; 3 Department of Zoology, Trinity College Dublin, the University of Dublin, Dublin, Ireland; 4 Department of Biology, Evolutionary Ecology Group, University of Antwerp, Wilrijk, Belgium; 5 University College Dublin, School of Veterinary Medicine, Veterinary Science Centre, Belfield, Ireland; New York Medical College, UNITED STATES OF AMERICA

## Abstract

Invasive species can disrupt native epidemiological processes, potentially leading to the emergence of zoonotic pathogens. The relatively recent appearance and spread of the bank vole (*Clethrionomys glareolus*) in Ireland provides a unique model system to study these phenomena. The depauperate small mammal community in Ireland, combined with baseline data, allows us to identify the invasion gradient of *C. glareolus* and its effects on rodent-borne pathogens over time. A total of 498 *C. glareolus* and 584 *Apodemus sylvaticus* were sampled, via removal trapping, across nine sites in Ireland in 2016 and 2017, with six sites revisited in 2021 and 2022. 16S rRNA metabarcoding identified 10 putative pathogenic Operational Taxonomic Units (OTUs) present in these rodents, relating to four bacterial taxa, *Bartonella spp., Mycoplasma coccoides, Mycoplasma haemomuris and Mycoplasma penetrans,* and one family of protozoans, Sarcocystidae. Several epidemiological processes were found to be correlated with the *C. glareolus* invasion; firstly, *C. glareolus* in Ireland exhibited patterns consistent with the enemy release hypothesis, compared to native populations in France, and the native *A. sylvaticus* in Ireland. Secondly, a potential dilution effect, with reduced prevalence of *M. haemomuris* in *A. sylvaticus* at the *C.* *glareolus* invasion core compared to the invasion front, was observed*.* Finally, *C. glareolus* had increased prevalence of Sarcocystidae at the invasion front when compared to the invasion core, depending on the time and stage of invasion. These findings further our understanding of pathogen dynamics during biological invasions, demonstrating that invaders affect native host-pathogen communities differently as they advance through various stages of establishment.

## Author summary

When animals are introduced to new environments, they can disrupt local epidemiological patterns by changing how pathogens spread among native wildlife. The spread of the bank vole, a non-native rodent in Ireland, offers a valuable model system for studying how such invasions affect disease dynamics over time. In this study, we examined how the arrival of the bank vole is influencing the health of the native wood mouse. We found that in areas where bank voles have been established longer, wood mice carry fewer infections from a common blood parasite. At the same time, bank voles themselves carried fewer types of pathogens overall than wood mice, possibly giving them an advantage in terms of survival and reproduction. Interestingly, we also observed that bank voles were more likely to carry a specific group of parasites, called Sarcocystidae, in areas they had only recently invaded. These patterns suggest that invading species can reshape disease dynamics in complex and evolving ways. Understanding how these changes unfold over time is important for predicting future disease risks to wildlife and potentially to humans.

## Introduction

The emergence of zoonotic pathogens has seen a sharp increase since the 1940’s, with 60% of emerging infectious diseases originating from animals, including the recent outbreaks of SARS-CoV-2, Lassa, Ebola and Mpox viruses [1–7]. This increase in the frequency of emerging infectious diseases, which poses a risk to humans, livestock and indigenous wildlife populations, has been primarily attributed to environmental change, caused by landscape change, a reduction of habitat diversity and the introduction of invasive species [8–16].

A recent review by Bezerra-Santos *et al.* [15], highlights that invasive mammal species pose increased risks for public health by introducing zoonotic pathogens [17,18]. However, non-native species can also benefit from a reduction in natural enemies, such as predators or pathogens, compared to conspecifics in their native range (and often compared to the native species), through a process known as “enemy release” [19,20]. In relation to pathogens, this can happen because the pathogen, or the vector responsible for its transmission, is absent from the founder population, or because the environment in the new range is unsuitable to sustain the pathogen or vector [21–25]. This, in turn, may give invaders a competitive advantage over native species. Additionally, invasive species can influence host-pathogen ecological interactions in native hosts in several other ways. Firstly, invasive species can co-invade with new pathogens, which may infect native hosts, leading to a process known as pathogen spillover [26]. The establishment of invasive species may also lead to a dilution of native pathogens, where the presence of a host with a lower competence for a specific pathogen can reduce the circulation of that pathogen in competent hosts [27–29]. On the other hand, invasive host species can also have an increased competency for native pathogens, causing a spillback of native pathogens that amplifies infection in native hosts [30,31].

Rodents, the largest order of mammals, make up one of the most common group of invasive species worldwide, affecting native ecosystems, food production and human health [32]. Additionally, a recent review by Dharmarajan *et al.* [6], highlighted that rodents and bats harbour the highest proportion of zoonotic agents, identifying these taxa as species of interest for studying zoonoses. Therefore, rodents make excellent models for studying the eco-epidemiology of pathogens due to their presence in large numbers and broad distributions, playing an important role as reservoirs for pathogens causing zoonotic disease; such as, orthohantaviruses, *Leptospira* and *Bartonella* species, or *Toxoplasma gondii* [6,33–37]. Finally, studies on rodent invasions have demonstrated the interplay between rodent pathogen interactions and biological invasions, for example, helminth studies in both Ireland and Senegal have observed patterns of enemy release in invasive rodents when comparing sites of long established populations to more recently invaded sites [24,31,38,39].

The case of the bank vole, *Clethrionomys glareolus* invasion in Ireland presents a unique opportunity to explore changes in epidemiological dynamics during a biological invasion [37], particularly due to the depauperate woodland rodent community in Ireland, that includes only the invasive *C. glareolus* and the native woodmouse, *Apodemus sylvaticus* [40]. Species depauperate systems provide a valuable scenario to tease out the effects of invasive species on native pathogen communities. For example, in this instance the depauperate rodent community in Ireland has allowed for easier identification of the effects the *C. glareolus* invasion has on the native *A. sylvaticus* population in previous studies [31,39]. Furthermore, *C. glareolus* in Ireland has a single identifiable introduction point in the south west, near Foynes, Co. Limerick, and the likely source population from Germany, which was identified through mitochondrial DNA [41]. In addition, studies have shown *C. glareolus* in Ireland to have an expansion rate of 2.23 – 2.63 km per year in unconstrained areas [42]. Furthermore, previous studies have shown a reduced relative abundances of *A. sylvaticus* at sites invaded by *C. glareolus* [39,43]. This has allowed for the establishment of cross-sectional studies with an invasion gradient, containing sites from the invasion core (established from the introduction point, where *C. glareolus* exhibits it’s highest relative abundance), invasion front (sites where *C. glareolus* had most recently invaded), and uninvaded sites (*A. sylvaticus* only) [39]. These factors combined, provide an excellent case study to identify the epidemiological effects that the *C. glareolus* invasion has on native pathogen communities in *A. sylvaticus*, such as spillover, dilution, or spillback.

The development of high-throughput sequencing, and in particular metabarcoding techniques, facilitates a more holistic approach to studying the rodent pathobiome [44]. These methodologies provide a global picture of pathogen diversity within host species, improving our understanding of the relationship between host-parasite interactions and biological invasions. In the Irish context, rodent helminth parasite communities have been explored previously by Loxton *et al.* [23,31] and Stuart *et al.* [39]. However, no such data exists on pathogenic microparasites in this model system. Furthermore, there remains a knowledge gap concerning longitudinal studies focusing on changes in pathogen communities during biological invasions. Such studies are essential for fully exploring changes in host-pathogen interactions, such as pathogen loss in invasive species, as cross-sectional studies only capture a single timepoint of this dynamic process [37,39,45–47].

In this study, we aim to use 16S rRNA amplicon sequencing to characterise the bacterial community with zoonotic potential or pathogenic potential to rodents associated with *C. glareolus* and *A. sylvaticus* in Ireland within a longitudinal study. We performed a spatiotemporal analysis of this community along the invasion gradient described above, using rodents removed in 2016 from a previous study by Stuart *et al.* [39], with subsequent removal sampling at these sites in 2017, 2021 and 2022. In this study, we use the 16s rRNA amplicon metabarcoding approach developed by Galan *et al*. [48], and recently used by Bouilloud *et al.* [49]. Focusing on putative pathogenic taxa recorded in samples collected between 2016 and 2022, this present study addresses three main hypotheses: (i) whether *C. glareolus* exhibits patterns consistent with the enemy release hypothesis, including a lower pathogen species richness of potentially pathogenic taxa when compared to its native range and the native *A. sylvaticus;* (ii) whether the presence of *C. glareolus* alters the pathogen communities present in the native *A. sylvaticus* as expected under the dilution or spillback hypotheses; finally (iii) whether pathogen prevalence changes over time in both rodent species, as the invader becomes established.

## Materials & methods

### Ethics statement

This study was carried out under ethical approval from the universities Trinity College Dublin animal research ethics committee and Munster Technological University ethics board, in compliance with the Health Products Regulatory Association (HPRA) of Ireland for euthanasia of the rodents sampled (Authorisation Number: AE22171/I004).

### Field work & sample collection

**Fig 1:**
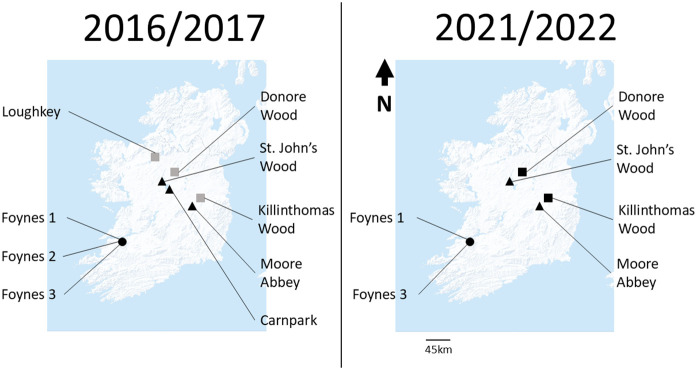
Map of Ireland, showing sampling sites visited in 2016, 2017, 2021 and 2022. Circles indicate the invasion core; triangles indicate the invasion front in 2016 (‘front 2016’) and squares indicate the invasion front in 2021 (‘front 2021’). The grey colour shows sites that were uninvaded by *Clethrionomys glareolus* on a given date, while the black colour shows sites that were invaded. “Base map layer from https://mapswire.com/maps/ireland/, provided under a creative commons licence (CC-BY 4.0).”.

Nine sites were selected for field sampling in 2016, as described in Stuart *et al.* [39]. These were divied into three regions based on their invasion status; including three invasion ‘core’ sites in Foynes (Foynes 1, 2 & 3), three 2016 invasion front sites (named ‘front 2016’ herein, i.e., Moore Abbey, Carnpark, and St. John’s), and three 2021 invasion front sites (named ‘front 2021’ herein, i.e., Killinthomas, Loughkey and Donore, uninvaded in 2016) (Fig 1). In 2016, rodents were sampled at all of these nine sites in spring and autumn trapping sessions and revisited in autumn 2017. For the longitudinal aspect of this study, only six of these sites were revisited in spring and autumn 2021, and spring 2022 (S1 Table). These sites included two of the ‘core’ sites in Foynes (Foynes 1 and 3, not Foynes 2), two invasion ‘front 2016’ sites (Moore Abbey and St. John’s wood, not Carnpark) and two invasion “front 2021” sites (Killinthomas and Donore, not Loughkey) (Fig 1).

Longworth traps, baited using peanuts, bird seed mix and a piece of carrot, were placed 5-10m apart in transects of a maximum of 20 traps. A maximum of 24 animals from each species were removed from each site per season and year of sampling. The maximum sample size was calculated based on the estimated sample required to observe at least one infected individual based on a prevalence of 12%, and at least 1 success, with 95% confidence intervals (*i.e.,* a sample size of 24 should allow detection of all pathogens with a prevalence of 12% and above, allowing detection of rare pathogens, calculated using Sampsize tool available at: https://sampsize.sourceforge.net/iface/index.html#prev). Traps were placed in the field overnight and checked early the next morning. Rodents were inspected in the field and transported back to the laboratory. If insufficient numbers were trapped, the site was revisited for an additional night within the same season.

### Sample collection and host characterisation

Rodents were euthanised using Isoflurane (1000mg/g, under license from the Health Products Regulatory Authority). The spleens were removed immediately and stored in RNAlater at 4°C for 24 hours, then placed into the freezer at -20°C for further metabarcoding analysis. Rodents were classified into three age categories, juvenile, young adult and mature adult, as described in Behnke *et al.* [50]. These were based on the criteria of rodent length (nose to anus; in millimetres) and weight (grams) as described by Loxton *et al.* [23,31] and used by Stuart *et al.* [39]. For animals found to be at the limit of each classification, visual assessments of maturity were used for final determination, as described by Stuart *et al.* [39].

### Pathogen detection in Irish rodent spleen samples

The 16S metabarcoding analysis was conducted as described by Galan *et al.* [48]. DNA was extracted from the rodent spleens using DNeasy 96 Tissue Kit (Qiagen, Germany). This organ was selected by Galan *et al.* [48], as the spleen is responsible for detecting and removing blood-borne microrganisms. Extracted DNA was screened for putative pathogenic Operational Taxonomic Units (OTUs) using the v4 hyper variable region of the 16S rRNA gene with universal primers (16S-V4F 5’-GTGCCAGCMGCCGCGGTAA-3’ and 16S-V4R 5’-GGACTACHVGGGTWTCTAATCC-3’), via Illumina MiSeq sequencing. Finally, PCR amplification, sequencing and taxonomic identification were carried out as described by Galan *et al.* [48]. From the clusters of microorganism DNA detected, putative pathogenic OTUs were filtered and selected [51]. First, we discarded putative false positive results associated with sequence counts below two OTU-specific thresholds, which checked respectively for putative cross-contamination between samples during DNA extraction and PCR steps on the one hand, and incorrect assignment due to the generation of mixed clusters on the flowcell during Illumina sequencing on the other hand [52]. Then, for each sample, and each OTU, we kept only the occurrences confirmed by the two PCR replicates of the same sample. Finally, the putative pathogenic OTUs were selected using their taxonomic affiliations and based on their potential zoonotic risk and pathogen impact on rodents, to separate them from commensal species [51]. It should be noted that 16S rRNA amplicon sequencing primarily only allows detection at the genus level for some bacterial taxa and may not reliably resolve species or strain level diversity without targeted, genus‑specific genetic markers.

### Statistical analyses

#### Calculation of prevalence and species richness.

Data were analysed using the R version 4.3.1 working under the RStudio software hoods (2023.06.1 Build 524). The packages Plyr [53] and ggplot2 [54] were used to create tables and figures. Separate putative pathogenic OTUs of the same pathogen species were combined into potentially pathogenic taxa for analysis. For each putative pathogenic taxa, prevalence was calculated by the total number of infected individuals divided by the total population sampled. Confidence intervals (maximum and minimum) were calculated to 95% using the R package PropCIs [55]. Pathogen species richness was calculated by counting the total number of putative pathogenic taxa recorded in an individual.

#### Examining patterns of infection relating to the enemy release hypothesis.

Firstly, bacterial data collected from *C. glareolus* and *A. sylvaticus* in Ireland from 2021 was compared to data collected from rodents trapped in forest sites in France, sampled in 2021 by Pradel *et al.* [56], using the same 16S metabarcoding approach [48]. These data were examined by comparing prevalences of potentially pathogenic bacterial taxa in rodents from Ireland and France, to examine if less species were detected and at a lower prevalence in Ireland, related to the enemy release hypothesis. However, due to differences in assigning age and maturity to animals in each country, statistical analysis could not be conducted. Likewise, while the bacterial pathogens were filtered using the same methodology, two extra species of potentially pathogenic Sarcocystidae were included in the Irish data, and consequently meant that these pathogen species could not be included in the comparison. Differences between pathogen communities in hosts from France and Ireland were examined using Bray–Curtis dissimilarities calculated in R using the package vegan. Where community level analysis was not appropriate due to low numbers of infected hosts, differences in pathogen prevalence between countries were assessed using direct comparisons of infection frequencies. Fisher’s exact tests were used when expected cell counts were low, and Pearson’s chi-square tests (without continuity correction) were used when expected counts were sufficient.

#### Examining patterns of infection in Irish rodents.

To investigate patterns of infection in each host species in Ireland, pathogens with prevalence above 5% in the overall dataset (all sampling dates and sites) were selected for statistical analysis using generalised linear mixed models (GLMM’s) and a binomial distribution [24]. The threshold of 5% prevalence was selected as variations below this threshold would be too low for appropriate statistical testing. GLMM’s from the package glmmTMB were used [57]. GLMMs were selected for these analyses to allow the inclusion of both fixed and random effects in the models, and the appropriate distribution for each type of data being analysed. Due to the depauperate pathogen communities in Ireland, zero-inflated negative binomial distributions were used to investigate patterns in pathogen species richness instead of Poisson distributions used by Diagne *et al.* [24].

Two strategies were used to analyse these data;

1. **The invasion gradient analysis**: only prevalence data from 2016 (and all nine sites) were included to test the influence of the invasion gradient on infection patterns in both *A. sylvaticus* and *C. glareolus,* as this year had a very clear “core”, “front” (front 2016) and “uninvaded” (front 2021) sites as per the study by Stuart *et al.* [39] (see Fig 1). An additional model examining the difference in pathogen species richness between both rodent species in Ireland, and a separate model focusing on *A. sylvaticus* only, was conducted using these data from 2016.
2. **The longitudinal analysis:** prevalence data from all four years were used, but only the six sites that were sampled each year were selected, excluding Foynes 2, Carnpark and Loughkey, to keep the data comparable, as these sites were not revisited in 2021 and (Table 1). In this approach, time was included in the models as a numerical variable counting years from baseline time, i.e., 2016 = 1.

**Table 1:** Models and data used for the two approaches of this study, the invasion gradient analysis and the longitudinal analysis. Time:Season indicates the interaction that was used between time and season. Time is defined as years since 2016 baseline, with 2016 = 1.

	Invasion gradient analysis	Longitudinal analysis
**Fixed variables**	Season, Region, Age Class, Sex Rodent Species*	Time, Season, Region, Age Class, Sex, Time:Season
**Random variables**	Site	Site
**Years**	2016 only	All (2016, 2017, 2021, 2022)
**Sites**	All (Fig 1.)	6 repeated sites; Foynes 1 & 3, Killinthomas, Donore, Moore Abbey and St. John’s.

*The fixed variable Rodent species was only used in the model investigating differences in pathogen species richness between *Clethrionomys glareolus* and *Apodemus sylvaticus* in Ireland.

Factors used in each model included season, region (invasion region; core, front 2016, front 2021), sex, age class (juvenile, young adult and mature) as fixed effects, with site included as a random effect variable (Table 1). The longitudinal analysis included the additional fixed effects of time, as years since baseline (2016), and an interaction between season and time. An additional fixed factor, rodent species, was used for the model investigating the variance in pathogen species richness between Irish populations of *C. glareolus* and *A. sylvaticus* in 2016. The MuMIn package was used to refine and find the best model based on lowest Akaike information criterion corrected for small sample size (AICc) value [58]. The DHARMa package was then used to check model fit and the performance package to test for multicollinearity and overdispersion [59,60]. The optimisation function, Optim, using the Broyden, Fletcher, Goldfarb and Shanno (BFGS) method was used in cases where model optimisation was needed. Post-hoc tests using Tukey Kramer analysis were used for factors with three or more modalities, while post-hoc Pearson’s correlation tests were used to analyse changes in prevalence with time. In cases where no GLMM could be fitted, non-parametric tests were used, such as a Wilcoxon Rank Sum test.

These analyses used the categorical variable of region (invasion region; core, front 2016, front 2021) as a proxy for invasion levels of *C. glareolus* relative to *A. sylvaticus* to aid in the interpretation of the results and increase statistical power through increased number of samples per variable. In order to justify our classification of invasion region in the analysis, the percentage of *A. sylvaticus* relative to *C. glareolus* was tested against invasion region. For these analyses, the total number *A. sylvaticus* relative to *C. glareolus* trapped in each site in each season within a given year was calculated, generating a percentage of *A. sylvaticus* relative to *C. glareolus.* These numbers were then assigned to each sample corresponding to their trapping site, season and year*.* Finally, to justify the classification of regions, this assigned percentage of *A. sylvaticus* relative to *C. glareolus* in the dataset was tested against region using a Kruskal-Wallis test, and a post-hoc Dunn’s test, through the dunn.test R package [61], to check differences between regions in both 2016 only, and the longitudinal dataset, as the data followed a non-normal distribution.

## Results

### Pathogens detected in Irish rodent spleen samples

Results are presented following the analytical structure described in the Materials and Methods: (i) pathogen detection and prevalence, (ii) comparison with native‑range populations to examine enemy release, (iii) invasion‑gradient analyses (2016), and (iv) longitudinal analyses (2016–2022).The 16S analyses of 584 *A. sylvaticus* and 498 *C. glareolus* trapped over the four years of sampling in Ireland revealed 10 putative pathogenic OTUs corresponding to five potentially pathogenic taxa (Table 2; Sequencing information, raw sequence reads and prevalence tables are available to the following link: https://zenodo.org/records/14883529). These included one OTU of the genus *Bartonella*, a bacterial genus potentially including zoonotic species (e.g., *B. henselae*) [62], three OTUs of *Mycoplasma haemomuris* (combined for analysis), one OTU of *Mycoplasma coccoides* and one OTU of *Mycoplasma penetrans* (known to be zoonotic). These three *Mycoplasma* species have previously been associated with immunological and pathological effects in rodents, particularly through modulation of host immune responses to co-infecting pathogens [63–65]. While more recent studies suggest that Haemoplasmas (Hemotropic mycoplasmas that include the species *M. coccoides* and *M. haemomuris*) may be largely subclinical in wildlife they have also highlighted the need for further studies increasing their geographic range and elucidating transmission [66]. Finally, we detected four OTUs of Sarcocystidae (combined for analysis, a family of protozoans, with some species that are zoonotic, and others that are pathogenic for rodents) [67,68]. All five taxa were detected in *C. glareolus*, all of which had a 1% prevalence or below, except for Sarcocystidae*,* which had a prevalence of 37.15%*.* All potentially pathogenic taxa were also found in *A. sylvaticus* (Table 2; the prevalence for each region per year available in S2 Table), except *M. penetrans*. However, it should be noted that only one individual *C. glareolus* was found to be positive for this pathogen out of 498 tested.

**Table 2:** Prevalence (%) of potentially pathogenic taxa in all *Apodemus sylvaticus* and *Clethrionomys glareolus* sampled in Ireland from 2016 - 2022. The 95% confidence interval (maximum and minimum value) is detailed in brackets.

	Transmission	*Apodemus sylvaticus*	*Clethrionomys* *glareolus*
**Pathogen**		***n = 584***	***n = 498***
*Bartonella*	Vector (flea)	51.54 (47.40 - 55.66)	0.2 (0.01 - 1.11)
*Mycoplasma haemomuris*	Vector (lice)*	44.01 (39.93 - 48.14)	1 (0.33 - 2.33)
*M. coccoides*	Vector (lice)*	44.18 (40.10 - 48.31)	0.2 (0.01 - 1.11)
*M. penetrans*	Sexual	0.00	0.2 (0.01 - 1.11)
Sarcocystidae	Dependent on species, *i.e.,* Environmental (Direct life cycle), or intermediate host (indirect life cycle) in some cases.	1.71 (0.82 - 3.13)	37.15 (32.89 - 41.56)

*Mycoplasma transmission has been linked to ectoparasites such as lice, based on early observations and PCR detection in arthropod vectors, although more experimental evidence for biological transmission is required [69].

### Patterns of infection relating to the enemy release hypothesis

**Fig 2:**
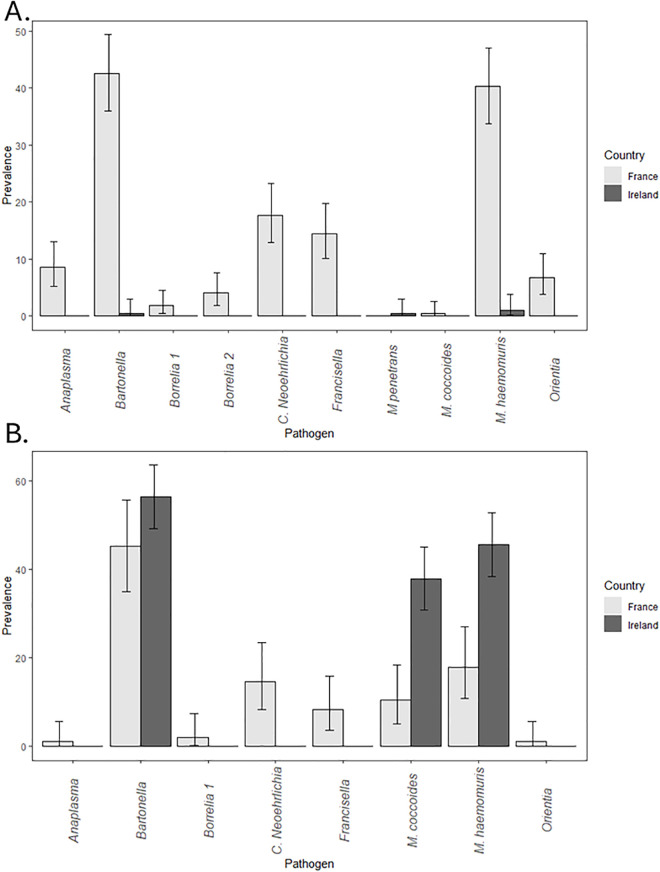
Prevalence of potentially pathogenic bacterial taxa observed in 2021 in both France (*Clethrionomys glareolus* native range, light grey) and Ireland (*C. glareolus* invasive range, dark grey) in A. *C. glareolus* and B. *Apodemus sylvaticus.* Error bars represent 95% confidence intervals. ***C.*** *Neoehrlichia* represents *Candidatus Neoehrlichia.*

Based on data from 2021, *C. glareolus* in Ireland (n = 189) exhibited fewer potentially pathogenic bacterial taxa than *C. glareolus* sampled in forest sites in France (n = 221). Most notably, *Anaplasma, Borrelia, Candidatus Neoehrlichia, Francisella and Orientia* were absent from the Irish samples. In addition, lower prevalences of *Bartonella* and *M. haemomuris* were also observed in the Irish *C. glareolus* specimens (Fig 2A). In contrast, *A. sylvaticus* individuals trapped in Ireland (*n =* 193) had a similar prevalence of *Bartonella* compared to forest site populations in France in 2021 (*n* = 95), but higher prevalences of *M. haemomuris and M. coccoides*. As above, *Anaplasma, Borrelia, Candidatus Neoehrlichia, Francisella and Orientia* were not detected in the Irish *A. sylvaticus* samples (Fig 2B).

Due to the high proportion of *C. glareolus* trapped in Ireland in which no pathogens were detected, it was not possible to conduct community level similarity analyses for this species. Removal of pathogen negative individuals resulted in only five Irish *C. glareolus* remaining (one positive for *Bartonella*, two for *M. haemomuris*, and one for *M. penetrans*), which was insufficient for appropriate multivariate community analysis. Therefore, pathogen-specific prevalence comparisons were conducted for the three bacterial taxa detected in both France and Ireland: *Bartonella*, *M. haemomuris*, and *M. coccoides*.

*C. glareolus* from Ireland were found to have significantly lower prevalences of *Bartonella* (Ireland: 0.53%; France: 42.53%; Fisher’s exact test, *p* < 0.01) and *M. haemomuris* (Ireland: 1.06%; France: 40.27%; Fisher’s exact test, *p* < 0.01) than those from France. No significant association was detected between country and prevalence of *M. coccoides* in *C. glareolus* (Ireland: 0%; France: 0.45%; Fisher’s exact test, *p* > 0.05).

In contrast, *A. sylvaticus* from Ireland showed significantly higher prevalences of *M. coccoides* (Ireland: 37.82%; France: 10.52%; χ² = 23.13, *p* < 0.01) and *M. haemomuris* (Ireland: 45.60%; France: 17.89%; χ² = 21.08, *p* < 0.01) than those from France. No significant association was detected between country and prevalence of *Bartonella* in *A. sylvaticus* (Ireland: 56.48%; France: 45.26%; χ² = 3.21, *p* > 0.05).

### Validation of invasion region criteria for statistical models & progress of *Clethrionomys glareolus* invasion

**Fig 3:**
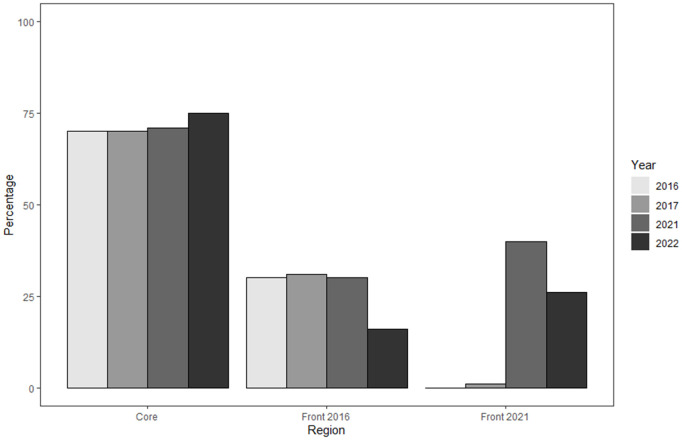
Percentage *Clethrionomys glareolus* relative to *Apodemus sylvaticus* in each region for each year. Data for 2016 and 2017 includes all 9 sites.

In the 2016 data for the invasion gradient analysis, the percentage of *A. sylvaticus* relative to *C. glareolus* was directly correlated with the region of invasion. The core had the lowest percentage of *A. sylvaticus* relative to *C. glareolus,* followed by front 2016, and finally front 2021 was completely uninvaded, with 100% *A. sylvaticus* (Kruskal Wallis, chi^2^ = 271.99, df = 2, *p* < 0.001; Dunn’s test post-hoc analysis, All regions, *p* < 0.001) (Fig 3). Our capture data thus confirms the invasion categories initially designed.

In the longitudinal data, the core had a lower percentage of *A. sylvaticus* relative to *C. glareolus* when compared to both invasion front regions, front 2016 and front 2021. However, the front 2021 was rapidly invaded in 2021 and 2022 and consequently no difference was observed between the percentage of *A. sylvaticus* in front 2016 and front 2021 (Kruskal Wallis, chi^2^ = 606.57, df = 2, *p* < 0.001; Dunn’s test post-hoc analysis, Core – Front 2016, *p* < 0.001; Core – Front 2021, *p* < 0.001; Front 2016 – Front 2021, *p* = 0.1039) (Fig 3).

### Invasion gradient analysis

#### Pathogen richness.

The best supported model (lowest AICc value; 813.979) explaining variations in the species richness of potentially pathogenic taxa between *C. glareolus* and *A. sylvaticus* in 2016 included only rodent species (Table 3). *C. glareolus* (x̅ = 0.46) was shown to have a lower mean number of pathogen species compared to *A. sylvaticus* (x̅ = 1.37) (GLMM, family = Zero inflated negative binomial, *z* = -8.068, *p* < 0.001). However, poor model fit was still observed when examining residuals, hence nonparametric tests were carried out to confirm these findings. Using a Wilcoxon Rank Sum test, a significant difference in species richness between *C. glareolus* and *A. sylvaticus* was confirmed, with the invasive *C. glareolus* (med = 0) having a lower median number of pathogens compared to *A. sylvaticus* (med = 1) (*W* = 23262, *p* < 0.001).

**Table 3:** Summary of significant factors in pathogen species richness and pathogens prevalence detected when looking at both species combined, *Apodemus sylvaticus* independently and pathogen prevalence in *Clethrionomys glareolus* independently.

	Statistically significant factors observed in invasion gradient analysis (2016)	Statistically significant factors observed in longitudinal analysis (2016 - 2022)
**Both Rodent Species**		
**Pathogen Species Richness**	Species (Voles)***	NA
***Apodemus sylvaticus***	**n = 203**	**n = 500**
**Pathogen Species Richness**	Sex (M)*	NA
***Bartonella***	Season (Spring)**, Sex (M)*	**Full:** Season(Spring)***, Sex(M)* Sp: Time*
***Mycoplasma haemomuris***	Age class (Mature)***	Season(Spring)**, Region(Front 2016)**, Region(Front2021)**, Age class(Juvenile)***, Age class (Mature)*
***Mycoplasma coccoides***	Age class (Mature)*	Time*, Age class(Mature)**
***Clethrionomys glareolus***	**n = 148**	**n = 411**
**Sarcocystidae**	Region (Front 2016)**	**Full:** Time***, Season(Spring)***, Region(Front 2021)*, Sex(M)*, Age class(Mature)* Sp: Region(Front 2016)*, Region(Front 2021)***, Age class(Mature)* Au: Time***, Sex(M)*

Full = full model including all years and seasons. In some instances, per season models were ran due to multicollinearity, this is indicated by Sp = spring only model, Au = autumn only model. Time indicates years as a numerical value since baseline, with 2016 = 1. n represents sample sizes in the full model for each analysis. Statistically significant factors include the associated variable (level) in brackets. Asterisks indicate level of significance, with * = *p* < 0.5, ** = *p* < 0.01, and *** = *p* < 0.001.

When focusing on *A.* sylvaticus only, using the invasion gradient analysis, no effect for the *C. glareolus* invasion was observed on the species richness of potentially pathogenic taxa. The best supported model (lowest AICc value; 565.089) included only sex (Table 3). Sex was shown to be statistically significant with males (x̅ = 1.54) having a higher mean pathogen species richness compared to females (x̅ = 1.14) (GLMM, family = Zero inflated negative binomial, *z* = 2.391, *p* = 0.017). As before, due to poor model fit, these findings were confirmed using a Wilcoxon Rank Sum test, showing males (med = 2) have a higher median species richness compared to females (med = 1) (*W* = 3830.5, *p* < 0.01).

#### Apodemus sylvaticus.

Below, we present the results of the invasion-gradient analyses for *Bartonella*, *M. haemomuris* and *M. coccoides* in *Apodemus sylvaticus* sampled in Ireland.

#### Bartonella.

Considering the invasion gradient analysis, the best supported model (lowest AICc value; 271.876) included season and sex (Table 3). *Bartonella* prevalences were higher in autumn compared to spring (GLMM, family = Binomial, *z* = -2.741, *p* = 0.00612), while males were more infected than females (GLMM, family = Binomial, *z* = 2.404, *p* < 0.05).

#### Mycoplasma haemomuris.

Considering the invasion gradient analysis, the best supported model (lowest AICc value; 268.721) included only age class (Table 3). No effect for region was observed. Age class was statistically significant (Age class: Mature, GLMM, family = Binomial, *z* = 3.405, *p* < 0.001). Post-hoc analysis showed that mature adults had significantly higher prevalences compared to young adults and juveniles. No difference between young adults and juveniles was observed, possibly due to the low number of juveniles in the dataset (n = 9) (Tukey Kramer analysis; Mature – Young adults, *p* < 0.01; Mature – Juveniles, *p* < 0.05; Young adults – Juveniles, *p* = 0.062).

#### Mycoplasma coccoides.

For the invasion gradient analysis, the best supported model (lowest AICc value from MuMIn; 285.4) included only the null model, therefore the second lowest AICc model (285.486) was used, including only age class and sex (Table 3). Age class was the only statistically significant variable (Age class: Mature, GLMM, family = Binomial, *z* = -2.115, *p* < 0.05). However, during post-hoc analysis no statistical differences was observed between pairs of age class (Tukey Kramer analysis; Mature – Young adults, *p* = 0.24; Mature – Juveniles, *p* = 0.92; Young adults – Juveniles, *p* = 0.89). No effect of the *C. glareolus* invasion gradient on *M. coccoides* prevalence was observed, as region was excluded from the lowest AICc model.

#### Clethrionomys glareolus.

Regarding *C. glareolus* in Ireland*,* the patterns of infection with Sarcocystidae observed during the invasion gradient were examined.

### Sarcocystidae

**Fig 4:**
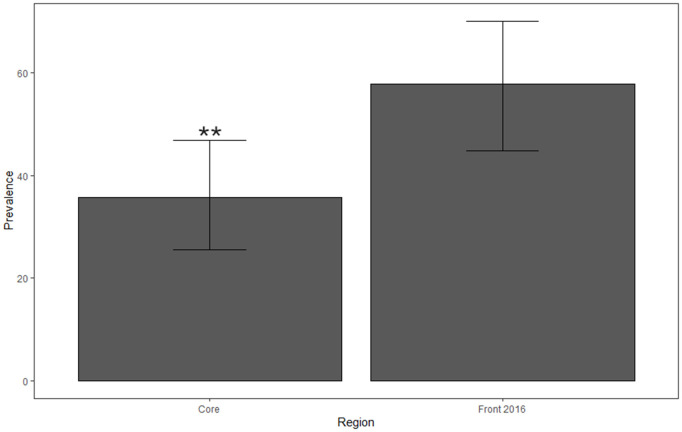
Prevalence of Sarcocystidae in *Clethrionomys glareolus* per region in 2016. Error bars represent 95% confidence intervals. Asterisks indicate level of statistical significance (** = p < 0.01), with the core being significantly different from Front 2016.

Considering the invasion gradient analysis on the prevalence of Sarcocystidae in *C. glareolus,* the best supported model (lowest AICc value; 202.816) included only region (Table 3)*.* The invasion front (front 2016) showed a significantly higher prevalence when compared to the core (Region: Front 2016, GLMM, family = Binomial, *z* = 2.652, *p* < 0.01) (Fig 4). Similarly, the same invasion gradient analysis was performed on a single OTU of Sarcocystidae specific to *C. glareolus*. The best supported model (lowest AICc value; 201.422) included region and age class. Region showed the same pattern as the analysis on all Sarcocystidae, with higher prevalence of this Sarcocystidae OTU in the front 2016 compared to the core (Region: Front 2016, GLMM, family = Binomial, *z* = 2.187, *p* < 0.05). Age class was not statistically significant (Age class: Mature, GLMM, family = Binomial, z = -1;871, *p* = 0.0613; Age class: Juvenile, GLMM, family = Binomial, z = 0.453, *p =* 0.65).

### Longitudinal analyses

#### Apodemus sylvaticus.

Below we present the patterns of infections observed in the invasion gradient analyses for the pathogen species *Bartonella, M. haemomuris* and *M. coccoides* in *A. sylvaticus* sampled in Ireland

#### Bartonella.

In the longitudinal analysis, the best supported model (lowest AICc value; 672.6) included time, season, region, and sex, with an interaction between time and season (Table 3). The interaction between time and season was shown to have a high variance inflation factor (VIF), meaning the covariates were correlated and potentially affecting the model through multicollinearity. To account for this, the interaction was removed in the full model (AICc 674.665), and separate GLMM’s were conducted for each season. In the full model, no effect for time or region was observed (GLMM, family = Binomial, z = 1.904, *p* = 0.057). *A. sylvaticus* in autumn showed higher prevalences of *Bartonella* than in spring (GLMM, family = Binomial, *z* = -3.708, *p* < 0.001). Alongside this, males exhibited higher prevalences compared to females (GLMM, family = Binomial, *z* = 2.241, *p* < 0.05).

In the seasonal models, the best supported model (lowest AICc value; 354.832) for spring included time and sex. No effect for region was observed (Table 3). Prevalence was positively correlated (correlation coefficient; 0.15) with time (GLMM, family = Binomial, *z* = 2.344, *p* = 0.019; Pearsons’s correlation test, *t* = 2.4751, *p* < 0.05). Sex did not significantly influence the prevalence of *Bartonella* in *A. sylvaticus* in the spring only model (GLMM, family = Binomial, z = 1.942, *p* = 0.052). In the autumn only analysis, the best supported model included only region (lowest AICc value; 322.788), however, no factor significantly influenced *Bartonella* prevalence.

#### Mycoplasma haemomuris.

**Fig 5:**
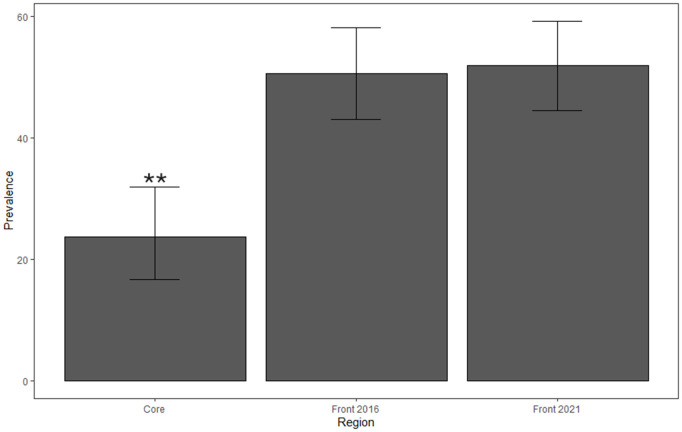
Prevalence of *Mycoplasma haemomuris* in *Apodemus sylvaticus* per region in the longitudinal analysis (all years). Error bars represent 95% confidence intervals. Asterisks indicate level of statistical significance (** = p < 0.01), with the core being significantly different from Front 2016 and front 2021, and with no statistical difference between Front 2016 and Front 2021.

In the longitudinal analysis, no effect for time was observed, and the best supported model selected (lowest AICc value; 626.339) included the factors season, region, and age class (Table 3). Region was shown to have a significant effect on the prevalence of *M. haemomuris* (Region: Front 2016, GLMM, family = Binomial, *z* = 2.744, *p* < 0.01; Region: Front 2021, GLMM, family = Binomial, *z* = 2.874, *p* < 0.01). Post-hoc analysis demonstrated that the core had a lower prevalence of *M. haemomuris* compared to front 2016 and front 2021, with no difference observed between front 2016 and front 2021 (Fig 5) (Tukey Kramer analysis; Core – Front 2016, *p* < 0.001; Core – Front 2021, *p* < 0.001; Front 2016 – Front 2021, *p* = 0.96). Secondly, spring had higher prevalences compared to autumn (GLMM, family = Binomial, *z* = 2.987, *p* < 0.01). Finally, age class was shown to be a statistically significant factor (Age class: Juvenile, GLMM, family = Binomial, *z* = -3.414, *p* < 0.001; Age class: Mature, GLMM, family = Binomial, *z* = 2.146, *p* < 0.05). Post-hoc analysis showed mature adults were the most infected, followed by subadults, with juveniles least infected (Tukey Kramer analysis; Mature - Young adults, *p* < 0.01; Mature – Juveniles, *p* < 0.001; Juveniles – Young adults, *p* < 0.001).

#### Mycoplasma coccoides.

**Fig 6:**
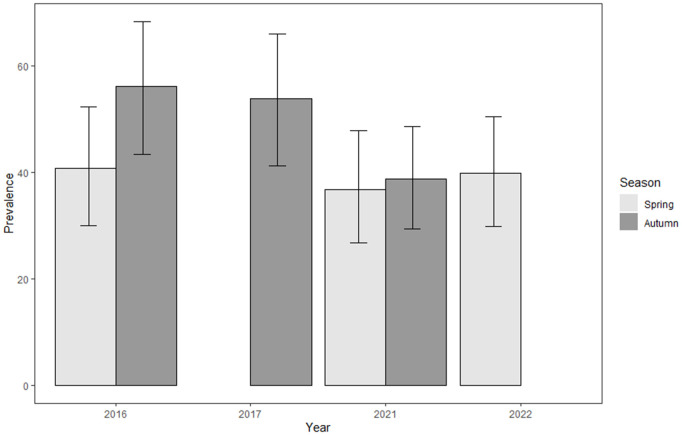
Prevalence of *Mycoplasma coccoides* in *Apodemus sylvaticus* for each season and year sampled. Error bars represent 95% confidence intervals.

The longitudinal analysis showed that the best supported model (lowest AICc value; 677.786) included time, sex and age class (Table 3). Time was shown to be a significant factor (GLMM, family = Binomial, *z* = -2.175, *p* < 0.05), with a negative correlation (-0.1) between prevalence and time (years) (Pearson’s correlation test, t = -2.2929, p < 0.05). This can be observed in Fig 6, where a reduction in prevalence is seen over time. Age class was also shown to affect prevalence (Age class: Mature, GLMM, family = Binomial, *z* = -3.04, *p* < 0.01). Post-hoc analysis demonstrated young adults being more infected than mature adults, however, no statistical differences were observed between mature adults and juveniles and between juveniles and young adults (Tukey Kramer analysis; Young adults – Mature, *p* < 0.05; Mature – Juveniles, *p* = 0.97; Young adults – Juveniles, *p* = 0.097).

#### Clethrionomys glareolus.

Below we present the patterns of infection of *C. glareolus* with Sarcocystidae observed during longitudinal analyses.

### Sarcocystidae

The longitudinal analysis of the prevalence of Sarcocystidae in *C. glareolus* found the best supported model (lowest AICc value; 460.593) included time, season, region, sex, age class and an interaction between time and season. A high VIF was observed for the interaction and hence it was removed from the final model (AICc value; 499.807 (Table 3). Time was shown to be statistically significant (GLMM, family = Binomial, *z* = -4.849, *p* < 0.001). Post-hoc analysis found that prevalence is negatively correlated (-0.22) with time (years since baseline) (Pearson’s correlation test; *t* = -4.6122, *p* < 0.001). Higher prevalence was observed in spring compared *t*o autumn (GLMM, family = Binomial, *z* = 3.547, *p* < 0.001). Region was shown to be statistically significant (Region: Front 2021, GLMM, family = Binomial, *z* = 2.143, *p* < 0.05). Post-hoc analysis demonstrates that the core had a lower prevalence compared to front 2016 but not statistically different from front 2021, while no statistical difference was observed between front 2016 and front 2021 (Tukey Kramer analysis; Core – Front 2016, *p* < 0.05; Core – Front 2021, *p* = 0.5; Front 2016 – Front 2021, *p* = 0.53). Females had higher prevalences than males (GLMM, family = Binomial, *z* = -2.216, *p* < 0.05). Finally, age class was shown to be a statistically significant factor (Age class: Mature, GLMM, family = Binomial, *z* = -2.322, *p* < 0.05). Post-hoc analysis showed that juveniles were more infected than mature adults. No statistical difference was observed between mature adults and young adults, or young adults and juveniles (Tukey Kramer analysis; Juveniles – Mature, *p* < 0.01; Juveniles – Young adults, *p* = 0.14; Mature – Young adults, *p* = 0.15).

Separate binomial GLMMs for each season were run on the longitudinal models to account for the removed interaction between season and time (Table 3). In spring, the best supported model (lowest AICc value; 232.189) included region and age class. Region was shown to be statistically significant (Region: Front 2016, GLMM, family = Binomial, *z* = 2.188, *p* < 0.05; Region: Front 2021, GLMM, family = Binomial, *z* = 3.769, *p* < 0.001). Post-hoc analysis demonstrated that the core had a lower prevalence when compared to both front 2016 and front 2021, while no difference was observed in the post hoc analysis between the two front regions identified in 2016 and 2021 (Tukey Kramer analysis; Core – Front 2016 *p* < 0.01; Core – Front 2021 *p* < 0.001; Front 2016 – Front 2021, *p* = 0.92). Age class was also statistically significant (Age class: Mature, GLMM, family = Binomial, *z* = -2.112, *p* < 0.05). Post-hoc analysis showed juveniles and young adults more likely to be infected compared to mature adults, but no statistical difference was observed between juveniles and young adults (Tukey Kramer analysis; Mature – Juveniles, *p* < 0.01; Mature – Young adults, *p* < 0.05; Young adults – Juveniles, *p* = 0.14).

Finally, the best supported model (lowest AICc value; 224.497) for autumn included time, sex, and age class. No effect for region was observed. Time was shown to be statistically significant (GLMM, family = Binomial, *z* = -6.766, *p* < 0.001) with time being negatively correlated (-0.5) with prevalence (Pearsons’s correlation test, *t* = -8.5352, *p* < 0.001). Females had higher prevalences compared *t*o males (GLMM, family = Binomial, *z* = -2.231, *p* < 0.05). Age class did not significantly influence the prevalence of Sarcocystidae in this analysis (Age class: Mature, GLMM, family = Binomial, z = -0.848, *p* = 0.4; Age class; Juvenile, GLMM, family = Binomial, z = 1.727, *p* = 0.084).

## Discussion

The aim of this study was to investigate the effect that an invasive species may have on native pathogen communities, including zoonotic species, using the bio-invasion of Ireland by *C. glareolus* as a model system. While previous studies on rodent-borne microparasites in Ireland have focused on single bacterial species, such as *Bartonella* or *Borrelia* [70–72], this is the first time 16SrRNA metabarcoding techniques have been used to identify the full communities of potentially zoonotic pathogens, and their prevalence levels, that are circulating in Irish populations of *C. glareolus and A. sylvaticus*. Furthermore, this study has created an important baseline for rodent-borne disease studies in Ireland, and highlights new important areas of research to guide future research in Ireland, and potentially other invasion systems (e.g., [73]).

### Rodent-borne pathogens observed in Ireland

Firstly, we discovered that Irish rodents exhibit a depauperate pathogen richness, with OTUs relating to only four pathogenic bacterial species and one family of protozoans in sampled rodent populations. Although the species in the Sarcocystidae OTUs were not identified in the present study, comparison with published sequences using NCBI BLAST indicated these may contain pathogens of veterinary and medical importance, including *Toxoplasma, Eimeria* and *Neospora*. Finally, this study examined the importance of considering time when examining the effect biological invaders have on pathogen communities. For example, in contrast to a previous study by Telfer *et al.,* [72] we detected *Bartonella* in *C. glareolus* in Ireland, albeit at a low prevalence rate. This result was surprising as the previous study had postulated that *C. glareolus* in Ireland might be resistant to native strains [72]. Further molecular work is required to determine if the *Bartonella* observed during this study is the same species of *Bartonella* observed in mice during the study by Telfer *et al.,* [72].

It is also worth noting that despite being native, *A. sylvaticus* also exhibited a depauperate community of potentially pathogenic bacterial taxa when compared to French populations, with no *Anaplasma, Candidatus Neoehrlichia, Francisella* or *Orientia* observed in the Irish population. This is consistent with island biogeography theory, which states that species diversity is limited by their size and isolation from the mainland [74,75]. The absence of certain vector-borne pathogens might also be explained by the low vector diversity in Ireland. For example, reviews in Ireland and France have shown Ireland to have 13 species of ticks, compared to approximately 40 tick species in France [76,77]. However, assays specifically targeting these pathogens would need to be carried out to confirm their absence. Furthermore, *Borrelia* was also notably absent from Irish rodents in this study, although it had been detected, albeit at low prevalences, in Irish rodents by previous studies [70,71]. This discrepancy may again be a result of the methods and target organ used in the present study, with previous studies using ear biopsies and species specific PCR [70,71]. Also, while *Apodemus* species and *C. glareolus* are considered important aspects of the *Borrelia* life cycle in Europe, these rodents may play a limited role as reservoir in Ireland, with the rodent-associated, *Borrelia afzelii*, being less common in Ireland [70,71,78,79]. Additionally, the absence of *Anaplasma* from rodents sampled in the present study agree with findings reported by Flattery *et al.* [80], who screened questing nymphal *Ixodes ricinus* ticks collected across Ireland for the presence of *Anaplasma phagocytophilum* and only found groEL ecotype 1, a strain that is not thought to be associated with rodents.

### Enemy release in the invasive *Clethrionomys glareolus*

Two patterns consistent with the enemy release hypothesis were observed for bacterial pathogens in the invasive *C. glareolus* population in Ireland for the first time. Firstly, a comparison of the number of bacterial taxa in Irish and French *C. glareolus* populations indicated that the population of *C. glareolus* sampled in Ireland had fewer potentially pathogenic bacterial taxa than conspecifics in their native range. This can be seen particularly in the 2021 data, where the Irish *C. glareolus* individuals had fewer potentially pathogenic bacterial taxa and lower prevalences compared to *C. glareolus* populations in France. In particular, a number of potentially pathogenic taxa were completely absent from *C. glareolus* populations in Ireland including: *Anaplasma, Candidatus Neoehrlichia, Francisella and Orientia*. Further studies should be conducted to confirm this and incorporate consistent techniques in assigning age classifications in both countries allowing for more in-depth statistical analysis between samples from Ireland and France. Secondly, *C. glareolus* exhibited enemy release from potentially pathogenic bacterial taxa in comparison to the native *A. sylvaticus* in Ireland. While *C. glareolus* was found to have one more bacterial taxon when compared to *A. sylvaticus, C. glareolus* has lower prevalences of these bacterial taxa in the overall data, and a significantly lower pathogen species richness compared to *A. sylvaticus* in the invasion gradient analysis (2016 only). Consistent with these findings, both *Bartonella* and *M. haemomuris* occurred at significantly lower prevalences in *C. glareolus* populations in Ireland compared with those in France.

These findings are comparable to studies investigating the helminth communities in the invasive *C. glareolus* in Ireland [23,39]. These authors found the same two examples of enemy release, i.e., *C. glareolus* had a lower helminth species richness compared to its native range, and to the native *A. sylvaticus* in Ireland [23,39]. Likewise, similar patterns have been observed in other invasion systems. For example in the Senegal invasion system, Diagne *et al.* [24] demonstrated that native rodents (*Mastomys erythroleucus* and *Mastomys natalensis*) had a greater species richness of pathogenic bacterial OTUs when compared to non-native rodents (*Mus musculus domesticus* and *Rattus rattus*). These findings from investigating invasion systems in different countries and invasive species can provide insights into the underlying mechanisms contributing to how these species survive, establish and outcompete native fauna when colonising new environments. For example, this pattern of enemy release could allow the invasive species to direct more energy from immune responses into growth or fecundity, potentially giving invasive species a competitive advantage over native species [81].

Stuart *et al.* [39] described a third example of enemy release in the Irish population of *C. glareolus,* namely the reduced abundance of the directly transmitted nematode *A. tianjinsensis* in *C. glareolus* in the invasion front compared to the invasion core in spring and autumn in 2016 [39]. A similar example of enemy release at the invasion front was not found during this present study. In contrast, in the invasion gradient analysis, *C. glareolus* was more likely to be infected in the invasion front, compared to the more established invasion core, with significantly higher prevalences of Sarcocystidae in front 2016 compared to the core. This pattern was also observed in 2016 for the single Sarcocystidae OTU which seems to be specific to *C. glareolus*, with no *A. sylvaticus* found infected by this OTU in this study. These findings are in direct contrast to the enemy release hypothesis, as the more recently invaded populations exhibited higher infection rates, highlighting that infection patterns may be highly pathogen specific. However, interpretation of these patterns should be treated with caution, as 16S rRNA metabarcoding does not allow reliable species level resolution within the Sarcocystidae, and different species may vary in transmission dynamics.

This result was even more surprising given that male *C. glareolus* at the invasion front are thought to be more timid and less exploratory than their conspecifics at core sites [82], characteristics which would be expected to reduce their exposure to pathogens. On the other hand, there also seems to be a strong link between spatial behaviour and behaviour traits, with shy but thorough explorers using larger areas [82]. If animals are exploring larger areas at the expansion front, this could potentially result in greater exposures to pathogens.

Finally, a possible explanation for the increased infection rate at the invasion front might lie in the immunology of *C. glareolus* during its invasion of Ireland. Pathogens may lag behind biological invasions, as the population density of the invading species at the front is too low to maintain sufficient transmission. Consequently, the reallocation of resources from immune responses, known as the evolution of increased competitive ability hypothesis and enemy release from pathogens, could be favoured in invasive populations [22,47]. This could potentially leave *C. glareolus* in the invasion front relatively immunologically naïve and hence, susceptible to increased Sarcocystidae infection when the population increases over a certain threshold. This would be further supported by the findings of the longitudinal analysis, that juveniles have a higher prevalence of Sarcocystidae compared to mature adults, as juveniles would potentially be more immunologically naïve. In a study of the population genomics of *C. glareolus* in Ireland, White *et al.* [83] identified the selection of genes with immunological function in *C. glareolus* furthest from the point of introduction. However, to fully understand these mechanisms, further investigations of the eco-immunology of invasive *C. glareolus* populations, and identification of the species present within these Sarcocystidae OTUs are required.

### Effect of *Clethrionomys glareolus* invasion on pathogen communities in the native *Apodemus sylvaticus*

It was hypothesised that the presence of *C. glareolus* might affect the pathogen communities in the native *A. sylvaticus*. However, no dilution or amplification effect resulting from the *C. glareolus* invasion gradient on pathogen species richness in *A. sylvaticus* was observed in the present study. Likewise, in the invasion gradient analysis (2016 data only), no effect for invasion region was found on the prevalence of *A. sylvaticus’* pathogens. However, using the longitudinal data, the increased relative abundance of *C. glareolus* at the site of introduction apparently caused a dilution effect for *M. haemomuris* in *A. sylvaticus*. In this instance the core showed significantly reduced prevalences of *M. haemomuris* in *A. sylvaticus* compared to conspecifics from the invasion front regions (front 2016 and front 2021). No difference was observed between front 2016 and front 2021 indicating that the reduction in *M. haemomuris* prevalence may be directly attributable to the higher *C. glareolus* abundance at the core.

In a previous study investigating the impact of forest anthropization on small mammal pathogens, Bouilloud [84] detected a dilution effect for *M. haemomuris* (Strain 2) associated with increased small mammal diversity in France. The author argued that the declining abundance of its most competent host, *C. glareolus,* as host species diversity increased, might explain the dilution of *M. haemomuris* at these sites.

The dilution of *M. haemomuris,* in *A. sylvaticus* in Ireland (associated with the presence of *C. glareolus*), and the dilution of *M. haemomuris* (strain 2) in France (presumed to be due to an increase in small mammal diversity), demonstrate that multiple factors (such as climate change, invasive species, deforestation and urbanisation) can alter rodent populations and consequently pathogen prevalences. Understanding the underlying mechanisms driving these patterns can therefore improve predictions of disease transmission and inform disease management strategies. Interestingly, the dilution pattern identified for *M. haemomuris* was not observed for *M. coccoides*. The driver behind the different patterns of these *Mycoplasma* spp. may possibly be a result of the specific behaviour of the respective vector species, *Polyplax serrata* in the case of *M. coccoides* and *Polyplax spinulosa* in the case of *M. haemomuris* [85,86]. However, studies on these pathogens vector species are based on mice and rats’ laboratory experiments only. Therefore, field-based studies are needed to confirm the possibility that *C. glareolus* might be a less favourable host for the vector transmitting *M. haemomuris*, or the bacteria itself, in Ireland, which could result in a dilution effect in the native *A. sylvaticus.*

Surprisingly, we detected no dilution effect for *Bartonella* in *A. sylvaticus* in this present study. *Bartonella birtlesii* and *Bartonella taylorii* have previously been recorded in Ireland, with prevalences in *A. sylvaticus* decreasing with increased *C. glareolus* densities [72]. These seemingly contradictory observations may be due to the time that has elapsed since the study by Telfer *et al.* [72] and the present study. It is possible for example, that the initial invasion of *C. glareolus* disrupted these pathogens, but they have recovered since. On the other hand, only a single *C. glareolus* individual was found to be infected with *Bartonella* here, corroborating the suggestion that *C. glareolus* in Ireland is not a competent host for this pathogen species. Hence, the observed difference may stem from the detection techniques employed, as Telfer *et al.* [72] utilized bacterial cultivation and genus-specific PCR assays. In contrast the present study only found one OTU of *Bartonella,* using 16SrRNA amplification and illumina sequencing an approach that may have limited sensitivity for resolving Bartonella species or strains compared with genus-specific assays for example using gltA and rpoB gene sequencing. Nevertheless, the consistently low detection of Bartonella in *C glareolus* across sites and years suggests that host competence differences may still contribute to the observed patterns. Further work, using genus specific primers, such as those used by Telfer *et al.* [72] would be needed to examine *Bartonella* positive rodents to determine species identity, and hence identify whether dilution does still occur in the Irish *A. sylvaticus* population.

### Changes in rodent pathogen communities over time

Finally, the effect of time on the prevalence of the various pathogenic taxa, as the *C. glareolus* invasion progressed was examined. Prevalence decreased between 2016 and 2022, for both *M. coccoides* in *A. sylvaticus* and Sarcocystidae in *C. glareolus*. However, examining the spring longitudinal data only, *Bartonella* prevalence in *A. sylvaticus* increased between 2016 and 2022. These patterns show that pathogen communities are dynamic, and longitudinal studies are essential for fully understanding the changes that occur in pathogen communities. Cornet *et al.* [22], expressed the need for longitudinal studies to examine the time-lag for invasive species between invasion and adapting to the invasion front. A recent review by Brian and Catford [87] highlighted that time since introduction is a significant factor when investigating the enemy release hypothesis, as enemy impact on the invader will potentially increase with time, as pathogens adapt to the invasive species. In our case, the dilution effect for *M. haemomuris* in *A. sylvaticus* was only observed in the longitudinal data, an observation that could potentially have been missed in a single timepoint study. Moreover, *M. haemomuris, M. penetrans* and *Bartonella* were not detected in *C. glareolus* in the baseline 2016 data. While this could be attributed to the increased sample size afforded by multiple years of sampling, focusing on a single year, could have resulted in missing the dilution for *M. haemomuris* in *A. sylvaticus* and the assumption that *C. glareolus* is not infected by these pathogens in Ireland. Similarly, the recently reported *Babesia microti* ‘Munich’ strain in Ireland was detected exclusively in *C. glareolus* located in the eastern part of the country, which corresponds to the *C. glareolus* invasion front [73]. The primary vector of this strain, *Ixodes trianguliceps*, has also been mainly reported in eastern Ireland [88]. If the assumption that *B. microti* is confined to eastern regions of Ireland is correct, and if sampling of *C. glareolus* had occurred prior to the invasion reaching this area, it is possible that this pathogen would not have been detected and that *B. microti* would have been assumed to be absent from Ireland. This further demonstrates the importance of long-term studies of pathogens in invasive species, particularly for detecting the emergence of pathogens in newly colonised areas. Finally, more long-term data is needed to fully understand if rodents and their pathogens fluctuate through multi-annual cycles, which is seen in other countries where long-term data is gathered, such as Finland [89–91].

## Conclusion

In conclusion, patterns consistent with the enemy release hypothesis were observed in the Irish *C. glareolus* population. Despite exhibiting higher prevalences of Sarcocystidae, *C. glareolus* in Ireland shows reduced pathogen species richness, and lower prevalences of bacterial taxa, compared to the native *A. sylvaticus,* and fewer bacterial taxa compared to the native range of the species. These findings increase our understanding of the mechanism of how invasive species thrive in new environments and outcompete native fauna, however, eco-immunological data are required to fully understand these patterns of enemy release. Secondly, the present study showed the disruptive effect of non-native host species on native pathogen systems, with the example of a dilution effect and reduced prevalence of *M. haemomuris* in *A. sylvaticus* at the invasion core compared to invasion fronts, where *C. glareolus* populations exhibit higher abundance. These findings show how changes in biodiversity, such as the addition of new hosts with varying levels of competencies for a given pathogen, may disrupt pathogen communities and host-pathogen dynamics. Further work is also needed to identify the Sarcocystidae and *Bartonella* species detected in this study in order to fully understand the pathogen community dynamics, as genetic differences may reveal different levels of host specificity and help explain host-pathogen interactions. Finally, time, as the *C. glareolus* invasion progresses, was shown to be correlated, both positively and negatively, with three pathogen taxa during this study, illustrating that invasion systems are dynamic, and pathogen communities can fluctuate as the invader becomes more established. By conducting a longitudinal investigation, we demonstrated that long-term data are essential to fully understand eco-evolutionary processes driving invasion dynamics and success, preventing conclusions drawn from single timepoint studies being assumed as long-term changes in pathogen communities. Disease dynamics during biological invasions cannot be generalised across all host–pathogen associations, nor reliably predicted from a single stage of invasion. Instead, long‑term investigations employing broad, multi‑pathogen approaches are required to capture the dynamic and pathogen‑specific nature of disease processes as invaders establish and spread.

## Supporting information

S1 Table Field work dates. This details the date in which field work was conducted.
(XLSX)

S2 Table Prevalence summary region and year. This provides a summary of the pathogen prevalences per host species for each invasion region and year during this study.
(XLSX)

S3 Table The sequencing of spleens from the 1,082 rodents from Ireland presented in this study was carried out between 2016 and 2022, sometimes including samples from other countries outside the scope of this study, and the sequencing data were analysed in three stages over different periods. Details of the sequencing process are presented in the file ‘Sequencing_informations_spleen_samples.xlsx’ on Zenodo (https://zenodo.org/records/14883529).
(XLSX)
