# Supplementary material for: Longitudinal study of a rodent invasion reveals shifts in host pathogen interactions
Source: PLoS Pathog. 2026 Jul 23;22(7):e1014349. doi: 10.1371/journal.ppat.1014349 (PMC13395368; doi:10.1371/journal.ppat.1014349)
Supplement: Attachment — Submitted filename: PPATHOGENS-D-25-01038.pdf [file ppat.1014349.s004.pdf]

PLOS Pathogens

Longitudinal study of a rodent invasion reveals shifts in host pathogen interactions.

--Manuscript Draft--

Manuscript Number:	PPATHOGENS-D-25-01038
Full Title:	Longitudinal study of a rodent invasion reveals shifts in host pathogen interactions.
Short Title:	Longitudinal study of a rodent invasion reveals shifts in host pathogen interactions.
Article Type:	Research Article
Section/Category:	Evolutionary Biology - Bacteria
Keywords:	Zoonotic pathogens, epidemiology, rodents, enemy release, 16S rRNA metabarcoding, biological invasions
Abstract:	Invasive species can disrupt native epidemiological processes, potentially leading to the emergence of zoonotic pathogens. The relatively recent appearance and spread of the bank vole (Clethrionomys glareolus) in Ireland provides a unique model system to study these phenomena. The depauperate small mammal community in Ireland, combined with baseline data, allows us to identify the invasion gradient of C. glareolus and its effects on rodent-borne pathogens over time. A total of 498 C. glareolus and 584 Apodemus sylvaticus were sampled across nine sites in Ireland in 2016 and 2017, with six sites revisited in 2021 and 2022. 16S rRNA metabarcoding identified 10 putative pathogenic Operational Taxonomic Units (OTUs) present in these rodents, relating to four bacterial taxa, Bartonella spp., Mycoplasma coccoides, Mycoplasma haemomuris and Mycoplasma penetrans, and one family of protozoans, Sarcocystidae. Several epidemiological processes were found to be correlated with the C. glareolus invasion. C. glareolus in Ireland exhibited patterns consistent with the enemy release hypothesis, compared to native populations in France, and the native A. sylvaticus in Ireland. Likewise, a potential dilution effect, with reduced prevalence of M. haemomuris in A. sylvaticus at the C. glareolus invasion core compared to the invasion front, was observed. Finally, C. glareolus had increased prevalence of Sarcocystidae at the invasion front when compared to the invasion core, depending on the time and stage of invasion. These findings further our understanding of pathogen dynamics during biological invasions, demonstrating that invaders affect native host-pathogen communities differently as they advance through various stages of establishment.
Additional Information:	
Question	Response
Government Employee<br><br>Are you or any of the contributing authors an employee of the United States government?<br><br>Manuscripts authored by one or more US Government employees are not copyrighted, but are licensed under a CC0 Public Domain Dedication, which allows unlimited distribution and reuse of the article for any lawful purpose. This is a legal requirement for US Government employees.	No - No authors are employees of the U.S. government.

This will be typeset if the manuscript is accepted for publication.	
**Financial Disclosure**<br><br>Enter a financial disclosure statement that describes the sources of funding for the work included in this submission. Review the submission guidelines for detailed requirements. View published research articles from *PLOS Pathogens* for specific examples.<br><br>This statement is required for submission and **will appear in the published article** if the submission is accepted. Please make sure it is accurate.<br><br>**Funded studies**<br>Enter a statement with the following details:<br>• Initials of the authors who received each award<br>• Grant numbers awarded to each author<br>• The full name of each funder<br>• URL of each funder website<br>• Did the sponsors or funders play any role in the study design, data collection and analysis, decision to publish, or preparation of the manuscript?<br><br>Did you receive funding for this work?	Yes
Please add funding details.<br>as follow-up to "**Financial Disclosure**<br><br>Enter a financial disclosure statement that describes the sources of funding for the work included in this submission. Review the submission guidelines for detailed requirements. View published research articles from *PLOS Pathogens* for specific examples.<br><br>This statement is required for submission and **will appear in the published article** if the submission is accepted. Please make sure it is accurate.	This research was funded through the 2018–2019 BiodivERsA joint call for research proposals, under the BiodivErsA3 ERA-Net COFUND programme, and with the funding organisations the EPA Research Programme 2021–2030 and cofunded by Agence Nationale de la Recherche (ANR), Research Foundation– Flanders (FWO), National Science Centre, Poland (NCN) and Deutsche Forschungsgemeinschaft (DFG) (https://www.biodiversa.eu/ AMcM, MG, NC,CH, VS, AZ, PS).<br>This project is funded under the EPA Research Programme 2021-2030. The EPA Research Programme is a Government of Ireland initiative funded by the Department of the Environment, Climate and Communications (project number 2019-NC-MS-10) (https://www.epa.ie/ AMcM).<br>2016 and 2017 sampling was funded by the Irish Research Council, Government of Ireland Postdoctoral Fellowship Programme and by the Marie Curie Research Grants Scheme, European Union, grant (749312, INVASIMMUN) (https://research.ie/ https://marie-sklodowska-curie-actions.ec.europa.eu/ PS).

Powered by Editorial Manager® and ProduXion Manager® from Aries Systems Corporation

**Funded studies**<br>Enter a statement with the following details:<br>• Initials of the authors who received each award<br>• Grant numbers awarded to each author<br>• The full name of each funder<br>• URL of each funder website<br>• Did the sponsors or funders play any role in the study design, data collection and analysis, decision to publish, or preparation of the manuscript?<br><br>Did you receive funding for this work?"	
Please select the country of your main research funder (please select carefully as in some cases this is used in fee calculation).<br>as follow-up to "**Financial Disclosure**<br><br>Enter a financial disclosure statement that describes the sources of funding for the work included in this submission. Review the submission guidelines for detailed requirements. View published research articles from *PLOS Pathogens* for specific examples.<br><br>This statement is required for submission and **will appear in the published article** if the submission is accepted. Please make sure it is accurate.<br><br>**Funded studies**<br>Enter a statement with the following details:<br>• Initials of the authors who received each award<br>• Grant numbers awarded to each author<br>• The full name of each funder<br>• URL of each funder website<br>• Did the sponsors or funders play any role in the study design, data collection and analysis, decision to publish, or preparation of the manuscript?<br><br>Did you receive funding for this work?"	IRELAND - IE
**Competing Interests**	Authors declare no conflict of interest

On behalf of all authors, disclose any competing interests that could be perceived to bias this work.<br><br>This statement will be typeset if the manuscript is accepted for publication.<br><br>Review the instructions link below and PLOS Pathogens' competing interests policy to determine what information must be disclosed at submission.	
**Data Availability**<br><br>Provide a **Data Availability Statement** in the box below. This statement should detail where the data used in this submission can be accessed. This statement will be typeset if the manuscript is accepted for publication.<br><br>Before publication, authors are required to make all data underlying their findings fully available, without restriction. Review our PLOS Data Policy page for detailed information on this policy. Instructions for writing your Data Availability statement can be accessed via the Instructions link below.	Supplementary material includes raw data

Powered by Editorial Manager® and ProduXion Manager® from Aries Systems Corporation

Manuscript

Click here to access/download;Manuscript;16s OTU analysis PLOS Pathogens submission.docx

Title:

Longitudinal study of a rodent invasion reveals shifts in host pathogen interactions.

Short Title:

Longitudinal study of a rodent invasion reveals shifts in host pathogen interactions.

Author Information:

Andrew McManus[1], Maxime Galan[2], Nathalie Charbonnel[2], Celia Holland[3], Vincent Sluydts[4], Annetta Zintl[5], Peter Stuart[1].

[1] Munster Technological University, Department of Biological and Pharmaceutical Sciences, Tralee, Co. Kerry, Ireland.

[2] CBGP, INRAE, CIRAD, Institut Agro, IRD, Univ Montpellier, Montpellier, France.

[3] Trinity College Dublin, the University of Dublin, Department of Zoology, College Green, Dublin 2, Ireland.

[4] University of Antwerp, Department of Biology, Evolutionary Ecology Group, University of Antwerp, 2610 Wilrijk, Belgium.

[5] University College Dublin, School of Veterinary Medicine, Veterinary Science Centre, Belfield, Dublin 4, Ireland.

Corresponding Author:

Peter Stuart; peter.stuart@mtu.ie

Abstract

Invasive species can disrupt native epidemiological processes, potentially leading to the emergence of zoonotic pathogens. The relatively recent appearance and spread of the bank vole (*Clethrionomys glareolus*) in Ireland provides a unique model system to study these phenomena. The depauperate small mammal community in Ireland, combined with baseline data, allows us to identify the invasion gradient of *C. glareolus* and its effects on rodent-borne pathogens over time. A total of 498 *C. glareolus* and 584 *Apodemus sylvaticus* were sampled across nine sites in Ireland in 2016 and 2017, with six sites revisited in 2021 and 2022. 16S rRNA metabarcoding identified 10 putative pathogenic Operational Taxonomic Units (OTUs) present in these rodents, relating to four bacterial taxa, *Bartonella spp., Mycoplasma coccoides, Mycoplasma haemomuris and Mycoplasma penetrans,* and one family of protozoans, Sarcocystidae. Several epidemiological processes were found to be correlated with the *C. glareolus* invasion. *C. glareolus* in Ireland exhibited patterns consistent with the enemy release hypothesis, compared to native populations in France, and the native *A. sylvaticus* in Ireland. Likewise, a potential dilution effect, with reduced prevalence of *M. haemomuris* in *A. sylvaticus* at the *C. glareolus* invasion core compared to the invasion front, was observed. Finally, *C. glareolus* had increased prevalence of Sarcocystidae at the invasion front when compared to the invasion core, depending on the time and stage of invasion. These findings further our understanding of pathogen dynamics during biological invasions, demonstrating that invaders affect native host-pathogen communities differently as they advance through various stages of establishment.

Author summary:

When animals are introduced to new environments, they can disrupt local disease patterns by changing how pathogens spread among native wildlife. The spread of the bank vole, a non-native rodent in Ireland, offers a valuable model system for studying how such invasions affect disease dynamics over time. In this study, we examined how the arrival of the bank vole is influencing the health of the native wood mouse. We found that in areas where bank voles have been established longer, wood mice carry fewer infections from a common blood parasite. At the same time, bank voles themselves carried fewer types of pathogens overall than wood mice, possibly giving them an advantage in terms of survival and reproduction. Interestingly, we also observed that bank voles were more likely to carry a specific group of parasites, called Sarcocystidae, in areas they had only recently invaded. These patterns suggest that invading species can reshape disease dynamics in complex and

evolving ways. Understanding how these changes unfold over time is important for predicting future disease risks to wildlife and potentially to humans..

Keywords:

Zoonotic pathogens, epidemiology, rodents, enemy release, 16S rRNA metabarcoding, biological invasions

Introduction

The emergence of zoonotic pathogens has seen a sharp increase since the 1940's, with 60% of emerging infectious diseases originating from animals, including the recent outbreaks of SARS-CoV-2, Lassa, Ebola and Mpox viruses (1-7). This increase in the frequency of emerging infectious diseases, which poses a risk to humans, livestock and indigenous wildlife populations, has been primarily attributed to environmental change, caused by landscape change, a reduction of habitat diversity and the introduction of invasive species (8-16).

A recent review by Bezerra-Santos *et al.* (15), highlights that invasive mammal species pose increased risks for public health by introducing zoonotic pathogens (17, 18). However, non-native species can also benefit from a reduction in pathogens compared to their native range (and often compared to the native species), through an epidemiological process known as "enemy release". This can happen because the pathogen, or their vector, is absent from the founder population, or because the environment in the new range is unsuitable (19-23). This, in turn, may give invaders a competitive advantage over native species. Additionally, invasive species can influence host-pathogen ecological interactions in native hosts in several other ways. Firstly, invasive species can co-invade with new pathogens, which may infect native hosts, leading to a process known as pathogen spillover (24). The establishment of invasive species may also lead to a dilution of native pathogens, where the presence of a host with a lower competence for a specific pathogen can reduce the circulation of that pathogen in competent hosts (25-27). On the other hand, invasive host species can also have an increased competency for native pathogens, causing a spillback of native pathogens that amplifies infection in native hosts (28, 29).

Rodents, the largest order of mammals, make up one of the most common group of invasive species worldwide, affecting native ecosystems, food production and human health (30). Additionally, a recent review by Dharmarajan *et al.* (6), highlighted that rodents and bats harbour the highest proportion of zoonotic agents,

identifying these taxa as species of interest for studying zoonoses. Therefore, rodents make excellent models for studying the eco-epidemiology of pathogens due to their presence in large numbers and broad distributions, playing an important role as reservoirs for pathogens causing zoonotic disease; including among others, orthohantaviruses, *Leptospira* and *Bartonella* species, or *Toxoplasma gondii* (6, 31-35). Finally, studies on rodent invasions have demonstrated the interplay between rodent pathogen interactions and biological invasions (22, 29, 36, 37).

The case of the bank vole, *Clethrionomys glareolus* (formerly *Myodes glareolus*), invasion in Ireland presents a unique opportunity to explore changes in epidemiological dynamics during a biological invasion (35). This is particularly due to the depauperate woodland rodent community in Ireland, that includes the invasive *C. glareolus* and the native woodmouse, *Apodemus sylvaticus* (38). Species depauperate systems provide a valuable scenario to tease out the effects of invasive species on native pathogen communities. For example, in this instance the depauperate rodent community in Ireland has allowed for easier identification of the effects the *C. glareolus* invasion has on the native *A. sylvaticus* population in previous studies (29, 37). Furthermore, *C. glareolus* in Ireland has a single identifiable introduction point in the south west, near Foynes, Co. Limerick and the likely source population from Germany identified through mitochondrial DNA (39). In addition, studies have shown *C. glareolus* in Ireland to have an expansion rate of 2.23 – 2.63 km per year in unconstrained areas (40). This has allowed for the establishment of cross-sectional studies with an invasion gradient, containing sites from the invasion core (established from the introduction point), invasion front (sites where *C. glareolus* had most recently invaded), and uninvaded sites (*A. sylvaticus* only) (37). These factors combined, provide an excellent case study to identify the epidemiological effects that the *C. glareolus* invasion has on native pathogen communities in *A. sylvaticus*, such as spillover, dilution, or spillback.

The development of high-throughput sequencing, and in particular the metabarcoding technique, facilitates a holistic approach to studying the rodent pathobiome (41). The resulting global picture of pathogen diversity within hosts should improve our understanding of the interlinkages between host-parasite interactions and biological invasions. In the Irish context, rodent helminth parasite communities have been explored previously by Loxton *et al.* (21, 29) and Stuart *et al.* (37), but no such data exists on pathogenic microparasites in this model system. Similarly, there remains a knowledge gap surrounding longitudinal studies on pathogens during biological invasions. Such studies are essential for fully exploring changes in host-pathogen interactions, such as pathogen loss in invasive species, as cross-sectional studies only capture a single timepoint of this dynamic process (35, 37, 42-44).

In this study, we aim to describe the community of microparasites that could be zoonotic or pathogenic for rodents, including bacteria and protozoa, of *C. glareolus* and *A. sylvaticus* in Ireland. We performed a spatiotemporal analysis of this community along the invasion gradient described above, and rodents sampled in 2016 from a previous study by Stuart *et al.* (37), with subsequent sampling in 2017, 2021 and 2022. In this stu[illegible] we use the 16s rRNA amplicon metabarcoding approach developed by Galan *et al.* (45), and used in the recent study by Bouilloud *et al.* (46). Focusing on putative pathogenic taxa recorded in samples collected between 2016 and 2022, this present study addresses three main hypotheses: Firstly, whether *C. glareolus* exhibits patterns consistent with the enemy release hypothesis, including a lower pathogen species richness of potentially pathogenic taxa when compared to its native range and the native *A. sylvaticus,* secondly, whether the presence of *C. glareolus* alters the pathogen communities present in the native *A. sylvaticus* as expected under the dilution or spillback bypotheses, finally, whether pathogen prevalence changes over time in both rodent species, as the invader becomes established.

Materials & methods

Nine sites were selected for field analyses in 2016, as described in Stuart *et al.* (37). This included three invasion 'core' sites in Foynes (Foynes 1, 2 & 3), three 2016 invasion front sites ('front 2016', i.e. Moore Abbey, Carnpark, and St. John's), and three 2021 invasion front sites ('front 2021', i.e. Killinthomas, Loughkey and Donore, uninvaded in 2016) (Figure 1). In 2016, rodents were sampled at all of these nine sites in spring and autumn trapping sessions and revisited in autumn 2017. For the longitudinal aspect of this study, only six of these sites were revisited in spring and autumn 2021, and spring 2022 (Supplementary table 1). These sites included the 'core' sites in Foynes (Foynes 1 and 3, not Foynes 2), invasion front in 2016 ('front 2016', Moore Abbey and St. John's wood, not Carnpark) and the invasion front in 2021, ('front 2021', Killinthomas and Donore, not Loughkey) (Figure 1).

Longworth traps, baited using peanuts, bird seed mix and a piece of carrot, were placed 5-10m apart in transects of a maximum of 20 traps. A maximum of 24 animals from each species were taken from each site per season. The maximum sample size was calculated based on the estimated sample required to observe at least one infected individual based on a prevalence of 12% with 95% confidence intervals (*i.e.* a sample size of 24 should allow detection of all pathogens with a prevalence of 12% and above, allowing detection of rare pathogens). Traps were placed in the field overnight and checked early the next morning. Rodents were inspected in the field

and transported back to the laboratory. If insufficient numbers were trapped, the site was revisited for an additional night within the same season.

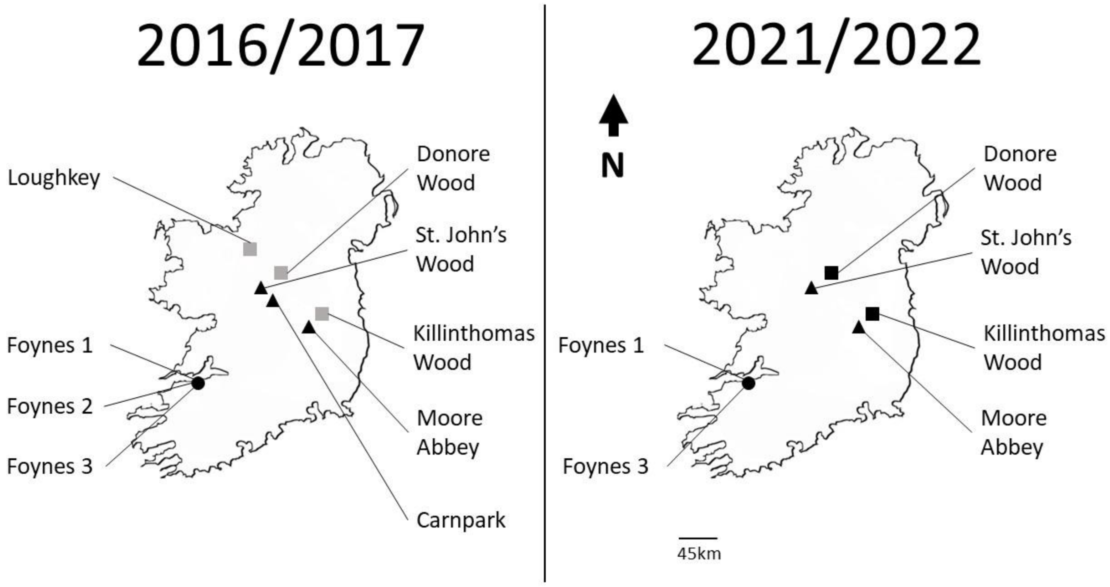

Figure 1. Map of Irish sampling sites visited in 2016, 2017, 2021 and 2022. Circles indicate the invasion core; triangles indicate the invasion front in 2016 and squares indicate the invasion front in 2021. The grey colour shows sites that were uninvaded by *Clethrionomys glareolus* on a given date, while the black colour shows sites that were invaded.

A total of 498 *C. glareolus* and 584 *A. sylvaticus* were sampled. Rodents were euthanised using Isoflurane (1000mg/g, under license from the Health Products Regulatory Authority). The spleens were removed immediately and stored in RNAlater at 4°C for 24 hours, then placed into the freezer at -20°C for further metabarcoding analysis. Rodents were classified into three age categories, juvenile, young adult and mature adult as described in Behnke *et al.* (47), using rodent length (nose to anus; in millimetres) and weight (grams) as described by Loxton *et al.* (21, 29) and used by Stuart *et al.* (37). For animals found to be at the limit of each classification, visual assessments of maturity were used as described by Stuart *et al.* (37).

The 16S metabarcoding analysis was conducted as described by Galan *et al.* (45). DNA was extracted from the rodent spleens using DNeasy 96 Tissue Kit (Qiagen, Germany). This organ was selected by Galan *et al.* (45), as the spleen is responsible for detecting and removing blood-borne microrganisms. Extracted DNA was screened for putative pathogenic Operational Taxonomic Units (OTUs) using the v4 hyper variable region of the 16S rRNA gene with universal primers (16S-V4F 5'-GTGCCAGCMGCCGCGGTAA-3' and 16S-V4R 5'-

GGACTACHVGGGTWTCTAATCC-3'), via Illumina MiSeq sequencing. Finally, PCR amplification, sequencing and taxonomic identification were carried out as described by Galan *et al.* (45). Pathogens were filtered and selected based on their potential zoonotic risk and pathogen impact on rodents (48).

Statistical analyses

Data were analysed using the R version 4.3.1 working under the RStudio software hoods (2023.06.1 Build 524). The packages Plyr (49) and ggplot2 (50) were used to create tables and figures. For each putative pathogenic taxa, prevalence was calculated by the total number of infected individuals divided by the total population sampled. Confidence intervals were calculated to 95% using the R package PropCIs (51).

Firstly, data collected from *C. glareolus* and *A. sylvaticus* in Ireland from 2021 was compared to data collected from rodents trapped in forest sites in France, sampled in 2021 (52), using the same 16S metabarcoding approach (45), to examine if less species were detected and at a lower prevalence in Ireland, related to the enemy release hypothesis. Due to differences in assigning age and maturity to animals in each country, further analysis could not be conducted.

To investigate patterns of infection in each host species in Ireland, pathogens with prevalence above 5% in the overall dataset (all sampling dates and sites) were selected for statistical analysis using generalised linear mixed models (GLMM's) and a binomial distribution (22). The threshold of 5% prevalence was selected as variations below this threshold would be too low for appropriate statistical testing. GLMM's from the package glmmTMB were used (53). GLMMs were selected for these analyses to allow the inclusion of both fixed and random effects in the models, and the appropriate distribution for each type of data being analysed. Pathogen species richness was calculated by counting the total number of putative pathogenic taxa recorded in an individual. Due to the depauperate pathogen communities in Ireland, zero-inflated negative binomial distributions were used to investigate patterns in pathogen species richness instead of Poisson distributions used by Diagne *et al.* (22).

Two strategies were used to analyse the results;

1. The invasion gradient analysis: only data from 2016 (and all nine sites) were included to test the influence of the invasion gradient on infection patterns in both *A. sylvaticus* and *C. glareolus,* as this year had a very clear "core", "front" (front 2016) and "uninvaded" (front 2021) sites as per the study by Stuart *et al.* (37) (see Figure 1). An additional overall model examining the difference in pathogen species richness between both rodent

species was conducted using these data from 2016.

2. The longitudinal analysis: data from all four years were used, but only the six sites that were sampled each year were selected, excluding Foynes 2, Carnpark and Loughkey, to keep the data comparable (Table 1). In this approach, time was included in the models as a numerical variable counting years from baseline time, i.e. 2016 = 1.

The percentage of *A. sylvaticus* relative to *C. glareolus* was tested against region to justify the classification of these sampling sites in the analyses. For these analyses, the total number *A. sylvaticus* relative to *C. glareolus* trapped in each site in each season within a given year was calculated, generating a percentage of *A. sylvaticus* relative to *C. glareolus,* and assigned to each sample corresponding to trapping site, season and year. The percentage of *A. sylvaticus* relative to *C. glareolus* in the dataset was tested against region using a Kruskal-Wallis test, and a post-hoc Dunn's test, through the dunn.test R package (54), to check differences between regions in both 2016 only and the longitudinal dataset, as the data followed a non-normal distribution.

Following the justification of the criteria in this factor, GLMMs were used for both analysis strategies. Factors used in each model included season, region (invasion region; core, front 2016, front 2021), sex, age class (juvenile, young adult and mature) as fixed effects, with site included as a random effect variable (Table 1). The longitudinal analysis included the additional fixed effects of time, as years since baseline (2016), and an interaction between season and time. An additional fixed factor, rodent species, was used for the overall model investigating the variance in pathogen species richness between *C. glareolus* and *A. sylvaticus* in 2016. The MuMIn package was used to refine and find the best model based on lowest Akaike information criterion corrected for small sample size (AICc) value (55). The DHARMa package was then used to check model fit and the performance package to test for multicollinearity and overdispersion (56, 57). The optimisation function, Optim, using the Broyden, Fletcher, Goldfarb and Shanno (BFGS) method was used in cases where model optimisation was needed. In the case where no model could be fitted, non-parametric tests were used, such as a Wilcoxon Rank Sum test. Post-hoc tests using Tukey Kramer analysis were used for factors with three or more modalities, while post-hoc Pearson's correlation tests were used to analyse changes in prevalence with time.

Table 1. Models and data used for the two approaches of this study, the invasion gradient analysis and the longitudinal analysis. Time:Season indicates the interaction that was used between time and season. Time is defined as years since 2016 baseline, with 2016 = 1.

	Invasion gradient analysis	Longitudinal analysis
**Fixed variables**	Season, Region, Age Class, Sex	Time, Season, Region, Age Class, Sex, Time:Season
**Random variables**	Site	Site
**Years**	2016 only	All (2016, 2017, 2021, 2022)
**Sites**	All (Figure 1.)	6 repeated sites; Foynes 1 & 3, Killinthomas, Donore, Moore Abbey and St. John's.
Clethrionomys glareolus	n = 148	n = 411
Apodemus sylvaticus	n = 203	n = 500

Results

Validation of invasion region criteria for models and progress of *Clethrionomys glareolus* invasion

In the 2016 data for the invasion gradient analysis, the percentage of *A. sylvaticus* relative to *C. glareolus* was directly correlated with the region of invasion. The core had the lowest percentage of *A. sylvaticus* relative to *C. glareolus*, followed by front 2016, and finally front 2021 was completely uninvaded, with 100% *A. sylvaticus* (Kruskal Wallis, $chi^2 = 271.99$, df = 2, $p < 0.001$; Dunn's test post-hoc analysis, All regions, $p < 0.001$) (Figure 2). Our capture data thus confirms the invasion categories initially designed.

In the longitudinal data, the core had a lower percentage of *A. sylvaticus* when compared to both invasion front regions, front 2016 and front 2021. However, the front 2021 was rapidly invaded in 2021 and 2022 and consequently no difference was observed between the percentage of *A. sylvaticus* in front 2016 and front 2021 (Kruskal Wallis, $chi^2 = 606.57$, df = 2, $p < 0.001$; Dunn's test post-hoc analysis, Core – Front 2016, $p < 0.001$; Core – Front 2021, $p < 0.001$; Front 2016 – Front 2021, $p > 0.05$) (Figure 2).

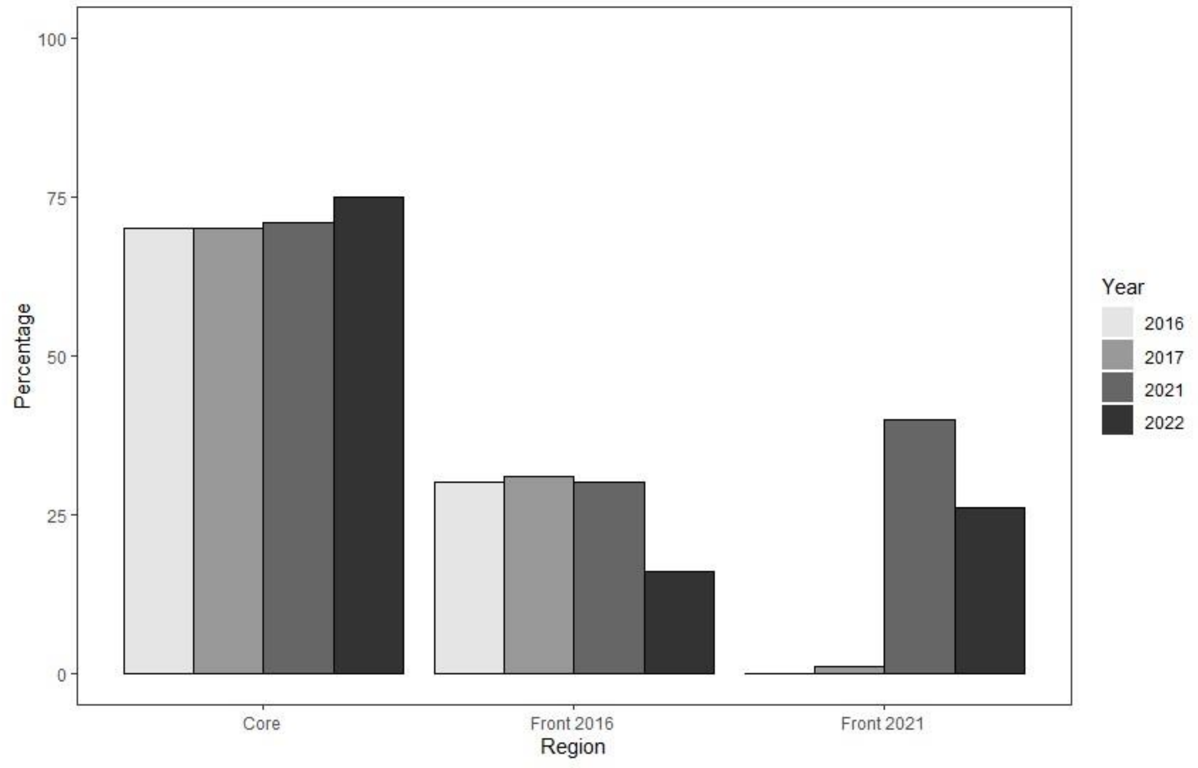

Figure 2. Percentage *Clethrionomys glareolus* relative to *Apodemus sylvaticus* in each region for each year. Data for 2016 and 2017 includes all 9 sites.

Pathogens detected in Irish rodent spleen samples

The 16S analyses of 584 *A. sylvaticus* and 498 *C. glareolus* trapped over the four years of sampling in Ireland revealed 10 putative pathogenic OTUs corresponding to five potentially pathogenic taxa (Tab[illegible]; Sequencing information, raw sequence reads and prevalence tables are available to the following link: https://zenodo.org/records/14883529). These included one OTU of the genus *Bartonella*, a bacterial genus potentially including zoonotic species (e.g. *B. henselae*), three OTUs of *Mycoplasma haemomuris* (combined for analysis, and known to be pathogenic for rodents), one OTU of *Mycoplasma coccoides* (known to be pathogenic for rodents), one OTU of *Mycoplasma penetrans* (known to be zoonotic), and four OTUs of Sarcocystidae (combined for analysis, a family of protozoans, with some species that are zoonotic, and others that are pathogenic for rodents). All five taxa were detected in *C. glareolus*, all of which had a 1% prevalence or below, except for Sarcocystidae, which had a prevalence of 37.15%. All potentially pathogenic taxa were also found in *A. sylvaticus* (Table 2), except *M. penetrans*. However, it should be noted that only one individual *C. glareolus*

was found to be positive for this pathogen out of 498 tested.

Table 2. Prevalence (%) of potentially pathogenic taxa in all *Apodemus sylvaticus* and *Clethrionomys glareolus* sampled in Ireland from 2016 - 2022. The 95% confidence interval (maximum and minimum value) is detailed in brackets.

	**Transmission**	***Apodemus sylvaticus***	***Clethrionomys glareolus***
**Pathogen**		***n = 584***	***n = 498***
Bartonella	Vector (flea)	51.54 (47.40 - 55.66)	0.2 (0.01 - 1.11)
Mycoplasma haemomuris	Vector (lice)	44.01 (39.93 - 48.14)	1 (0.33 - 2.33)
M. coccoides	Vector (lice)	44.18 (40.10 - 48.31)	0.2 (0.01 - 1.11)
M. penetrans	Sexual	0.00	0.2 (0.01 - 1.11)
Sarcocystidae	Dependent on species, *i.e.* Environmental (Direct life cycle), or intermediate host (indirect life cycle) in some cases.	1.71 (0.82 - 3.13)	37.15 (32.89 - 41.56)

Table 3. Summary of significant factors in pathogen species richness and pathogens prevalence detected.

	**Statistically significant factors observed in invasion gradient analysis (2016)**	**Statistically significant factors observed in longitudinal analysis (2016 - 2022)**
**Both Rodent Species**		
**Species Richness**	Species (Voles)***	NA
Apodemus sylvaticus		
**Species Richness**	Sex (M)*	NA
Bartonella	Season (Spring)**, Sex (M)*	**Full:** Season(Spring)***, Sex(M)*<br>**Sp:** Time*
Mycoplasma haemomuris	Age class (Mature)***	Season(Spring)**, Region(Front 2016)**, Region(Front2021)**, Age class(Juvenile)***, Age class (Mature)*
Mycoplasma coccoides	Age class (Mature)*	Time*, Age class(Mature)**
Clethrionomys glareolus		
**Sarcocystidae**	Region (Front 2016)**	**Full:** Time***, Season(Spring)***, Region(Front 2021)*, Sex(M)*, Age class(Mature)*<br>**Sp**: Region(Front 2016)*, Region(Front 2021)***, Age class(Mature)*<br>**Au:** Time***, Sex(M)*

Full = full model including all years and seasons. In some instances, per season models were ran due to multicollinearity, this is indicated by Sp = spring only model, Au = autumn only model. Time indicates years as a numerical value since baseline, with 2016 = 1. Statistically significant factors include variables in brackets. Asterisks indicate level of significance, with * = $p < 0.5$, ** = $p < 0.01$, and *** = $p < 0.001$.

Based on data from 2021, *C. glareolus* in Ireland (n = 189) exhibited fewer potentially pathogenic bacterial taxa than *C. glareolus* sampled in forest sites in France (n=221). Most notably, *Anaplasma, Borrelia, Candidatus Neoehrlichia, Francisella and Orientia* were absent from the Irish samples. In addition, lower prevalences of *Bartonella* and *M. haemomuris* were also observed in the Irish *C. glareolus* specimens (Figure 3a). Moreover, *A. sylvaticus* individuals trapped in Ireland (*n* = 193) had a similar prevalence of *Bartonella* compared to forest site

populations in France in 2021 (n = 95), but higher prevalences of *M. haemomuris and M. coccoides*. As above, *Anaplasma, Borrelia, Candidatus Neoehrlichia, Francisella and Orientia* were not detected in the Irish *A. sylvaticus* samples (Figure 3b).

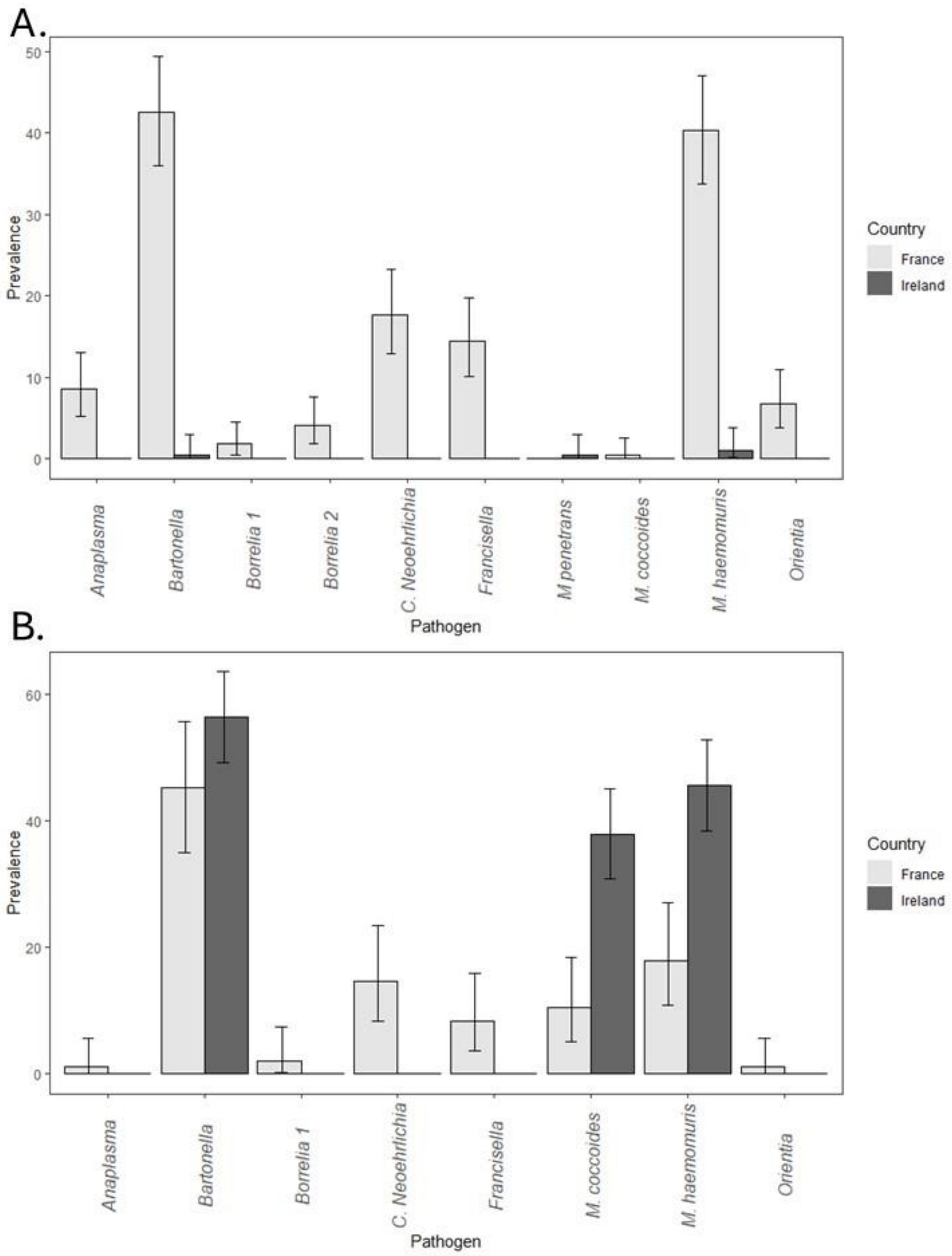

Figure 3. Prevalence of potentially pathogenic bacterial taxa observed in 2021 in both France (*Clethrionomys*

glareolus native range, light grey) and Ireland (*C. glareolus* invasive range, dark grey) in **A.** *C. glareolus* and **B.** *Apodemus sylvaticus*. Error bars represent 95% confidence intervals ~~(Maximum and Minimum).~~ *C. Neoehrlichia* represents *Candidatus Neoehrlichia.*

Pathogen richness

The best model (lowest AICc value) explaining variations in the species richness of potentially pathogenic taxa between *C. glareolus* and *A. sylvaticus* in 2016 included only rodent species. *C. glareolus* ($\bar{x}$ = 0.46) was shown to have a lower mean number of pathogen species compared to *A. sylvaticus* ($\bar{x}$ = 1.37) (GLMM, family = Zero inflated negative binomial, z = -8.068, p < 0.001). However, poor model fit was still observed when examining residuals, hence nonparametric tests were carried out to confirm these findings. Using a Wilcoxon Rank Sum test, a significant difference in species richness between *C. glareolus* and *A. sylvaticus* was confirmed, with the invasive *C. glareolus* (med = 0) having a lower median number of pathogens compared to *A. sylvaticus* (med = 1) (W = 23262, p < 0.001).

When focusing on *A.* sylvaticus, using the invasion gradient analysis, no effect for the *C. glareolus* invasion was observed on the species richness of potentially pathogenic taxa. The best model (lowest AICc value) included only sex. Sex was shown to be statistically significant with males ($\bar{x}$ = 1.54) having a higher mean pathogen species richness compared to females ($\bar{x}$ = 1.14) (GLMM, family = Zero inflated negative binomial, z = 2.391, p = 0.017). As before, due to poor model fit, these findings were confirmed using a Wilcoxon Rank Sum test, showing males (med = 2) have a higher median species richness compared to females (med = 1) (W = 3830.5, p < 0.01).

Apodemus sylvaticus

The patterns of infections observed in the invasion gradient and longitudinal analyses for the pathogen species *Bartonella, M. haemomuris* and *M. coccoides* were examined in *A. sylvaticus* sampled in Ireland.

Bartonella

Considering the invasion gradient analysis, the best model (lowest AICc value) included season and sex. *Bartonella* prevalences were higher in autumn compared to spring (GLMM, family = Binomial, $z = -2.741$, $p = 0.00612$), while males were more infected than females (GLMM, family = Binomial, $z = 2.404$, $p < 0.05$).

In the longitudinal analysis, the best model (lowest AICc value) included time, season, region, and sex, with an interaction between time and season. The interaction between time and season was shown to have a high variance inflation factor (VIF), meaning the covariates were correlated and potentially affecting the model through multicollinearity. To account for this, the interaction was removed in the overall model, and separate GLMM's were conducted for each season. In the overall model, no effect for time or region was observed ($p > 0.05$). *A. sylvaticus* in autumn showed higher prevalences of *Bartonella* than in spring (GLMM, family = Binomial, $z = -3.708$, $p < 0.001$). Alongside this, males exhibited higher prevalences compared to females (GLMM, family = Binomial, $z = 2.241$, $p < 0.05$).

In the seasonal models, the best model (lowest AICc value) for spring included time and sex. No effect for region was observed. Prevalence was positively correlated (0.15) with time (GLMM, family = Binomial, $z = 2.344$, $p = 0.019$; Pearsons's correlation test, $t = 2.4751$, $p < 0.05$). Sex did not significantly influence the prevalence of *Bartonella* in *A. sylvaticus* in the spring only model ($p > 0.05$). In the autumn only analysis, no factor significantly influenced *Bartonella* prevalence.

Mycoplasma haemomuris

Considering the invasion gradient analysis, the best model (lowest AICc value) included only age class. No effect for region was observed. Age class was statistically significant (Age class: Mature, GLMM, family = Binomial, $z = 3.405$, $p < 0.001$). Post-hoc analysis showed that mature adults had significantly higher prevalences compared to young adults and juveniles. No difference between young adults and juveniles was observed, possibly due to the low number of juveniles in the dataset (n = 9) (Tukey Kramer analysis; Mature – Young adults, $p < 0.01$; Mature – Juveniles, $p < 0.05$; Young adults – Juveniles, $p > 0.05$).

In the longitudinal analysis, no effect for time was observed, and the best model selected (lowest AICc value) included the factors season, region, and age class. Region was shown to have a significant effect on the prevalence of *M. haemomuris* (Region: Front 2016, GLMM, family = Binomial, $z = 2.744$, $p < 0.01$; Region:

Front 2021, GLMM, family = Binomial, $z = 2.874$, $p < 0.01$). Post-hoc analysis demonstrated that the core had a lower prevalence of *M. haemomuris* compared to front 2016 and front 2021, with no difference observed between front 2016 and front 2021 (Figure 4) (Tukey Kramer analysis; Core – Front 2016, $p < 0.001$; Core – Front 2021, $p < 0.001$; Front 2016 – Front 2021, $p > 0.05$). Secondly, spring had higher prevalences compared to autumn (GLMM, family = Binomial, $z = 2.987$, $p < 0.01$). Finally, age class was shown to be a statistically significant factor (Age class: Juvenile, GLMM, family = Binomial, $z = -3.414$, $p < 0.001$; Age class: Mature, GLMM, family = Binomial, $z = 2.146$, $p < 0.05$). Post-hoc analysis showed mature adults were the most infected, followed by subadults, with juveniles least infected (Tukey Kramer analysis; Mature - Young adults, $p < 0.01$; Mature – Juveniles, $p < 0.001$; Juveniles – Young adults, $p < 0.001$).

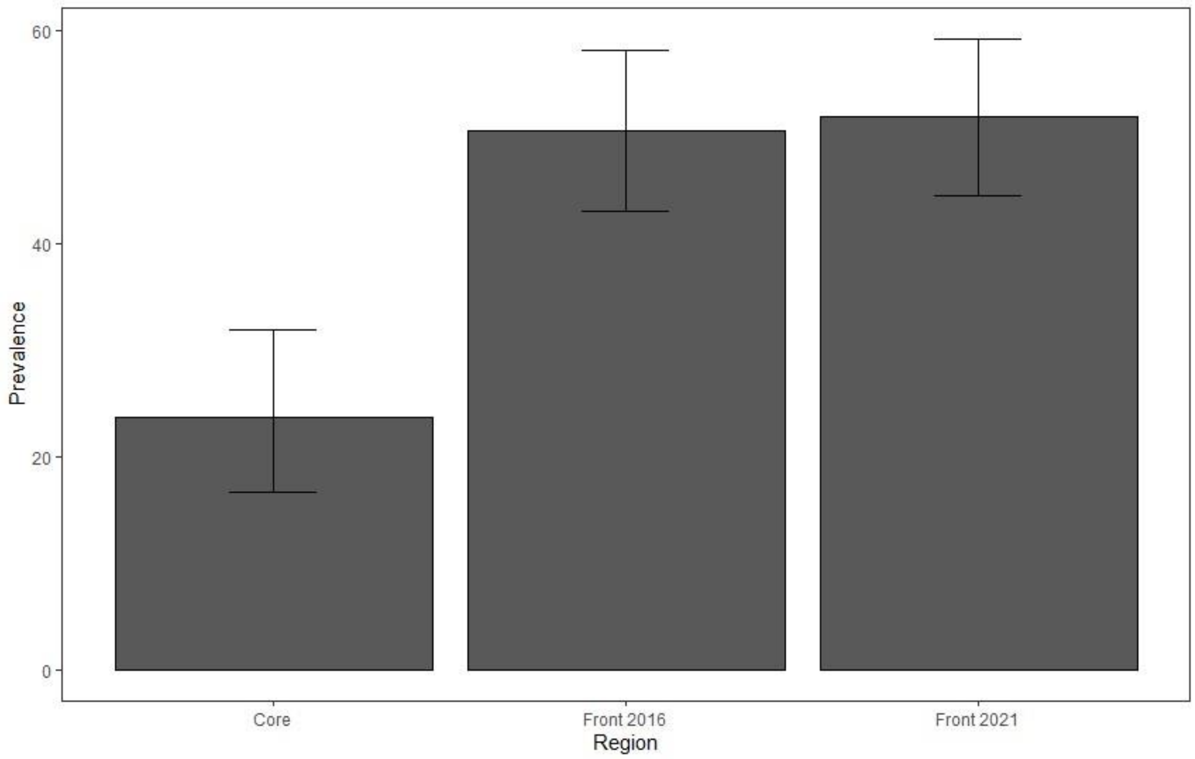

Figure 4. Prevalence of *Mycoplasma haemomuris* in *Apodemus sylvaticus* per region in the longitudinal analysis (all years). Error bars represent 95% confidence intervals ~~(Maximum and Minimum).~~

Mycoplasma coccoides

For the invasion gradient analysis, the best model (lowest AICc value) included only the null model, therefore the second lowest AICc model was used, including only age class and sex. Age class was the only statistically

significant variable (Age class: Mature, GLMM, family = Binomial, $z = -2.115$, $p < 0.05$). However, during post-hoc analysis no statistical differences was observed between pairs of age class (Tukey Kramer analysis, $p > 0.05$). No effect of the *C. glareolus* invasion gradient on *M. coccoides* prevalence was observed, as region was excluded from the lowest AICc model.

The longitudinal analysis showed that the best model (lowest AICc value) included time, sex and age class. Time was shown to be a significant factor (GLMM, family = Binomial, $z = -2.175$, $p < 0.05$), with a negative correlation (-0.1) between prevalence and time (years) (Pearson's correlation test, t=-2.2929, p < 0.05). This can be observed in Figure 5, where a reduction in prevalence is seen over time. Age class was also shown to affect prevalence (Age class: Mature, GLMM, family = Binomial, $z = -3.04$, $p < 0.01$). Post-hoc analysis demonstrated young adults being more infected than mature adults, however, no statistical differences were observed between mature adults and juveniles and between juveniles and young adults (Tukey Kramer analysis; Young adults – Mature, $p < 0.05$; Mature – Juveniles, $p > 0.05$; Young adults – Juveniles, $p > 0.05$).

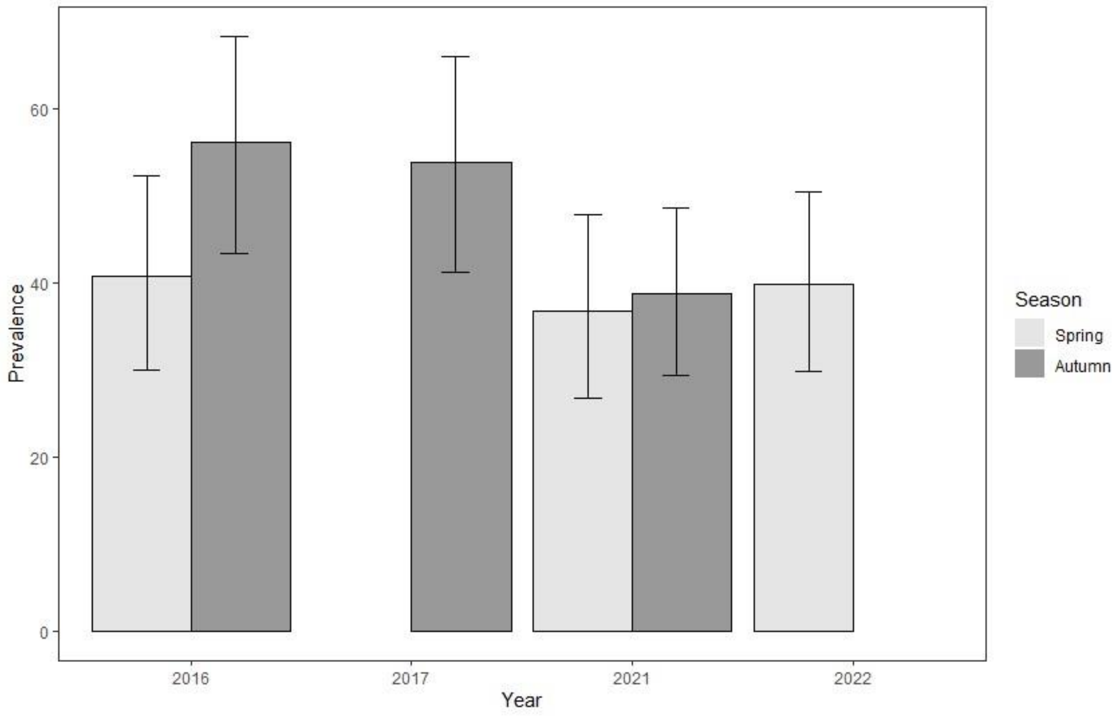

Figure 5. Prevalence of *Mycoplasma coccoides* in *Apodemus sylvaticus* for each season and year sampled. Error bars represent 95% confidence intervals ~~(Maximum and Minimum)~~.

Clethrionomys glareolus

Regarding *C. glareolus* in Ireland, the patterns of infection with Sarcocystidae observed during the invasion gradient and longitudinal analyses were examined.

Sarcocystidae

Considering the invasion gradient analysis on the prevalence of Sarcocystidae in *C. glareolus,* the best model (lowest AICc value) included only region. The invasion front (front 2016) showed a significantly higher prevalence when compared to the core (Region: Front 2016, GLMM, family = Binomial, $z = 2.652$, $p < 0.01$) (Figure 6). Similarly, the same invasion gradient analysis was performed on a single OTU of Sarcocystidae specific to *C. glareolus*. The best model (lowest AICc value) included region and age class. Region showed the same pattern as the analysis on all Sarcocystidae, with higher prevalence of this Sarcocystidae OTU in the front 2016 compared to the core (Region: Front 2016, GLMM, family = Binomial, $z = 2.187$, $p < 0.05$). Age class was not statistically significant ($p > 0.05$).

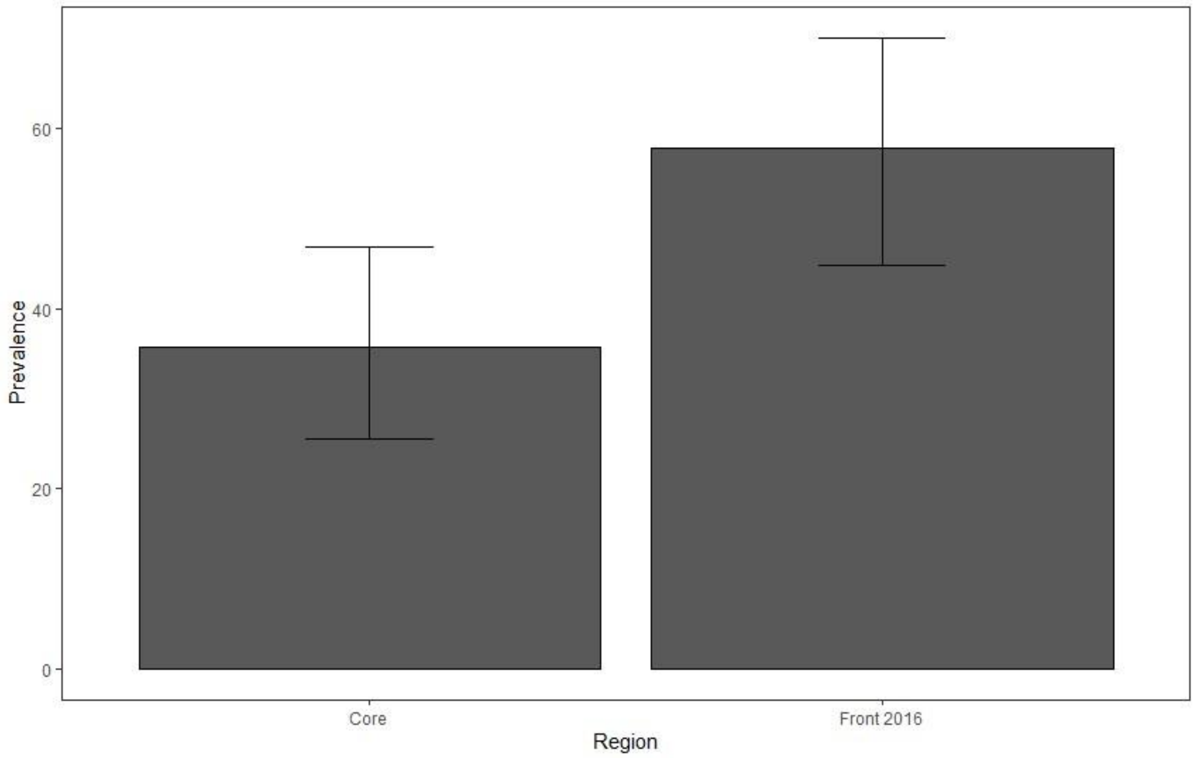

Figure 6. Prevalence of Sarcocystidae in *Clethrionomys glareolus* per region in 2016. Error bars represent 95% confidence intervals ~~(Minimum and Maximum~~).

The longitudinal analysis of the prevalence of Sarcocystidae in *C. glareolus* found the best model (lowest AICc value) included time, season, region, sex, age class and an interaction between time and season. A high VIF was observed for the interaction and hence it was removed from the final model. Time was shown to be statistically significant (GLMM, family = Binomial, $z = -4.849$, $p < 0.001$). Post-hoc analysis found that prevalence is negatively correlated (-0.22) with time (years since baseline) (Pearson's correlation test; $t = -4.6122$, $p < 0.001$, Figure 7). Higher prevalence was observed in spring compared to autumn (GLMM, family = Binomial, $z = 3.547$, $p < 0.001$). Region was shown to be statistically significant (Region: Front 2021, GLMM, family = Binomial, $z = 2.143$, $p < 0.05$). Post-hoc analysis demonstrates that the core had a lower prevalence compared to front 2016 but not statistically different from front 2021, while no statistical difference was observed between front 2016 and front 2021 (Tukey Kramer analysis; Core – Front 2016, $p < 0.05$; Core – Front 2021, $p > 0.05$; Front 2016 – Front 2021, $p > 0.05$). Females had higher prevalences than males (GLMM, family = Binomial, $z = -2.216$, $p < 0.05$). Finally, age class was shown to be a statistically significant factor (Age class: Mature, GLMM, family = Binomial, $z = -2.322$, $p < 0.05$). Post-hoc analysis showed that juveniles were more infected

than mature adults. No statistical difference was observed between mature adults and young adults, or young adults and juveniles (Tukey Kramer analysis; Juveniles – Mature, $p < 0.01$; Juveniles – Young adults, $p > 0.05$; Mature – Young adults, $p > 0.05$).

Separate binomial GLMMs for each season were run on the longitudinal models to account for the removed interaction between season and time. In spring, the best model (lowest AICc value) included region and age class. Region was shown to be statistically significant (Region: Front 2016, GLMM, family = Binomial, $z = 2.188$, $p < 0.05$; Region: Front 2021, GLMM, family = Binomial, $z = 3.769$, $p < 0.001$). Post-hoc analysis demonstrated that the core had a lower prevalence when compared to both front 2016 and front 2021, while no difference was observed in the post hoc analysis between the two front regions identified in 2016 and 2021 (Tukey Kramer analysis; Core – Front 2016 $p < 0.01$; Core – Front 2021 $p < 0.001$; Front 2016 – Front 2021, $p > 0.05$). Age class was also statistically significant (Age class: Mature, GLMM, family = Binomial, $z = -2.112$, $p < 0.05$). Post-hoc analysis showed juveniles and young adults more likely to be infected compared to mature adults, but no statistical difference was observed between juveniles and young adults (Tukey Kramer analysis; Mature – Juveniles, $p < 0.01$; Mature – Young adults, $p < 0.05$; Young adults – Juveniles, $p > 0.05$).

Finally, the best model (lowest AICc value) for autumn included time, sex, and age class. No effect for region was observed. Time was shown to be statistically significant (GLMM, family = Binomial, $z = -6.766$, $p < 0.001$) with time being negatively correlated (-0.5) with prevalence (Pearsons's correlation test, $t = -8.5352$, $p < 0.001$, Figure 7). Females had higher prevalences compared to males (GLMM, family = Binomial, $z = -2.231$, $p < 0.05$). Age class did not significantly influence the prevalence of Sarcocystidae in this analysis.

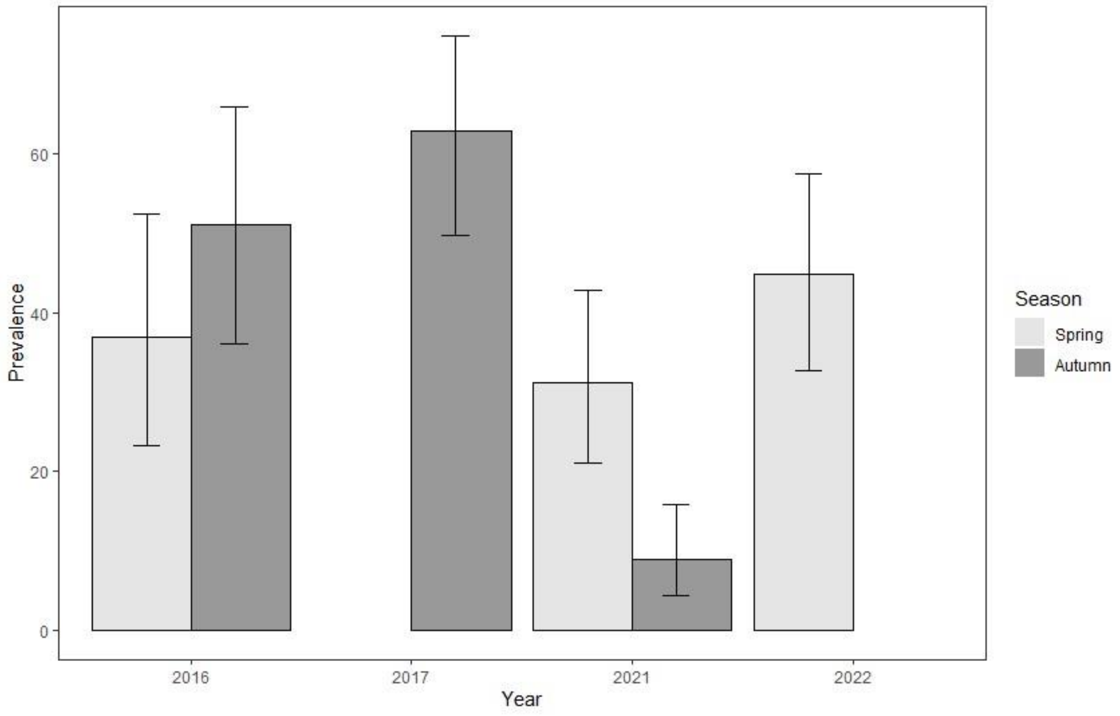

Figure 7. Prevalence of Sarcocystidae in *Clethrionomys glareolus* between each season per year. Error bars represent 95% confidence intervals (Maximum and Minimum).

Discussion

The aim of this study was to investigate the effect an invasive species may have on native pathogen communities, including zoonotics, using the bio-invasion of Ireland by *C. glareolus* as a model system. While previous studies in Ireland have focused on single bacterial pathogens in rodents before, such as Bartonella or Borrelia (58-60), this is the first time 16SrRNA screening techniques have been used to identify potential pathogens of zoonotic risk circulating in Irish populations of *C. glareolus and A. sylvaticus*, creating an important baseline for rodent-borne disease studies in Ireland, and highlighting new important areas of research (e.g Zintl *et al.*, 2023 (61)).

Rodent-borne infectious risk in Ireland

Firstly, we discovered that Irish rodents exhibit a depauperate pathogen richness, with OTUs relating to four pathogenic bacterial species and one family of protozoans in sampled rodent populations. Although the species

in the Sarcocystidae OTUs were not identified in the present study, comparison with published sequences using NCBI BLAST indicated these may contain pathogens of veterinary and medical importance, including *Toxoplasma, Eimeria* or *Neospora*. Furthermore, we observed that the *C. glareolus* population in Ireland expresses patterns consistent with the enemy release hypothesis, and potentially disrupts the transmission of native pathogen species. Finally, this study examined the importance of considering time when examining the effect biological invaders have on pathogen communities. For example, in contrast to a previous study by Telfer *et al.*, (60) we detected *Bartonella* in *C. glareolus* in Ireland, albeit at a low prevalence rate. This result was surprising as the previous study had postulated that *C. glareolus* in Ireland might be resistant to native strains (60). Further molecular work is required to determine if the *Bartonella* observed during this study is the same species of *Bartonella* observed in mice during the study by Telfer *et al.*, (60).

It is also worth noting that despite being native, *A. sylvaticus* also exhibited a depauperate community of potentially pathogenic bacterial taxa when compared to French populations, with no *Anaplasma, Candidatus Neoehrlichia, Francisella* or *Orientia* observed in the Irish population. This is consistent with island biogeography theory, which states that species diversity is limited by their size and isolation from the mainland (62, 63). The absence of certain vector-borne pathogens might also be explained by the low vector diversity in Ireland. For example, reviews in Ireland and France have shown Ireland to have 13 species of ticks, compared to approximately 40 tick species in France (64, 65). However, assays specifically targeting these pathogens would need to be carried out to confirm their absence. Furthermore, *Borrelia* was also notably absent from Irish rodents in this study, although it had been detected, albeit at low prevalences, in Irish rodents by previous studies (58, 59). This discrepancy may again be a result of the methods and target organ used in the present study, with previous studies using ear biopsies and species specific PCR (58, 59). Also, while *Apodemus* species and *C. glareolus* are considered important aspects of the *Borrelia* life cycle in Europe, these rodents may play a limited role as reservoir in Ireland, with the rodent-associated, *Borrelia afzelii*, being less common in Ireland (58, 59, 66, 67). Additionally, the absence of *Anaplasma* from rodents sampled in the present study agree with findings reported by Flattery *et al.* (68), who screened questing nymphal *Ixodes ricinus* ticks collected across Ireland for the presence of *Anaplasma phagocytophilum* and only found groEL ecotype 1, a strain that is not thought to be associated with rodents.

Enemy release in the invasive *Clethrionomys glareolus*

Two patterns consistent with the enemy release hypothesis were observed for bacterial pathogens in the invasive *C. glareolus* population in Ireland for the first time, at both an international intraspecific scale and interspecific scale. Firstly, by comparing the number of bacterial taxa in Irish and French *C. glareolus* populations, the population of *C. glareolus* sampled in Ireland had fewer potentially pathogenic bacterial taxa when compared to conspecifics in their native range. This can be seen particularly in the 2021 data, where the Irish *C. glareolus* individuals had fewer potentially pathogenic bacterial taxa and lower prevalences compared to *C. glareolus* populations in France. In particular, a number of potentially pathogenic taxa were completely absent from *C. glareolus* populations in Ireland including: *Anaplasma, Candidatus Neoehrlichia, Francisella and Orientia*. Further studies should be conducted to confirm this and incorporate consistent aging techniques in both countries allowing for more in-depth statistical analysis between samples from Ireland and France. Secondly, *C. glareolus* exhibited enemy release from potentially pathogenic bacterial taxa in comparison to the native *A. sylvaticus* in Ireland. While *C. glareolus* was found to have one more bacterial taxon when compared to *A. sylvaticus, C. glareolus* has lower prevalences of these bacterial taxa in the overall data, and a significantly lower pathogen species richness compared to *A. sylvaticus* in the invasion gradient analysis (2016 only).

These findings are comparable to studies investigating the helminth communities in the invasive *C. glareolus* in Ireland (21, 37). These authors found the same two examples of enemy release, i.e. *C. glareolus* had a lower helminth species richness compared to its native range, and to the native *A. sylvaticus* in Ireland (21, 37). Likewise, similar patterns have been observed in other invasion systems. For example in the Senegal invasion system, Diagne *et al.* (22) demonstrated that native rodents (*Mastomys erythroleucus* and *Mastomys natalensis*) had a greater species richness of pathogenic bacterial OTUs when compared to non-native rodents (*Mus musculus domesticus* and *Rattus rattus*). These findings from investigating invasion systems in different countries and invasive species can provide insights into the underlying mechanisms contributing to how these species survive, establish and outcompete native fauna when colonising new environments. For example, this pattern of enemy release could allow the invasive species to direct more energy from immune responses into growth or fecundity, potentially giving invasive species a competitive advantage over native species (69).

Stuart *et al.* (37) described a third example of enemy release in the Irish population of *C. glareolus,* namely the reduced abundance of the directly transmitted nematode *A. tianjinsensis* in *C. glareolus* in the invasion front compared to the invasion core in spring and autumn in 2016 (37). A similar example of enemy release at the

invasion front was not found during this present study. In contrast, in the invasion gradient analysis, *C. glareolus* was more likely to be infected in the invasion front, compared to the more established invasion core, with significantly higher prevalences of Sarcocystidae in front 2016 compared to the core. This pattern was also observed in 2016 for the single Sarcocystidae OTU which seems to be specific to *C. glareolus*, with no *A. sylvaticus* found infected by this OTU in this study. These findings are in direct contrast to the enemy release hypothesis, as the more recently invaded populations exhibited higher infection rates, highlighting that infection patterns may be highly pathogen specific.

This result was all the more surprising given that male *C. glareolus* at the invasion front are thought to be more timid and less exploratory than their conspecifics at core sites (70), characteristics which would be expected to reduce their exposure to pathogens. On the other hand, there also seems to be a strong link between spatial behaviour and behaviour traits, with shy but thorough explorers using larger areas (70). If animals are exploring larger areas at the expansion front, this could potentially result in greater exposures to pathogens.

Finally, a possible explanation for the increased infection rate at the invasion front might lie in the immunology of *C. glareolus* during its invasion of Ireland. Pathogens may lag behind biological invasions, as the population density of the invading species at the front is too low to maintain sufficient transmission. Consequently, the reallocation of resources from immune responses, known as the evolution of increased competitive ability hypothesis and enemy release from pathogens, could be favoured in invasive populations (20, 44). This could potentially leave *C. glareolus* in the invasion front relatively immunologically naïve and hence, susceptible to increased Sarcocystidae infection when the population increases over a certain threshold. This would be further supported by the findings in the longitudinal analysis, that juveniles have a higher prevalence of Sarcocystidae compared to mature adults, as juveniles would potentially be more immunologically naïve. In a study of the population genomics of *C. glareolus* in Ireland, White *et al.* (71) identified the selection of genes with immunological function in *C. glareolus* furthest from the point of introduction. However, to fully understand these mechanisms further investigation of the eco-immunology of invasive *C. glareolus* populations, and identification of the species present within these Sarcocystidae OTUs is required.

Effect of *Clethrionomys glareolus* invasion on pathogen communities in the native *Apodemus sylvaticus*

The effect of the *C. glareolus* invasion on the pathogen communities in the native *A. sylvaticus* was also investigated. It was hypothesised that the presence of *C. glareolus* might affect the pathogen communities in the

native *A. sylvaticus*. However, no dilution or amplification effect resulting from the *C. glareolus* invasion gradient on pathogen species richness in *A. sylvaticus* in the present study. Likewise, in the invasion gradient analysis (2016 data only), no effect for invasion region was found on the prevalence of *A. sylvaticus'* pathogens. However, using the longitudinal data, the increased relative abundance of *C. glareolus* at the site of introduction apparently caused a dilution effect for *M. haemomuris* in *A. sylvaticus*. In this instance the core showed significantly reduced prevalences of *M. haemomuris* in *A. sylvaticus* compared to conspecifics from the invasion front regions (front 2016 and front 2021). No difference was observed between front 2016 and front 2021 indicating that the reduction in *M. haemomuris* prevalence may be directly attributable to the higher *C. glareolus* abundance at the core. The potential reason for a dilution effect being observed in the longitudinal data and not in the 2016 only data is likely due to the increased statistical power in the longitudinal analysis, as a result of the increased sample size.

In a previous study investigating the impact of forest anthropization on small mammal pathogens, Bouilloud (72) detected a dilution effect for *M. haemomuris* (Strain 2) associated with increased small mammal diversity in France. The author argued that the declining abundance of its most competent host, *C. glareolus,* as host species diversity increased, might explain the dilution of *M. haemomuris* at these sites. The dilution of *M. haemomuris,* in *A. sylvaticus* in Ireland due to the presence of *C. glareolus,* and the dilution of the strain 2 in France, presumably due to increased small mammal diversity, demonstrate that despite the multiple factors that can alter rodent populations (e.g. climate change, invasive species, deforestation and urbanisation), that understanding the underlying mechanisms behind rodent-borne disease can allow for better predictions of disease transmission and improved disease management. Interestingly, the dilution pattern identified for *M. haemomuris* was not observed for *M. coccoides*. The driver behind the different patterns of these *Mycoplasma* spp. may possibly be a result of the specific behaviour of the respective vector species, *Polyplax serrata* in the case of *M. coccoides* and *Polyplax spinulosa* in the case of *M. haemomuris* (73, 74). However, studies on these pathogens vector species are based on mice and rats' laboratory experiments only. Therefore, field-based studies are needed to confirm the possibility that *C. glareolus* might be a less favourable host for the vector transmitting *M. haemomuris*, or the bacteria itself, in Ireland, which could result in a dilution effect in the native *A. sylvaticus.*

Surprisingly, we detected no dilution effect for *Bartonella* in *A. sylvaticus* in this present study. *Bartonella birtlesii* and *Bartonella taylorii* have previously been recorded in Ireland, with prevalences in *A. sylvaticus* decreasing with increased *C. glareolus* densities (60). These seemingly contradictory observations may be due

to the time that has elapsed since the study by Telfer *et al.* (60) and the present study. It is possible for example, that the initial invasion of *C. glareolus* disrupted these pathogens, but they have recovered since. On the other hand, only a single *C. glareolus* individual was found to be infected with *Bartonella* here, corroborating the suggestion that *C. glareolus* in Ireland is not a competent host for this pathogen species. Hence, the observed difference may stem from the detection techniques employed, as Telfer *et al.* (60) utilized bacterial cultivation and genus-specific PCR assays, while this present study only found one OTU of *Bartonella,* using 16SrRNA amplification and illumina sequencing. Further work, using genus specific primers, such as those used by Telfer *et al.* (60) would be needed to examine *Bartonella* positive rodents to determine species identity, and hence identify whether dilution does still occur in the Irish *A. sylvaticus* population.

Changes in rodent pathogen communities over time

Finally, the effect of time on the prevalence of the various pathogenic taxa, as the *C. glareolus* invasion progressed was examined. Prevalence decreased between 2016 and 2022, for both *M. coccoides* in *A. sylvaticus* and Sarcocystidae in *C. glareolus*. However, examining the spring longitudinal data only, *Bartonella* prevalence in *A. sylvaticus* increased between 2016 and 2022. These patterns show that pathogen communities are dynamic, and longitudinal studies are essential for fully understanding the changes that occur in pathogen communities. Cornet *et al.* (20), expressed the need for longitudinal studies to examine the time-lag for invasive species between invasion and adapting to the invasion front. A recent review by Brian and Catford (75) highlighted that time since introduction is a significant factor when investigating the enemy release hypothesis, as enemy impact on the invader will potentially increase with time, as pathogens adapt to the invasive species. In our case, the dilution effect for *M. haemomuris* in *A. sylvaticus* was only observed in the longitudinal data, an observation that could potentially have been missed in a single timepoint study. Moreover, *M. haemomuris, M. penetrans* and *Bartonella* were not detected in *C. glareolus* in the baseline 2016 data. While this could be attributed to the increased sample size afforded by multiple years of sampling, focusing on a single year, could have resulted in missing the dilution for *M. haemomuris* in *A. sylvaticus* and the assumption that *C. glareolus* is not infected by these pathogens in Ireland. Similarly, the recently reported *Babesia microti* 'Munich' strain in Ireland was detected exclusively in *C. glareolus* located in the eastern part of the country, which corresponds to the *C. glareolus* invasion front (61). The primary vector of this strain, *Ixodes trianguliceps*, has also been mainly reported in eastern Ireland (76). If the assumption that *B. microti* is confined to the eastern regions of Ireland is

correct, this would further demonstrate the importance of long-term studies on the pathogens of invasive species, to monitor the emergence of new pathogens in newly colonised areas. Finally, more long-term data is needed to fully understand if rodents and their pathogens fluctuate through multi-annual cycles, which is seen in other countries where long-term data is gathered, such as Finland (77-79).

Conclusion

In conclusion, patterns consistent with the enemy release hypothesis were observed in the Irish *C. glareolus* population. *C. glareolus* in Ireland shows reduced pathogen species richness, and lower bacterial prevalences, compared to the native *A. sylvaticus* and fewer bacterial taxa compared to the native range of the species. These findings increase our understanding of the mechanism of how invasive species thrive in new environments and outcompete native fauna, however, eco-immunological data are required to fully understand these patterns of enemy release. Secondly, the present study showed the disruptive effect of non-native host species on native pathogen systems, with the example of a dilution effect and reduced prevalence of *M. haemomuris* in *A. sylvaticus* at the invasion core compared to invasion fronts, where *C. glareolus* populations exhibit higher abundance. These findings show how changes in biodiversity, such as the addition of new hosts with varying levels of competencies for a given pathogen, may disrupt pathogen communities and host-pathogen dynamics. Further~~,~~ work is ~~also~~ needed to identify the Sarcocystidae and *Bartonella* species detected in this study in order to fully understand the pathogen community dynamics, as genetic differences may reveal different levels of host specificity and help explain host-pathogen interactions. Finally, time, as the *C. glareolus* invasion progresses, was shown to be correlated, both positively and negatively, with three pathogen taxa during this study, illustrating that invasion systems are dynamic, and pathogen communities can fluctuate as the invader becomes more established. By conducting a longitudinal investigation, we demonstrate that long-term data are essential to fully understand eco-evolutionary processes driving invasion dynamics and success, preventing conclusions drawn from single timepoint studies being assumed as long-term changes in pathogen communities.

Acknowledgements

We would like to thank Ian Montgomery, Colin Lawton and John Rochford for loaning their traps. We would also like to thank Breda Fitzpatrick, Patrick Dalton, Hugh O'Connor and the other technical staff at MTU for

their help and loan of equipment. Finally, we would like to thank TCD students, volunteers and interns, Laura McCarthy, Lucie Veitová and Paula Curtin for their help in the field and lab processing the Irish samples. We are grateful to Audrey Weber (AGAP laboratory) and the GenSeq facilities for the Illumina MiSeq sequencing, and the Genotoul bioinformatics platform Toulouse Midi-Pyrénées (Bioinfo Genotoul). Thank you to all the BioRodDis team members who have been vital to this study through the sharing of data, discussion of findings and statistical analyses. We would also like to thank Niamh Reilly for helping proofread the manuscript.

Statements & Declarations

Funding

This research was funded through the 2018–2019 BiodivERsA joint call for research proposals, under the BiodivErsA3 ERA-Net COFUND programme, and with the funding organisations EPA Research Programme, Agence Nationale de la Recherche (ANR), Research Foundation–Flanders (FWO), National Science Centre, Poland (NCN) and Deutsche Forschungsgemeinschaft (DFG). This project is funded under the EPA Research Programme 2021–2030 and cofunded by Agence Nationale de la Recherche (ANR), Research Foundation–Flanders (FWO), National Science Centre, Poland (NCN) and Deutsche Forschungsgemeinschaft (DFG).

This project is funded under the EPA Research Programme 2021-2030. The EPA Research Programme is a Government of Ireland initiative funded by the Department of the Environment, Climate and Communications (project number 2019-NC-MS-10).

2016 and 2017 sampling was funded by the Irish Research Council, Government of Ireland Postdoctoral Fellowship Programme and by the Marie Curie Research Grants Scheme, European Union, grant (749312, INVASIMMUN).

Ethics statement:

This study was carried out under ethical approval from the universities Trinity College Dublin animal research ethics committee and Munster Technological University ethics board, in compliance with the Health Products

Regulatory Association (HPRA) of Ireland for euthanasia of the rodents sampled (Authorisation Number: AE22171/I004).

Competing Interests:

The authors declare no competing or financial interests.

Author contributions.

Andrew McManus, Celia Holland, Nathalie Charbonnel, Maxime Galan and Peter Stuart contributed to the study conception and design. Material preparation, data collection and analysis were performed by Andrew McManus, Nathalie Charbonnel, Maxime Galan, Peter Stuart and Vincent Sluydst. The first draft of the manuscript was prepared by Andrew McManus and all authors commented on previous version of the manuscript. All authors have read and approved the final manuscript.

References

1. Jones KE, Patel NG, Levy MA, Storeygard A, Balk D, Gittleman JL, et al. Global trends in emerging infectious diseases. Nature. 2008;451(7181):990-3. doi: 10.1038/nature06536
2. Brown K, Leggat PA. Human monkeypox: current state of knowledge and implications for the future. Tropical medicine and infectious disease. 2016;1(1):8. doi: 10.3390/tropicalmed1010008
3. Mackenzie JS, Smith DW. COVID-19: a novel zoonotic disease caused by a coronavirus from China: what we know and what we don't. Microbiology Australia. 2020;41(1):45-50. doi: 10.1071/MA20013
4. Roche B, Garchitorena A, Guégan JF, Arnal A, Roiz D, Morand S, et al. Was the COVID-19 pandemic avoidable? A call for a "solution-oriented" approach in pathogen evolutionary ecology to prevent future outbreaks. Ecology letters. 2020. doi: 10.1111/ele.13586
5. Heinrich ML, Boisen ML, Nelson DK, Bush DJ, Cross RW, Koval AP, et al. Antibodies from Sierra Leonean and Nigerian Lassa fever survivors cross-react with recombinant proteins representing Lassa viruses of divergent lineages. Scientific Reports. 2020;10(1):1-19. doi: 10.1038/s41598-020-72539-w

6. Dharmarajan G, Li R, Chanda E, Dean KR, Dirzo R, Jakobsen KS, et al. The animal origin of major human infectious diseases: what can past epidemics teach us about preventing the next pandemic? Zoonoses. 2022;2(1).

7. Dhawan M, Choudhary OP. Emergence of monkeypox: risk assessment and containment measures. Travel Medicine and Infectious Disease. 2022;49:102392. doi: 10.1016/j.tmaid.2022.102392

8. Daszak P, Cunningham AA, Hyatt AD. Emerging infectious diseases of wildlife--threats to biodiversity and human health. science. 2000;287(5452):443-9. DOI: 10.1126/science.287.5452.443

9. Wikelski M, Foufopoulos J, Vargas H, Snell H. Galápagos birds and diseases: invasive pathogens as threats for island species. Ecology and Society. 2004;9(1).

10. Wilcox BA, Gubler DJ. Disease ecology and the global emergence of zoonotic pathogens. Environmental Health and Preventive Medicine. 2005;10(5):263-72. doi: 10.1265/ehpm.10.263

11. Hatcher MJ, Dick JT, Dunn AM. Disease emergence and invasions. Functional Ecology. 2012;26(6):1275-87. doi: 10.1111/j.1365-2435.2012.02031.x

12. Becker DJ, Albery GF, Kessler MK, Lunn TJ, Falvo CA, Czirják GÁ, et al. Macroimmunology: The drivers and consequences of spatial patterns in wildlife immune defence. Journal of Animal Ecology. 2020;89(4):972-95. doi: 10.1111/1365-2656.13166

13. IPBES. Global assessment report on biodiversity and ecosystem services of the Intergovernmental Science-Policy Platform on Biodiversity and Ecosystem Services. In: Brondízio ES, Settele J, Díaz S, Ngo HT, (Editors), editors.: IPBES secretariat, Bonn, Germany; 2019. doi: 10.5281/zenodo.3831673

14. Schreiner CL, Nuismer SL, Basinski AJ. When to vaccinate a fluctuating wildlife population: Is timing everything? Journal of Applied Ecology. 2020;57(2):307-19. doi: 10.1111/1365-2664.13539

15. Bezerra-Santos MA, Dantas-Torres F, Mendoza-Roldan JA, Thompson RA, Modry D, Otranto D. Invasive mammalian wildlife and the risk of zoonotic parasites. Trends in parasitology. 2023;39(9):786-98. DOI: 10.1016/j.pt.2023.06.004

16. Eby P, Peel AJ, Hoegh A, Madden W, Giles JR, Hudson PJ, et al. Pathogen spillover driven by rapid changes in bat ecology. Nature. 2023;613(7943):340-4. doi: 10.1038/s41586-022-05506-2

17. Bonesi L, Palazon S. The American mink in Europe: status, impacts, and control. Biological conservation. 2007;134(4):470-83. doi: 10.1016/j.biocon.2006.09.006

18. Salgado I. Is the raccoon (Procyon lotor) out of control in Europe? Biodiversity and Conservation. 2018;27(9):2243-56. doi: 10.1007/s10531-018-1535-9

19. Lee KA, Klasing KC. A role for immunology in invasion biology. Trends in Ecology & Evolution. 2004;19(10):523-9. DOI: 10.1016/j.tree.2004.07.012

20. Cornet S, Brouat C, Diagne C, Charbonnel N. Eco-immunology and bioinvasion: Revisiting the evolution of increased competitive ability hypotheses. Evolutionary Applications. 2016;9(8):952-62. doi: 10.1111/eva.12406

21. Loxton KC, Lawton C, Stafford P, Holland CV. Reduced helminth parasitism in the introduced bank vole (Myodes glareolus): More parasites lost than gained. International Journal for Parasitology: Parasites and Wildlife. 2016;5(2):175-83. doi: 10.1016/j.ijppaw.2016.05.005

22. Diagne C, Galan M, Tamisier L, d'Ambrosio J, Dalecky A, Bâ K, et al. Ecological and sanitary impacts of bacterial communities associated to biological invasions in African commensal rodent communities. Scientific reports. 2017;7(1): 14995. doi: 10.1038/s41598-017-14880-1

23. Chalkowski K, Lepczyk CA, Zohdy S. Parasite ecology of invasive species: conceptual framework and new hypotheses. Trends in parasitology. 2018;34(8):655-63. DOI: 10.1016/j.pt.2018.05.008

24. Dunn AM, Hatcher MJ. Parasites and biological invasions: parallels, interactions, and control. TRENDS in Parasitology. 2015;31(5):189-99. DOI: 10.1016/j.pt.2014.12.003

25. Schmidt KA, Ostfeld RS. Biodiversity and the dilution effect in disease ecology. Ecology. 2001;82(3):609-19. doi: 10.1890/0012-9658(2001)082[0609:BATDEI]2.0.CO;2

26. Tierney PA, Caffrey JM, Vogel S, Matthews SM, Costantini E, Holland CV. Invasive freshwater fish (Leuciscus leuciscus) acts as a sink for a parasite of native brown trout Salmo trutta. Biological Invasions. 2020;22(7):2235-50. doi: 10.1007/s10530-020-02253-1

27. Keesing F, Ostfeld RS. Dilution effects in disease ecology. Ecology Letters. 2021;24(11):2490-505. doi: 10.1111/ele.13875

28. Kelly D, Paterson R, Townsend C, Poulin R, Tompkins D. Parasite spillback: a neglected concept in invasion ecology? Ecology. 2009;90(8):2047-56. doi: 10.1890/08-1085.1

29. Loxton KC, Lawton C, Stafford P, Holland CV. Parasite dynamics in an invaded ecosystem: helminth communities of native wood mice are impacted by the invasive bank vole. Parasitology. 2017;144(11):1476-89. doi: 10.1017/S0031182017000981

30. Diagne C, Ballesteros-Mejia L, Cuthbert RN, Bodey TW, Fantle-Lepczyk J, Angulo E, et al. Economic costs of invasive rodents worldwide: the tip of the iceberg. PeerJ. 2023;11:e14935. doi: 10.7717/peerj.14935

31. Meerburg BG, Singleton GR, Kijlstra A. Rodent-borne diseases and their risks for public health. Critical Reviews in Microbiology. 2009;35(3):221-70. doi: 10.1080/10408410902989837

32. Pedersen AB, Babayan SA. Wild immunology. Molecular ecology. 2011;20(5):872-80. doi: 10.1111/j.1365-294X.2010.04938.x

33. Han BA, Schmidt JP, Bowden SE, Drake JM. Rodent reservoirs of future zoonotic diseases. Proceedings of the National Academy of Sciences. 2015;112(22):7039-44. doi: 10.1073/pnas.1501598112

34. Jackson J. Immunology in wild nonmodel rodents: an ecological context for studies of health and disease. Parasite Immunology. 2015;37(5):220-32. doi: 10.1111/pim.12180

35. McManus A, Holland CV, Henttonen H, Stuart P. The Invasive Bank Vole (Myodes glareolus): A Model System for Studying Parasites and Ecoimmunology during a Biological Invasion. Animals. 2021;11(9):2529. doi: 10.3390/ani11092529

36. Diagne C, Granjon L, Gueye MS, Kane M, Niang Y, Tatard C, et al. Association between temporal patterns in helminth assemblages and successful range expansion of exotic Mus musculus domesticus in Senegal. Biological Invasions. 2020;22(10):3003-16. doi: 10.1007/s10530-020-02304-7

37. Stuart P, Paredis L, Henttonen H, Lawton C, Torres CAO, Holland CV. The hidden faces of a biological invasion: parasite dynamics of invaders and natives. International Journal for Parasitology. 2020;50(2):111-123. doi: 10.1016/j.ijpara.2019.11.003.

38. Marnell F, Looney D, Lawton C. Ireland Red List No. 12: Terrestrial Mammals. National Parks and Wildlife Service, Department of the Culture, Heritage and the Gaeltacht, Dublin, Ireland; 2019.

39. Stuart P, Mirimin L, Cross T, Sleeman D, Buckley NJ, Telfer S, et al. The origin of Irish bank voles Clethrionomys glareolus assessed by mitochondrial DNA analysis. The Irish Naturalists' Journal. 2007:440-6.

40. White TA, Lundy MG, Montgomery WI, Montgomery S, Perkins SE, Lawton C, et al. Range expansion in an invasive small mammal: influence of life-history and habitat quality. Biological Invasions. 2012;14(10):2203-15. doi: 10.1007/s10530-012-0225-x

41. Razzauti M, Galan M, Bernard M, Maman S, Klopp C, Charbonnel N, et al. A comparison between transcriptome sequencing and 16S metagenomics for detection of bacterial pathogens in wildlife. PLoS Neglected Tropical Diseases. 2015;9(8):e0003929. doi: 10.1371/journal.pntd.0003929

42. Hazel S, Bennett M, Chantrey J, Bown K, Cavanagh R, Jones T, et al. A longitudinal study of an endemic disease in its wildlife reservoir: cowpox and wild rodents. Epidemiology & Infection. 2000;124(3):551-62. doi: 10.1017/S0950268899003799

43. Davis S, Calvet E, Leirs H. Fluctuating Rodent Populations and Risk to Humans from Rodent-Borne Zoonoses. Vector-Borne and Zoonotic Diseases. 2005;5(4):305-14. doi: 10.1089/vbz.2005.5.305

44. White TA, Perkins SE. The ecoimmunology of invasive species. Functional Ecology. 2012;26(6):1313-23. doi: 10.1111/1365-2435.12012

45. Galan M, Razzauti M, Bard E, Bernard M, Brouat C, Charbonnel N, et al. 16S rRNA amplicon sequencing for epidemiological surveys of bacteria in wildlife. MSystems. 2016;1(4):e00032-16. doi: 10.1128/msystems.00032

46. Bouilloud M, Galan M, Dubois A, Diagne C, Marianneau P, Roche B, et al. Three-way relationships between gut microbiota, helminth assemblages and bacterial infections in wild rodent populations. Peer Community Journal. 2023;3. doi: 10.24072/pcjournal.243.

47. Behnke J, Barnard C, Bajer A, Bray D, Dinmore J, Frake K, et al. Variation in the helminth community structure in bank voles (Clethrionomys glareolus) from three comparable localities in the Mazury Lake District region of Poland. Parasitology. 2001;123(4):401-14. doi: 10.1017/S0031182001008605

48. Abbate JL, Galan M, Razzauti M, Sironen T, Voutilainen L, Henttonen H, et al. Pathogen community composition and co-infection patterns in a wild community of rodents. Peer Community Journal; 2024. doi: 10.24072/pcjournal.370.

49. Wickham H. Plyr 1.8.8 R package 2022 [Available from: https://CRAN.R-project.org/package=plyr.

50. Wickham H, Chang W, Henry L, Pedersen TL, Takahashi K, Wilke C, et al. GGplot2 3.4.3 R package 2023 [Available from: https://CRAN.R-project.org/package=ggplot2.

51. Scherer R. PropCIs R package 2018 [Available from: https://CRAN.R-project.org/package=PropCIs.

52. Pradel J, Bouilloud M, Loiseau A, Piry S, Galan M, Artige E, et al. Small terrestrial mammals (Rodentia and Soricomorpha) along a gradient of forest anthropisation (reserves, managed forests, urban parks) in France. Biodiversity Data Journal. 2022;10. doi: 10.3897/BDJ.10.e95214

53. Brooks M, Bolker B, Kristensen K, Maechler M, Magnusson A, McGillycuddy M, et al. glmmTMB 1.1.7 R package 2023 [Available from: https://CRAN.R-project.org/package=glmmTMB.

54. Dinno A. dunn.test: Dunn's Test of Multiple Comparisons Using Rank Sums 2024 [Available from: https://cran.r-project.org/package=dunn.test.

55. Bartoń K. MuMIn: Multi-Model Inference, R package 2023 [Available from: https://CRAN.R-project.org/package=MuMIn.

56. Hartig F, Lohse L. DHARMa 0.4.6 R package 2022 [Available from: https://CRAN.R-project.org/package=DHARMa.

57. Lüdecke D, Makowski D, Ben-Shachar MS, Patil I, Waggoner P, Wiernik BM, et al. Performance 0.10.4 R package 2023 [Available from: https://CRAN.R-project.org/package=performance.

58. Gray J, Kirstein F, Robertson J, Stein J, Kahl O. Borrelia burgdorferi sensu lato in Ixodes ricinus ticks and rodents in a recreational park in south-western Ireland. Experimental & applied acarology. 1999;23:717-29. doi: 10.1023/A:1006233700194

59. Gray J, Robertson J, Key S. Limited role of rodents as reservoirs of Borrelia burgdorferi sensu lato in Ireland. European Journal of Epidemiology. 2000;16:101-3. doi: 10.1023/A:1007642908546

60. Telfer S, Bown KJ, Sekules R, Begon M, Hayden T, Birtles R. Disruption of a host-parasite system following the introduction of an exotic host species. Parasitology. 2005;130(6):661-8. doi: 10.1017/S0031182005007250

61. Zintl A, McManus A, Galan M, Diquattro M, Giuffredi L, Charbonnel N, et al. Presence and Identity Of Babesia Microti In Ireland. Ticks and Tick-borne Diseases. 2023;14(6): 102221. doi: 10.1016/j.ttbdis.2023.102221

62. Poulin R, Morand S. The diversity of parasites. The quarterly review of biology. 2000;75(3):277-93. doi: 10.1086/393500

63. Chisholm RA, Fung T, Chimalakonda D, O'Dwyer JP. Maintenance of biodiversity on islands. Proceedings of the Royal Society B: Biological Sciences. 2016;283(1829):20160102. doi: 10.1098/rspb.2016.0102

64. Zintl A, Moutailler S, Stuart P, Paredis L, Dutraive J, Gonzalez E, et al. Ticks and Tick-borne diseases in Ireland. Irish Veterinary Journal. 2017;70(1):1-10. doi: 10.1186/s13620-017-0084-y

65. Boulanger N, Boyer P, Talagrand-Reboul E, Hansmann Y. Ticks and tick-borne diseases. Medecine et maladies infectieuses. 2019;49(2):87-97. doi: 10.1016/j.medmal.2019.01.007

66. Kirstein F, Rijpkema S, Molkenboer M, Gray J. The distribution and prevalence of B. burgdorferi genomospecies in Ixodes ricinus ticks in Ireland. European journal of epidemiology. 1997;13:67-72. doi: 10.1023/A:1007360422975

67. Zintl A, Zaid T, McKiernan F, Naranjo-Lucena A, Gray J, Brosnan S, et al. Update on the presence of Ixodes ricinus at the western limit of its range and the prevalence of Borrelia burgdorferi sensu lato. Ticks and Tick-borne Diseases. 2020;11(6):101518. doi: 10.1016/j.ttbdis.2020.101518

68. Flattery A, McKiernan F, Browne J, Gray J, Zaid T, O'Connor J, et al. The prevalence and distribution of Anaplasma phagocytophilum genotypes in Ixodes ricinus nymphs collected from farm- and woodland sites in Ireland. Ticks and Tick-borne Diseases. 2022;13(3):101928. doi: 10.1016/j.ttbdis.2022.101928

69. Colautti RI, Ricciardi A, Grigorovich IA, MacIsaac HJ. Is invasion success explained by the enemy release hypothesis? Ecology letters. 2004;7(8):721-33. doi: 10.1111/j.1461-0248.2004.00616.x

70. Eccard JA, Mazza V, Holland C, Stuart P. The timid invasion: behavioural adjustments and range expansion in a non-native rodent. Proceedings of the Royal Society B. 2023;290(2003):20230823. doi: 10.1098/rspb.2023.0823

71. White TA, Perkins SE, Heckel G, Searle JB. Adaptive evolution during an ongoing range expansion: the invasive bank vole (Myodes glareolus) in Ireland. Molecular Ecology. 2013;22(11):2971-85. doi: 10.1111/mec.12343

72. Bouilloud M. Impact De L’anthropisation Sur Les Liens Entre Les Communautés De Petits Mammifères, Leur Microbiote Et Les Dangers Zoonotiques: : PhD Thesis, Institut agro, Montpellier; 2023.

73. Eliot CP. The insect vector for the natural transmission of Eperythrozoon coccoides in mice. Science. 1936;84(2183):397-. DOI:10.1126/science.84.2183.397.a

74. Crystal MM. The mechanism of transmission of Haemobartonella muris (Mayer) of rats by the spined rat louse, Polyplax spinulosa (Burmeister). The Journal of parasitology. 1958:603-6. doi: 10.2307/3274543

75. Brian JI, Catford JA. A mechanistic framework of enemy release. Ecology Letters. 2023;26(12):2147-66. doi : 10.1111/ele.14329

76. Kelly TC, Sleeman DP, Fennessy GJ, Dillon A, Walton GA, Gray J. The Mammal Ticks (Acari: Ixodoidea) of Ireland: Their Distribution and Hosts. The Irish Naturalists' Journal. 2001;26(10):363-70.

77. Haukisalmi V, Henttonen H, Tenora F. Population dynamics of common and rare helminths in cyclic vole populations. The Journal of Animal Ecology. 1988:807-25. doi: 10.2307/5094

78. Laakkonen J, Oksanen A, Soveri T, Henttonen H. Dynamics of intestinal coccidia in peak density Microtus agrestis, Microtus oeconomus and Clethrionomus glareolus populations in Finland. Ecography. 1998;21(2):135-9. doi: 10.1111/j.1600-0587.1998.tb00667.x

79. Voutilainen L, Kallio ER, Niemimaa J, Vapalahti O, Henttonen H. Temporal dynamics of Puumala hantavirus infection in cyclic populations of bank voles. Scientific Reports. 2016;6(1): 21323. doi: 10.1038/srep21323

Click here to access/download;Figure;Fig 1 forest sites.tif

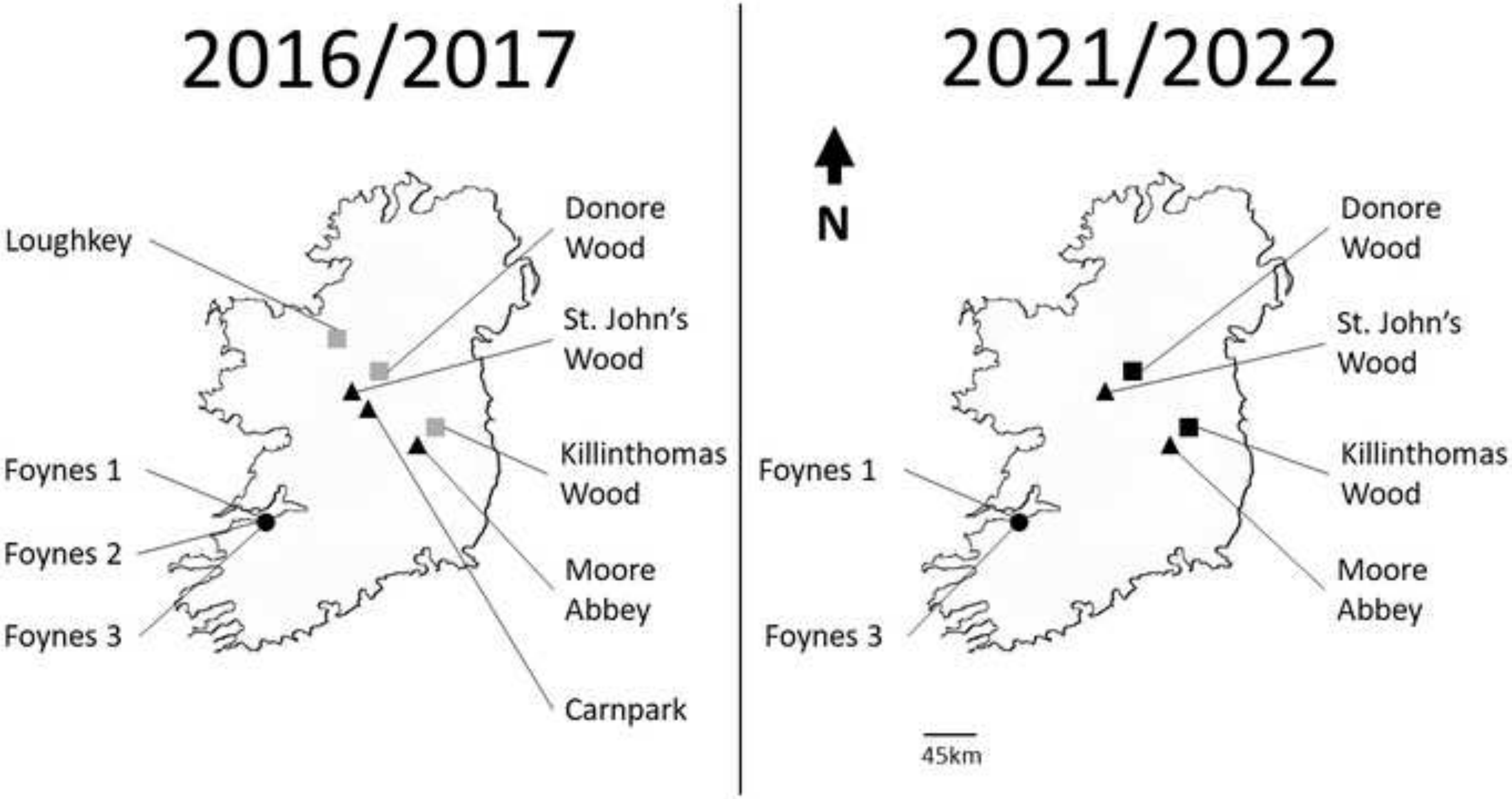

Click here to access/download;Figure;Fig 2 percentage clethrionomys.png

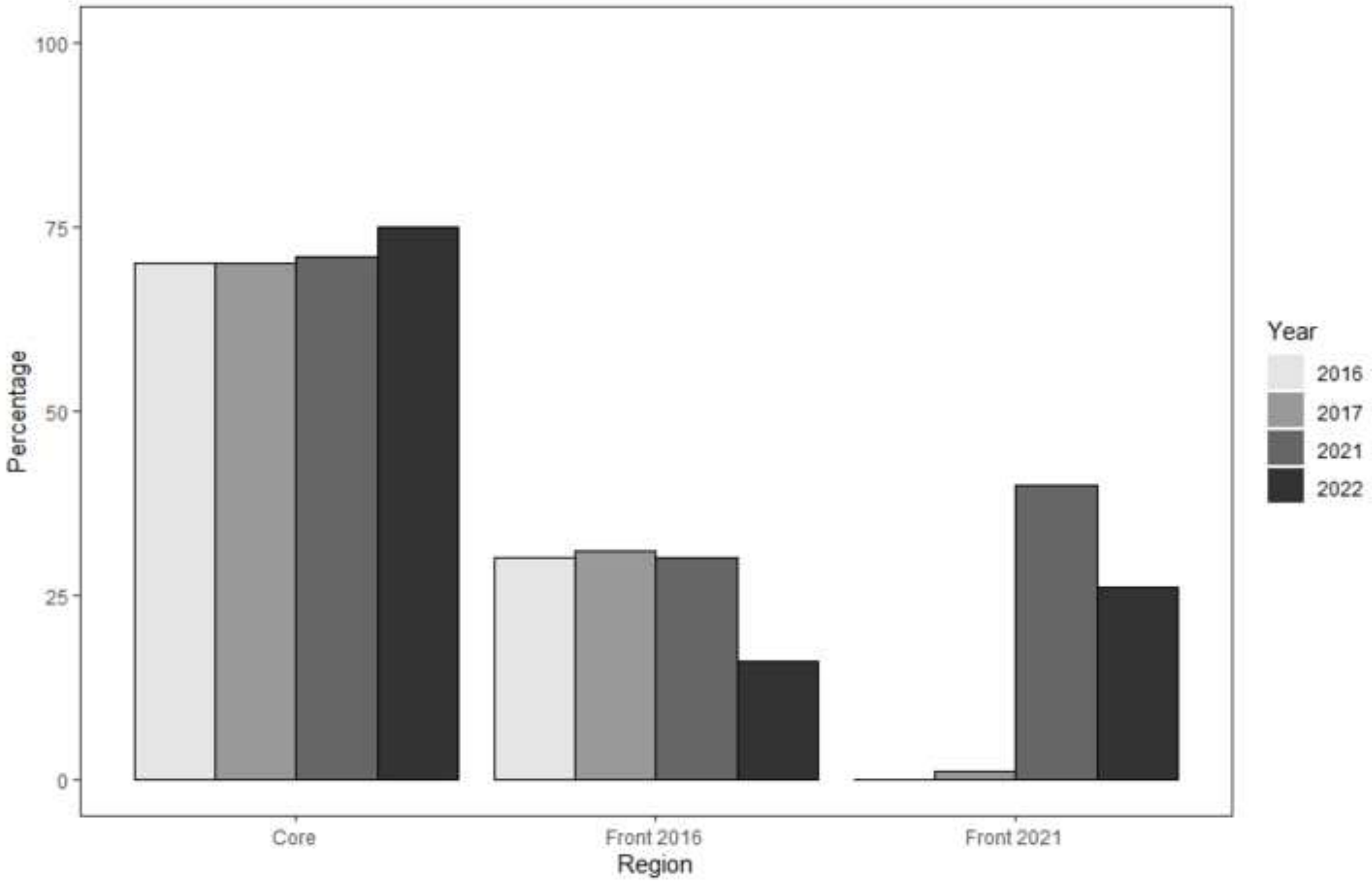

Click here to access/download;Figure;Fig 3 Ireland vs France axes fixed.tif

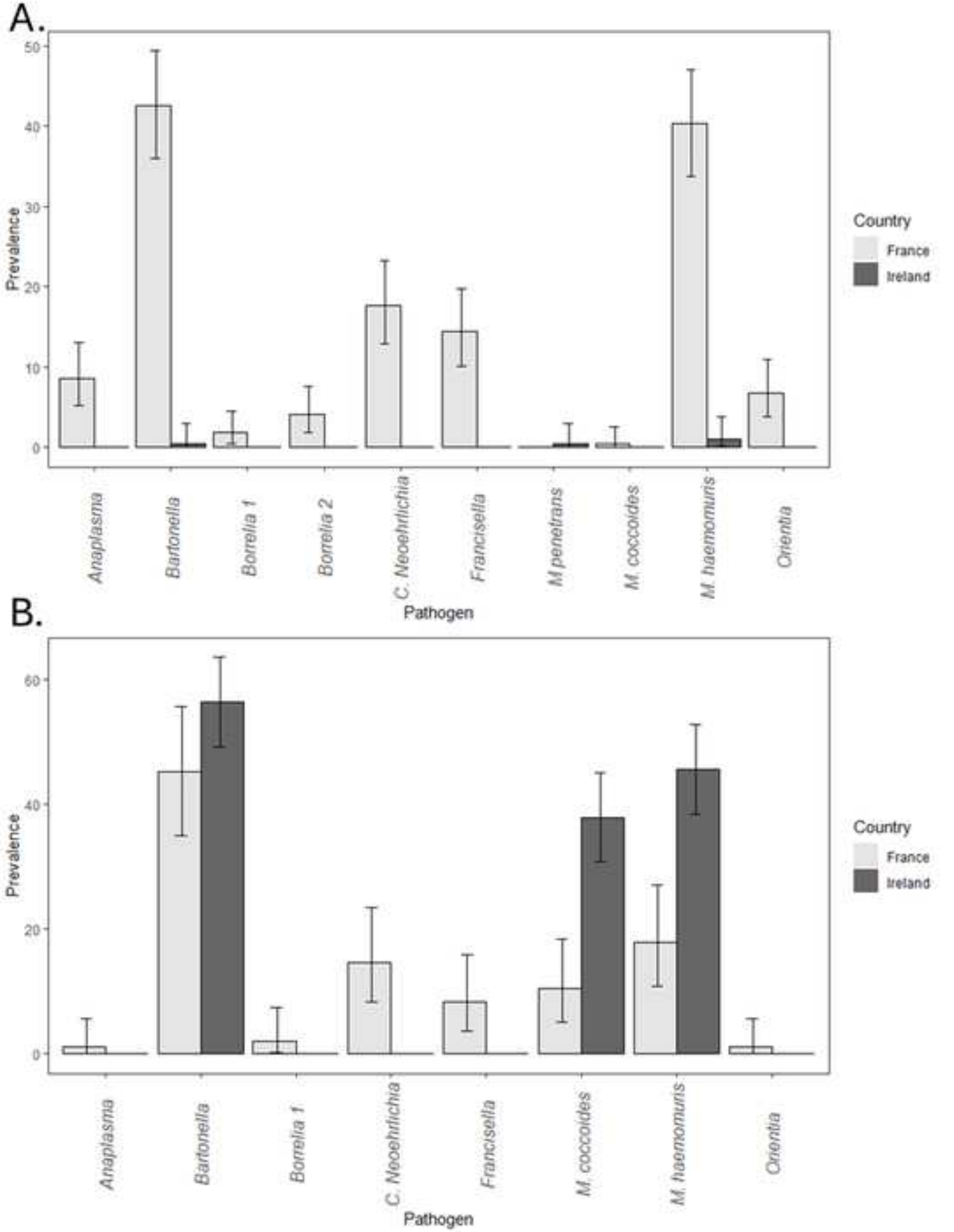

Click here to access/download;Figure;fig 4 Mycoplasma haemomuris longitudinal.png

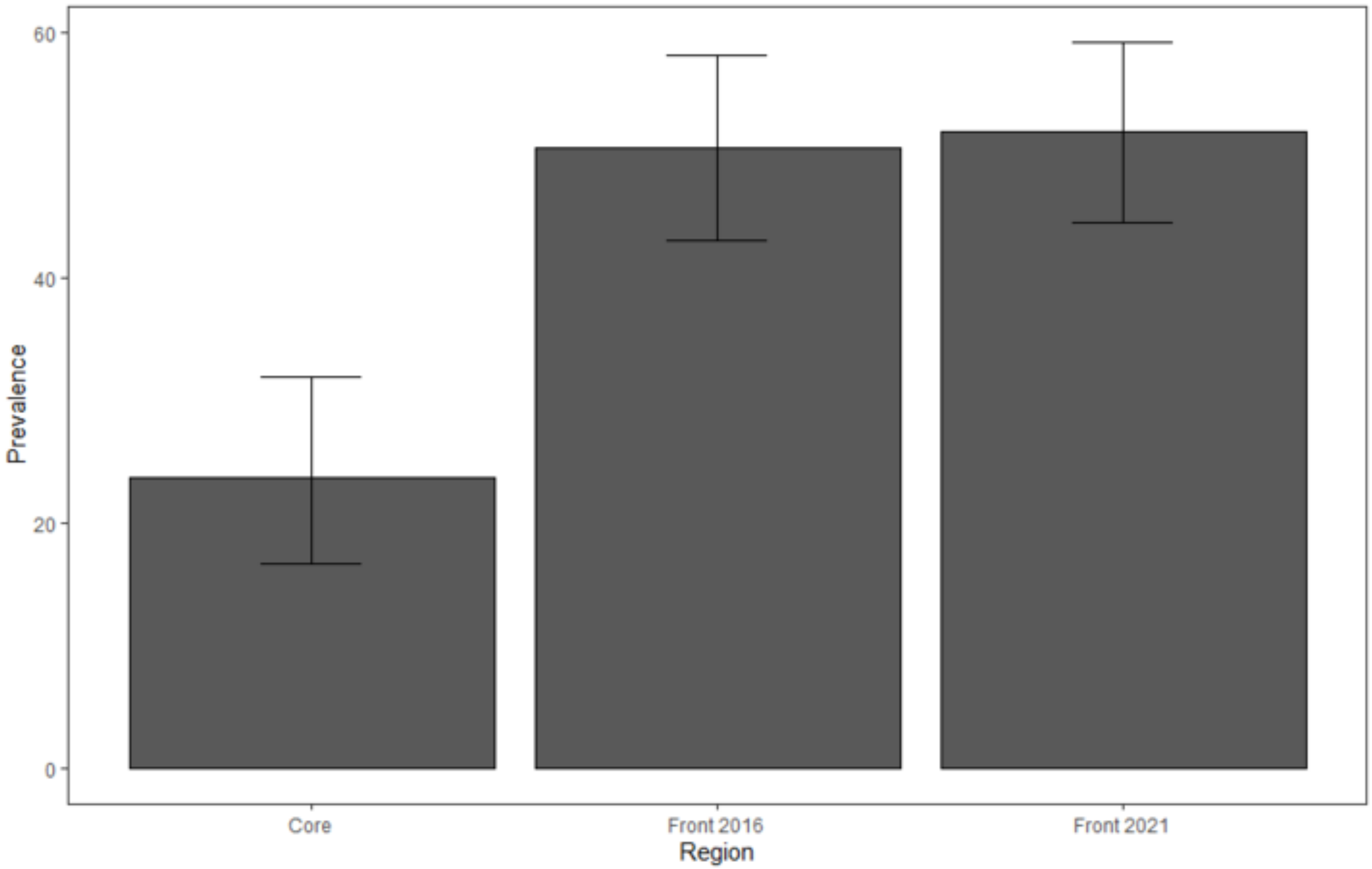

Click here to access/download;Figure;fig 5 mycoplasma coccoides fixed seasons order.png

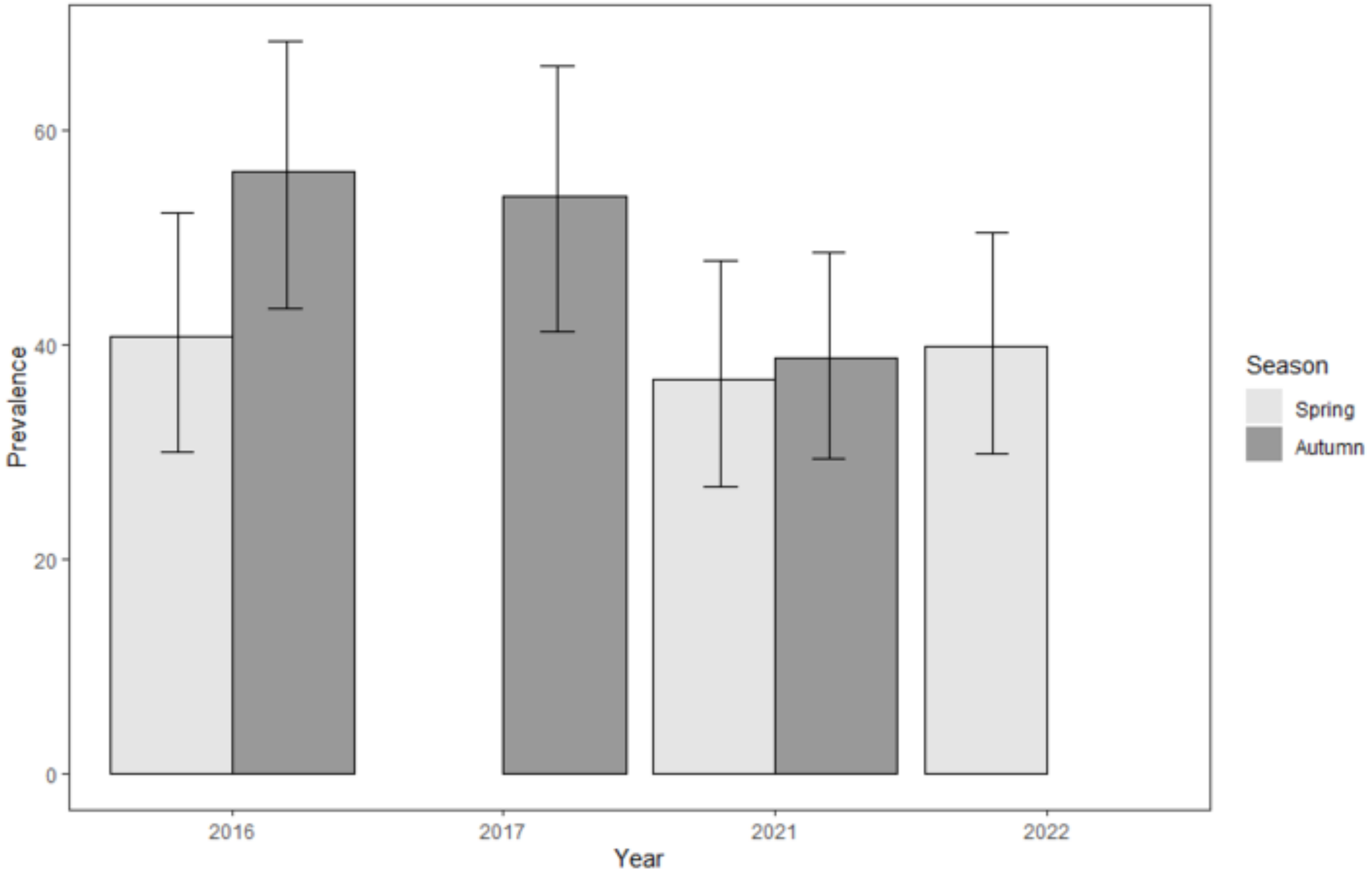

Click here to access/download;Figure;Fig 6 Sarcocystidae 2016 only.png

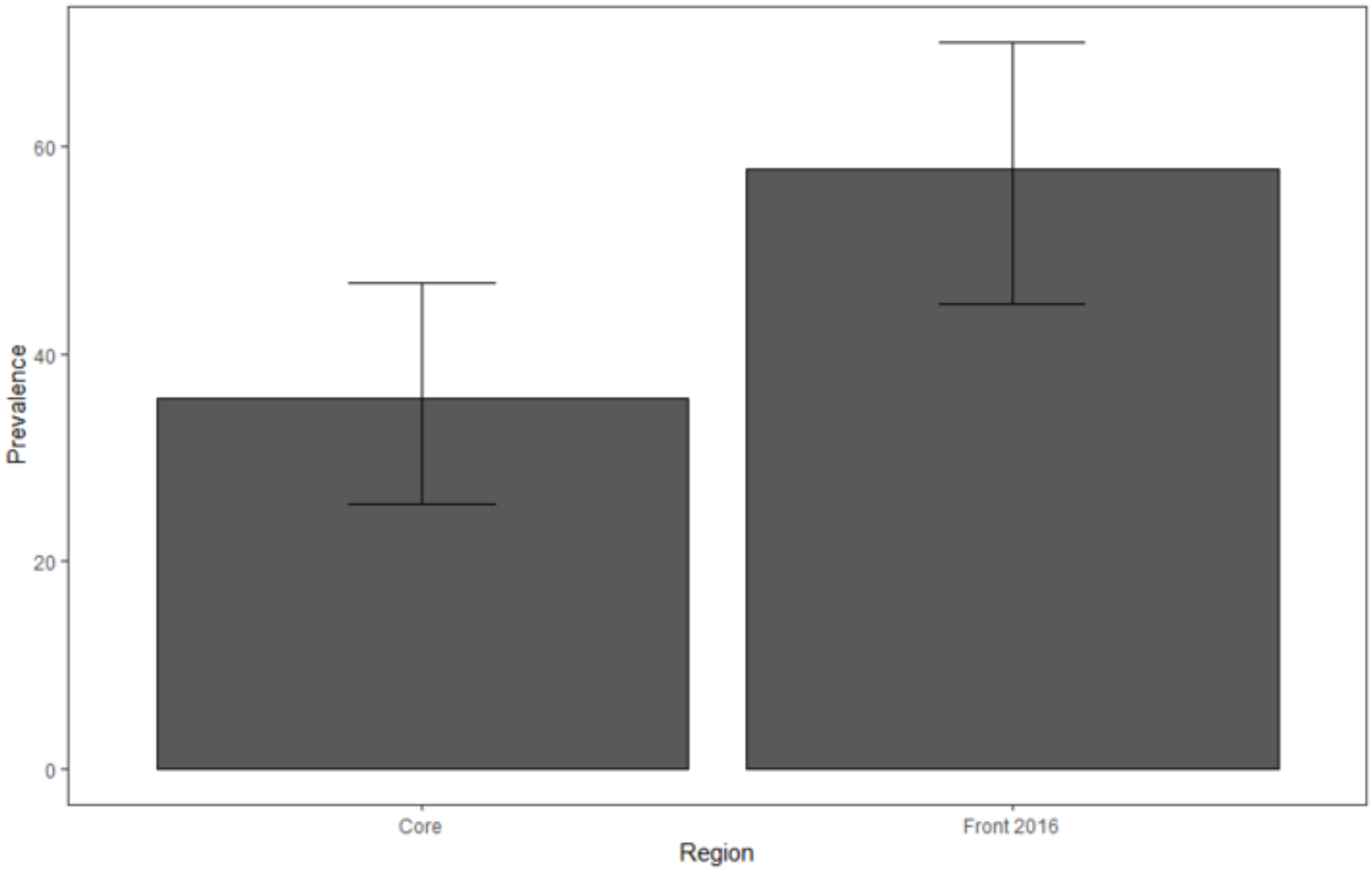

Click here to access/download;Figure;Fig 7 Sarcocystidae vole seasons order fixed.png

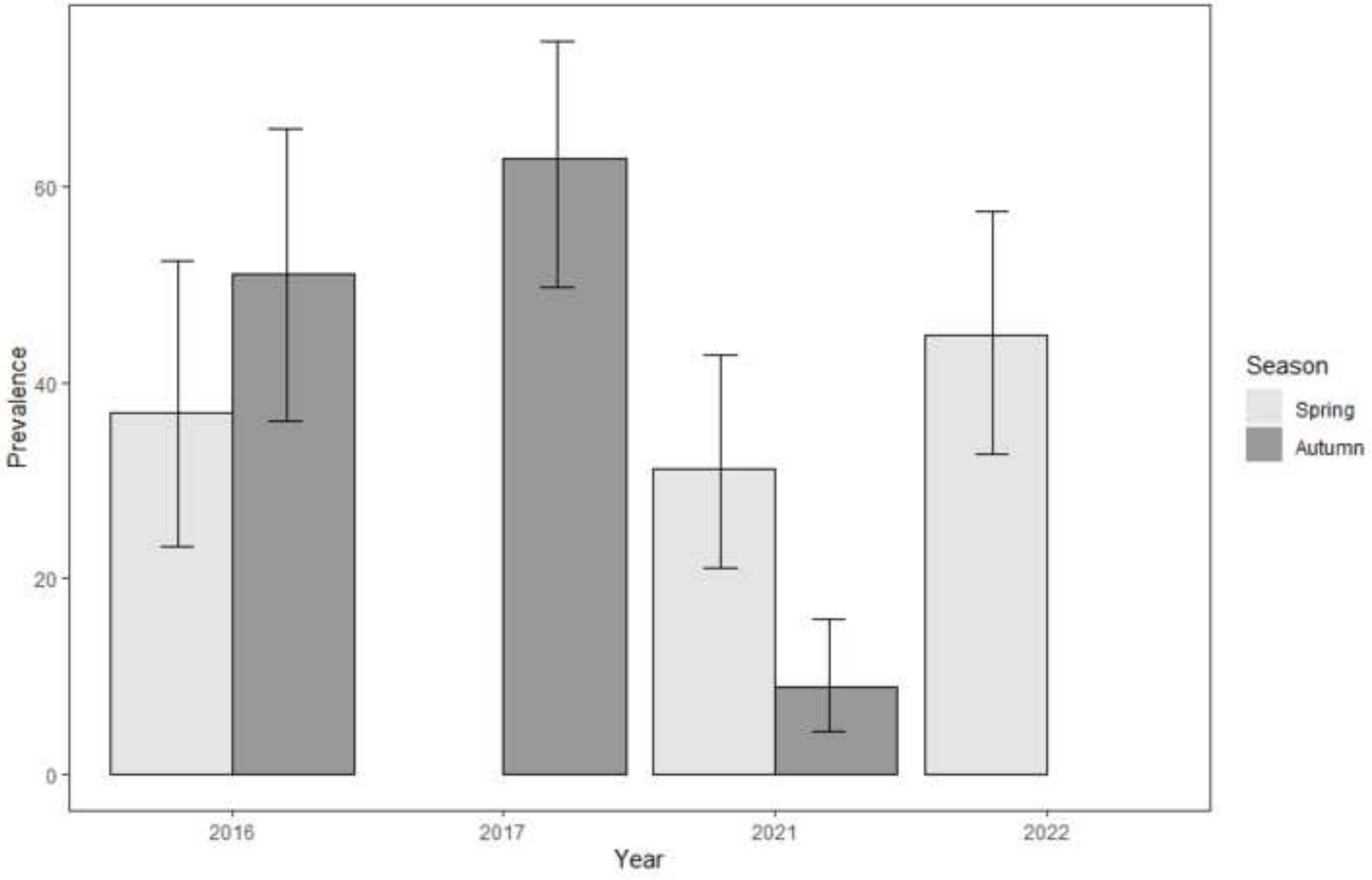

Click here to access/download
Supporting Information
Supplementary Table 1 - Field work dates.docx

Click here to access/download
Supporting Information
Supplementary Table 1 - Field work dates.xlsx

Data Review URL

Click here to download Data Review URL
http://zenodo.org/records/14883529
