# Supplementary material for: Longitudinal study of a rodent invasion reveals shifts in host pathogen interactions
Source: PLoS Pathog. 2026 Jul 23;22(7):e1014349. doi: 10.1371/journal.ppat.1014349 (PMC13395368; doi:10.1371/journal.ppat.1014349)
Supplement: Attachment — Submitted filename: PPATHOGENS-D-25-01038_R1_reviewer.pdf [file ppat.1014349.s006.pdf]

PLOS Pathogens

Longitudinal study of a rodent invasion reveals shifts in host pathogen interactions.

--Manuscript Draft--

**Manuscript Number:**	PPATHOGENS-D-25-01038R1
**Full Title:**	Longitudinal study of a rodent invasion reveals shifts in host pathogen interactions.
**Short Title:**	Longitudinal study of a rodent invasion reveals shifts in host pathogen interactions.
**Article Type:**	Research Article
**Section/Category:**	Evolutionary Biology - Bacteria
**Keywords:**	Zoonotic pathogens, epidemiology, rodents, enemy release, 16S rRNA metabarcoding, biological invasions
**Abstract:**	Invasive species can disrupt native epidemiological processes, potentially leading to the emergence of zoonotic pathogens. The relatively recent appearance and spread of the bank vole (Clethrionomys glareolus) in Ireland provides a unique model system to study these phenomena. The depauperate small mammal community in Ireland, combined with baseline data, allows us to identify the invasion gradient of C. glareolus and its effects on rodent-borne pathogens over time. A total of 498 C. glareolus and 584 Apodemus sylvaticus were sampled, via removal trapping, across nine sites in Ireland in 2016 and 2017, with six sites revisited in 2021 and 2022. 16S rRNA metabarcoding identified 10 putative pathogenic Operational Taxonomic Units (OTUs) present in these rodents, relating to four bacterial taxa, Bartonella spp., Mycoplasma coccoides, Mycoplasma haemomuris and Mycoplasma penetrans, and one family of protozoans, Sarcocystidae. Several epidemiological processes were found to be correlated with the C. glareolus invasion; firstly, C. glareolus in Ireland exhibited patterns consistent with the enemy release hypothesis, compared to native populations in France, and the native A. sylvaticus in Ireland. Secondly, a potential dilution effect, with reduced prevalence of M. haemomuris in A. sylvaticus at the C. glareolus invasion core compared to the invasion front, was observed. Finally, C. glareolus had increased prevalence of Sarcocystidae at the invasion front when compared to the invasion core, depending on the time and stage of invasion. These findings further our understanding of pathogen dynamics during biological invasions, demonstrating that invaders affect native host-pathogen communities differently as they advance through various stages of establishment.
**Additional Information:**	
**Question**	**Response**
**Government Employee**<br><br>Are you or any of the contributing authors an employee of the United States government?<br><br>Manuscripts authored by one or more US Government employees are not copyrighted, but are licensed under a CC0 Public Domain Dedication, which allows unlimited distribution and reuse of the article for any lawful purpose. This is a legal requirement for US Government employees.	No - No authors are employees of the U.S. government.

Powered by Editorial Manager® and ProduXion Manager® from Aries Systems Corporation

This will be typeset if the manuscript is accepted for publication.	
**Financial Disclosure**<br><br>Enter a financial disclosure statement that describes the sources of funding for the work included in this submission. Review the submission guidelines for detailed requirements. View published research articles from *PLOS Pathogens* for specific examples.<br><br>This statement is required for submission and **will appear in the published article** if the submission is accepted. Please make sure it is accurate.<br><br>**Funded studies**<br>Enter a statement with the following details:<br>• Initials of the authors who received each award<br>• Grant numbers awarded to each author<br>• The full name of each funder<br>• URL of each funder website<br>• Did the sponsors or funders play any role in the study design, data collection and analysis, decision to publish, or preparation of the manuscript?<br><br>Did you receive funding for this work?	Yes
Please add funding details.<br>as follow-up to "**Financial Disclosure**<br><br>Enter a financial disclosure statement that describes the sources of funding for the work included in this submission. Review the submission guidelines for detailed requirements. View published research articles from *PLOS Pathogens* for specific examples.<br><br>This statement is required for submission and **will appear in the published article** if the submission is accepted. Please make sure it is accurate.	This research was funded through the 2018–2019 BiodivERsA joint call for research proposals, under the BiodivErsA3 ERA-Net COFUND programme, and with the funding organisations the EPA Research Programme 2021–2030 and cofunded by Agence Nationale de la Recherche (ANR), Research Foundation– Flanders (FWO), National Science Centre, Poland (NCN) and Deutsche Forschungsgemeinschaft (DFG) (https://www.biodiversa.eu/ AMcM, MG, NC,CH, VS, AZ, PS).<br>This project is funded under the EPA Research Programme 2021-2030. The EPA Research Programme is a Government of Ireland initiative funded by the Department of the Environment, Climate and Communications (project number 2019-NC-MS-10) (https://www.epa.ie/ AMcM).<br>2016 and 2017 sampling was funded by the Irish Research Council, Government of Ireland Postdoctoral Fellowship Programme and by the Marie Curie Research Grants Scheme, European Union, grant (749312, INVASIMMUN) (https://research.ie/ https://marie-sklodowska-curie-actions.ec.europa.eu/ PS).<br>The funders had no role in study design, data collection and analysis, decision to publish, or preparation of the manuscript.<br>The author, Andrew McManus, received a stipend to complete this research from the EPA Research Programme as part of their PhD studies.

Powered by Editorial Manager® and ProduXion Manager® from Aries Systems Corporation

**Funded studies**<br>Enter a statement with the following details:<br>• Initials of the authors who received each award<br>• Grant numbers awarded to each author<br>• The full name of each funder<br>• URL of each funder website<br>• Did the sponsors or funders play any role in the study design, data collection and analysis, decision to publish, or preparation of the manuscript?<br><br>Did you receive funding for this work?"	
Please select the country of your main research funder (please select carefully as in some cases this is used in fee calculation).<br>as follow-up to "**Financial Disclosure**<br><br>Enter a financial disclosure statement that describes the sources of funding for the work included in this submission. Review the submission guidelines for detailed requirements. View published research articles from *PLOS Pathogens* for specific examples.<br><br>This statement is required for submission and **will appear in the published article** if the submission is accepted. Please make sure it is accurate.<br><br>**Funded studies**<br>Enter a statement with the following details:<br>• Initials of the authors who received each award<br>• Grant numbers awarded to each author<br>• The full name of each funder<br>• URL of each funder website<br>• Did the sponsors or funders play any role in the study design, data collection and analysis, decision to publish, or preparation of the manuscript?<br><br>Did you receive funding for this work?"	IRELAND - IE
**Competing Interests**	The authors have declared that no competing interest exists.

On behalf of all authors, disclose any competing interests that could be perceived to bias this work.<br><br>This statement will be typeset if the manuscript is accepted for publication.<br><br>Review the instructions link below and PLOS Pathogens' competing interests policy to determine what information must be disclosed at submission.	
**Data Availability**<br><br>Provide a **Data Availability Statement** in the box below. This statement should detail where the data used in this submission can be accessed. This statement will be typeset if the manuscript is accepted for publication.<br><br>Before publication, authors are required to make all data underlying their findings fully available, without restriction. Review our PLOS Data Policy page for detailed information on this policy. Instructions for writing your Data Availability statement can be accessed via the Instructions link below.	Supplementary material includes raw data, this is attached as a Zenodo link in the manuscript.

Dear Editor and Reviewers,

We would like to thank you for taking the time to review our manuscript. All three reviewers comments have been consolidated below, with a numbering system attached in order to aid clarity. We have attached how we addressed your comments, and our replies in red below. Line numbers mentioned in our replies reflect the “tracked changes” document.

Yours faithfully,

The Authors

Reply to Reviewers' Comments:

Reviewer's Responses to Questions

Part I - Summary
Please use this section to discuss strengths/weaknesses of study, novelty/significance, general execution and scholarship.

Reviewer #1: Dear authors,

1. I was pleased to review this manuscript on the hidden dynamics of pathogen transmission in a biological invasion context. I think that the longitudinal study is extremely interesting and certainly deserves publication after a few issues are resolved.
My concerns are mainly about the statistical analyses, I have left detailed comments in the pdf attached, but I think that the methods are slightly confusing and lack of an input form an expert in statistics, which could improve the explanation and the rationale behind the chosen methodology and the reporting of the results. That said, I do not think the methodology is wrong, just needs to be reported in a better way, and there are some things to be clarified (see the comments in the document attached).

Thank you for taking the time to thoroughly review the manuscript and we are glad you agree it is worthy of publication. Thanks also for your useful suggestion on how to improve the manuscript. The statistical analysis section has been reworked to improve clarity as per comments 53, 57, 59 and 70. We have addressed these comments you and other reviewers have left on the manuscript here, please see the list below below:

2. Line 27: “were sampled” – comment “what do you mean sampled? removal trapping? which samples were collected?”

Replaced with: “were sampled, via removal trapping,” on line 28.

3. Comment: “specify the matrix otherwise 10 OTUs seem reductive”

The sequencing of spleens from the 1,082 rodents from Ireland presented in this study was carried out between 2016 and 2022, sometimes including samples from other countries outside the scope of this study, and the sequencing data were analysed in three stages over different periods. Details of the sequencing process are presented in the file

‘Sequencing_informations_spleen_samples.xlsx’ on Zenodo (https://zenodo.org/records/14883529). Thus, for the three batches of samples, we obtained:

- 2016, Batch No. 1 (379 samples): total of 761 OTUs representing 5,212,747 reads, including 10 putative pathogenic OTUs representing 4,604,340 reads (88% of the reads)
- 2017, Batch No. 2 (189 samples): total of 401 OTUs representing 5,099,867 reads, including 8 putative pathogenic OTUs representing 4,774,704 reads (93% of reads)
- 2022, Lot No. 3 (672 samples): total of 1,272 OTUs representing 10,566,359 reads, including 10 putative pathogenic OTUs representing 8,442,958 reads (80% of reads)

We believe that these details would considerably weigh down the description of the results, so we have decided not to include these numbers in the manuscript.

4. Line 31: “Several epidemiological processes were found to be correlated with the *C. glareolus* invasion.” – Comment “example? it is too vague even for an abstract”

This was rephrased so it links to the epidemiological processes that are described subsequently, with “firstly” on line 32 and “secondly” on line 34.

5. Strikethrough removing the second full stop on line 52.

This has been removed.

6. Line 66: “This can happen because the pathogen, or their vector, is absent from the founder population, or because the environment in the new range is unsuitable (19-23). This, in turn, may give invaders a competitive advantage over native species.”

Comment: “I think this should be slightly rephrased for clarity and expanded for readers who are not familiar with epidemiology. Also, maybe would be nice to add the publication where this theory was developed for the first time”

This was subsequently rephrased and and the Keane and Crawley (2002) and Torchin et al., 2003 references were added on lines 64-69, now reading: “However, non-native species can also benefit from a reduction in natural enemies, such as predators or pathogens, compared to conspecifics in their native range (and often compared to the native species), through a process known as “enemy release”(19, 20). In relation to pathogens, this can happen because the pathogen, or the vector responsible for its transmission, is absent from the founder population, or because the environment in the new range is unsuitable to sustain the pathogen or vector (21-25).”

7. Strikethrough removing “including among others” and replacing with “such as” on line 80.

This was accepted and now reads “such as” instead on line 82 of the tracked changes document.

8. Line 82: “this is a little awkward, please rephrase. maybe expand just a liitle bit”

This was expanded to provide extra clarification, and now reads as: “Finally, studies on rodent invasions have demonstrated the interplay between rodent pathogen interactions and biological invasions, for example, helminth studies in both Ireland and Senegal have

observed patterns of enemy release in invasive rodents when comparing sites of long established populations to more recently invaded sites (24, 31, 38, 39).” On lines 83-86.

9. Strikethrough removing “this is and replacing with a comma on line 88.

Line 86: insert “only” in this sentence.

These suggestions were accepted and inserted on lines 88/89, now reading: “(37), particularly due to the depauperate woodland rodent community in Ireland, that includes only the invasive *C. glareolus* and the native woodmouse, *Apodemus sylvaticus* (40).”

10. Line 91, comma inserted after Co. Limerick

Comma was inserted in the text here on line 94.

11. Line 99: “this paragraph is slightly clunky, maybe try to improve the connection among sentences; although, I understand what you are saying and it is correct”

This paragraph has been rephrased to improve connection among sentences starting on line 104, now reading as:

“The development of high-throughput sequencing, and in particular metabarcoding techniques, facilitates a more holistic approach to studying the rodent pathobiome (44). These methodologies provide a global picture of pathogen diversity within host species, improving our understanding of the relationship between host-parasite interactions and biological invasions. In the Irish context, rodent helminth parasite communities have been explored previously by Loxton et al. (23, 31) and Stuart et al. (39). However, no such data exists on pathogenic microparasites in this model system. Furthermore, there remains a knowledge gap concerning longitudinal studies focusing on changes in pathogen communities during biological invasions. Such studies are essential for fully exploring changes in host-pathogen interactions, such as pathogen loss in invasive species, as cross-sectional studies only capture a single timepoint of this dynamic process (37, 39, 45-47).

In this study, we aim to address these knowledge gaps by describing the community of microparasites that could be zoonotic or pathogenic for rodents, including bacteria and protozoa, of *C. glareolus* and *A. sylvaticus* in Ireland, through a longitudinal study.”

12. Line 111: the subject is rodents? if so, and rodents sampled....were subsequently sampled in 2017, 2021, and 2022.

Rephrased to acknowledge this on lines 116-118: “We performed a spatiotemporal analysis of this community along the invasion gradient described above, using rodents removed in 2016 from a previous study by Stuart et al. (39), with subsequent removal sampling at these sites in 2017, 2021 and 2022.”

13. Line 111, comma after “in this study” and line 112 “recently used by”

Comma and wording suggestions were inserted on lines 118-120, now reading: “In this study, we use the 16S rRNA amplicon metabarcoding approach developed by Galan et al. (48), and recently used by Bouilloud et al. (49).”

14. Line 114-119: “non need to capitalise firstly. I would use semicolon to separate the hypotheses with some sort or numbering (e.g. i, ii, iii, or any of your choice)” and “in the first hypothesis, pathogenic for the vole? or zoonotic?”

The hypotheses have been restructured as you suggested on lines 121, 123 and 125. Furthermore, the first hypothesis relates to all pathogenic species, hence the terminology “patterns consistent with the enemy release hypothesis”, the pathogenicity of pathogens found are later described in the results with improved references as per your comments.

Now reads as: “Focusing on putative pathogenic taxa recorded in samples collected between 2016 and 2022, this present study addresses three main hypotheses: (i) whether *C. glareolus* exhibits patterns consistent with the enemy release hypothesis, including a lower pathogen species richness of potentially pathogenic taxa when compared to its native range and the native *A. sylvaticus*; (ii) whether the presence of *C. glareolus* alters the pathogen communities present in the native *A. sylvaticus* as expected under the dilution or spillback hypotheses; finally (iii) whether pathogen prevalence changes over time in both rodent species, as the invader becomes established.”

15. Line 121: “this section might benefit a reorganization of the sections, splitting it into defined data/sample collection, molecular data analysis, statistical analysis

also, it is less well written than the introduction. it is difficult to follow at times, and there are redundant sentences, especially the stat section need same polishing”

Rephrased and reorganised the methods section entirely (lines 128-261), see your comment below for details.

16. Line 132: “it needs to be clear that is removal trapping, and all ethical and technical aspects related to removal should be mentioned (or refer to the methods in another publication)”

Rephrased on line 141 to read: “A maximum of 24 animals from each species were removed from each site per season and year of sampling.”

As for the ethics, this is stated clearly in the designated section as per the publishers formatting on lines 820-824 on the tracked changes document.

17. Line 165: I know that there are references, but it be worth expanding this a little bit to specify how and why you filter which (presumed) pathogens

This has been rephrased to clarify they were identified based on their taxonomic affiliations on lines 169-179, now reading: “From the clusters of microorganism DNA detected, putative pathogenic OTUs were filtered and selected (51). First, we discarded putative false positive results associated with sequence counts below two OTU-specific thresholds, which checked respectively for putative cross-contamination between samples during DNA extraction and PCR steps on the one hand, and incorrect assignment due to the generation of mixed clusters on the flowcell during Illumina sequencing on the other hand (52). Then, for each sample, and each OTU, we kept only the occurrences confirmed by the two PCR replicates of the same sample. Finally, the putative pathogenic OTUs were selected using their

taxonomic affiliations and based on their potential zoonotic risk and pathogen impact on rodents, to separate them from commensal species (51).”

18. Line 165: so, what did you compare? the overall OTUs? which test? did you have the raw data from France?

This has been clarified that no statistical analysis could be conducted due to differences in how the data and rodents were processed on lines 193-201, now reading: “Firstly, bacterial data collected from *C. glareolus* and *A. sylvaticus* in Ireland from 2021 was compared to data collected from rodents trapped in forest sites in France, sampled in 2021 by Pradel et al. (56), using the same 16S metabarcoding approach (48). These data were examined by comparing prevalences of potentially pathogenic bacterial taxa in rodents from Ireland and France, to examine if less species were detected and at a lower prevalence in Ireland, related to the enemy release hypothesis. However, due to differences in assigning age and maturity to animals in each country, statistical analysis could not be conducted. Likewise, while the bacterial pathogens were filtered using the same methodology, two extra species of potentially pathogenic Sarcocystidae were included in the Irish data, and consequently meant that these species could not be included in the comparison.”

19. Line 172: is that reported elsewhere? do you think you might have missed something interesting or important?”

This has been replicated in studies by Diagne et al. – The logic in this is that prevalences below 5% would fall into the margin of error and proper statistical analysis was not feasible. In this instance, the prevalences below 5% are a handful of individual positives out of the entire dataset and statistical analyses would be extremely zero inflated. In this study, 498 *C. glareolus* and 584 *A. sylvaticus* were sampled across four years, therefore, we are confident that all patterns of infection were explored.

20. Line 183: “is this the same analysis as the comparison with French data?”

Clarified above that no statistical analysis was conducted. This section has also been rephrased to improve clarity as per comment 53 below.

21. Line 187: “baseline time should be T0, but 1 is also fine, bu it would be clearer to keep the variable name year and then use T1, T2, etc..”

This has no impact on the analysis or interpretation, however baseline as year 1 was chosen to avoid any complications regarding zero inflation should it have arisen.

22. Line 189: “I think I get it but it is a little awkward and also, as this percentage is not included in the models it is confusing here. What was the rationale behind this analysis?” and line 196: “What do you mean?”

Invasion region as a category was used as a proxy for the “percentage of *Apodemus* relative to C. glareolus” in order to quantify the effects of the invasion gradient, as the bank vole overtakes the woodmouse in numbers. Hence the assigned value for percentage *Apodemus* to each sample was tested against region to verify if our classification of region holds true for the analysis, this has been rephrased and moved to a better location on lines 246-255 to provide clarity:

“These analyses used the categorical variable of region as a proxy for invasion levels *of C. glareolus* relative to *A. sylvaticus* to aid in the interpretation of the results and increase statistical power through increased number of samples per variable. In order to justify our classification of invasion region in the analysis, the percentage of *A. sylvaticus* relative to *C. glareolus* was tested against invasion region. For these analyses, the total number *A. sylvaticus* relative to *C. glareolus* trapped in each site in each season within a given year was calculated, generating a percentage of *A. sylvaticus* relative to *C. glareolus*. These numbers were then assigned to each sample corresponding to their trapping site, season and year. Finally, to justify the classification of regions, this assigned percentage of *A. sylvaticus* relative to *C. glareolus* in the dataset was tested against region using a Kruskal-Wallis test, and a post-hoc Dunn’s test, through the dunn.test R package (58), to check differences between regions in both 2016 only, and the longitudinal dataset, as the data followed a non-normal distribution.”

23. Line 206: “This not clear to me”

In the case where GLMMs could not be fitted, non-parametric tests were conducted, where the data does not follow a normal distribution. This was the case with the pathogen species richness models; hence non-parametric tests were used to verify model findings.

This clarification was added on line 242 and now reads “In cases where no GLMM could be fitted, non-parametric tests were used, such as a Wilcoxon Rank Sum test.”

24. Line 210: “It would be good to include the formulation of the model to highlight the response variable and the distribution used (I see they are in the text, but an equation or even the R prompt would be great for clarity)”

Adding these would introduce complexity to table where the intent of this figure was to provide a summary of the factors and portions of the datasets used in each analysis (i.e. a reference for all factors investigated when interpreting the results section) and has been left as such. The model formation is specified in the text itself, as different response variables/distributions are used depending on the data being analysed, e.g. negative binomial for pathogen species richness and binomial for pathogen prevalence. The response variables and distributions used are explained in lines 203-212. While the strategy for each analysis (invasion gradient and longitudinal) are explained from lines 213 to 245. Additionally, the distribution family used for each GLMM is also repeated in the results section to aid the reader.

25. Comment on same table: “this is a result”

Sample sizes removed and put with the model summary table 3 on line 289 and explanation added below on line 292: “n represents sample sizes in the full model for each analysis”.

26. Line 215: “once the methods are organised please follow the same order for the results maybe the overall section is a bit too long”

This has been rephrased and restructured, the new section is broken up into each analysis and then the results within it to help break up the text better. A detailed description of the restructuring is provided below in comment 53.

27. Line 223: “maybe in the intro you should cite some studies demonstrating that voles tend to overtake mice population to understand this point”

This has been added with appropriate references on lines 97-101: “Furthermore, previous studies have shown a reduced relative abundances of *A. sylvaticus* at sites invaded *by C. glareolus* (39, 43). This has allowed for the establishment of cross-sectional studies with an invasion gradient, containing sites from the invasion core (established from the introduction point, where *C. glareolus* exhibits it’s highest relative abundance), invasion front (sites where *C. glareolus* had most recently invaded), and uninvaded sites (*A. sylvaticus* only) (39).”

28. Line 229: “this shows it the other way round compared to the methods. also does 100% is the total population of voles+mice or only mice? this figure is unclear to me”

The data itself was analysed as percentage of *Apodemus* relative to *C. glareolus* as this was the measure we used to identify how invaded a site had become. However, it was chosen to display the progress of the *C. glareolus* invasion using the percentage of *C. glareolus*, relative to *A. sylvaticus* (the direct inverse). This more clearly shows how invaded the regions were as 0% *C. glareolus* is more straightforward than 100% *A. sylvaticus.* Additionally, the 100% represents the total rodent community, i.e. at the core it is dominated by *C. glareolus* and approximately 75% in 2022, the remaining approximate 25% are *A. sylvaticus*. Therefore, the front 2021 in 2016 showing 0% *C. glareolus* means the site is 100% *A. sylvaticus.*

29. Line 236: “references? also these adds up to 9”

This has been rechecked, and these add up to 10 OTUs. References have been added for the pathogenicity of the mycoplasmas: “These included one OTU of the genus *Bartonella*, a bacterial genus potentially including zoonotic species (e.g. *B. henselae*) (62), three OTUs of *Mycoplasma haemomuris* (combined for analysis), one OTU of *Mycoplasma coccoides* and one OTU of *Mycoplasma penetrans* (known to be zoonotic). These three *Mycoplasma* species have traditionally been associated with pathology in rodents (63-65). While more recent studies suggest that *Haemoplasma* may be largely subclinical in wildlife they have also highlighted the need for further studies increasing their geographic range and elucidating transmission (66). Finally, we detected four OTUs of Sarcocystidae (combined for

analysis, a family of protozoans, with some species that are zoonotic, and others that are pathogenic for rodents) (67, 68).

30. Line 247: “this recurrent terminology is very vague and needs to be clarified in the methods (see relative comment)”

The term potentially pathogenic is used due to the small fragments of DNA from the 16S methodology. The terminology itself has been clarified in the methods section and used consistently throughout to help clarity: “described by Galan et al. (48). From the clusters of microorganism DNA detected, putative pathogenic OTUs were filtered and selected” and “Separate putative pathogenic OTUs of the same pathogen species were combined into potentially pathogenic taxa for analysis” on lines 169 and 183.

31. Line 253: this table is not the best way to report modelling results. information regarding metrics usually used to assess model fit and variance explanation proportion are missing. also the table is not cited in the text

This summary table is intended as a brief summary of the statistically significant factors in a concise table, allowing the reader to use this as reference for subsequent results and discussion sections. The Z, P and AICc values are added throughout the text to help aid these results, likewise the table is now referenced throughout the results section, including adding “In the autumn only analysis, the best model included only region (lowest AICc value; 322.788), however, no factor significantly influenced *Bartonella* prevalence.” to line 533-534, to further clarify the model.

32. Line 260: this section should be put before the modelling results, and the latter need to be further expanded beyond table 3. Also, figure 3 needs asterisks (or another appropriate symbol to visualise significant differences among each of the coupled bars)

The results section has been restructured to bring the pathogens found to the top of the results section, with the comparison with France directly below this. Asterisks cannot be added as statistical analysis was not conducted as per comments on the statistical analysis section. The comparison with the French samples is still left below the modelling table 3 as this is a summary of the overall results found in the Irish samples and is most appropriate at the top of the results section. However, the enemy release section is discussed before the modelling results themselves for pathogen species richness and pathogen prevalence in each host spp.

33. Line 277: maybe this section would be appropriate for a restructured table 3

The entirety of the results section has now been restructured to reflect the statistical analysis section. Table 3 has been left as is, so as to provide a simple summary for readers as per above comment.

34. Lines 278 and 286: “it is redundant to present both” and “again showing both is redundant, and maybe, if there is poot fit, the problem lies in the structure and type of data, you do not solve it changing test.”

Both were kept as the final models were observed to show poor fit, therefore non-parametric tests were used to confirm our findings (following tests of normality for the data). See comment above regarding non-parametric tests.

35. Line 294: “these results (all sections regarding statistical analyses) might have been better displayed using a table like table 3 (which should be mentioned here I guess), but with AIC, z and p values for each model and each factor” and “the reporting of statistical results is confusing and difficult to follow, I am not sure the methodology fully matches the reported results. the methods and results section regarding statistical testing, although might not be entirely wrong might benefit from a review from an expert in statistics. I think some tests are redundant and the result section might be streamlined with better tables and figures”

These have been explained as text in order to help understand the differences between individual groups, i.e. it can be explained if juveniles are more infected, or which region etc. The table only provides a summary of the statistically significant factors to aid as a reference for the discussion, as the results are quite long. The statistical analysis section has been reorganised alongside the results section to reflect the same order and layout to improve clarity.

For example, the section on *Bartonella* during the invasion gradient now reads as: “Considering the invasion gradient analysis, the best model (lowest AICc value; 271.876) included season and sex (Table 3). *Bartonella* prevalences were higher in autumn compared to spring (GLMM, family = Binomial, $z = -2.741$, $p = 0.00612$), while males were more infected than females (GLMM, family = Binomial, $z = 2.404$, $p < 0.05$).”

36. Line 303: Why not keep just one of the two?

Both models were kept due to the nature of these data being analysed; 2017 and 2022 only have a single season, consequently these variables were inherently linked. Consequently, a high VIF was observed with the interaction between the two variables. Therefore, separate models were run on each season so the effect of time during spring and the effect of time on autumn data could be explored, which did show different patterns of infection, based on the season being focused upon.

37. Line 337/338: “please place significance letters oor symbols on top of the bars” and strikethrough on “(Maximum and Minimum)”

The asterisks have been added to the factor that is statistically significant, on line 551 of the tracked changes document. Likewise, the maximum and minimum has been removed on each figure but a single clarification added to the statistical analysis section instead to make up for this, on line 186: “Confidence intervals (maximum and minimum) were calculated to

95% using the R package PropCIs (55).” This has also been done for the Sarcocystidae figure 4 on line 476.

38. Line 340: “same comments apply here, also for the figure” and “I do not understand why you did GLMM, correlation, non parametric tests, post-hoc etc. all together”

The figure has been kept as described below relating to seasonal figures. Asterisks weren’t added as the significant factor relates to time, which is shown per year and season here, to reflect that some years only contain one season’s data.

Post-hoc tests were used to determine the differences between groups with more than two variables. In the case of the correlation test, this was done post-hoc to show the correlations of the levels of certain pathogens with time.

39. Line 360: “and the other pathogens? table 2 showed some prevalence”

Prevalences are below 5% and in the case of this dataset, that is only a handful of individuals, therefore it would be inappropriate to conduct statistical analyses on these pathogens (as per comment 19.

40. Line 364: “overall the section is difficult to follow, maybe too much was tried to convey, while more target results, functional to the hypotheses would have been better. Another option would be to summarise the results in some tables”

The section has been split up based on the new results section structure as per comments below into invasion gradient and longitudinal analysis. Splitting this up should break up the results to allow for better understanding. Likewise, the statistically significant results are summarised in Table 3.

As with previous comments, the statistical analysis was chosen based on the hypotheses, while confounding factors still need to be considered, as season, sex, age etc can all have effects on pathogen prevalence and need to be fully explored to determine if the hypotheses hold true.

41. Line 415: interesting, good references, maybe need a little bit of restructuring and streamlining to convey the main messages more effectively.

Rephrasing and streamlining of text in the discussion section from lines 608 to793 to improve flow. Your specific comments on this section have been addressed through the comments directly below:

42. Line 416: a bit clunky, can be rephrased to improve flow and Line 422: the number should suffice

This has been rephrased, now on line 609-617 on the tracked changes document, to improve clarity and reduce clunkiness. The text and date for Zintl et al. (2023) was removed and reference number kept also:

“The aim of this study was to investigate the effect an invasive species may have on native pathogen communities, including zoonotic species, using the bio-invasion of Ireland by C. glareolus as a model system. While previous studies on rodent-borne microparasites in Ireland have focused on single bacterial species, such as Bartonella or Borrelia (70-72), this is the first time 16SrRNA metabarcoding techniques have been used to identify the full communities of potentially zoonotic pathogens, and their prevalence levels, that are circulating in Irish populations of C. glareolus and A. sylvaticus. Furthermore, this study has created an important baseline for rodent-borne disease studies in Ireland, and highlights new important areas of research to guide future research in Ireland, and potentially other invasion systems (e.g (73)).”

43. Line 424: you keep changing names of sub-sections and combinations of words such as pathogen risk, zoonotic risk, infectious risk, etc. this is confusing. it is especially confusing whehter your focus is on pathogens pathogenic for rodents or zoonotic pathogen considered a human health risk. I suggest keep a coherent nomenclature throughout the manuscript and restructuring the sub-section headlines so that they match as much as possible in the methods, results, discussion, etc

The subsection here has been renamed to “Rodent-borne pathogens observed in Ireland” to clear up confusion on line 619. Additionally, this sub section has been kept in this location, as this is the logical first step to discuss what pathogens are observed in irish rodents. Followed by subheadings specific to the topics hypothesised, i.e. patterns of enemy release, whether the bank vole has an effect on the wood mouse pathogens, and finally whether time (as the bank vole becomes more established in Ireland) has an impact.

44. Line 426: do you have a reference? if you are mentioning France, then add the reference

This is a general statement based on the findings of the paper, we only detected 5 pathogen species in Irish rodents, therefore no reference is provided. In the next paragraph the comparison with the French samples is discussed.

45. Line 429: this is in the next section, why also here?

This sentence reading “Furthermore, we observed that the *C. glareolus* population in Ireland expressed patterns consistent with the enemy release hypothesis, and potentially disrupts the transmission of native pathogen species.” has since been removed to avoid repetition on lines 624-626.

46. Line 439: so not the effect of invasive voles?

Yes, while the bank vole was shown to potentially impact the prevalence of *M. haemomuris,* the depauperate community already present in the native wood mouse, would most likely be due to Ireland being an Island. Likewise, this would also possibly explain the lack of pathogens picked up by the bank vole.

47. Line 443: I think this is a missed opportunity for a greater pathogen diversity assessment

While we accept this is a limitation of the study, the data for this paper serves as a baseline for future work outlined in the discussion, and as discussed in papers like Zintl et al., 2023. The 16S methology was chosen in order to get a broad picture of all the potential pathogen species infecting the rodents involved in this invasion system, and to fully understand the patterns in these pathogen communities caused by the bank vole invasion (see comment 57 below for detailed explanation).

48. Line 458: please rephrase, I am not sure I understand

This was meant to explain that patterns consistent with the enemy release hypothesis were observed between the bank vole and wood mouse in Ireland, and between the bank vole in Ireland and its native range (France). This has been clarified by removing the phrase altogether and explaining the different patterns of enemy release, now reading as; "Two patterns consistent with the enemy release hypothesis were observed for bacterial pathogens in the invasive *C. glareolus* population in Ireland for the first time. Firstly, a comparison of the number of bacterial taxa in Irish and French *C. glareolus* populations indicated that the population of *C. glareolus* sampled in Ireland had fewer potentially pathogenic bacterial taxa than conspecifics in their native range." on line 653-656 on the tracked changes document.

49. Line 514: the main verb is missing, do you meant resulted instead of resulting?

This has been fixed by adding "was observed" on line 711, now reading: "However, no dilution or amplification effect resulting from the *C. glareolus* invasion gradient on pathogen species richness in *A. sylvaticus* was observed in the present study."

50. Line 554: this is the best section of the discussion and the most interesting, I would expand a bit. To make it more relevant I would very briefly mention the significance of each pathogen (effect on rodent survival, zoonotic potential, etc.)

While we appreciate that these are interesting topics, we worry that expanding this discussion would take away from the main messages/hypotheses of the paper, which is how invasive species, and time since invasion can have significant impacts on pathogen communities. Furthermore, we think that cross-sectional studies are not always sufficient to fully tease out the complex epidemiological processes during these invasions.

51. Line 580: careful that Sarcocystidae are not bacteria

This has been clarified on lines 776-778 that Sarcocystidae were not included in the comment regarding bacteria by changing the phrasing, now reading as: “Despite exhibiting higher prevalences of Sarcocystidae, *C. glareolus* in Ireland shows reduced pathogen species richness, and lower prevalences of bacterial taxa, compared to the native *A. sylvaticus* and fewer bacterial taxa compared to the native range of the species.”

52. Line 639: please double check the formatting, as there are some typos and minor errors, in particular most species names are not italicised (I highlighted some but not all), as well as capitalisation where is not needed and other minor mistakes

Thank you for pointing out these formatting errors. We have rectified these in the reference list through lines 836 to 1091. This included lowering sentence case where all caps had been inserted, making species names italic and the end bracket from the links to R packages (“]”).

In the case of reference 8, Daszak et al. the double hyphen is how the paper is titled, and the reference is correct.

The tracked changes include some details that were in the original submission (missing DOIs, some incorrect page numbers for references 24, 39 and 87, a missing “PhD Thesis” from 84, and formatting the indent to tidy up the reference block), that were added back in, as the endnote reference manager reformats every time new references were added.

53. Then, I think that the structure of methods, results, and consequently discussion should be improve to streamline the main message, and the sub-sections need to be restructured/reordered so that the reader can follow the different aspects through the various section of the manuscript.

Sub sections have been added and structured to provide clarity, including rephrasing of sentences to provide clarity (see lines128 to 179 for methods, line 180 to 261 for statistical analysis). Additionally, the results section has been restructured to follow the order of the statistical analysis section and break up the results into an easier format (lines 262 - 607), as follows:

Methods:

Field work & sample collection

Laboratory work & analysis

Statistical analysis

Calculation of prevalence and species richness

Examining patterns of the enemy release hypothesis

Examining patterns of infection in Irish rodents

Results

Pathogens detected in Irish rodent spleen samples

Patterns of infection relating to the enemy release hypothesis

Validation of invasion region criteria for statistical models & progresss of *Clethrionomys glareolus* invasion

Invasion gradient analysis

(includes pathogen richness and host specific model results)

Longitudinal analysis

(includes host specific model results)

54. Hence, I also left comments for the images that sometimes can be improved to be clearer and more immediate.

Seasonal figure 7 have been removed, however, figure 5 was kept (now figure 6) as it shows the change in prevalence over time, while acknowledging the seasonal limitations of the 2017 and 2022 data. Grayscale has been kept for accessibility reasons (addressed in a comment 78 below).

55. Minor issues regard the precision of some statement, the formatting of the reference list and other minor comments that can be found in the pdf attached.

These formatting issues in the reference section have been addressed in the above comment 52.

All the details are reported in the comments in the document attached.
I hope that my comments will improve the quality of the manuscript and will give it the relevance it deserves.

56. Reviewer #2: This interesting manuscript investigates pathogen dynamics in an invasive rodent through a longitudinal study conducted in both core invasion zones and the invasion front. It also examines the relationships between the invasive species, its pathogens, and a native rodent species.

The article provides valuable insights into invasive species and their associated parasites/pathogens within the framework of the enemy release hypothesis. The analysis is robust, supported by a solid sample size and state-of-the-art pathogen detection methods. The study is also well contextualized through comparisons with data from France. The authors appropriately acknowledge the study's limitations and outline relevant avenues for future research.

Thank you for taking the time to review out manuscript and we are glad you consider it a valuable contribution

57. Reviewer #3: Invasive species can influence epidemiological processes through a range of direct and indirect mechanisms, and gaining a better understanding of these dynamics is of clear value both theoretically and practically. McManus et al. have taken advantage of a unique "natural" experiment involving the recent spread of the bank vole (Clethrionomys glareolus) in Ireland to explore the epidemiological consequences of host invasion. Their study compares rodent-borne microbial taxa across a geographic gradient, including sites in France (the native range), as well as core and front areas of the invasion in Ireland. By

incorporating both cross-sectional and longitudinal data, and by comparing patterns in a second rodent species (Apodemus sylvaticus) that is native to both regions, the authors aim to disentangle potential effects of invasion history on microbial communities. Their findings suggest patterns that may be consistent with enemy release and dilution effects, and they also highlight temporal dynamics in pathogen prevalence along the invasion gradient.

I commend the authors for their creative and ambitious study design, the integration of multiple data types, and the rigorous statistical approach. However, I have concerns about whether the manuscript meets the criteria of originality, impact, and scientific depth expected for publication in PLOS Pathogens, which emphasizes advances in pathogen biology. In my view, the primary limitations stem from the reliance on 16S rRNA metabarcoding in a relatively small and well-characterized host–microbe system. Although this method is valuable in more complex microbial communities and could serve as a preliminary tool to identify candidate species in this context, its application here leads to limited taxonomic resolution. As a result, it constrains the ability to draw confident conclusions regarding microbial identity, pathogenic potential, and ecological significance, particularly given the lower sensitivity of this approach. Additionally, the statistical analyses, though comprehensive, feel exploratory rather than hypothesis-driven, which makes it difficult to pinpoint key results and interpret them with confidence. As such, I am not fully convinced that the study yields novel or robust insights beyond those already offered by prior work focused on specific pathogens within this system. For these reasons, I recommend that the authors consider submitting their manuscript to a journal with a broader ecological or invasion biology focus. I offer detailed comments below in the hope that they will help strengthen the manuscript in its next iteration.

We understand that the 16S rRNA metabarcoding has its limitations, and that it is well characterised in rodent systems. However, in the case of the Irish rodent invasion system, this is the first time any large-scale pathogen screening has been done, aside from studies on helminth systems or smaller studies focusing on specific pathogens. The 16S approach has been successfully applied when investigating pathogen communities in other invasion systems, such as the studies by Diagne *et al.,* in Senegal. We chose 16S rRNA metabarcoding over other species-specific assays for individual pathogens, as this helps identify the potential pathogenic taxa in this current invasion system, while also allowing for analysis of pathogen communities as a whole, rather than focusing on a single pathogen species at a time. It is clear from our investigation that in a longitudinal study of an invasion, there will be winners and losers amongst the pathogens and a 16S approach allows for these simultaneous patterns to be detected. We believe this longitudinal survey during an invasion was a knowledge gap that needed to be filled. The knowledge generated will be useful to future studies of pathogens in invasional systems by providing valuable information helping narrow down which taxonomic groups to further investigate specific pathogens in. This has already happened as a result of the present study (e.g Zintl et al. 2023 study on *Babesia microti*)

Relating to the statistical analysis, while it may appear to be exploratory in nature, the statistical analysis was designed with a focus of describing the effect of the bank vole invasion on pathogen communities in the native wood mouse, and the effect of the invasion on the bank vole's own pathogen species. We aimed to investigate both the effect of the *C. glareolus* invasion gradient, and the effect of time, as the invader becomes more established. Hence, we used separate analyses for these hypotheses; i.e. one using a single year where the invasion gradient was most prominent, with uninvaded *Apodemus* only sites; and a longitudinal study focusing on multiple years data but only the sites which had been repeated in 2021 and 2022. Finally, the statistical analysis also includes other confounding

factors such as age, sex, seasonality etc. which have all been proven to influence pathogen prevalence in other systems and hence need to be included in order to fully understand the ongoing epidemiological processes in the Irish rodent system.

Part II – Major Issues: Key Experiments Required for Acceptance
Please use this section to detail the key new experiments or modifications of existing experiments that should be absolutely required to validate study conclusions.

Generally, there should be no more than 3 such required experiments or major modifications for a "Major Revision" recommendation. If more than 3 experiments are necessary to validate the study conclusions, then you are encouraged to recommend "Reject".

58. Reviewer #1: The methods section might benefit a reorganisation of the sub-sections, splitting it into defined data/sample collection, molecular data analysis, statistical analysis. Also, it is less well written than the introduction and it is difficult to follow at times, e.g. there are redundant sentences.

Addressed in points above.

59. The statistical analyses need to be reviewed, and so the relative results section. See the general comments and the comments in the document attached. The reporting of statistical results is confusing and difficult to follow, I am not sure the methodology fully matches the reported results. The methods and results section regarding statistical testing, although might not be entirely wrong might benefit from a review from an expert in statistics. I think some tests are redundant and the result section might be streamlined with better tables and figures.

We have attempted to address this by improving the clarity of the statistical analysis section, and streamlining the results to follow the same sequence (see above comments).

Additionally, in some instances, the analyses required splitting into “seasonal models” (where a high VIF was observed in the interaction between season and time). These were kept due to the differing patterns observed in the analyses of each season in isolation. Hence just removing the interaction and not acknowledging this would be inappropriate to describe all the ongoing epidemiological processes in this system.

Comments to the pdf have also been addressed individually above.

Reviewer #2: --

Reviewer #3: 1. Taxonomic identification and interpretation of the biology of microbes
60. o 1a. The focus on genus and family levels limits the capacity to draw meaningful conclusions about pathogenicity and the biology of the microbes. For instance, while all members of Sarcocystidae are parasitic, their clinical relevance varies. Some may cause subclinical or negligible effects, particularly in well-adapted hosts.

Due to the methods used, it is not possible to obtain species level taxonomic resolution for the *Bartonella* or Sarcocystidae species present. Consequently, the limited ability to draw

conclusions from the taxonomic resolution has been addressed throughout the discussion, furthermore we have identified future work which would be required to verify the drivers behind these patterns observed in this study. As noted previously, while the 16S approach does not always provide species-level identification, it offers a broad overview of putative pathogens present in the samples. This breadth enables the identification of simultaneously occurring processes and helps to narrow down the range of specific pathogens for targeted investigation in future studies.

61. o 1b. Some statements regarding pathogenicity may require further support. For example, existing literature suggests limited pathogenic effects of Bartonella and Mycoplasma spp. in rodent hosts. If the authors are aware of studies suggesting otherwise, citing them would provide a more balanced view.

Bartonella was described as zoonotic in the manuscript and has been left as such with a reference added to support, likewise for the Sarcocystidae. References for the mycoplasmas, and a caveat have been added. Line 267- 272 now reads as: “These included one OTU of the genus *Bartonella*, a bacterial genus potentially including zoonotic species (e.g. *B. henselae*) (62), three OTUs of *Mycoplasma haemomuris* (combined for analysis), one OTU of *Mycoplasma coccoides* and one OTU of *Mycoplasma penetrans* (known to be zoonotic). These three *Mycoplasma* species have traditionally been associated with pathology in rodents (63-65). While more recent studies suggest that *Haemoplasma* may be largely subclinical in wildlife they have also highlighted the need for further studies increasing their geographic range and elucidating transmission (66).”

62. o 1c. It would be helpful to describe the criteria used to classify OTUs as putative pathogens. At the genus level, groups like Rickettsia and Francisella include both endosymbionts and pathogens, so additional clarification is warranted to avoid overinterpretation.

This has been clarified on lines 169 to 179: “From the clusters of microorganism DNA detected, putative pathogenic OTUs were filtered and selected (51). First, we discarded putative false positive results associated with sequence counts below two OTU-specific thresholds, which checked respectively for putative cross-contamination between samples during DNA extraction and PCR steps on the one hand, and incorrect assignment due to the generation of mixed clusters on the flowcell during Illumina sequencing on the other hand (52). Then, for each sample, and each OTU, we kept only the occurrences confirmed by the two PCR replicates of the same sample. Finally, the putative pathogenic OTUs were selected using their taxonomic affiliations and based on their potential zoonotic risk and pathogen impact on rodents, to separate them from commensal species (51).”

63. 2. Limitations of the 16S rRNA metabarcoding approach
The chosen method may underrepresent certain taxa and has limited resolution for accurately detecting specific pathogens.

The reason for choosing this method has been clarified in response to comments in Part 1 above.

64. 3. Use of statistical analyses, experimental design, and result presentation
65. o 3a. The results section could be streamlined, for example by referring readers directly to Table 3 where appropriate.

The results section has been streamlined by improving formatting and structure (see comment regarding methods/results). Furthermore, the AICc values, and references to table 3 have been added throughout the results section in order to provide more detail on the models and clarity to the reader.

66. o 3b. While the seasonal breakdown of figures (e.g., Figs. 5, 7) reflects the statistical analysis, its relevance to the main research questions could be reconsidered.

Figure 5 (now figure 6 in new document) was kept as it shows the reduction in prevalence with time, while also acknowledging that certain years have only single seasons data. This is part of the main hypotheses. Figure 7 has since been removed

67. o 3c. Given the specific hypotheses about invasion dynamics, the authors may consider targeted model comparisons. For example, comparing the AIC of models with and without the region × year × species interaction Similarly, it may be informative to assess whether species differences are more pronounced in Ireland than in France.

For each GLMM in this present study, the model was run through the package MuMIn, which takes all the variables and interactions fed into the GLMM and tests the models against each other with and without each factor to find the lowest AICc model (which best describe the data). The only interaction included an interaction with season and year due to the 2017 and 2022 data only containing a single season's data.

68. o 3d. To assess a dilution effect, it may be clearer to directly correlate the relative abundance of C. glareolus with pathogen prevalence.

Grouping by region affords more statistical power than using relative abundance, as not all sites within each region contain equal numbers of animals sampled. This would be particularly evident in the invasion gradient analysis which included the 2016 only data, to avoid the influence of time, where due to the single year of sampling each site would exhibit fewer samples. Hence region was correlated with the assigned "percentage *Apodemus*" for each sample to show that region could be used as a proxy in this situation. To further establish whether invasion region was the contributing factor, site was included as a random effect variable. This was to account for differences in pathogen prevalence that may occur between sites in each region, due to climatic, environmental or other variables.

69. o 3e. Since the abundance of C. glareolus varied over time in the invasion front regions, summarizing infection rates across regions (e.g., in Fig. 4) may obscure temporal effects. Similarly, time alone may not serve as a reliable proxy for invasion in regions like the core, where host abundance remained stable (e.g., line 350).

The infection rates across regions and years have now been summarised in Supplementary table 2. Furthermore, this study focuses on the temporal effects, as the invasive host becomes more established on the island, to test whether assumptions made from cross-sectional studies hold true when tested over time. Further work would need to be carried out to investigate the changes in specific regions, such as the core where bank vole relative abundance has stayed relatively stable, or the 2021 invasion front which has become rapidly invaded between the 2016 and 2021 period.

70. o 3f. For the longitudinal data, it might be useful to analyze year-to-year differences as a dependent variable or apply repeated measures analyses.

This might work on more even datasets, however, as the data here is limited by the two years of full data (both seasons; spring and autumn) and two years of one season only (i.e. 2017 only has autumn, while 2022 only has spring). In this instance we decided to go for a more conservative approach, using GLMMs and fitting a year*season interaction, as our primary interest was in the change in infection patterns over time, while accounting for seasonal effects on pathogen prevalence.

71. o 3h. Some of the conclusions, such as the reduction in potentially pathogenic taxa in Irish C. glareolus populations, would benefit from supporting figures. For instance, a comparison of pathogen community composition between Irish and French C. glareolus and Apodemus populations would clarify whether community differences are stronger in the invasive host.

This has been included in Figure 3, on line 306 of the tracked changes document.

72. o 3i. It is unclear why Table 1 presents separate analyses for each host species rather than including host species as a model factor.

The invasive host, *C. glareolus,* in Ireland exhibits a different pathogen community to the native *A. sylvaticus,* which can be seen in Table 2. Including *C. glareolus* in the analyses for *A. sylvaticus and* vice versa would introduce a significant number of zeroes to the analysis. Likewise, the main focus of the analysis is to provide insights into how the presence of *C. glareolus* effects the pathogens in *A. sylvaticus,* and how its own pathogens are affected, hence host species specific models were chosen as the most appropriate models to investigate this.

73. o 3j. Splitting Table 2 by year and region might make patterns more interpretable.

This has been done and included in supplementary table 2, with a reference in the text as follows; "The prevalence for each region per year available in Supplementary Table 2" on line 276-277.

74. o 3k. Where results are non-significant, providing exact p-values would improve transparency.

P values have been added throughout the results section. In instances where only the P-value was given for GLMMs, the full Z and P values have been given, such as for the Sarcocystidae on line 471: "Age class was not statistically significant (Age class: Mature, GLMM, family = Binomial, z = -1;871, p = 0.0613; Age class: Juvenile, GLMM, family = Binomial, z = 0.453, p = 0.65)." and lines 604-606: "Age class did not significantly influence the prevalence of Sarcocystidae in this analysis (Age class: Mature, GLMM, family = Binomial, z = -0.848, p = 0.4; Age class; Juvenile, GLMM, family = Binomial, z = 1.727, p = 0.084)."

Part III – Minor Issues: Editorial and Data Presentation Modifications
Please use this section for editorial suggestions as well as relatively minor modifications of existing data that would enhance clarity.

75. Reviewer #1: The manuscript has great potential and it shows interesting results. More clarity and better presentation of the results are needed to convey the main message that gets a little bit lost. Minor mistakes, and formatting issues are reported in the comments included in the attached document.

Addressed in response to comments from reviewer 1 as described above, results have been restructured to mirror the new statistical analysis section. The summary table 3 is included towards the start of the results section as it provides a key reference point for the reader to come back to when reading the discussion.

76. Reviewer #2: I only have few minor comments:

Page 5, lines 133–135: It is unclear how the sample size was determined. It would be helpful to include the formula or method used for the calculation.

Sample size calculation were clarified and a link to the tool that was used is provided as follows: "The maximum sample size was calculated based on the estimated sample required to observe at least one infected individual based on a prevalence of 12%, and at least 1 success, with 95% confidence intervals (i.e. a sample size of 24 should allow detection of all pathogens with a prevalence of 12% and above, allowing detection of rare pathogens, calculated using Sampsize tool available at: https://sampsize.sourceforge.net/iface/index.html#prev)." on lines 141-146.

77. Discussion:
Both rodent species exhibit wide geographic distributions and overlap spatially in several areas, including regions where they are both native. I suggest adding more background on the known interactions between these species. Is there evidence of competition, displacement, or other ecological interactions? Since the authors analyze the relative abundances of the native species in relation to the presence of the invasive species, and compare pathogen occurrence and prevalence between them, providing additional information on their interactions in other regions would strengthen the discussion.

This has been added to the introduction, referencing previous studies on the Irish rodent system, now reading: "Furthermore, previous studies have shown a reduced relative abundances of *A. sylvaticus* at sites invaded by *C. glareolus* (39, 43)." on lines 97-98.

78. Figures: Figures would be more visually engaging and easier to interpret if presented in color rather than grayscale.

While colour can be essential to understanding some figures, this paper only uses histograms to visualise our findings. In this case grayscale provides a clear contrast for readers with different forms of colour-blindness, making our figures universally accessible. Additionally, it allows for printing without coloured ink or toners. Asterisks have been included to indicate statistical significance where appropriate.

79. Reviewer #3: 1. Defining key terms such as “invasion gradient,” “invasion front,” and “invasion core” early in the text would help orient readers.

These are described in the introduction, highlighting that the core would have the highest relative abundance of *C. glareolus*; “This has allowed for the establishment of cross-sectional studies with an invasion gradient, containing sites from the invasion core (established from the introduction point, where *C. glareolus* exhibits it’s highest relative abundance), invasion front (sites where *C. glareolus* had most recently invaded), and uninvaded sites (*A. sylvaticus* only) (39).” on lines 98-101. Furthermore, it has been clarified in the methods section which sites are located in each of these regions.

80. 2. Line 118: It may be more appropriate to refer to the “amplification effect hypothesis” rather than the “spillback hypothesis.”

In this context we would argue that any increases in pathogen prevalences directly attributable the *C. glareolus* invasion would be considered spillback, as the amplification in the pathogen would be a result of the presence of the non-native host.

81. 3. Consider using a consistent metric for relative abundance throughout (i.e., proportion of C. glareolus vs. percentage of A. Sylvaticus; lines 189 and 219 and Fig. 2).

This reads as “percentage of *A.sylvaticus* relative to *C. glareolus*” in the methods section, now moved to lines 246 – 255. However, the proportion of *C. glareolus* relative to *A. sylvaticus* is used in the figure in order to aid interpretation of how invaded the sites are, as 100% *A. sylvaticus* for the invasion front 2021 would seem more confusing than the levels of *C. glareolus*.

The results section from lines 315 to 324 now mention percentage only and make each paragraph clear its “percentage of *A. sylvaticus* relative to *C. glareolus*” on lines 315-322.

82. 4. Line 237: B. henselae is rarely found in rodents (1–2%), and mainly in rats. Most Bartonella strains are not pathogenic to their rodent hosts.

This was intended to provide an example of a *Bartonella* species that this cluster may relate to, it is further described in the discussion section that further work is needed to identify the species of *Bartonell*a that are in this OTU cluster. Additionally, this present study only claimed that *Bartonella* was zoonotic, rather than pathogenic to rodents. Finally, I have added the below reference to this statement, which shows *A. sylvaticus* has the potential to carry *B. henselae*:

Engbæk, K. and Lawson, P.A. (2004), Identification of *Bartonella* species in rodents, shrews and cats in Denmark: detection of two *B. henselae* variants, one in cats and the other in the long-tailed field mouse[±]. APMIS, 112: 336-341. https://doi.org/10.1111/j.1600-0463.2004.apm1120603.x

83. 5. Table 2: The evidence for M. haemomuris transmission via lice is dated and limited. More recent findings suggest direct host-to-host transmission may be more important.

A caveat acknowledging this has been added to the table 2 legend: “*Mycoplasma* transmission has been linked to ectoparasites such as lice, based on early observations and

PCR detection in arthropod vectors, although more experimental evidence for biological transmission is required (69).”

84. 6. Table 3: Please clarify what the values in parentheses represent—are they levels with the strongest effect (this does not appear to be the case)?

This was described in the footnote under the table. We have further clarified this by adding the term; “Statistically significant factors include the associated variable (level) in brackets.”

85. 7. Lines 260–261: Please clarify whether the authors refer to regional species richness or per-host richness, and consider supporting this with appropriate statistics or a figure.

This was clarified in the statistical analysis section on lines 187-188, now reading: “Pathogen species richness was calculated by counting the total number of putative pathogenic taxa recorded in an individual.”

86. 8. Fig. 3: Including Sarcocystidae here would make the figure more representative of the study’s full scope.

The patterns of infection that were consistent with the enemy release hypothesis were observed mainly in the bacterial species. As the Irish vole populations are quite heavily infected with Sarcocystidae, therefore comparing bacterial communities would be appropriate. Furthermore, Sarcocystidae were filtered differently between the French and Irish datasets making the prevalences incomparable as the French only kept 2 OTU’s, while our data includes four OTU’s. This would make comparing these prevalences inappropriate as they stand. Additionally excluding two OTU’s from the Irish data to make this comparable would also increase confusion for the reader.

87. 9. Line 311: Please specify whether the value 0.15 is a correlation coefficient.

This has been added in on line 530: “Prevalence was positively correlated (correlation coefficient; 0.15) with time (GLMM, family = Binomial, $z = 2.344$, $p = 0.019$; Pearsons’s correlation test, $t = 2.4751$, $p < 0.05$).”

88. 10. Line 341: If the intercept-only model is the best fit, this may suggest no explanatory variable significantly improves the model. Proceeding to additional models should be justified.

The AICc differs between these models by approximately 0.1, Therefore, it is acceptable as the delta AICc is less than 5, likewise age shows some statistical significance, which means there is some importance here regarding pathogen prevalence, even though the post-hoc analysis shows no difference between age groups. Hence, this has been kept, and the AICc values for each added to the results section, now reading: “For the invasion gradient analysis, the best model (lowest AICc value from MuMIn; 285.4) included only the null model, therefore the second lowest AICc model (285.486) was used, including only age class and sex (Table 3).”

89. 11. Lines 463–464: This pattern appears to be shared by A. Sylvaticus as well—clarification would be helpful.
This is explained from lines 632 to 648 prior when discussing rodent-borne pathogens in Ireland.

Manuscript

Click here to access/download;Manuscript;16s OTU analysis-R1 consilidated copy.docx

Title:

Longitudinal study of a rodent invasion reveals shifts in host pathogen interactions.

Short Title:

Longitudinal study of a rodent invasion reveals shifts in host pathogen interactions.

Author Information:

Andrew McManus[1], Maxime Galan[2], Nathalie Charbonnel[2], Celia Holland[3], Vincent Sluydts[4], Annetta Zintl[5], Peter Stuart[1].

[1] Munster Technological University, Department of Biological and Pharmaceutical Sciences, Tralee, Co. Kerry, Ireland.

[2] CBGP, INRAE, CIRAD, Institut Agro, IRD, Univ Montpellier, Montpellier, France.

[3] Trinity College Dublin, the University of Dublin, Department of Zoology, College Green, Dublin 2, Ireland.

[4] University of Antwerp, Department of Biology, Evolutionary Ecology Group, University of Antwerp, 2610 Wilrijk, Belgium.

[5] University College Dublin, School of Veterinary Medicine, Veterinary Science Centre, Belfield, Dublin 4, Ireland.

Corresponding Author:

Peter Stuart; peter.stuart@mtu.ie

// Abstract

Invasive species can disrupt native epidemiological processes, potentially leading to the emergence of zoonotic pathogens. The relatively recent appearance and spread of the bank vole (*Clethrionomys glareolus*) in Ireland provides a unique model system to study these phenomena. The depauperate small mammal community in Ireland, combined with baseline data, allows us to identify the invasion gradient of *C. glareolus* and its effects on rodent-borne pathogens over time. A total of 498 *C. glareolus* and 584 *Apodemus sylvaticus* were sampled, via removal trapping, across nine sites in Ireland in 2016 and 2017, with six sites revisited in 2021 and 2022. 16S rRNA metabarcoding identified 10 putative pathogenic Operational Taxonomic Units (OTUs) present in these rodents, relating to four bacterial taxa, *Bartonella spp., Mycoplasma coccoides, Mycoplasma haemomuris and Mycoplasma penetrans,* and one family of protozoans, Sarcocystidae. Several epidemiological processes were found to be correlated with the *C. glareolus* invasion; firstly, *C. glareolus* in Ireland exhibited patterns consistent with the enemy release hypothesis, compared to native populations in France, and the native *A. sylvaticus* in Ireland. Secondly, a potential dilution effect, with reduced prevalence of *M. haemomuris* in *A. sylvaticus* at the *C. glareolus* invasion core compared to the invasion front, was observed. Finally, *C. glareolus* had increased prevalence of Sarcocystidae at the invasion front when compared to the invasion core, depending on the time and stage of invasion. These findings further our understanding of pathogen dynamics during biological invasions, demonstrating that invaders affect native host-pathogen communities differently as they advance through various stages of establishment.

Author summary:

When animals are introduced to new environments, they can disrupt local ~~disease~~ patterns by changing how pathogens spread among native wildlife. The spread of the bank vole, a non-native rodent in Ireland, offers a valuable model system for studying how such invasions affect disease dynamics over time. In this study, we examined how the arrival of the bank vole is influencing the health of the native wood mouse. We found that in areas where bank voles have been established longer, wood mice carry fewer infections from a common blood parasite. At the same time, bank voles themselves carried fewer types of pathogens overall than wood mice, possibly giving them an advantage in terms of survival and reproduction. Interestingly, we also observed that bank voles were more likely to carry a specific group of parasites, called Sarcocystidae, in areas they had only recently invaded. These patterns suggest that invading species can reshape disease dynamics in complex and

evolving ways. Understanding how these changes unfold over time is important for predicting future disease risks to wildlife and potentially to humans.

Keywords:

Zoonotic pathogens, epidemiology, rodents, enemy release, 16S rRNA metabarcoding, biological invasions

Introduction

The emergence of zoonotic pathogens has seen a sharp increase since the 1940's, with 60% of emerging infectious diseases originating from animals, including the recent outbreaks of SARS-CoV-2, Lassa, Ebola and Mpox viruses (1-7). This increase in the frequency of emerging infectious diseases, which poses a risk to humans, livestock and indigenous wildlife populations, has been primarily attributed to environmental change, caused by landscape change, a reduction of habitat diversity and the introduction of invasive species (8-16).

A recent review by Bezerra-Santos *et al.* (15), highlights that invasive mammal species pose increased risks for public health by introducing zoonotic pathogens (17, 18). However, non-native species can also benefit from a reduction in natural enemies, such as predators or pathogens, compared to conspecifics in their native range (and often compared to the native species), through a process known as "enemy release"(19, 20). In relation to pathogens, this can happen because the pathogen, or the vector responsible for its transmission, is absent from the founder population, or because the environment in the new range is unsuitable to sustain the pathogen or vector (21-25). This, in turn, may give invaders a competitive advantage over native species. Additionally, invasive species can influence host-pathogen ecological interactions in native hosts in several other ways. Firstly, invasive species can co-invade with new pathogens, which may infect native hosts, leading to a process known as pathogen spillover (26). The establishment of invasive species may also lead to a dilution of native pathogens, where the presence of a host with a lower competence for a specific pathogen can reduce the circulation of that pathogen in competent hosts (27-29). On the other hand, invasive host species can also have an increased competency for native pathogens, causing a spillback of native pathogens that amplifies infection in native hosts (30, 31).

Rodents, the largest order of mammals, make up one of the most common group of invasive species worldwide, affecting native ecosystems, food production and human health (32). Additionally, a recent review by Dharmarajan *et al.* (6), highlighted that rodents and bats harbour the highest proportion of zoonotic agents, identifying these taxa as species of interest for studying zoonoses. Therefore, rodents make excellent models for studying the eco-epidemiology of pathogens due to their presence in large numbers and broad distributions, playing an important role as reservoirs for pathogens causing zoonotic disease; such as, orthohantaviruses, *Leptospira* and *Bartonella* species, or *Toxoplasma gondii* (6, 33-37). Finally, studies on rodent invasions have demonstrated the interplay between rodent pathogen interactions and biological invasions, for example, helminth studies in both Ireland and Senegal have observed patterns of enemy release in invasive rodents when comparing sites of long established populations to more recently invaded sites (24, 31, 38, 39).

The case of the bank vole, *Clethrionomys glareolus* (formerly *Myodes glareolus*), invasion in Ireland presents a unique opportunity to explore changes in epidemiological dynamics during a biological invasion (37), particularly due to the depauperate woodland rodent community in Ireland, that includes only the invasive *C. glareolus* and the native woodmouse, *Apodemus sylvaticus* (40). Species depauperate systems provide a valuable scenario to tease out the effects of invasive species on native pathogen communities. For example, in this instance the depauperate rodent community in Ireland has allowed for easier identification of the effects the *C. glareolus* invasion has on the native *A. sylvaticus* population in previous studies (31, 39). Furthermore, *C. glareolus* in Ireland has a single identifiable introduction point in the south west, near Foynes, Co. Limerick, and the likely source population from Germany identified through mitochondrial DNA (41). In addition, studies have shown *C. glareolus* in Ireland to have an expansion rate of 2.23 – 2.63 km per year in unconstrained areas (42). Furthermore, previous studies have shown a reduced relative abundances of *A. sylvaticus* at sites invaded by *C. glareolus* (39, 43). This has allowed for the establishment of cross-sectional studies with an invasion gradient, containing sites from the invasion core (established from the introduction point, where *C. glareolus* exhibits it's highest relative abundance), invasion front (sites where *C. glareolus* had most recently invaded), and uninvaded sites (*A. sylvaticus* only) (39). These factors combined, provide an excellent case study to identify the epidemiological effects that the *C. glareolus* invasion has on native pathogen communities in *A. sylvaticus*, such as spillover, dilution, or spillback.

The development of high-throughput sequencing, and in particular metabarcoding techniques, facilitates a more holistic approach to studying the rodent pathobiome (44). These methodologies provide a global picture of pathogen diversity within host species, improving our understanding of the relationship between host-parasite

interactions and biological invasions. In the Irish context, rodent helminth parasite communities have been explored previously by Loxton *et al.* (23, 31) and Stuart *et al.* (39). However, no such data exists on pathogenic microparasites in this model system. Furthermore, there remains a knowledge gap concerning longitudinal studies focusing on changes in pathogen communities during biological invasions. Such studies are essential for fully exploring changes in host-pathogen interactions, such as pathogen loss in invasive species, as cross-sectional studies only capture a single timepoint of this dynamic process (37, 39, 45-47).

In this study, we aim to address these knowledge gaps by describing the community of microparasites that could be zoonotic or pathogenic for rodents, including bacteria and protozoa, of *C. glareolus* and *A. sylvaticus* in Ireland, through a longitudinal study. We performed a spatiotemporal analysis of this community along the invasion gradient described above, using rodents removed in 2016 from a previous study by Stuart *et al.* (39), with subsequent removal sampling at these sites in 2017, 2021 and 2022. In this study, we use the 16s rRNA amplicon metabarcoding approach developed by Galan *et al.* (48), and recently used by Bouilloud *et al.* (49). Focusing on putative pathogenic taxa recorded in samples collected between 2016 and 2022, this present study addresses three main hypotheses: (i) whether *C. glareolus* exhibits patterns consistent with the enemy release hypothesis, including a lower pathogen species richness of potentially pathogenic taxa when compared to its native range and the native *A. sylvaticus;* (ii) whether the presence of *C. glareolus* alters the pathogen communities present in the native *A. sylvaticus* as expected under the dilution or spillback hypotheses; finally (iii) whether pathogen prevalence changes over time in both rodent species, as the invader becomes established.

Materials & methods

Field work & sample collection

Nine sites were selected for field sampling in 2016, as described in Stuart *et al.* (39). These were divied into three regions based on their invasion status; including three invasion 'core' sites in Foynes (Foynes 1, 2 & 3), three 2016 invasion front sites (named 'front 2016' herein, i.e. Moore Abbey, Carnpark, and St. John's), and three 2021 invasion front sites (named 'front 2021' herein, i.e. Killinthomas, Loughkey and Donore, uninvaded in 2016) (Figure 1). In 2016, rodents were sampled at all of these nine sites in spring and autumn trapping sessions and revisited in autumn 2017. For the longitudinal aspect of this study, only six of these sites were revisited in spring and autumn 2021, and spring 2022 (Supplementary table 1). These sites included two of the

‘core’ sites in Foynes (Foynes 1 and 3, not Foynes 2), two invasion ‘front 2016’ sites (Moore Abbey and St. John’s wood, not Carnpark) and two invasion “front 2021” sites (Killinthomas and Donore, not Loughkey) (Figure 1).

Longworth traps, baited using peanuts, bird seed mix and a piece of carrot, were placed 5-10m apart in transects of a maximum of 20 traps. A maximum of 24 animals from each species were removed from each site per season and year of sampling. The maximum sample size was calculated based on the estimated sample required to observe at least one infected individual based on a prevalence of 12%, and at least 1 success, with 95% confidence intervals (*i.e.* a sample size of 24 should allow detection of all pathogens with a prevalence of 12% and above, allowing detection of rare pathogens, calculated using Sampsize tool available at: https://sampsize.sourceforge.net/iface/index.html#prev). Traps were placed in the field overnight and checked early the next morning. Rodents were inspected in the field and transported back to the laboratory. If insufficient numbers were trapped, the site was revisited for an additional night within the same season.

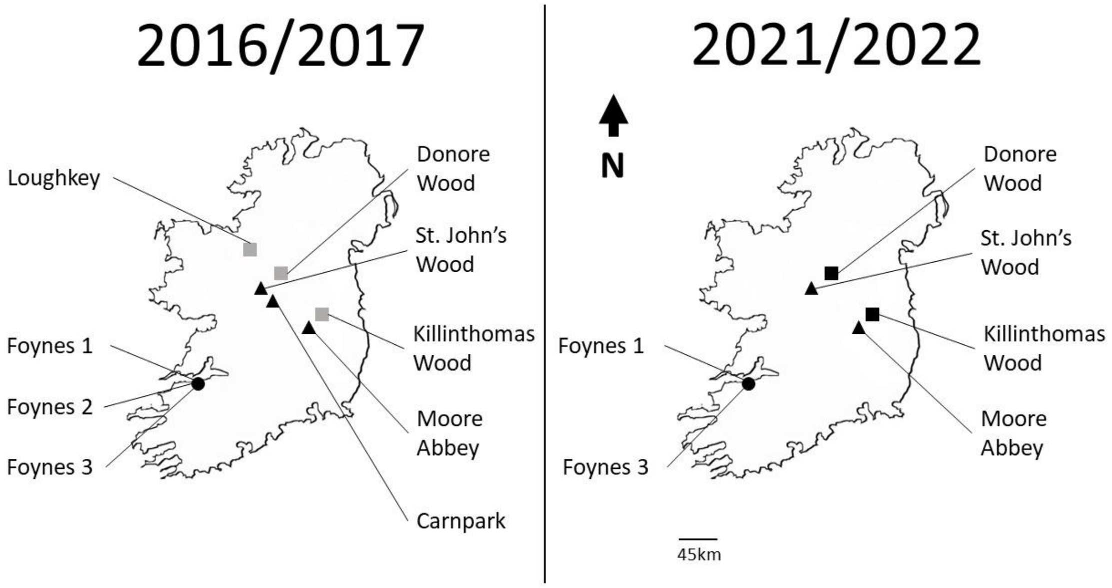

Figure 1. Map of Ireland, showing sampling sites visited in 2016, 2017, 2021 and 2022. Circles indicate the invasion core; triangles indicate the invasion front in 2016 (‘front 2016’) and squares indicate the invasion front in 2021 (‘front 2021’). The grey colour shows sites that were uninvaded by *Clethrionomys glareolus* on a given date, while the black colour shows sites that were invaded.

Laboratory work & analysis

A total of 498 *C. glareolus* and 584 *A. sylvaticus* were sampled across the four years studied. Rodents were euthanised using Isoflurane (1000mg/g, under license from the Health Products Regulatory Authority). The spleens were removed immediately and stored in RNAlater at 4°C for 24 hours, then placed into the freezer at -20°C for further metabarcoding analysis. Rodents were classified into three age categories, juvenile, young adult and mature adult, as described in Behnke *et al.* (50). These were based on the criteria of rodent length (nose to anus; in millimetres) and weight (grams) as described by Loxton *et al.* (23, 31) and used by Stuart *et al.* (39). For animals found to be at the limit of each classification, visual assessments of maturity were used for final determination, as described by Stuart *et al.* (39).

The 16S metabarcoding analysis was conducted as described by Galan *et al.* (48). DNA was extracted from the rodent spleens using DNeasy 96 Tissue Kit (Qiagen, Germany). This organ was selected by Galan *et al.* (48), as the spleen is responsible for detecting and removing blood-borne microrganisms. Extracted DNA was screened for putative pathogenic Operational Taxonomic Units (OTUs) using the v4 hyper variable region of the 16S rRNA gene with universal primers (16S-V4F 5'-GTGCCAGCMGCCGCGGTAA-3' and 16S-V4R 5'-GGACTACHVGGGTWTCTAATCC-3'), via Illumina MiSeq sequencing. Finally, PCR amplification, sequencing and taxonomic identification were carried out as described by Galan *et al.* (48). From the clusters of microorganism DNA detected, putative pathogenic OTUs were filtered and selected (51). First, we discarded putative false positive results associated with sequence counts below two OTU-specific thresholds, which checked respectively for putative cross-contamination between samples during DNA extraction and PCR steps on the one hand, and incorrect assignment due to the generation of mixed clusters on the flowcell during Illumina sequencing on the other hand (52). Then, for each sample, and each OTU, we kept only the occurrences confirmed by the two PCR replicates of the same sample. Finally, the putative pathogenic OTUs were selected using their taxonomic affiliations and based on their potential zoonotic risk and pathogen impact on rodents, to separate them from commensal species (51).

Statistical analyses

Calculation of prevalence and species richness

Data were analysed using the R version 4.3.1 working under the RStudio software hoods (2023.06.1 Build 524). The packages Plyr (53) and ggplot2 (54) were used to create tables and figures. Separate putative pathogenic

OTUs of the same pathogen species were combined into potentially pathogenic taxa for analysis. For each putative pathogenic taxa, prevalence was calculated by the total number of infected individuals divided by the total population sampled. Confidence intervals (maximum and minimum) were calculated to 95% using the R package PropCIs (55). Pathogen species richness was calculated by counting the total number of putative pathogenic taxa recorded in an individual.

Examining patterns of the enemy release hypothesis

Firstly, bacterial data collected from *C. glareolus* and *A. sylvaticus* in Ireland from 2021 was compared to data collected from rodents trapped in forest sites in France, sampled in 2021 by Pradel *et al.* (56), using the same 16S metabarcoding approach (48). These data were examined by comparing prevalences of potentially pathogenic bacterial taxa in rodents from Ireland and France, to examine if less species were detected and at a lower prevalence in Ireland, related to the enemy release hypothesis. However, due to differences in assigning age and maturity to animals in each country, statistical analysis could not be conducted. Likewise, while the bacterial pathogens were filtered using the same methodology, two extra species of potentially pathogenic Sarcocystidae were included in the Irish data, and consequently meant that these pathogen species could not be included in the comparison.

Examining patterns of infection in Irish rodents

To investigate patterns of infection in each host species in Ireland, pathogens with prevalence above 5% in the overall dataset (all sampling dates and sites) were selected for statistical analysis using generalised linear mixed models (GLMM's) and a binomial distribution (24). The threshold of 5% prevalence was selected as variations below this threshold would be too low for appropriate statistical testing. GLMM's from the package glmmTMB were used (57). GLMMs were selected for these analyses to allow the inclusion of both fixed and random effects in the models, and the appropriate distribution for each type of data being analysed. Due to the depauperate pathogen communities in Ireland, zero-inflated negative binomial distributions were used to investigate patterns in pathogen species richness instead of Poisson distributions used by Diagne *et al.* (24).

Two strategies were used to analyse these data;

1. **The invasion gradient analysis**: only data from 2016 (and all nine sites) were included to test the influence of the invasion gradient on infection patterns in both *A. sylvaticus* and *C. glareolus*, as this year had a very clear "core", "front" (front 2016) and "uninvaded" (front 2021) sites as per the study by Stuart *et al.* (39) (see Figure 1). An additional model examining the difference in pathogen species richness between both rodent species in Ireland, and a separate model focusing on *A. sylvaticus* only, was conducted using these data from 2016.

2. **The longitudinal analysis:** data from all four years were used, but only the six sites that were sampled each year were selected, excluding Foynes 2, Carnpark and Loughkey, to keep the data comparable (Table 1). In this approach, time was included in the models as a numerical variable counting years from baseline time, i.e. 2016 = 1.

Factors used in each model included season, region (invasion region; core, front 2016, front 2021), sex, age class (juvenile, young adult and mature) as fixed effects, with site included as a random effect variable (Table 1). The longitudinal analysis included the additional fixed effects of time, as years since baseline (2016), and an interaction between season and time. An additional fixed factor, rodent species, was used for the model investigating the variance in pathogen species richness between Irish populations of *C. glareolus* and *A. sylvaticus* in 2016. The MuMIn package was used to refine and find the best model based on lowest Akaike information criterion corrected for small sample size (AICc) value (59). The DHARMa package was then used to check model fit and the performance package to test for multicollinearity and overdispersion (60, 61). The optimisation function, Optim, using the Broyden, Fletcher, Goldfarb and Shanno (BFGS) method was used in cases where model optimisation was needed. Post-hoc tests using Tukey Kramer analysis were used for factors with three or more modalities, while post-hoc Pearson's correlation tests were used to analyse changes in prevalence with time. In cases where no GLMM could be fitted, non-parametric tests were used, such as a Wilcoxon Rank Sum test.

These analyses used the categorical variable of region as a proxy for invasion levels of *C. glareolus* relative to *A. sylvaticus* to aid in the interpretation of the results and increase statistical power through increased number of samples per variable. In order to justify our classification of invasion region in the analysis, the percentage of *A. sylvaticus* relative to *C. glareolus* was tested against invasion region. For these analyses, the total number *A. sylvaticus* relative to *C. glareolus* trapped in each site in each season within a given year was calculated, generating a percentage of *A. sylvaticus* relative to *C. glareolus.* These numbers were then assigned to each sample corresponding to their trapping site, season and year. Finally, to justify the classification of regions, this assigned percentage of *A. sylvaticus* relative to *C. glareolus* in the dataset was tested against region using a

Kruskal-Wallis test, and a post-hoc Dunn's test, through the dunn.test R package (58), to check differences between regions in both 2016 only, and the longitudinal dataset, as the data followed a non-normal distribution.

Table 1. Models and data used for the two approaches of this study, the invasion gradient analysis and the longitudinal analysis. Time:Season indicates the interaction that was used between time and season. Time is defined as years since 2016 baseline, with 2016 = 1.

	**Invasion gradient analysis**	**Longitudinal analysis**
**Fixed variables**	Season, Region, Age Class, Sex	Time, Season, Region, Age Class, Sex, Time:Season
**Random variables**	Site	Site
**Years**	2016 only	All (2016, 2017, 2021, 2022)
**Sites**	All (Figure 1.)	6 repeated sites; Foynes 1 & 3, Killinthomas, Donore, Moore Abbey and St. John's.

Results

Pathogens detected in Irish rodent spleen samples

The 16S analyses of 584 *A. sylvaticus* and 498 *C. glareolus* trapped over the four years of sampling in Ireland revealed 10 putative pathogenic OTUs corresponding to five potentially pathogenic taxa (Table 3; Sequencing information, raw sequence reads and prevalence tables are available to the following link: https://zenodo.org/records/14883529). These included one OTU of the genus *Bartonella*, a bacterial genus potentially including zoonotic species (e.g. *B. henselae*) (62), three OTUs of *Mycoplasma haemomuris* (combined for analysis), one OTU of *Mycoplasma coccoides* and one OTU of *Mycoplasma penetrans* (known to be zoonotic). These three *Mycoplasma* species have traditionally been associated with pathology in rodents (63-65). While more recent studies suggest that *Haemoplasma* may be largely subclinical in wildlife they have also highlighted the need for further studies increasing their geographic range and elucidating transmission (66). Finally, we detected four OTUs of Sarcocystidae (combined for analysis, a family of protozoans, with some

species that are zoonotic, and others that are pathogenic for rodents) (67, 68). All five taxa were detected in *C. glareolus*, all of which had a 1% prevalence or below, except for Sarcocystidae*,* which had a prevalence of 37.15%. All potentially pathogenic taxa were also found in *A. sylvaticus* (Table 2; the prevalence for each region per year available in Supplementary Table 2), except *M. penetrans*. However, it should be noted that only one individual *C. glareolus* was found to be positive for this pathogen out of 498 tested.

Table 2. Prevalence (%) of potentially pathogenic taxa in all *Apodemus sylvaticus* and *Clethrionomys glareolus* sampled in Ireland from 2016 - 2022. The 95% confidence interval (maximum and minimum value) is detailed in brackets.

	**Transmission**	***Apodemus sylvaticus***	***Clethrionomys glareolus***
**Pathogen**		***n = 584***	***n = 498***
Bartonella	Vector (flea)	51.54 (47.40 - 55.66)	0.2 (0.01 - 1.11)
Mycoplasma haemomuris	Vector (lice)*	44.01 (39.93 - 48.14)	1 (0.33 - 2.33)
M. coccoides	Vector (lice)*	44.18 (40.10 - 48.31)	0.2 (0.01 - 1.11)
M. penetrans	Sexual	0.00	0.2 (0.01 - 1.11)
Sarcocystidae	Dependent on species, *i.e.* Environmental (Direct life cycle), or intermediate host (indirect life cycle) in some cases.	1.71 (0.82 - 3.13)	37.15 (32.89 - 41.56)

**Mycoplasma* transmission has been linked to ectoparasites such as lice, based on early observations and PCR detection in arthropod vectors, although more experimental evidence for biological transmission is required (69).

Table 3. Summary of significant factors in pathogen species richness and pathogens prevalence detected.

	**Statistically significant factors observed in invasion gradient analysis (2016)**	**Statistically significant factors observed in longitudinal analysis (2016 - 2022)**
**Both Rodent Species**		
**Species Richness**	Species (Voles)***	NA
Apodemus sylvaticus	**n = 203**	**n = 500**
**Species Richness**	Sex (M)*	NA
Bartonella	Season (Spring)**, Sex (M)*	**Full:** Season(Spring)***, Sex(M)*<br>**Sp:** Time*
Mycoplasma haemomuris	Age class (Mature)***	Season(Spring)**, Region(Front 2016)**, Region(Front2021)**, Age class(Juvenile)***, Age class (Mature)*
Mycoplasma coccoides	Age class (Mature)*	Time*, Age class(Mature)**
Clethrionomys glareolus	**n = 148**	**n = 411**
**Sarcocystidae**	Region (Front 2016)**	**Full:** Time***, Season(Spring)***, Region(Front 2021)*, Sex(M)*, Age class(Mature)*<br>**Sp**: Region(Front 2016)*, Region(Front 2021)***, Age class(Mature)*<br>**Au:** Time***, Sex(M)*

Full = full model including all years and seasons. In some instances, per season models were ran due to multicollinearity, this is indicated by Sp = spring only model, Au = autumn only model. Time indicates years as a numerical value since baseline, with 2016 = 1. n represents sample sizes in the full model for each analysis. Statistically significant factors include the associated variable (level) in brackets. Asterisks indicate level of significance, with * = $p < 0.5$, ** = $p < 0.01$, and *** = $p < 0.001$.

Patterns of infection relating to the enemy release hypothesis

Based on data from 2021, *C. glareolus* in Ireland (n = 189) exhibited fewer potentially pathogenic bacterial taxa than *C. glareolus* sampled in forest sites in France (n=221). Most notably, *Anaplasma, Borrelia, Candidatus Neoehrlichia, Francisella and Orientia* were absent from the Irish samples. In addition, lower prevalences of *Bartonella* and *M. haemomuris* were also observed in the Irish *C. glareolus* specimens (Figure 2a). Moreover, *A. sylvaticus* individuals trapped in Ireland (*n* = 193) had a similar prevalence of *Bartonella* compared to forest site populations in France in 2021 (*n* = 95), but higher prevalences of *M. haemomuris and M. coccoides*. As above,

Anaplasma, Borrelia, Candidatus Neoehrlichia, Francisella and Orientia were not detected in the Irish *A. sylvaticus* samples (Figure 2b).

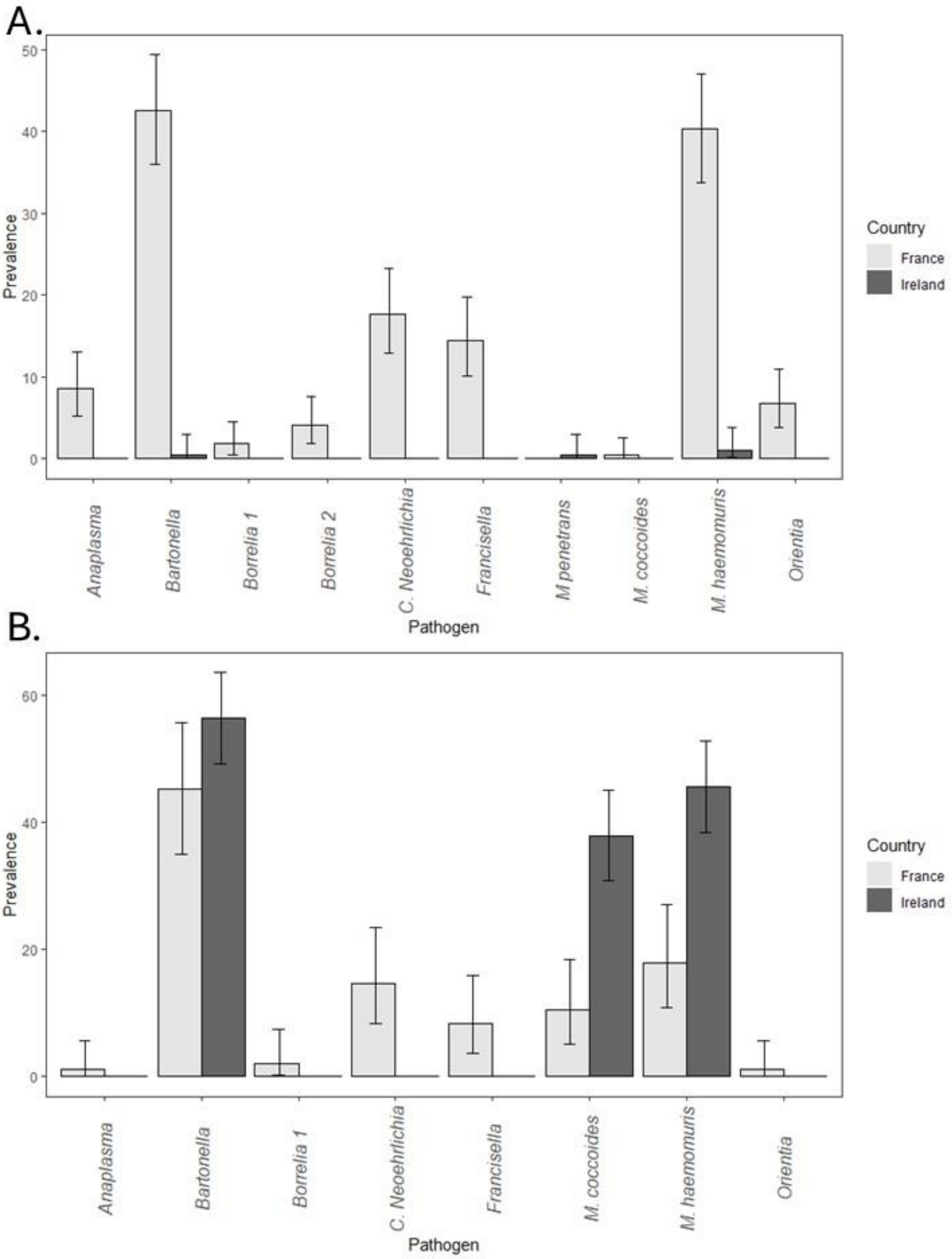

Figure 2. Prevalence of potentially pathogenic bacterial taxa observed in 2021 in both France (*Clethrionomys glareolus* native range, light grey) and Ireland (*C. glareolus* invasive range, dark grey) in **A.** *C. glareolus* and **B.**

Apodemus sylvaticus. Error bars represent 95% confidence intervals. *C. Neoehrlichia* represents *Candidatus Neoehrlichia.*

Validation of invasion region criteria for statistical models & progress of *Clethrionomys glareolus* invasion

In the 2016 data for the invasion gradient analysis, the percentage of *A. sylvaticus* relative to *C. glareolus* was directly correlated with the region of invasion. The core had the lowest percentage of *A. sylvaticus* relative to *C. glareolus,* followed by front 2016, and finally front 2021 was completely uninvaded, with 100% *A. sylvaticus* (Kruskal Wallis, $chi^2 = 271.99$, df = 2, $p < 0.001$; Dunn's test post-hoc analysis, All regions, $p < 0.001$) (Figure 3). Our capture data thus confirms the invasion categories initially designed.

In the longitudinal data, the core had a lower percentage of *A. sylvaticus* relative to *C. glareolus* when compared to both invasion front regions, front 2016 and front 2021. However, the front 2021 was rapidly invaded in 2021 and 2022 and consequently no difference was observed between the percentage of *A. sylvaticus* in front 2016 and front 2021 (Kruskal Wallis, $chi^2 = 606.57$, df = 2, $p < 0.001$; Dunn's test post-hoc analysis, Core – Front 2016, $p < 0.001$; Core – Front 2021, $p < 0.001$; Front 2016 – Front 2021, $p = 0.1039$) (Figure 3).

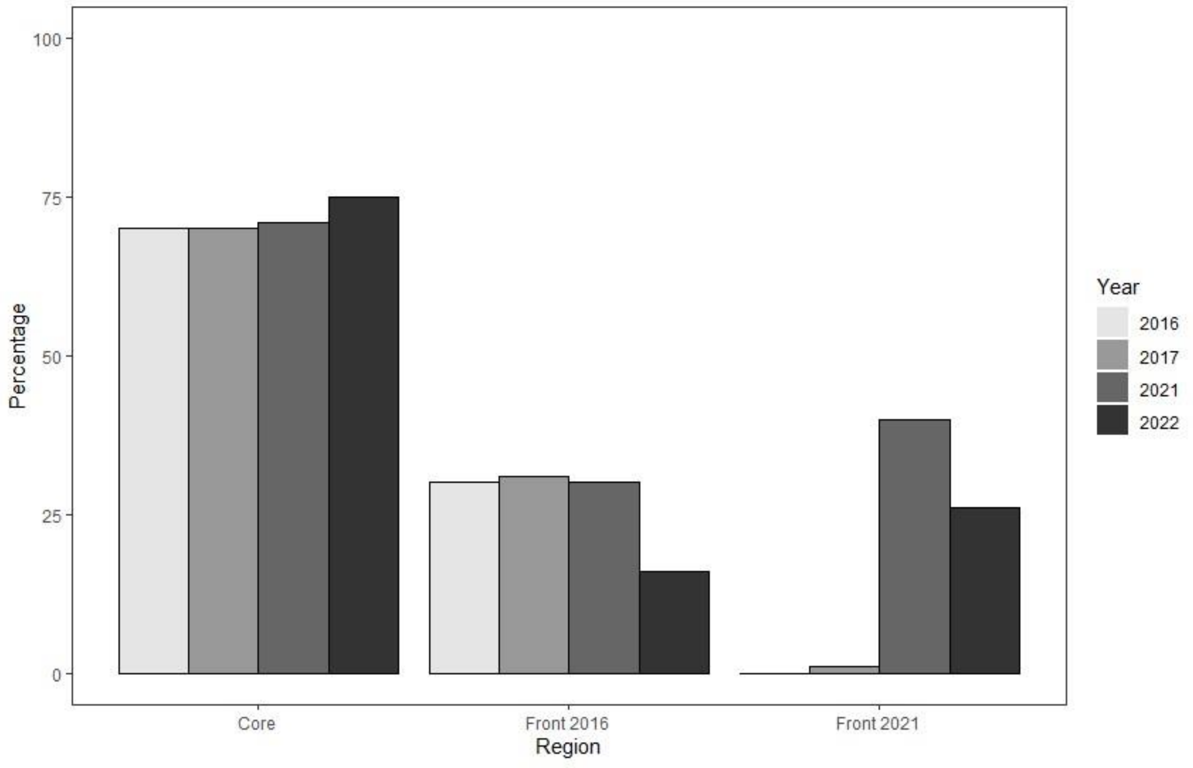

Figure 3. Percentage *Clethrionomys glareolus* relative to *Apodemus sylvaticus* in each region for each year. Data for 2016 and 2017 includes all 9 sites.

Invasion gradient analysis:

Pathogen richness

The best model (lowest AICc value; 813.979) explaining variations in the species richness of potentially pathogenic taxa between *C. glareolus* and *A. sylvaticus* in 2016 included only rodent species (Table 3). *C. glareolus* ($\bar{x}$ = 0.46) was shown to have a lower mean number of pathogen species compared to *A. sylvaticus* ($\bar{x}$ = 1.37) (GLMM, family = Zero inflated negative binomial, $z = -8.068$, $p < 0.001$). However, poor model fit was still observed when examining residuals, hence nonparametric tests were carried out to confirm these findings. Using a Wilcoxon Rank Sum test, a significant difference in species richness between *C. glareolus* and *A. sylvaticus* was confirmed, with the invasive *C. glareolus* (med = 0) having a lower median number of pathogens compared to *A. sylvaticus* (med = 1) ($W = 23262$, $p < 0.001$).

When focusing on *A.* sylvaticus only, using the invasion gradient analysis, no effect for the *C. glareolus* invasion was observed on the species richness of potentially pathogenic taxa. The best model (lowest AICc value; 565.089) included only sex (Table 3). Sex was shown to be statistically significant with males ($\bar{x} = 1.54$) having a higher mean pathogen species richness compared to females ($\bar{x} = 1.14$) (GLMM, family = Zero inflated negative binomial, $z = 2.391$, $p = 0.017$). As before, due to poor model fit, these findings were confirmed using a Wilcoxon Rank Sum test, showing males (med = 2) have a higher median species richness compared to females (med = 1) ($W = 3830.5$, $p < 0.01$).

Apodemus sylvaticus

The patterns of infections observed in the invasion gradient analyses for the pathogen species *Bartonella, M. haemomuris* and *M. coccoides* were examined in *A. sylvaticus* sampled in Ireland.

Bartonella

Considering the invasion gradient analysis, the best model (lowest AICc value; 271.876) included season and sex (Table 3). *Bartonella* prevalences were higher in autumn compared to spring (GLMM, family = Binomial, $z = -2.741$, $p = 0.00612$), while males were more infected than females (GLMM, family = Binomial, $z = 2.404$, $p < 0.05$).

Mycoplasma haemomuris

Considering the invasion gradient analysis, the best model (lowest AICc value; 268.721) included only age class (Table 3). No effect for region was observed. Age class was statistically significant (Age class: Mature, GLMM, family = Binomial, $z = 3.405$, $p < 0.001$). Post-hoc analysis showed that mature adults had significantly higher prevalences compared to young adults and juveniles. No difference between young adults and juveniles was observed, possibly due to the low number of juveniles in the dataset (n = 9) (Tukey Kramer analysis; Mature – Young adults, $p < 0.01$; Mature – Juveniles, $p < 0.05$; Young adults – Juveniles, $p = 0.062$).

Mycoplasma coccoides

For the invasion gradient analysis, the best model (lowest AICc value from MuMIn; 285.4) included only the null model, therefore the second lowest AICc model (285.486) was used, including only age class and sex (Table 3). Age class was the only statistically significant variable (Age class: Mature, GLMM, family = Binomial, $z = -2.115$, $p < 0.05$). However, during post-hoc analysis no statistical differences was observed between pairs of age class (Tukey Kramer analysis; Mature – Young adults, $p = 0.24$; Mature – Juveniles, $p = 0.92$; Young adults – Juveniles, $p = 0.89$). No effect of the *C. glareolus* invasion gradient on *M. coccoides* prevalence was observed, as region was excluded from the lowest AICc model.

Clethrionomys glareolus

Regarding *C. glareolus* in Ireland*,* the patterns of infection with Sarcocystidae observed during the invasion gradient were examined.

Sarcocystidae

Considering the invasion gradient analysis on the prevalence of Sarcocystidae in *C. glareolus,* the best model (lowest AICc value; 202.816) included only region (Table 3). The invasion front (front 2016) showed a significantly higher prevalence when compared to the core (Region: Front 2016, GLMM, family = Binomial, $z = 2.652$, $p < 0.01$) (Figure 4). Similarly, the same invasion gradient analysis was performed on a single OTU of Sarcocystidae specific to *C. glareolus*. The best model (lowest AICc value; 201.422) included region and age class. Region showed the same pattern as the analysis on all Sarcocystidae, with higher prevalence of this Sarcocystidae OTU in the front 2016 compared to the core (Region: Front 2016, GLMM, family = Binomial, $z = 2.187$, $p < 0.05$). Age class was not statistically significant (Age class: Mature, GLMM, family = Binomial, z = -1;871, $p = 0.0613$; Age class: Juvenile, GLMM, family = Binomial, z = 0.453, $p = 0.65$).

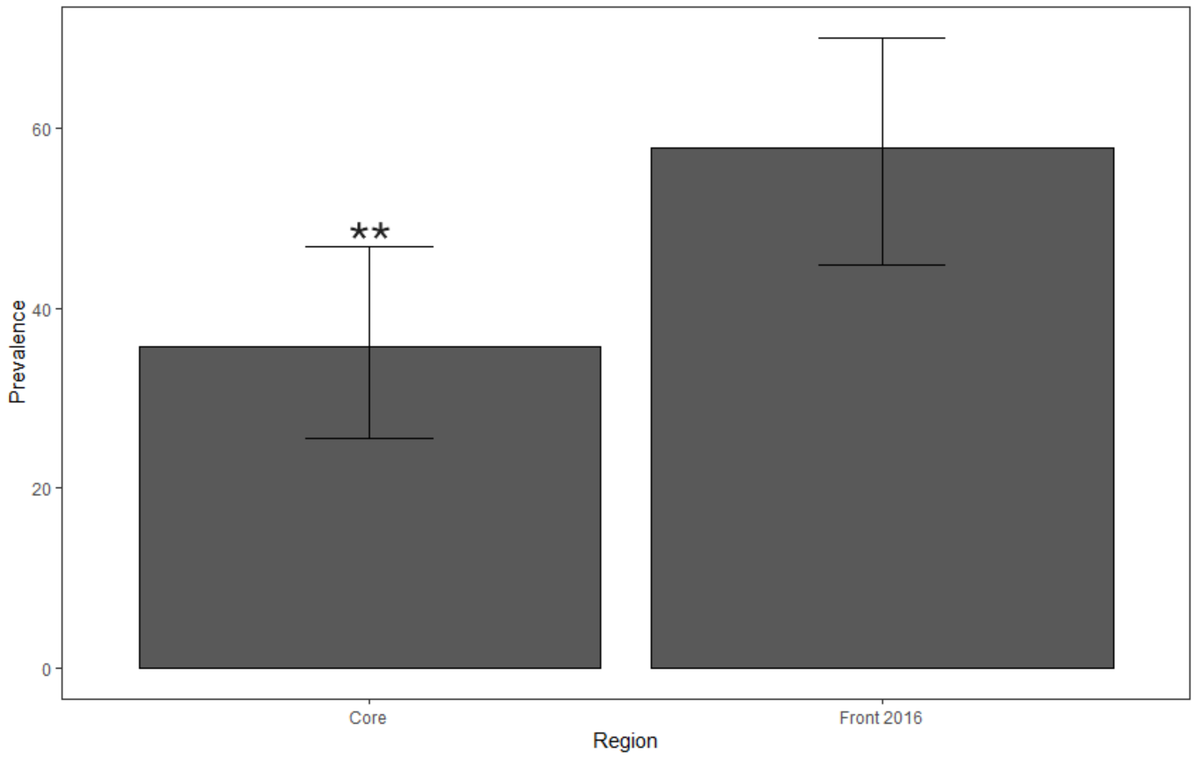

Figure 4. Prevalence of Sarcocystidae in *Clethrionomys glareolus* per region in 2016. Error bars represent 95% confidence intervals. Asterisks indicate level of statistical significant (** = p <0.01)

Longitudinal analyses

Apodemus sylvaticus

The patterns of infections observed in the invasion gradient analyses for the pathogen species *Bartonella, M. haemomuris* and *M. coccoides* were examined in *A. sylvaticus* sampled in Ireland.

Bartonella

In the longitudinal analysis, the best model (lowest AICc value; 672.6) included time, season, region, and sex, with an interaction between time and season (Table 3). The interaction between time and season was shown to have a high variance inflation factor (VIF), meaning the covariates were correlated and potentially affecting the model through multicollinearity. To account for this, the interaction was removed in the overall model (AICc 674.665), and separate GLMM's were conducted for each season. In the overall model, no effect for time or

region was observed (GLMM, family = Binomial, z = 1.904, $p = 0.057$). *A. sylvaticus* in autumn showed higher prevalences of *Bartonella* than in spring (GLMM, family = Binomial, $z = -3.708$, $p < 0.001$). Alongside this, males exhibited higher prevalences compared to females (GLMM, family = Binomial, $z = 2.241$, $p < 0.05$).

In the seasonal models, the best model (lowest AICc value; 354.832) for spring included time and sex. No effect for region was observed (Table 3). Prevalence was positively correlated (correlation coefficient; 0.15) with time (GLMM, family = Binomial, $z = 2.344$, $p = 0.019$; Pearsons's correlation test, $t = 2.4751$, $p < 0.05$). Sex did not significantly influence the prevalence of *Bartonella* in *A. sylvaticus* in the spring only model (GLMM, family = Binomial, z = 1.942, $p = 0.052$). In the autumn only analysis, the best model included only region (lowest AICc value; 322.788), however, no factor significantly influenced *Bartonella* prevalence.

Mycoplasma haemomuris

In the longitudinal analysis, no effect for time was observed, and the best model selected (lowest AICc value; 626.339) included the factors season, region, and age class (Table 3). Region was shown to have a significant effect on the prevalence of *M. haemomuris* (Region: Front 2016, GLMM, family = Binomial, $z = 2.744$, $p < 0.01$; Region: Front 2021, GLMM, family = Binomial, $z = 2.874$, $p < 0.01$). Post-hoc analysis demonstrated that the core had a lower prevalence of *M. haemomuris* compared to front 2016 and front 2021, with no difference observed between front 2016 and front 2021 (Figure 5) (Tukey Kramer analysis; Core – Front 2016, $p < 0.001$; Core – Front 2021, $p < 0.001$; Front 2016 – Front 2021, $p = 0.96$). Secondly, spring had higher prevalences compared to autumn (GLMM, family = Binomial, $z = 2.987$, $p < 0.01$). Finally, age class was shown to be a statistically significant factor (Age class: Juvenile, GLMM, family = Binomial, $z = -3.414$, $p < 0.001$; Age class: Mature, GLMM, family = Binomial, $z = 2.146$, $p < 0.05$). Post-hoc analysis showed mature adults were the most infected, followed by subadults, with juveniles least infected (Tukey Kramer analysis; Mature - Young adults, $p < 0.01$; Mature – Juveniles, $p < 0.001$; Juveniles – Young adults, $p < 0.001$).

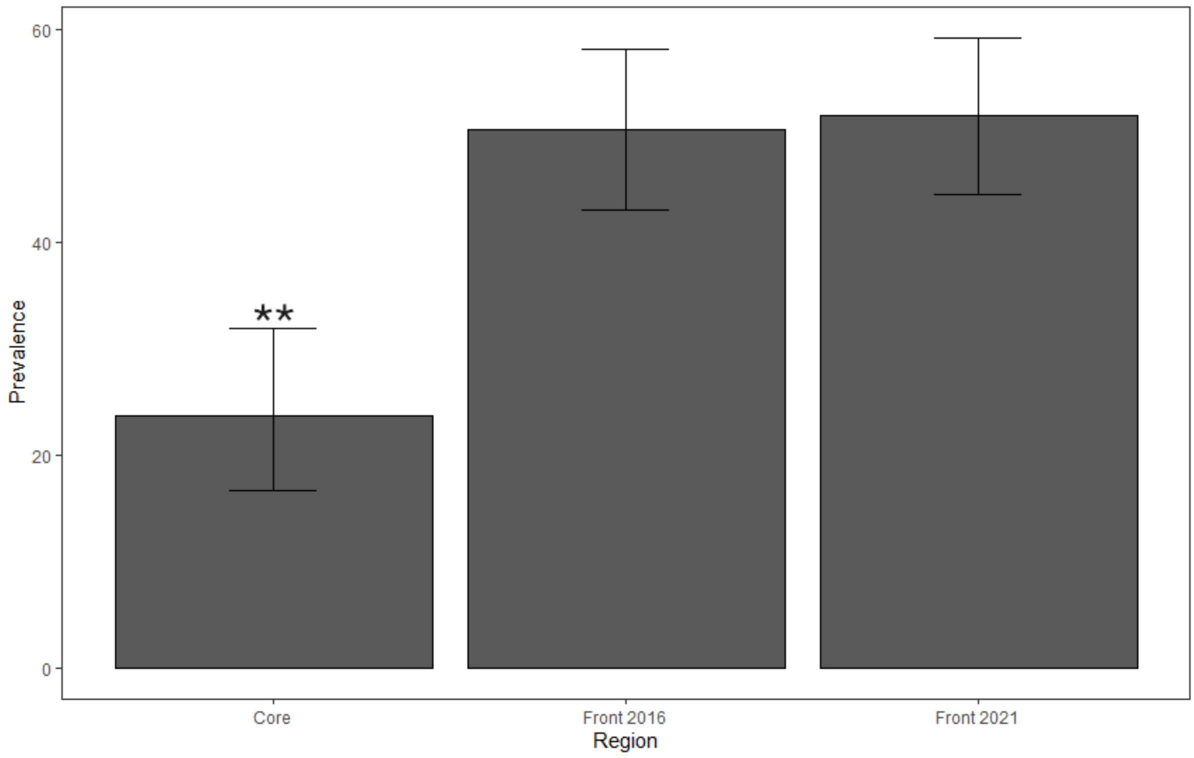

Figure 5. Prevalence of *Mycoplasma haemomuris* in *Apodemus sylvaticus* per region in the longitudinal analysis (all years). Error bars represent 95% confidence intervals. Asterisks indicate level of statistical significant (** = p <0.01)

Mycoplasma coccoides

The longitudinal analysis showed that the best model (lowest AICc value; 677.786) included time, sex and age class (Table 3). Time was shown to be a significant factor (GLMM, family = Binomial, $z = -2.175$, $p < 0.05$), with a negative correlation (-0.1) between prevalence and time (years) (Pearson's correlation test, t=-2.2929, p < 0.05). This can be observed in Figure 6, where a reduction in prevalence is seen over time. Age class was also shown to affect prevalence (Age class: Mature, GLMM, family = Binomial, $z = -3.04$, $p < 0.01$). Post-hoc analysis demonstrated young adults being more infected than mature adults, however, no statistical differences were observed between mature adults and juveniles and between juveniles and young adults (Tukey Kramer analysis; Young adults – Mature, $p < 0.05$; Mature – Juveniles, $p = 0.97$; Young adults – Juveniles, $p = 0.097$).

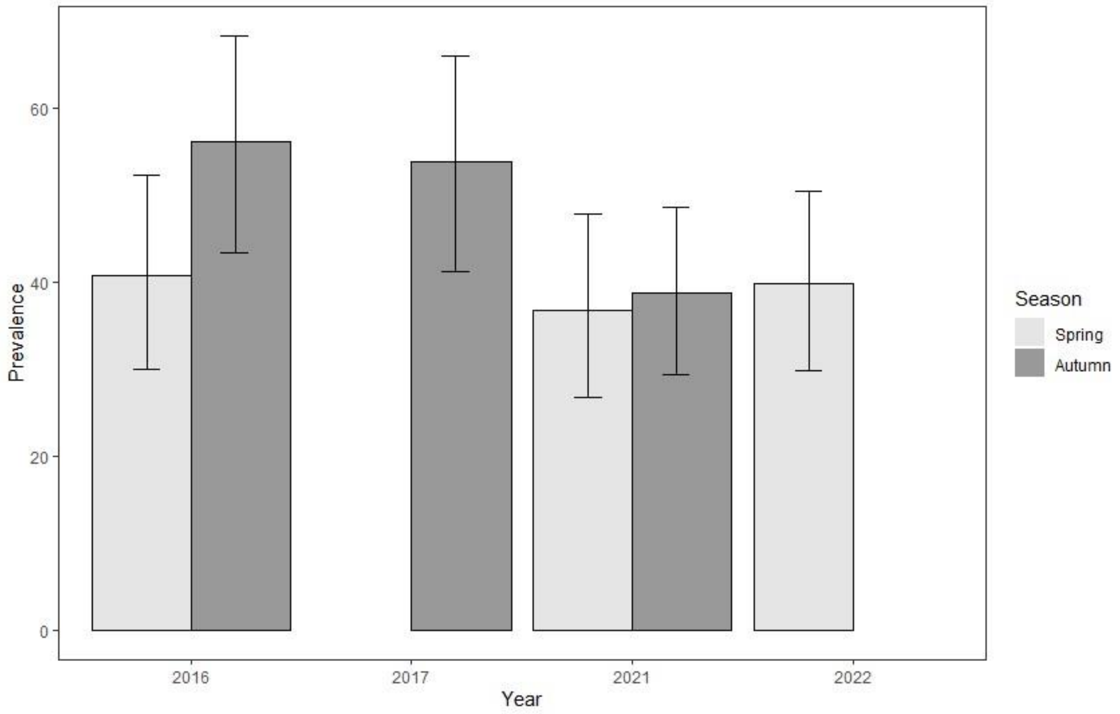

Figure 6. Prevalence of *Mycoplasma coccoides* in *Apodemus sylvaticus* for each season and year sampled. Error bars represent 95% confidence intervals.

Clethrionomys glareolus

Regarding *C. glareolus* in Ireland, the patterns of infection with Sarcocystidae observed during longitudinal analyses were examined.

Sarcocystidae

The longitudinal analysis of the prevalence of Sarcocystidae in *C. glareolus* found the best model (lowest AICc value; 460.593) included time, season, region, sex, age class and an interaction between time and season. A high VIF was observed for the interaction and hence it was removed from the final model (AICc value; 499.807 (Table 3). Time was shown to be statistically significant (GLMM, family = Binomial, $z = -4.849$, $p < 0.001$). Post-hoc analysis found that prevalence is negatively correlated (-0.22) with time (years since baseline) (Pearson's correlation test; $t = -4.6122$, $p < 0.001$). Higher prevalence was observed in spring compared to autumn (GLMM, family = Binomial, $z = 3.547$, $p < 0.001$). Region was shown to be statistically significant (Region: Front 2021, GLMM, family = Binomial, $z = 2.143$, $p < 0.05$). Post-hoc analysis demonstrates that the

core had a lower prevalence compared to front 2016 but not statistically different from front 2021, while no statistical difference was observed between front 2016 and front 2021 (Tukey Kramer analysis; Core – Front 2016, $p < 0.05$; Core – Front 2021, $p = 0.5$; Front 2016 – Front 2021, $p = 0.53$). Females had higher prevalences than males (GLMM, family = Binomial, $z = -2.216$, $p < 0.05$). Finally, age class was shown to be a statistically significant factor (Age class: Mature, GLMM, family = Binomial, $z = -2.322$, $p < 0.05$). Post-hoc analysis showed that juveniles were more infected than mature adults. No statistical difference was observed between mature adults and young adults, or young adults and juveniles (Tukey Kramer analysis; Juveniles – Mature, $p < 0.01$; Juveniles – Young adults, $p = 0.14$; Mature – Young adults, $p = 0.15$).

Separate binomial GLMMs for each season were run on the longitudinal models to account for the removed interaction between season and time (Table 3). In spring, the best model (lowest AICc value; 232.189) included region and age class. Region was shown to be statistically significant (Region: Front 2016, GLMM, family = Binomial, $z = 2.188$, $p < 0.05$; Region: Front 2021, GLMM, family = Binomial, $z = 3.769$, $p < 0.001$). Post-hoc analysis demonstrated that the core had a lower prevalence when compared to both front 2016 and front 2021, while no difference was observed in the post hoc analysis between the two front regions identified in 2016 and 2021 (Tukey Kramer analysis; Core – Front 2016 $p < 0.01$; Core – Front 2021 $p < 0.001$; Front 2016 – Front 2021, $p = 0.92$). Age class was also statistically significant (Age class: Mature, GLMM, family = Binomial, $z = -2.112$, $p < 0.05$). Post-hoc analysis showed juveniles and young adults more likely to be infected compared to mature adults, but no statistical difference was observed between juveniles and young adults (Tukey Kramer analysis; Mature – Juveniles, $p < 0.01$; Mature – Young adults, $p < 0.05$; Young adults – Juveniles, $p = 0.14$).

Finally, the best model (lowest AICc value; 224.497) for autumn included time, sex, and age class. No effect for region was observed. Time was shown to be statistically significant (GLMM, family = Binomial, $z = -6.766$, $p < 0.001$) with time being negatively correlated (-0.5) with prevalence (Pearsons's correlation test, $t = -8.5352$, $p < 0.001$). Females had higher prevalences compared to males (GLMM, family = Binomial, $z = -2.231$, $p < 0.05$). Age class did not significantly influence the prevalence of Sarcocystidae in this analysis (Age class: Mature, GLMM, family = Binomial, z = -0.848, $p = 0.4$; Age class; Juvenile, GLMM, family = Binomial, z = 1.727, $p = 0.084$).

Discussion

The aim of this study was to investigate the effect an invasive species may have on native pathogen communities, including zoonotic species, using the bio-invasion of Ireland by *C. glareolus* as a model system. While previous studies on rodent-borne microparasites in Ireland have focused on single bacterial species, such as Bartonella or Borrelia (70-72), this is the first time 16SrRNA metabarcoding techniques have been used to identify the full communities of potentially zoonotic pathogens, and their prevalence levels, that are circulating in Irish populations of *C. glareolus and A. sylvaticus*. Furthermore, this study has created an important baseline for rodent-borne disease studies in Ireland, and highlights new important areas of research to guide future research in Ireland, and potentially other invasion systems (e.g (73)).

Rodent-borne pathogens observed in Ireland

Firstly, we discovered that Irish rodents exhibit a depauperate pathogen richness, with OTUs relating to only four pathogenic bacterial species and one family of protozoans in sampled rodent populations. Although the species in the Sarcocystidae OTUs were not identified in the present study, comparison with published sequences using NCBI BLAST indicated these may contain pathogens of veterinary and medical importance, including *Toxoplasma, Eimeria* or *Neospora*. Finally, this study examined the importance of considering time when examining the effect biological invaders have on pathogen communities. For example, in contrast to a previous study by Telfer *et al.,* (72) we detected *Bartonella* in *C. glareolus* in Ireland, albeit at a low prevalence rate. This result was surprising as the previous study had postulated that *C. glareolus* in Ireland might be resistant to native strains (72). Further molecular work is required to determine if the *Bartonella* observed during this study is the same species of *Bartonella* observed in mice during the study by Telfer *et al.,* (72).

It is also worth noting that despite being native, *A. sylvaticus* also exhibited a depauperate community of potentially pathogenic bacterial taxa when compared to French populations, with no *Anaplasma, Candidatus Neoehrlichia, Francisella* or *Orientia* observed in the Irish population. This is consistent with island biogeography theory, which states that species diversity is limited by their size and isolation from the mainland (74, 75). The absence of certain vector-borne pathogens might also be explained by the low vector diversity in Ireland. For example, reviews in Ireland and France have shown Ireland to have 13 species of ticks, compared to approximately 40 tick species in France (76, 77). However, assays specifically targeting these pathogens would need to be carried out to confirm their absence. Furthermore, *Borrelia* was also notably absent from Irish

rodents in this study, although it had been detected, albeit at low prevalences, in Irish rodents by previous studies (70, 71). This discrepancy may again be a result of the methods and target organ used in the present study, with previous studies using ear biopsies and species specific PCR (70, 71). Also, while *Apodemus* species and *C. glareolus* are considered important aspects of the *Borrelia* life cycle in Europe, these rodents may play a limited role as reservoir in Ireland, with the rodent-associated, *Borrelia afzelii*, being less common in Ireland (70, 71, 78, 79). Additionally, the absence of *Anaplasma* from rodents sampled in the present study agree with findings reported by Flattery *et al.* (80), who screened questing nymphal *Ixodes ricinus* ticks collected across Ireland for the presence of *Anaplasma phagocytophilum* and only found groEL ecotype 1, a strain that is not thought to be associated with rodents.

Enemy release in the invasive *Clethrionomys glareolus*

Two patterns consistent with the enemy release hypothesis were observed for bacterial pathogens in the invasive *C. glareolus* population in Ireland for the first time. Firstly, a comparison of the number of bacterial taxa in Irish and French *C. glareolus* populations indicated that the population of *C. glareolus* sampled in Ireland had fewer potentially pathogenic bacterial taxa than conspecifics in their native range. This can be seen particularly in the 2021 data, where the Irish *C. glareolus* individuals had fewer potentially pathogenic bacterial taxa and lower prevalences compared to *C. glareolus* populations in France. In particular, a number of potentially pathogenic taxa were completely absent from *C. glareolus* populations in Ireland including: *Anaplasma, Candidatus Neoehrlichia, Francisella and Orientia*. Further studies should be conducted to confirm this and incorporate consistent aging techniques in both countries allowing for more in-depth statistical analysis between samples from Ireland and France. Secondly, *C. glareolus* exhibited enemy release from potentially pathogenic bacterial taxa in comparison to the native *A. sylvaticus* in Ireland. While *C. glareolus* was found to have one more bacterial taxon when compared to *A. sylvaticus, C. glareolus* has lower prevalences of these bacterial taxa in the overall data, and a significantly lower pathogen species richness compared to *A. sylvaticus* in the invasion gradient analysis (2016 only).

These findings are comparable to studies investigating the helminth communities in the invasive *C. glareolus* in Ireland (23, 39). These authors found the same two examples of enemy release, i.e. *C. glareolus* had a lower helminth species richness compared to its native range, and to the native *A. sylvaticus* in Ireland (23, 39).

Likewise, similar patterns have been observed in other invasion systems. For example in the Senegal invasion system, Diagne *et al.* (24) demonstrated that native rodents (*Mastomys erythroleucus* and *Mastomys natalensis*) had a greater species richness of pathogenic bacterial OTUs when compared to non-native rodents (*Mus musculus domesticus* and *Rattus rattus*). These findings from investigating invasion systems in different countries and invasive species can provide insights into the underlying mechanisms contributing to how these species survive, establish and outcompete native fauna when colonising new environments. For example, this pattern of enemy release could allow the invasive species to direct more energy from immune responses into growth or fecundity, potentially giving invasive species a competitive advantage over native species (81).

Stuart *et al.* (39) described a third example of enemy release in the Irish population of *C. glareolus,* namely the reduced abundance of the directly transmitted nematode *A. tianjinsensis* in *C. glareolus* in the invasion front compared to the invasion core in spring and autumn in 2016 (39). A similar example of enemy release at the invasion front was not found during this present study. In contrast, in the invasion gradient analysis, *C. glareolus* was more likely to be infected in the invasion front, compared to the more established invasion core, with significantly higher prevalences of Sarcocystidae in front 2016 compared to the core. This pattern was also observed in 2016 for the single Sarcocystidae OTU which seems to be specific to *C. glareolus*, with no *A. sylvaticus* found infected by this OTU in this study. These findings are in direct contrast to the enemy release hypothesis, as the more recently invaded populations exhibited higher infection rates, highlighting that infection patterns may be highly pathogen specific.

This result was all the more surprising given that male *C. glareolus* at the invasion front are thought to be more timid and less exploratory than their conspecifics at core sites (82), characteristics which would be expected to reduce their exposure to pathogens. On the other hand, there also seems to be a strong link between spatial behaviour and behaviour traits, with shy but thorough explorers using larger areas (82). If animals are exploring larger areas at the expansion front, this could potentially result in greater exposures to pathogens.

Finally, a possible explanation for the increased infection rate at the invasion front might lie in the immunology of *C. glareolus* during its invasion of Ireland. Pathogens may lag behind biological invasions, as the population density of the invading species at the front is too low to maintain sufficient transmission. Consequently, the reallocation of resources from immune responses, known as the evolution of increased competitive ability hypothesis and enemy release from pathogens, could be favoured in invasive populations (22, 47). This could potentially leave *C. glareolus* in the invasion front relatively immunologically naïve and hence, susceptible to

increased Sarcocystidae infection when the population increases over a certain threshold. This would be further supported by the findings in the longitudinal analysis, that juveniles have a higher prevalence of Sarcocystidae compared to mature adults, as juveniles would potentially be more immunologically naïve. In a study of the population genomics of *C. glareolus* in Ireland, White *et al.* (83) identified the selection of genes with immunological function in *C. glareolus* furthest from the point of introduction. However, to fully understand these mechanisms further investigation of the eco-immunology of invasive *C. glareolus* populations, and identification of the species present within these Sarcocystidae OTUs is required.

Effect of *Clethrionomys glareolus* invasion on pathogen communities in the native *Apodemus sylvaticus*

The effect of the *C. glareolus* invasion on the pathogen communities in the native *A. sylvaticus* was also investigated. It was hypothesised that the presence of *C. glareolus* might affect the pathogen communities in the native *A. sylvaticus*. However, no dilution or amplification effect resulting from the *C. glareolus* invasion gradient on pathogen species richness in *A. sylvaticus* was observed in the present study. Likewise, in the invasion gradient analysis (2016 data only), no effect for invasion region was found on the prevalence of *A. sylvaticus'* pathogens. However, using the longitudinal data, the increased relative abundance of *C. glareolus* at the site of introduction apparently caused a dilution effect for *M. haemomuris* in *A. sylvaticus*. In this instance the core showed significantly reduced prevalences of *M. haemomuris* in *A. sylvaticus* compared to conspecifics from the invasion front regions (front 2016 and front 2021). No difference was observed between front 2016 and front 2021 indicating that the reduction in *M. haemomuris* prevalence may be directly attributable to the higher *C. glareolus* abundance at the core. The potential reason for a dilution effect being observed in the longitudinal data and not in the 2016 only data is likely due to the increased statistical power in the longitudinal analysis, as a result of the increased sample size.

In a previous study investigating the impact of forest anthropization on small mammal pathogens, Bouilloud (84) detected a dilution effect for *M. haemomuris* (Strain 2) associated with increased small mammal diversity in France. The author argued that the declining abundance of its most competent host, *C. glareolus,* as host species diversity increased, might explain the dilution of *M. haemomuris* at these sites. The dilution of *M. haemomuris,* in *A. sylvaticus* in Ireland due to the presence of *C. glareolus,* and the dilution of the strain 2 in France, presumably due to increased small mammal diversity, demonstrate that despite the multiple factors that can alter rodent populations (e.g. climate change, invasive species, deforestation and urbanisation), that

understanding the underlying mechanisms behind rodent-borne disease can allow for better predictions of disease transmission and improved disease management. Interestingly, the dilution pattern identified for *M. haemomuris* was not observed for *M. coccoides*. The driver behind the different patterns of these *Mycoplasma* spp. may possibly be a result of the specific behaviour of the respective vector species, *Polyplax serrata* in the case of *M. coccoides* and *Polyplax spinulosa* in the case of *M. haemomuris* (85, 86). However, studies on these pathogens vector species are based on mice and rats' laboratory experiments only. Therefore, field-based studies are needed to confirm the possibility that *C. glareolus* might be a less favourable host for the vector transmitting *M. haemomuris*, or the bacteria itself, in Ireland, which could result in a dilution effect in the native *A. sylvaticus.*

Surprisingly, we detected no dilution effect for *Bartonella* in *A. sylvaticus* in this present study. *Bartonella birtlesii* and *Bartonella taylorii* have previously been recorded in Ireland, with prevalences in *A. sylvaticus* decreasing with increased *C. glareolus* densities (72). These seemingly contradictory observations may be due to the time that has elapsed since the study by Telfer *et al.* (72) and the present study. It is possible for example, that the initial invasion of *C. glareolus* disrupted these pathogens, but they have recovered since. On the other hand, only a single *C. glareolus* individual was found to be infected with *Bartonella* here, corroborating the suggestion that *C. glareolus* in Ireland is not a competent host for this pathogen species. Hence, the observed difference may stem from the detection techniques employed, as Telfer *et al.* (72) utilized bacterial cultivation and genus-specific PCR assays, while this present study only found one OTU of *Bartonella,* using 16SrRNA amplification and illumina sequencing. Further work, using genus specific primers, such as those used by Telfer *et al.* (72) would be needed to examine *Bartonella* positive rodents to determine species identity, and hence identify whether dilution does still occur in the Irish *A. sylvaticus* population.

Changes in rodent pathogen communities over time

Finally, the effect of time on the prevalence of the various pathogenic taxa, as the *C. glareolus* invasion progressed was examined. Prevalence decreased between 2016 and 2022, for both *M. coccoides* in *A. sylvaticus* and Sarcocystidae in *C. glareolus*. However, examining the spring longitudinal data only, *Bartonella* prevalence in *A. sylvaticus* increased between 2016 and 2022. These patterns show that pathogen communities are dynamic, and longitudinal studies are essential for fully understanding the changes that occur in pathogen communities. Cornet *et al.* (22), expressed the need for longitudinal studies to examine the time-lag for invasive species

between invasion and adapting to the invasion front. A recent review by Brian and Catford (87) highlighted that time since introduction is a significant factor when investigating the enemy release hypothesis, as enemy impact on the invader will potentially increase with time, as pathogens adapt to the invasive species. In our case, the dilution effect for *M. haemomuris* in *A. sylvaticus* was only observed in the longitudinal data, an observation that could potentially have been missed in a single timepoint study. Moreover, *M. haemomuris, M. penetrans* and *Bartonella* were not detected in *C. glareolus* in the baseline 2016 data. While this could be attributed to the increased sample size afforded by multiple years of sampling, focusing on a single year, could have resulted in missing the dilution for *M. haemomuris* in *A. sylvaticus* and the assumption that *C. glareolus* is not infected by these pathogens in Ireland. Similarly, the recently reported *Babesia microti* 'Munich' strain in Ireland was detected exclusively in *C. glareolus* located in the eastern part of the country, which corresponds to the *C. glareolus* invasion front (73). The primary vector of this strain, *Ixodes trianguliceps*, has also been mainly reported in eastern Ireland (88). If the assumption that *B. microti* is confined to the eastern regions of Ireland is correct, this would further demonstrate the importance of long-term studies on the pathogens of invasive species, to monitor the emergence of new pathogens in newly colonised areas. Finally, more long-term data is needed to fully understand if rodents and their pathogens fluctuate through multi-annual cycles, which is seen in other countries where long-term data is gathered, such as Finland (89-91).

Conclusion

In conclusion, patterns consistent with the enemy release hypothesis were observed in the Irish *C. glareolus* population. Despite exhibiting higher prevalences of Sarcocystidae, *C. glareolus* in Ireland shows reduced pathogen species richness, and lower prevalences of bacterial taxa, compared to the native *A. sylvaticus* and fewer bacterial taxa compared to the native range of the species. These findings increase our understanding of the mechanism of how invasive species thrive in new environments and outcompete native fauna, however, eco-immunological data are required to fully understand these patterns of enemy release. Secondly, the present study showed the disruptive effect of non-native host species on native pathogen systems, with the example of a dilution effect and reduced prevalence of *M. haemomuris* in *A. sylvaticus* at the invasion core compared to invasion fronts, where *C. glareolus* populations exhibit higher abundance. These findings show how changes in biodiversity, such as the addition of new hosts with varying levels of competencies for a given pathogen, may disrupt pathogen communities and host-pathogen dynamics. Further, work is also needed to identify the

Sarcocystidae and *Bartonella* species detected in this study in order to fully understand the pathogen community dynamics, as genetic differences may reveal different levels of host specificity and help explain host-pathogen interactions. Finally, time, as the *C. glareolus* invasion progresses, was shown to be correlated, both positively and negatively, with three pathogen taxa during this study, illustrating that invasion systems are dynamic, and pathogen communities can fluctuate as the invader becomes more established. By conducting a longitudinal investigation, we demonstrate that long-term data are essential to fully understand eco-evolutionary processes driving invasion dynamics and success, preventing conclusions drawn from single timepoint studies being assumed as long-term changes in pathogen communities.

Acknowledgements

We would like to thank Ian Montgomery, Colin Lawton and John Rochford for loaning their traps. We would also like to thank Breda Fitzpatrick, Patrick Dalton, Hugh O'Connor and the other technical staff at MTU for their help and loan of equipment. Finally, we would like to thank TCD students, volunteers and interns, Laura McCarthy, Lucie Veitová and Paula Curtin for their help in the field and lab processing the Irish samples. We are grateful to Audrey Weber (AGAP laboratory) and the GenSeq facilities for the Illumina MiSeq sequencing, and the Genotoul bioinformatics platform Toulouse Midi-Pyrénées (Bioinfo Genotoul). Thank you to all the BioRodDis team members who have been vital to this study through the sharing of data, discussion of findings and statistical analyses. We would also like to thank Niamh Reilly for helping proofread the manuscript.

Statements & Declarations

Funding

This research was funded through the 2018–2019 BiodivERsA joint call for research proposals, under the BiodivErsA3 ERA-Net COFUND programme, and with the funding organisations EPA Research Programme, Agence Nationale de la Recherche (ANR), Research Foundation–Flanders (FWO), National Science Centre, Poland (NCN) and Deutsche Forschungsgemeinschaft (DFG). This project is funded under the EPA Research Programme 2021–2030 and cofunded by Agence Nationale de la Recherche (ANR), Research Foundation–Flanders (FWO), National Science Centre, Poland (NCN) and Deutsche Forschungsgemeinschaft (DFG).

This project is funded under the EPA Research Programme 2021-2030. The EPA Research Programme is a Government of Ireland initiative funded by the Department of the Environment, Climate and Communications (project number 2019-NC-MS-10).

2016 and 2017 sampling was funded by the Irish Research Council, Government of Ireland Postdoctoral Fellowship Programme and by the Marie Curie Research Grants Scheme, European Union, grant (749312, INVASIMMUN).

The funders had no role in study design, data collection and analysis, decision to publish, or preparation of the manuscript.

The author, Andrew McManus, received a stipend to complete this research from the EPA Research Programme as part of their PhD studies.

Ethics statement:

This study was carried out under ethical approval from the universities Trinity College Dublin animal research ethics committee and Munster Technological University ethics board, in compliance with the Health Products Regulatory Association (HPRA) of Ireland for euthanasia of the rodents sampled (Authorisation Number: AE22171/I004).

Competing Interests:

The authors declare no competing or financial interests.

Author contributions.

Conceptualization: Andrew McManus, Celia Holland, Nathalie Charbonnel, Maxime Galan, Peter Stuart

Data Curation: Andrew McManus, Maxime Galan, Peter Stuart

Formal Analysis: Andrew McManus, Nathalie Charbonnel, Maxime Galan, Vincent Sluydst, Peter Stuart

Funding Acquisition: Nathalie Charbonnel, Peter Stuart

Investigation: Andrew McManus, Celia Holland, Nathalie Charbonnel, Maxime Galan, Vincent Sluydst, Peter Stuart

Methodology: Andrew McManus, Celia Holland, Nathalie Charbonnel, Maxime Galan, Vincent Sluydst, Peter Stuart

Project Administration: Nathalie Charbonnel, Peter Stuart

Resources: Andrew McManus, Nathalie Charbonnel, Maxime Galan, Peter Stuart

Software: Andrew McManus, Nathalie Charbonnel, Maxime Galan, Vincent Sluydst

Supervision: Celia Holland, Nathalie Charbonnel, Peter Stuart

Validation: Andrew McManus, Nathalie Charbonnel, Maxime Galan, Vincent Sluydst

Visualization: Andrew McManus

Writing – Original Draft Preparation: Andrew McManus, Peter Stuart

Writing – Review & Editing: Andrew McManus, Celia Holland, Nathalie Charbonnel, Maxime Galan, Vincent Sluydst, Peter Stuart, Annetta Zintl

References

1. Jones KE, Patel NG, Levy MA, Storeygard A, Balk D, Gittleman JL, et al. Global trends in emerging infectious diseases. Nature. 2008;451(7181):990-3. doi: 10.1038/nature06536
2. Brown K, Leggat PA. Human monkeypox: current state of knowledge and implications for the future. Tropical medicine and infectious disease. 2016;1(1):8. doi: 10.3390/tropicalmed1010008
3. Mackenzie JS, Smith DW. COVID-19: a novel zoonotic disease caused by a coronavirus from China: what we know and what we don't. Microbiology Australia. 2020;41(1):45-50. doi: 10.1071/MA20013
4. Roche B, Garchitorena A, Guégan JF, Arnal A, Roiz D, Morand S, et al. Was the COVID-19 pandemic avoidable? A call for a "solution-oriented" approach in pathogen evolutionary ecology to prevent future outbreaks. Ecology letters. 2020. doi: 10.1111/ele.13586
5. Heinrich ML, Boisen ML, Nelson DK, Bush DJ, Cross RW, Koval AP, et al. Antibodies from Sierra Leonean and Nigerian Lassa fever survivors cross-react with recombinant proteins representing Lassa viruses of divergent lineages. Scientific Reports. 2020;10(1):1-19. doi: 10.1038/s41598-020-72539-w

6. Dharmarajan G, Li R, Chanda E, Dean KR, Dirzo R, Jakobsen KS, et al. The animal origin of major human infectious diseases: what can past epidemics teach us about preventing the next pandemic? Zoonoses. 2022;2(1).
7. Dhawan M, Choudhary OP. Emergence of monkeypox: risk assessment and containment measures. Travel Medicine and Infectious Disease. 2022;49:102392. doi: 10.1016/j.tmaid.2022.102392
8. Daszak P, Cunningham AA, Hyatt AD. Emerging infectious diseases of wildlife--threats to biodiversity and human health. science. 2000;287(5452):443-9. doi: 10.1126/science.287.5452.443
9. Wikelski M, Foufopoulos J, Vargas H, Snell H. Galápagos birds and diseases: invasive pathogens as threats for island species. Ecology and Society. 2004;9(1).
10. Wilcox BA, Gubler DJ. Disease ecology and the global emergence of zoonotic pathogens. Environmental Health and Preventive Medicine. 2005;10(5):263-72. doi: 10.1265/ehpm.10.263
11. Hatcher MJ, Dick JT, Dunn AM. Disease emergence and invasions. Functional Ecology. 2012;26(6):1275-87. doi: 10.1111/j.1365-2435.2012.02031.x
12. Becker DJ, Albery GF, Kessler MK, Lunn TJ, Falvo CA, Czirják GÁ, et al. Macroimmunology: The drivers and consequences of spatial patterns in wildlife immune defence. Journal of Animal Ecology. 2020;89(4):972-95. doi: 10.1111/1365-2656.13166
13. IPBES. Global assessment report on biodiversity and ecosystem services of the Intergovernmental Science-Policy Platform on Biodiversity and Ecosystem Services. In: Brondízio ES, Settele J, Díaz S, Ngo HT, (Editors), editors.: IPBES secretariat, Bonn, Germany; 2019. doi: 10.5281/zenodo.3831673
14. Schreiner CL, Nuismer SL, Basinski AJ. When to vaccinate a fluctuating wildlife population: Is timing everything? Journal of Applied Ecology. 2020;57(2):307-19. doi: 10.1111/1365-2664.13539
15. Bezerra-Santos MA, Dantas-Torres F, Mendoza-Roldan JA, Thompson RA, Modry D, Otranto D. Invasive mammalian wildlife and the risk of zoonotic parasites. Trends in parasitology. 2023;39(9):786-98. DOI: 10.1016/j.pt.2023.06.004
16. Eby P, Peel AJ, Hoegh A, Madden W, Giles JR, Hudson PJ, et al. Pathogen spillover driven by rapid changes in bat ecology. Nature. 2023;613(7943):340-4. doi: 10.1038/s41586-022-05506-2
17. Bonesi L, Palazon S. The American mink in Europe: status, impacts, and control. Biological conservation. 2007;134(4):470-83. doi: 10.1016/j.biocon.2006.09.006
18. Salgado I. Is the raccoon (*Procyon lotor*) out of control in Europe? Biodiversity and Conservation. 2018;27(9):2243-56. doi: 10.1007/s10531-018-1535-9
19. Keane RM, Crawley MJ. Exotic plant invasions and the enemy release hypothesis. Trends in Ecology & Evolution. 2002;17(4):164-70. DOI: 10.1016/S0169-5347(02)02499-0
20. Torchin ME, Lafferty KD, Dobson AP, McKenzie VJ, Kuris AM. Introduced species and their missing parasites. Nature. 2003;421(6923):628-30. doi: https://doi.org/10.1038/nature01346
21. Lee KA, Klasing KC. A role for immunology in invasion biology. Trends in Ecology & Evolution. 2004;19(10):523-9. DOI: 10.1016/j.tree.2004.07.012
22. Cornet S, Brouat C, Diagne C, Charbonnel N. Eco-immunology and bioinvasion: Revisiting the evolution of increased competitive ability hypotheses. Evolutionary Applications. 2016;9(8):952-62. doi: 10.1111/eva.12406
23. Loxton KC, Lawton C, Stafford P, Holland CV. Reduced helminth parasitism in the introduced bank vole (*Myodes glareolus*): More parasites lost than gained. International Journal for Parasitology: Parasites and Wildlife. 2016;5(2):175-83. doi: 10.1016/j.ijppaw.2016.05.005
24. Diagne C, Galan M, Tamisier L, d'Ambrosio J, Dalecky A, Bâ K, et al. Ecological and sanitary impacts of bacterial communities associated to biological invasions in African commensal rodent communities. Scientific reports. 2017;7(1): 14995. doi: 10.1038/s41598-017-14880-1

25. Chalkowski K, Lepczyk CA, Zohdy S. Parasite ecology of invasive species: conceptual framework and new hypotheses. Trends in parasitology. 2018;34(8):655-63. DOI: 10.1016/j.pt.2018.05.008
26. Dunn AM, Hatcher MJ. Parasites and biological invasions: parallels, interactions, and control. Trends in Parasitology. 2015;31(5):189-99. DOI: 10.1016/j.pt.2014.12.003
27. Schmidt KA, Ostfeld RS. Biodiversity and the dilution effect in disease ecology. Ecology. 2001;82(3):609-19. doi: 10.1890/0012-9658(2001)082[0609:BATDEI]2.0.CO;2
28. Tierney PA, Caffrey JM, Vogel S, Matthews SM, Costantini E, Holland CV. Invasive freshwater fish (*Leuciscus leuciscus*) acts as a sink for a parasite of native brown trout Salmo trutta. Biological Invasions. 2020;22(7):2235-50. doi: 10.1007/s10530-020-02253-1
29. Keesing F, Ostfeld RS. Dilution effects in disease ecology. Ecology Letters. 2021;24(11):2490-505. doi: 10.1111/ele.13875
30. Kelly D, Paterson R, Townsend C, Poulin R, Tompkins D. Parasite spillback: a neglected concept in invasion ecology? Ecology. 2009;90(8):2047-56. doi: 10.1890/08-1085.1
31. Loxton KC, Lawton C, Stafford P, Holland CV. Parasite dynamics in an invaded ecosystem: helminth communities of native wood mice are impacted by the invasive bank vole. Parasitology. 2017;144(11):1476-89. doi: 10.1017/S0031182017000981
32. Diagne C, Ballesteros-Mejia L, Cuthbert RN, Bodey TW, Fantle-Lepczyk J, Angulo E, et al. Economic costs of invasive rodents worldwide: the tip of the iceberg. PeerJ. 2023;11:e14935. doi: 10.7717/peerj.14935
33. Meerburg BG, Singleton GR, Kijlstra A. Rodent-borne diseases and their risks for public health. Critical Reviews in Microbiology. 2009;35(3):221-70. doi: 10.1080/10408410902989837
34. Pedersen AB, Babayan SA. Wild immunology. Molecular ecology. 2011;20(5):872-80. doi: 10.1111/j.1365-294X.2010.04938.x
35. Han BA, Schmidt JP, Bowden SE, Drake JM. Rodent reservoirs of future zoonotic diseases. Proceedings of the National Academy of Sciences. 2015;112(22):7039-44. doi: 10.1073/pnas.1501598112
36. Jackson J. Immunology in wild nonmodel rodents: an ecological context for studies of health and disease. Parasite Immunology. 2015;37(5):220-32. doi: 10.1111/pim.12180
37. McManus A, Holland CV, Henttonen H, Stuart P. The Invasive Bank Vole (*Myodes glareolus*): A Model System for Studying Parasites and Ecoimmunology during a Biological Invasion. Animals. 2021;11(9):2529. doi: 10.3390/ani11092529
38. Diagne C, Granjon L, Gueye MS, Kane M, Niang Y, Tatard C, et al. Association between temporal patterns in helminth assemblages and successful range expansion of exotic *Mus musculus domesticus* in Senegal. Biological Invasions. 2020;22(10):3003-16. doi: 10.1007/s10530-020-02304-7
39. Stuart P, Paredis L, Henttonen H, Lawton C, Torres CAO, Holland CV. The hidden faces of a biological invasion: parasite dynamics of invaders and natives. International Journal for Parasitology. 2020;50(2):111-123. doi: 10.1016/j.ijpara.2019.11.003.
40. Marnell F, Looney D, Lawton C. Ireland Red List No. 12: Terrestrial Mammals. National Parks and Wildlife Service, Department of the Culture, Heritage and the Gaeltacht, Dublin, Ireland; 2019.
41. Stuart P, Mirimin L, Cross T, Sleeman D, Buckley NJ, Telfer S, et al. The origin of Irish bank voles *Clethrionomys glareolus* assessed by mitochondrial DNA analysis. The Irish Naturalists' Journal. 2007:440-6.
42. White TA, Lundy MG, Montgomery WI, Montgomery S, Perkins SE, Lawton C, et al. Range expansion in an invasive small mammal: influence of life-history and habitat quality. Biological Invasions. 2012;14(10):2203-15. doi: 10.1007/s10530-012-0225-x
43. Montgomery WI, Lundy MG, Reid N. 'Invasional meltdown': evidence for unexpected consequences and cumulative impacts of multispecies invasions. Biological Invasions. 2012;14(6):1111-25. doi: https://doi.org/10.1007/s10530-011-0142-4

44. Razzauti M, Galan M, Bernard M, Maman S, Klopp C, Charbonnel N, et al. A comparison between transcriptome sequencing and 16S metagenomics for detection of bacterial pathogens in wildlife. PLoS Neglected Tropical Diseases. 2015;9(8):e0003929. doi: 10.1371/journal.pntd.0003929
45. Hazel S, Bennett M, Chantrey J, Bown K, Cavanagh R, Jones T, et al. A longitudinal study of an endemic disease in its wildlife reservoir: cowpox and wild rodents. Epidemiology & Infection. 2000;124(3):551-62. doi: 10.1017/S0950268899003799
46. Davis S, Calvet E, Leirs H. Fluctuating Rodent Populations and Risk to Humans from Rodent-Borne Zoonoses. Vector-Borne and Zoonotic Diseases. 2005;5(4):305-14. doi: 10.1089/vbz.2005.5.305
47. White TA, Perkins SE. The ecoimmunology of invasive species. Functional Ecology. 2012;26(6):1313-23. doi: 10.1111/1365-2435.12012
48. Galan M, Razzauti M, Bard E, Bernard M, Brouat C, Charbonnel N, et al. 16S rRNA amplicon sequencing for epidemiological surveys of bacteria in wildlife. MSystems. 2016;1(4):e00032-16. doi: 10.1128/msystems.00032
49. Bouilloud M, Galan M, Dubois A, Diagne C, Marianneau P, Roche B, et al. Three-way relationships between gut microbiota, helminth assemblages and bacterial infections in wild rodent populations. Peer Community Journal. 2023;3. doi: 10.24072/pcjournal.243.
50. Behnke J, Barnard C, Bajer A, Bray D, Dinmore J, Frake K, et al. Variation in the helminth community structure in bank voles (*Clethrionomys glareolus*) from three comparable localities in the Mazury Lake District region of Poland. Parasitology. 2001;123(4):401-14. doi: 10.1017/S0031182001008605
51. Abbate JL, Galan M, Razzauti M, Sironen T, Voutilainen L, Henttonen H, et al. Pathogen community composition and co-infection patterns in a wild community of rodents. Peer Community Journal; 2024. doi: 10.24072/pcjournal.370.
52. Kircher M, Sawyer S, Meyer M. Double indexing overcomes inaccuracies in multiplex sequencing on the Illumina platform. Nucleic Acids Research. 2011;40(1):e3-e. doi: https://doi.org/10.1093/nar/gkr771
53. Wickham H. Plyr 1.8.8 R package 2022 [Available from: https://CRAN.R-project.org/package=plyr].
54. Wickham H, Chang W, Henry L, Pedersen TL, Takahashi K, Wilke C, et al. GGplot2 3.4.3 R package 2023 [Available from: https://CRAN.R-project.org/package=ggplot2].
55. Scherer R. PropCIs R package 2018 [Available from: https://CRAN.R-project.org/package=PropCIs].
56. Pradel J, Bouilloud M, Loiseau A, Piry S, Galan M, Artige E, et al. Small terrestrial mammals (Rodentia and Soricomorpha) along a gradient of forest anthropisation (reserves, managed forests, urban parks) in France. Biodiversity Data Journal. 2022;10. doi: 10.3897/BDJ.10.e95214
57. Brooks M, Bolker B, Kristensen K, Maechler M, Magnusson A, McGillycuddy M, et al. glmmTMB 1.1.7 R package 2023 [Available from: https://CRAN.R-project.org/package=glmmTMB].
58. Dinno A. dunn.test: Dunn's Test of Multiple Comparisons Using Rank Sums 2024 [Available from: https://cran.r-project.org/package=dunn.test].
59. Bartoń K. MuMIn: Multi-Model Inference, R package 2023 [Available from: https://CRAN.R-project.org/package=MuMIn].
60. Hartig F, Lohse L. DHARMa 0.4.6 R package 2022 [Available from: https://CRAN.R-project.org/package=DHARMa].
61. Lüdecke D, Makowski D, Ben-Shachar MS, Patil I, Waggoner P, Wiernik BM, et al. Performance 0.10.4 R package 2023 [Available from: https://CRAN.R-project.org/package=performance].
62. Engbæk K, Lawson PA. Identification of *Bartonella* species in rodents, shrews and cats in Denmark: detection of two *B. henselae* variants, one in cats and the other in the long-tailed

field mouse. APMIS. 2004;112(6):336-41. doi: https://doi.org/10.1111/j.1600-0463.2004.apm1120603.x
63. Voller A, Bidwell DE. The effect of *Eperythrozoon coccoides* infection in mice on superimposed *Plasmodium chabaudi* and Semliki Forest Virus infections. Annals of Tropical Medicine & Parasitology. 1968;62(3):342-8. doi: https://doi.org/10.1080/00034983.1968.11686569
64. Feng SH, Lo SC. Induced mouse spleen B-cell proliferation and secretion of immunoglobulin by lipid-associated membrane proteins of *Mycoplasma fermentans* incognitus and *Mycoplasma penetrans*. Infection and Immunity. 1994;62(9):3916-21. doi: https://doi.org/10.1128/iai.62.9.3916-3921.1994
65. Harasawa R, Fujita H, Kadosaka T, Ando S, Rikihisa Y. Proposal for '*Candidatus* Mycoplasma haemomuris subsp. musculi' in mice, and '*Candidatus* Mycoplasma haemomuris subsp. ratti' in rats. International Journal of Systematic and Evolutionary Microbiology. 2015;65(Pt_2):734-7. doi: https://doi.org/10.1099/ijs.0.069856-0
66. Millán J, Di Cataldo S, Volokhov DV, Becker DJ. Worldwide occurrence of haemoplasmas in wildlife: Insights into the patterns of infection, transmission, pathology and zoonotic potential. Transboundary and Emerging Diseases. 2021;68(6):3236-56. doi: https://doi.org/10.1111/tbed.13932
67. Smith DD. The Sarcocystidae: *Sarcocystis, Frenkelia, Toxoplasma, Besnoitia, Hammondia*, and *Cystoisospora*. The Journal of Protozoology. 1981;28(2):262-6. doi: https://doi.org/10.1111/j.1550-7408.1981.tb02848.x
68. Sanchez SG, Besteiro S. The pathogenicity and virulence of *Toxoplasma gondii*. Virulence. 2021;12(1):3095-114.
69. Arendt M, Stadler J, Ritzmann M, Ade J, Hoelzle K, Hoelzle LE. Hemotrophic Mycoplasmas—Vector Transmission in Livestock. Microorganisms. 2024;12(7):1278. doi: https://doi.org/10.3390/microorganisms12071278
70. Gray J, Kirstein F, Robertson J, Stein J, Kahl O. *Borrelia burgdorferi* sensu lato in *Ixodes ricinus* ticks and rodents in a recreational park in south-western Ireland. Experimental & applied acarology. 1999;23:717-29. doi: 10.1023/A:1006233700194
71. Gray J, Robertson J, Key S. Limited role of rodents as reservoirs of *Borrelia burgdorferi* sensu lato in Ireland. European Journal of Epidemiology. 2000;16:101-3. doi: 10.1023/A:1007642908546
72. Telfer S, Bown KJ, Sekules R, Begon M, Hayden T, Birtles R. Disruption of a host-parasite system following the introduction of an exotic host species. Parasitology. 2005;130(6):661-8. doi: 10.1017/S0031182005007250
73. Zintl A, McManus A, Galan M, Diquattro M, Giuffredi L, Charbonnel N, et al. Presence and Identity Of *Babesia microti* In Ireland. Available at SSRN 4373187. 2023. doi: 10.1016/j.ttbdis.2023.102221
74. Poulin R, Morand S. The diversity of parasites. The quarterly review of biology. 2000;75(3):277-93. doi: 10.1086/393500
75. Chisholm RA, Fung T, Chimalakonda D, O'Dwyer JP. Maintenance of biodiversity on islands. Proceedings of the Royal Society B: Biological Sciences. 2016;283(1829):20160102. doi: 10.1098/rspb.2016.0102
76. Zintl A, Moutailler S, Stuart P, Paredis L, Dutraive J, Gonzalez E, et al. Ticks and Tick-borne diseases in Ireland. Irish Veterinary Journal. 2017;70(1):1-10. doi: 10.1186/s13620-017-0084-y
77. Boulanger N, Boyer P, Talagrand-Reboul E, Hansmann Y. Ticks and tick-borne diseases. Medecine et maladies infectieuses. 2019;49(2):87-97. doi: 10.1016/j.medmal.2019.01.007
78. Kirstein F, Rijpkema S, Molkenboer M, Gray J. The distribution and prevalence of B. *burgdorferi* genomospecies in *Ixodes ricinus* ticks in Ireland. European journal of epidemiology. 1997;13:67-72. doi: 10.1023/A:1007360422975
79. Zintl A, Zaid T, McKiernan F, Naranjo-Lucena A, Gray J, Brosnan S, et al. Update on the presence of *Ixodes ricinus* at the western limit of its range and the prevalence of B*orrelia*

burgdorferi sensu lato. Ticks and Tick-borne Diseases. 2020;11(6):101518. doi: 10.1016/j.ttbdis.2020.101518
80. Flattery A, McKiernan F, Browne J, Gray J, Zaid T, O'Connor J, et al. The prevalence and distribution of *Anaplasma phagocytophilum* genotypes in *Ixodes ricinus* nymphs collected from farm- and woodland sites in Ireland. Ticks and Tick-borne Diseases. 2022;13(3):101928. doi: 10.1016/j.ttbdis.2022.101928
81. Colautti RI, Ricciardi A, Grigorovich IA, MacIsaac HJ. Is invasion success explained by the enemy release hypothesis? Ecology letters. 2004;7(8):721-33. doi: 10.1111/j.1461-0248.2004.00616.x
82. Eccard JA, Mazza V, Holland C, Stuart P. The timid invasion: behavioural adjustments and range expansion in a non-native rodent. Proceedings of the Royal Society B. 2023;290(2003):20230823. doi: 10.1098/rspb.2023.0823
83. White TA, Perkins SE, Heckel G, Searle JB. Adaptive evolution during an ongoing range expansion: the invasive bank vole (*Myodes glareolus*) in Ireland. Molecular Ecology. 2013;22(11):2971-85. doi: 10.1111/mec.12343
84. Bouilloud M. Impact De L'anthropisation Sur Les Liens Entre Les Communautés De Petits Mammifères, Leur Microbiote Et Les Dangers Zoonotiques: PhD Thesis, Institut agro, Montpellier; 2023.
85. Eliot CP. The insect vector for the natural transmission of *Eperythrozoon coccoides* in mice. Science. 1936;84(2183):397-. DOI:10.1126/science.84.2183.397.a
86. Crystal MM. The mechanism of transmission of *Haemobartonella muris* (Mayer) of rats by the spined rat louse, *Polyplax spinulosa* (Burmeister). The Journal of parasitology. 1958:603-6. doi: 10.2307/3274543
87. Brian JI, Catford JA. A mechanistic framework of enemy release. Ecology Letters. 2023 ;26(12):2147-66. doi : 10.1111/ele.14329
88. Kelly TC, Sleeman DP, Fennessy GJ, Dillon A, Walton GA, Gray J. The Mammal Ticks (Acari: Ixodoidea) of Ireland: Their Distribution and Hosts. The Irish Naturalists' Journal. 2001;26(10):363-70.
89. Haukisalmi V, Henttonen H, Tenora F. Population dynamics of common and rare helminths in cyclic vole populations. The Journal of Animal Ecology. 1988:807-25. doi: 10.2307/5094
90. Laakkonen J, Oksanen A, Soveri T, Henttonen H. Dynamics of intestinal coccidia in peak density *Microtus agrestis, Microtus oeconomus* and *Clethrionomus glareolus* populations in Finland. Ecography. 1998;21(2):135-9. doi: 10.1111/j.1600-0587.1998.tb00667.x
91. Voutilainen L, Kallio ER, Niemimaa J, Vapalahti O, Henttonen H. Temporal dynamics of Puumala hantavirus infection in cyclic populations of bank voles. Scientific Reports. 2016;6(1):1-15. doi: 10.1038/srep21323

Supporting Information

Supplementary Table 1 – Field work dates. This details the date in which field work was conducted

Supplementary Table 2 – Prevalence summary region and year. This provides a summary of the pathogen prevalences per host species for each invasion region and year during this study.

Fig 1

Click here to access/download;Figure;Figure 1..tif

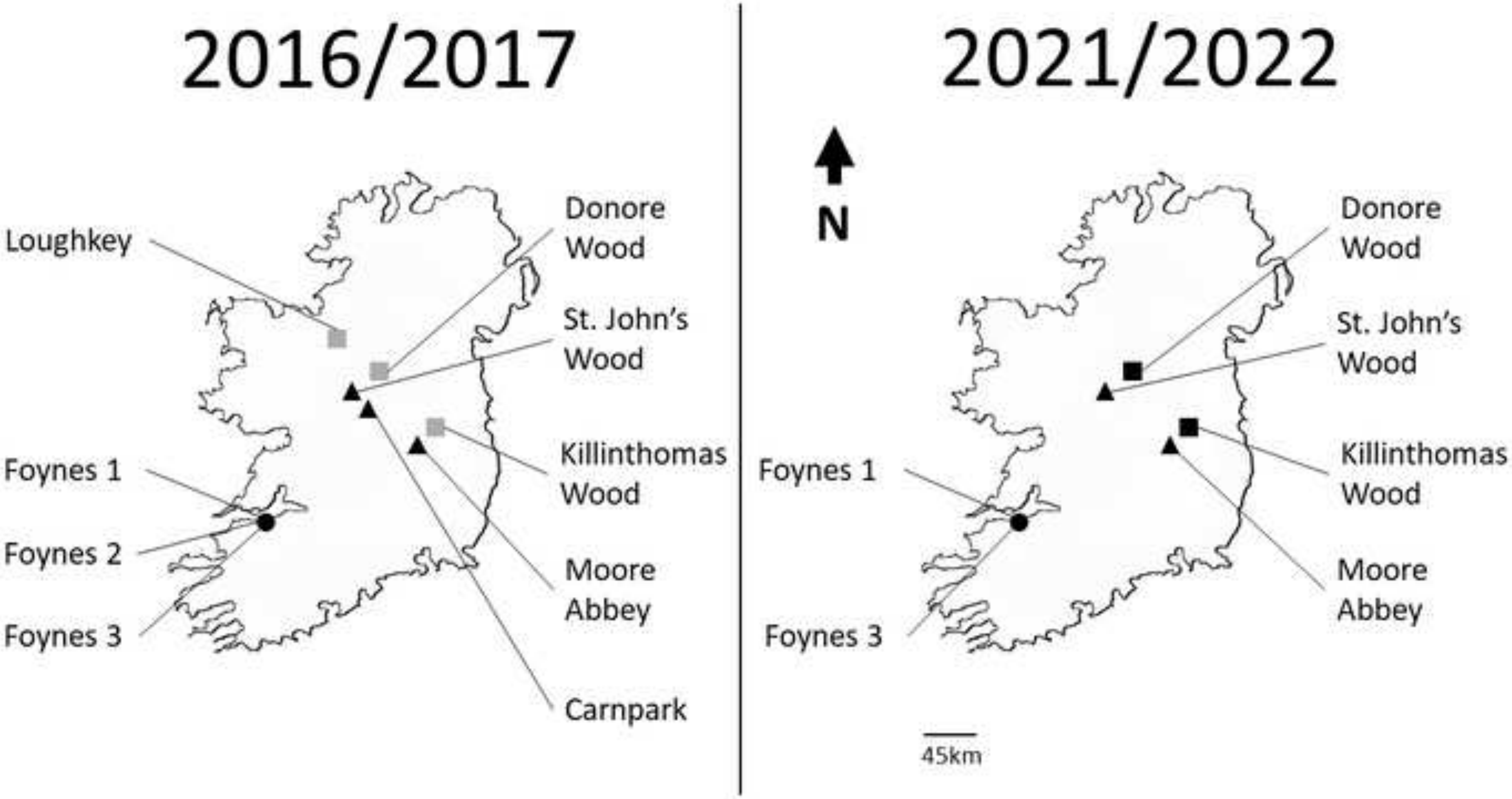

Fig 2

Click here to access/download;Figure;Figure 2..tif

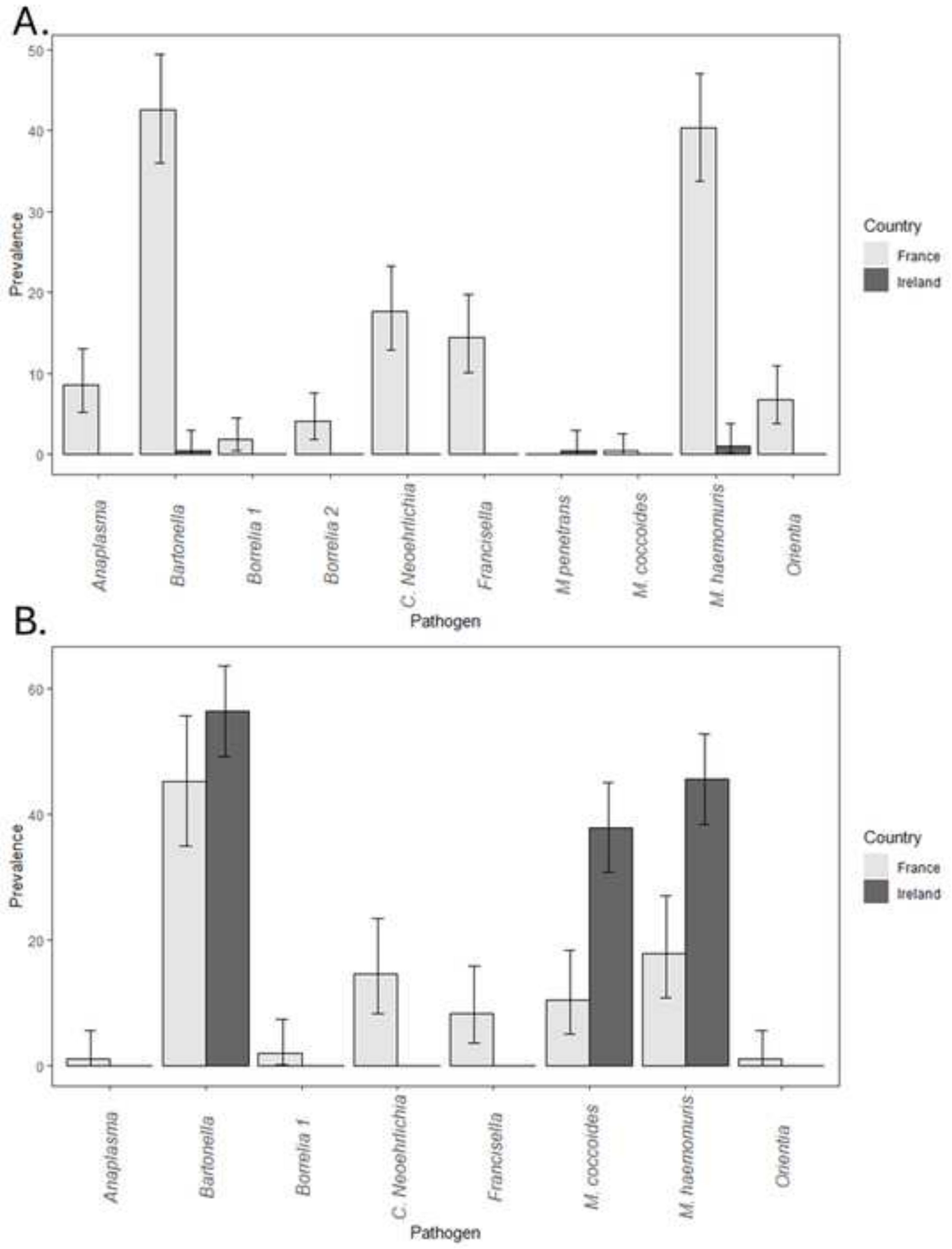

Fig 3

Click here to access/download;Figure;Figure 3..tif

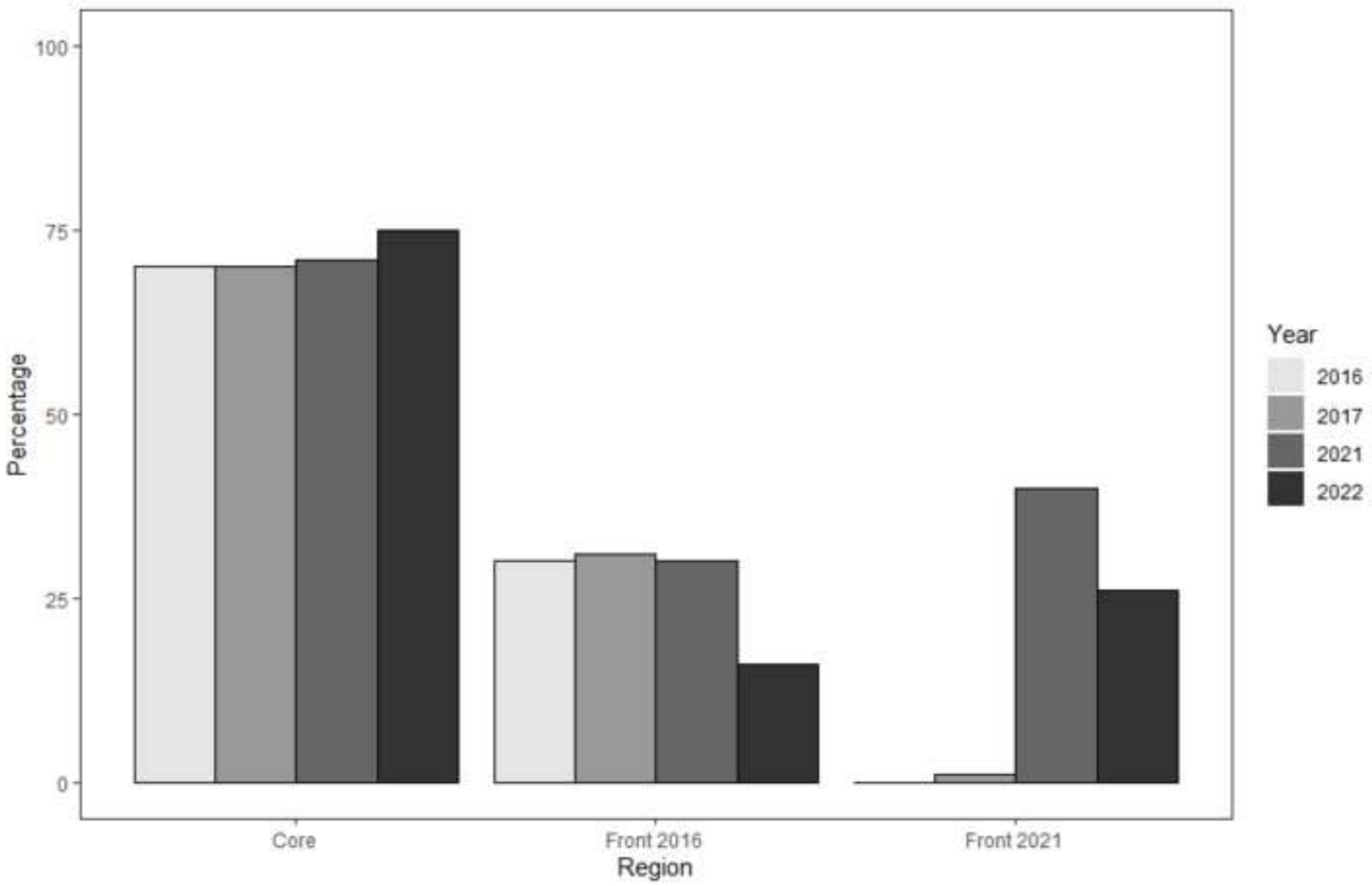

Fig 4

Click here to access/download;Figure;Figure 4..tif

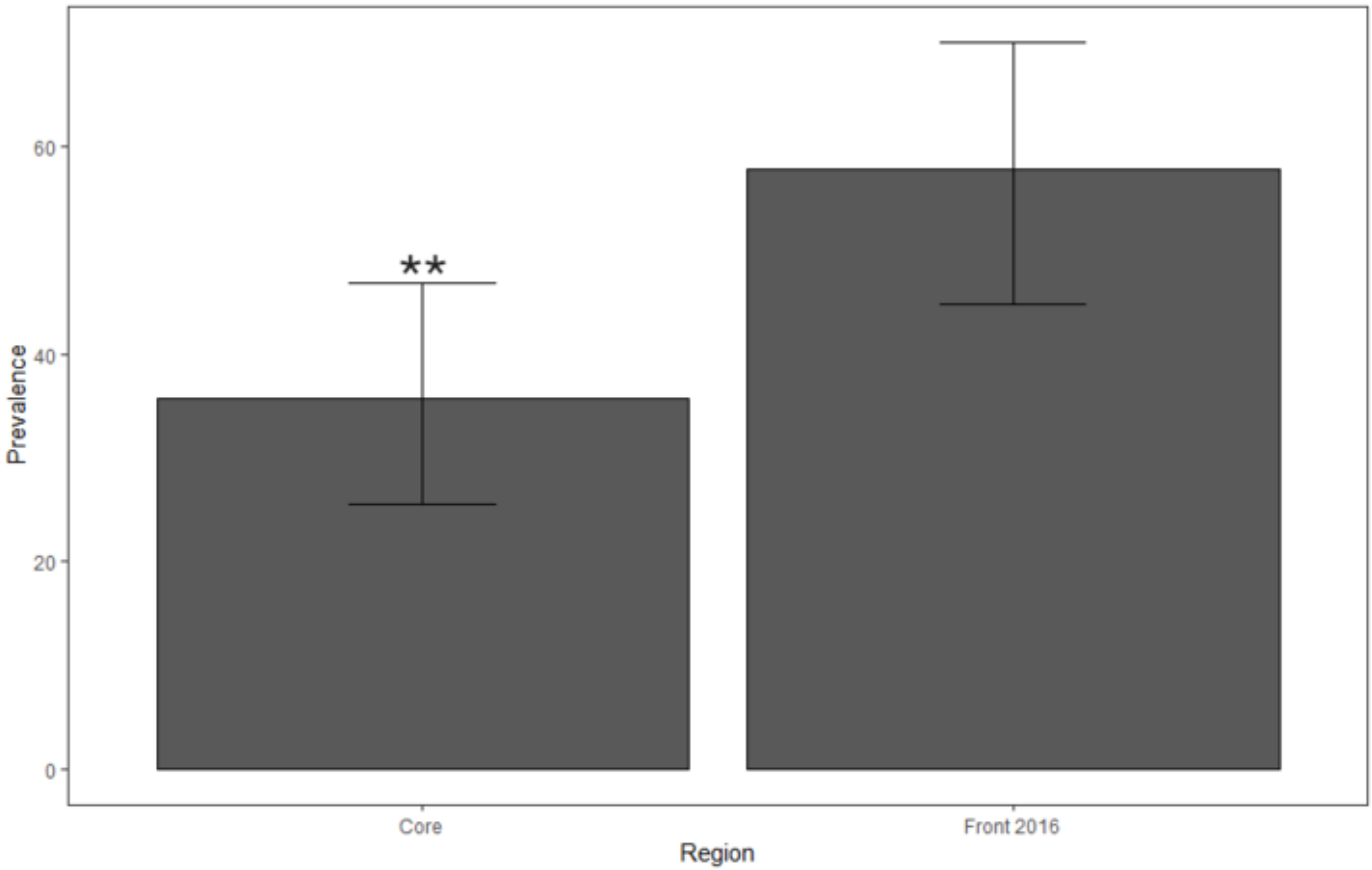

Fig 5

Click here to access/download;Figure;Figure 5..tif

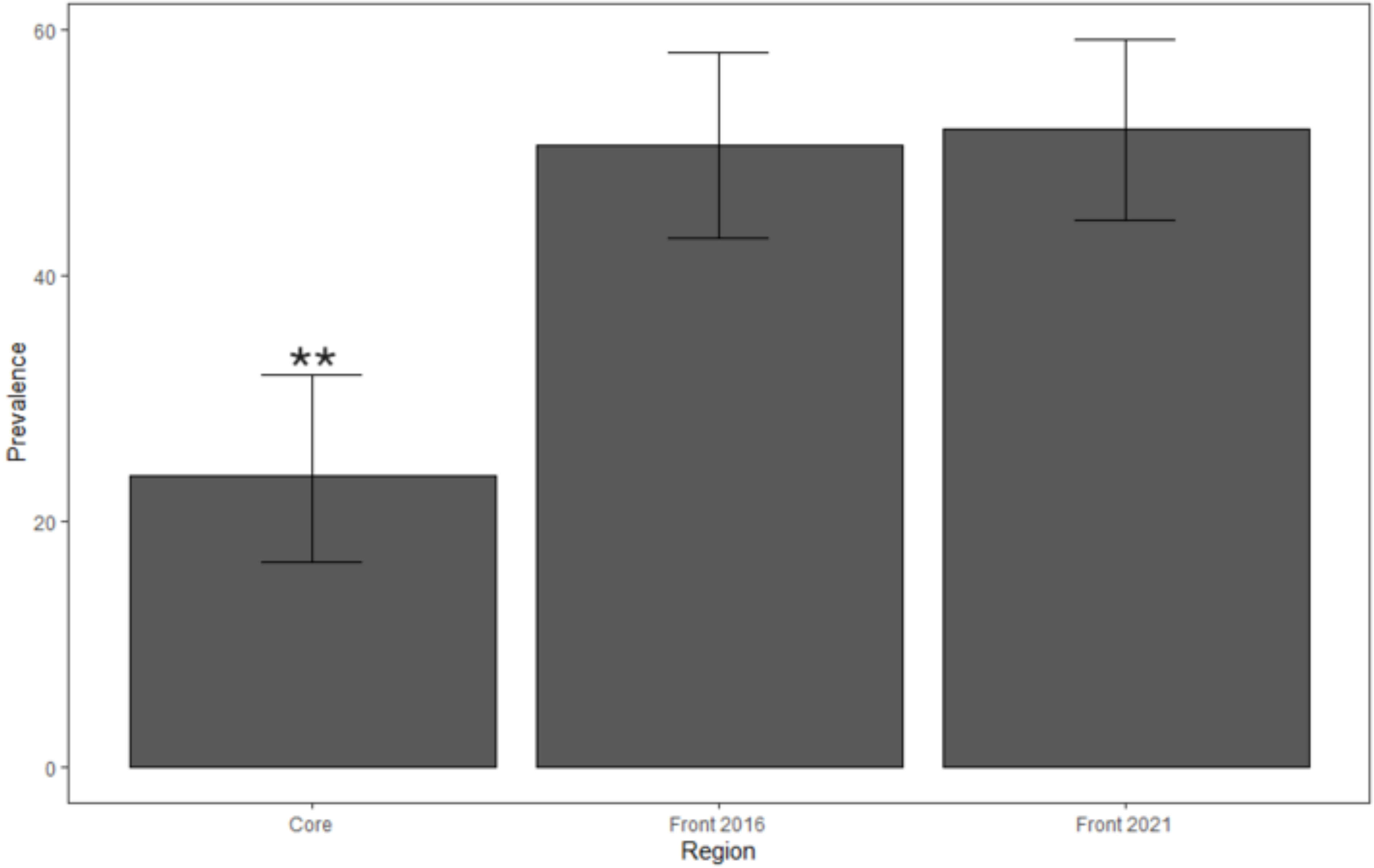

Fig 6

Click here to access/download;Figure;Figure 6..tif

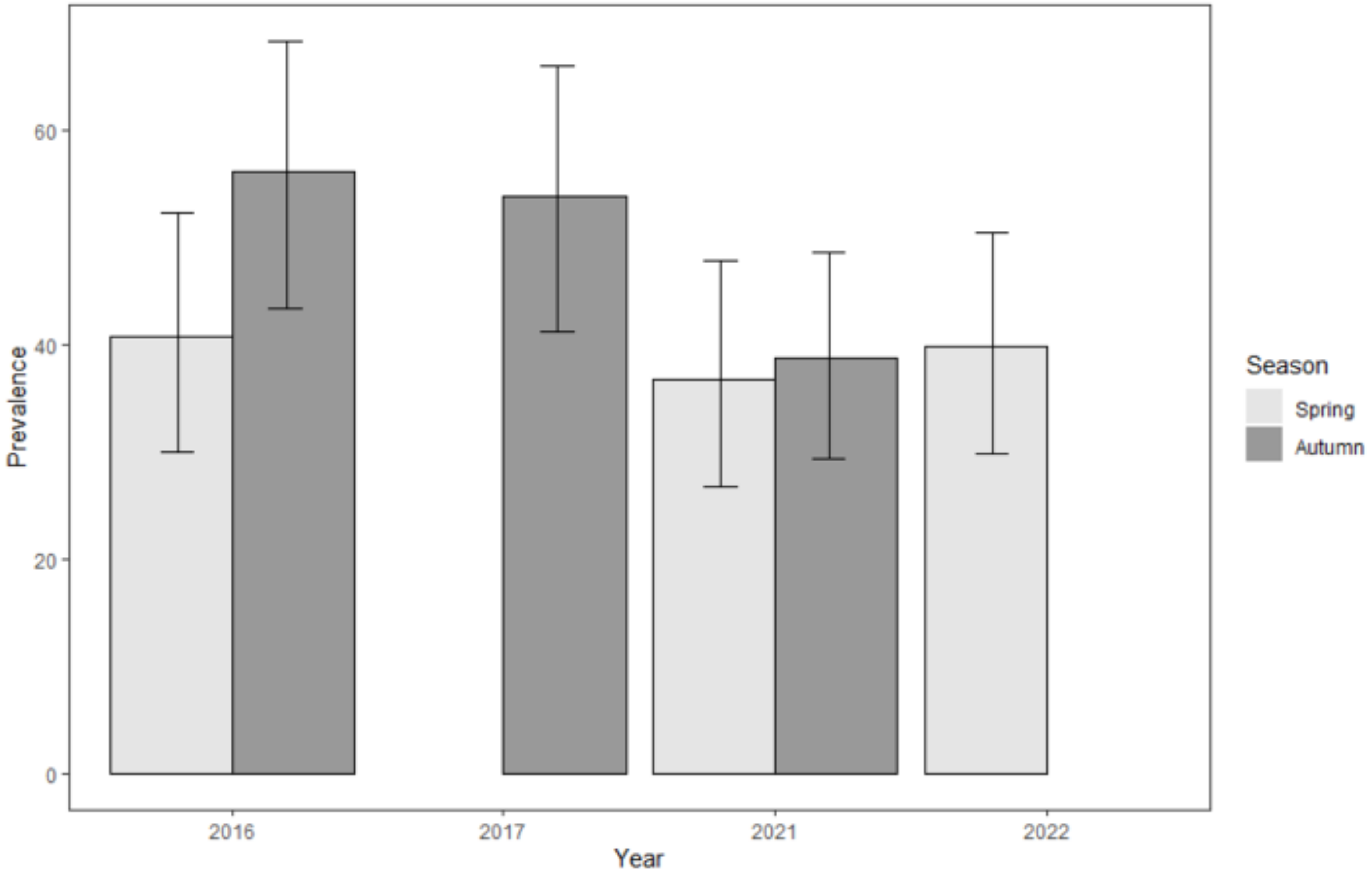

Table S2

Click here to access/download
Supporting Information
Supplementary Table 2 - Prevalence summary region and year.xlsx

Table S1

Click here to access/download
Supporting Information
Supplementary Table 1 - Field work dates.xlsx

Title:

Longitudinal study of a rodent invasion reveals shifts in host pathogen interactions.

Short Title:

Longitudinal study of a rodent invasion reveals shifts in host pathogen interactions.

Author Information:

Andrew McManus[1], Maxime Galan[2], Nathalie Charbonnel[2], Celia Holland[3], Vincent Sluydts[4], Annetta Zintl[5], Peter Stuart[1].

[1] Munster Technological University, Department of Biological and Pharmaceutical Sciences, Tralee, Co. Kerry, Ireland.

[2] CBGP, INRAE, CIRAD, Institut Agro, IRD, Univ Montpellier, Montpellier, France.

[3] Trinity College Dublin, the University of Dublin, Department of Zoology, College Green, Dublin 2, Ireland.

[4] University of Antwerp, Department of Biology, Evolutionary Ecology Group, University of Antwerp, 2610 Wilrijk, Belgium.

[5] University College Dublin, School of Veterinary Medicine, Veterinary Science Centre, Belfield, Dublin 4, Ireland.

Corresponding Author:

Peter Stuart; peter.stuart@mtu.ie

Abstract

Invasive species can disrupt native epidemiological processes, potentially leading to the emergence of zoonotic pathogens. The relatively recent appearance and spread of the bank vole (*Clethrionomys glareolus*) in Ireland provides a unique model system to study these phenomena. The depauperate small mammal community in Ireland, combined with baseline data, allows us to identify the invasion gradient of *C. glareolus* and its effects on rodent-borne pathogens over time. A total of 498 *C. glareolus* and 584 *Apodemus sylvaticus* were sampled, via removal trapping, across nine sites in Ireland in 2016 and 2017, with six sites revisited in 2021 and 2022. 16S rRNA metabarcoding identified 10 putative pathogenic Operational Taxonomic Units (OTUs) present in these rodents, relating to four bacterial taxa, *Bartonella spp., Mycoplasma coccoides, Mycoplasma haemomuris and Mycoplasma penetrans,* and one family of protozoans, Sarcocystidae. Several epidemiological processes were found to be correlated with the *C. glareolus* invasion; firstly,~~,~~ *C. glareolus* in Ireland exhibited patterns consistent with the enemy release hypothesis, compared to native populations in France, and the native *A. sylvaticus* in Ireland.~~ Likewise~~ Secondly, a potential dilution effect, with reduced prevalence of *M. haemomuris* in *A. sylvaticus* at the *C. glareolus* invasion core compared to the invasion front, was observed. Finally, *C. glareolus* had increased prevalence of Sarcocystidae at the invasion front when compared to the invasion core, depending on the time and stage of invasion. These findings further our understanding of pathogen dynamics during biological invasions, demonstrating that invaders affect native host-pathogen communities differently as they advance through various stages of establishment.

Author summary:

When animals are introduced to new environments, they can disrupt local disease patterns by changing how pathogens spread among native wildlife. The spread of the bank vole, a non-native rodent in Ireland, offers a valuable model system for studying how such invasions affect disease dynamics over time. In this study, we examined how the arrival of the bank vole is influencing the health of the native wood mouse. We found that in areas where bank voles have been established longer, wood mice carry fewer infections from a common blood parasite. At the same time, bank voles themselves carried fewer types of pathogens overall than wood mice, possibly giving them an advantage in terms of survival and reproduction. Interestingly, we also observed that bank voles were more likely to carry a specific group of parasites, called Sarcocystidae, in areas they had only recently invaded. These patterns suggest that invading species can reshape disease dynamics in complex and

evolving ways. Understanding how these changes unfold over time is important for predicting future disease risks to wildlife and potentially to humans.~~,~~

Keywords:

Zoonotic pathogens, epidemiology, rodents, enemy release, 16S rRNA metabarcoding, biological invasions

Introduction

The emergence of zoonotic pathogens has seen a sharp increase since the 1940's, with 60% of emerging infectious diseases originating from animals, including the recent outbreaks of SARS-CoV-2, Lassa, Ebola and Mpox viruses (1-7). This increase in the frequency of emerging infectious diseases, which poses a risk to humans, livestock and indigenous wildlife populations, has been primarily attributed to environmental change, caused by landscape change, a reduction of habitat diversity and the introduction of invasive species (8-16).

A recent review by Bezerra-Santos *et al.* (15), highlights that invasive mammal species pose increased risks for public health by introducing zoonotic pathogens (17, 18). However, non-native species can also benefit from a reduction in natural enemies, such as predators or pathogens, compared to conspecifics in their native range (and often compared to the native species), through a ~~an epidemiological~~ process known as "enemy release"(19, 20). In relation to pathogens, ~~T~~this can happen because the pathogen, or the~~ir~~ vector responsible for its transmission, is absent from the founder population, or because the environment in the new range is unsuitable to sustain the pathogen or vector (21-25). This, in turn, may give invaders a competitive advantage over native species. Additionally, invasive species can influence host-pathogen ecological interactions in native hosts in several other ways. Firstly, invasive species can co-invade with new pathogens, which may infect native hosts, leading to a process known as pathogen spillover (26). The establishment of invasive species may also lead to a dilution of native pathogens, where the presence of a host with a lower competence for a specific pathogen can reduce the circulation of that pathogen in competent hosts (27-29). On the other hand, invasive host species can also have an increased competency for native pathogens, causing a spillback of native pathogens that amplifies infection in native hosts (30, 31).

Rodents, the largest order of mammals, make up one of the most common group of invasive species worldwide, affecting native ecosystems, food production and human health (32). Additionally, a recent review by Dharmarajan *et al.* (6), highlighted that rodents and bats harbour the highest proportion of zoonotic agents, identifying these taxa as species of interest for studying zoonoses. Therefore, rodents make excellent models for studying the eco-epidemiology of pathogens due to their presence in large numbers and broad distributions, playing an important role as reservoirs for pathogens causing zoonotic disease; ~~including among others~~such as, orthohantaviruses, *Leptospira* and *Bartonella* species, or *Toxoplasma gondii* (6, 33-37). Finally, studies on rodent invasions have demonstrated the interplay between rodent pathogen interactions and biological invasions, for example, helminth studies in both Ireland and Senegal have observed patterns of enemy release in invasive rodents when comparing sites of long established populations to more recently invaded sites (24, 31, 38, 39).

The case of the bank vole, *Clethrionomys glareolus* (formerly *Myodes glareolus*), invasion in Ireland presents a unique opportunity to explore changes in epidemiological dynamics during a biological invasion (37), ~~. This is~~ particularly due to the depauperate woodland rodent community in Ireland, that includes only the invasive *C. glareolus* and the native woodmouse, *Apodemus sylvaticus* (40). Species depauperate systems provide a valuable scenario to tease out the effects of invasive species on native pathogen communities. For example, in this instance the depauperate rodent community in Ireland has allowed for easier identification of the effects the *C. glareolus* invasion has on the native *A. sylvaticus* population in previous studies (31, 39). Furthermore, *C. glareolus* in Ireland has a single identifiable introduction point in the south west, near Foynes, Co. Limerick, and the likely source population from Germany identified through mitochondrial DNA (41). In addition, studies have shown *C. glareolus* in Ireland to have an expansion rate of 2.23 – 2.63 km per year in unconstrained areas (42). Furthermore, previous studies have shown a reduced relative abundances of *A. sylvaticus* at sites invaded by *C. glareolus* (39, 43). This has allowed for the establishment of cross-sectional studies with an invasion gradient, containing sites from the invasion core (established from the introduction point, where *C. glareolus* exhibits it's highest relative abundance), invasion front (sites where *C. glareolus* had most recently invaded), and uninvaded sites (*A. sylvaticus* only) (39). These factors combined, provide an excellent case study to identify the epidemiological effects that the *C. glareolus* invasion has on native pathogen communities in *A. sylvaticus*, such as spillover, dilution, or spillback.

The development of high-throughput sequencing, and in particular ~~the~~ metabarcoding techniques, facilitates a more holistic approach to studying the rodent pathobiome (44). The ~~resulting~~se methodologies provide a global picture of pathogen diversity within host species, improving ~~should improve~~ our understanding of the

Formatted: Not Highlight

Field Code Changed

Formatted: Not Highlight

relationship~~interlinkages~~ between host-parasite interactions and biological invasions. In the Irish context, rodent helminth parasite communities have been explored previously by Loxton *et al.* (23, 31) and Stuart *et al.* (39). However, ~~but~~ no such data exists on pathogenic microparasites in this model system. ~~Similarly~~Furthermore, there remains a knowledge gap concerning ~~surrounding~~ longitudinal studies focusing on changes in pathogen communities~~s~~ during biological invasions. Such studies are essential for fully exploring changes in host-pathogen interactions, such as pathogen loss in invasive species, as cross-sectional studies only capture a single timepoint of this dynamic process (37, 39, 45-47).

In this study, we aim to address these knowledge gaps by describing~~e~~ the community of microparasites that could be zoonotic or pathogenic for rodents, including bacteria and protozoa, of *C. glareolus* and *A. sylvaticus* in Ireland, through a longitudinal study. We performed a spatiotemporal analysis of this community along the invasion gradient described above, ~~and~~ using rodents ~~sampled~~removed in 2016 from a previous study by Stuart *et al.* (39), with subsequent removal sampling at these sites in 2017, 2021 and 2022. In this study, we use the 16s rRNA amplicon metabarcoding approach developed by Galan *et al*. (48), and ~~used in the recent study~~recently used by Bouilloud *et al.* (49). Focusing on putative pathogenic taxa recorded in samples collected between 2016 and 2022, this present study addresses three main hypotheses: (i) ~~Firstly,~~ whether *C. glareolus* exhibits patterns consistent with the enemy release hypothesis, including a lower pathogen species richness of potentially pathogenic taxa when compared to its native range and the native *A. sylvaticus*;~~,~~ (ii) ~~secondly,~~ whether the presence of *C. glareolus* alters the pathogen communities present in the native *A. sylvaticus* as expected under the dilution or spillback ~~hypotheses~~hypotheses;~~,~~ finally~~,~~ (iii) whether pathogen prevalence changes over time in both rodent species, as the invader becomes established.

Materials & methods

Field work & sample collection

Formatted: Font: 12 pt, Bold

Nine sites were selected for field sampling~~analyses~~ in 2016, as described in Stuart *et al.* (39). These were divied into three regions based on their invasion status; ~~is~~ including~~ed~~ three invasion 'core' sites in Foynes (Foynes 1, 2 & 3), three 2016 invasion front sites (named 'front 2016' herein, i.e. Moore Abbey, Carnpark, and St. John's), and three 2021 invasion front sites (named 'front 2021' herein, i.e. Killinthomas, Loughkey and Donore, uninvaded in 2016) (Figure 1). In 2016, rodents were sampled at all of these nine sites in spring and autumn

trapping sessions and revisited in autumn 2017. For the longitudinal aspect of this study, only six of these sites were revisited in spring and autumn 2021, and spring 2022 (Supplementary table 1). These sites included two of the ‘core’ sites in Foynes (Foynes 1 and 3, not Foynes 2), two invasion ‘front 2016’ sites invasion front in 2016 (‘front 2016’, Moore Abbey and St. John’s wood, not Carnpark) and twohe invasion “front in 2021” sites, (‘front 2021’, Killinthomas and Donore, not Loughkey) (Figure 1).

Longworth traps, baited using peanuts, bird seed mix and a piece of carrot, were placed 5-10m apart in transects of a maximum of 20 traps. A maximum of 24 animals from each species were taken removed from each site per season and year of sampling. The maximum sample size was calculated based on the estimated sample required to observe at least one infected individual based on a prevalence of 12%, and at least 1 success, with 95% confidence intervals (*i.e.* a sample size of 24 should allow detection of all pathogens with a prevalence of 12% and above, allowing detection of rare pathogens, calculated using Sampsize tool available at: https://sampsize.sourceforge.net/iface/index.html#prev). Traps were placed in the field overnight and checked early the next morning. Rodents were inspected in the field and transported back to the laboratory. If insufficient numbers were trapped, the site was revisited for an additional night within the same season.

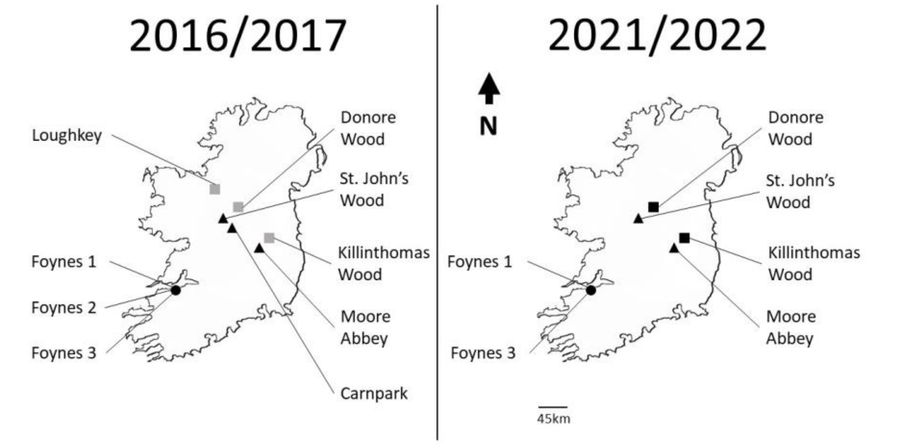

Figure 1. Map of Ireland, showing Irish sampling sites visited in 2016, 2017, 2021 and 2022. Circles indicate the invasion core; triangles indicate the invasion front in 2016 (‘front 2016’) and squares indicate the invasion front in 2021 (‘front 2021’). The grey colour shows sites that were uninvaded by *Clethrionomys glareolus* on a given date, while the black colour shows sites that were invaded.

Laboratory work & analysis

A total of 498 *C. glareolus* and 584 *A. sylvaticus* were sampled across the four years studied. Rodents were euthanised using Isoflurane (1000mg/g, under license from the Health Products Regulatory Authority). The spleens were removed immediately and stored in RNAlater at 4°C for 24 hours, then placed into the freezer at -20°C for further metabarcoding analysis. Rodents were classified into three age categories, juvenile, young adult and mature adult, as described in Behnke *et al.* (50)., These were based on the criteria ofusing rodent length (nose to anus; in millimetres) and weight (grams) as described by Loxton *et al.* (23, 31) and used by Stuart *et al.* (39). For animals found to be at the limit of each classification, visual assessments of maturity were used for final determination, as described by Stuart *et al.* (39).

The 16S metabarcoding analysis was conducted as described by Galan *et al.* (48). DNA was extracted from the rodent spleens using DNeasy 96 Tissue Kit (Qiagen, Germany). This organ was selected by Galan *et al.* (48), as the spleen is responsible for detecting and removing blood-borne microrganisms. Extracted DNA was screened for putative pathogenic Operational Taxonomic Units (OTUs) using the v4 hyper variable region of the 16S rRNA gene with universal primers (16S-V4F 5'-GTGCCAGCMGCCGCGGTAA-3' and 16S-V4R 5'-GGACTACHVGGGTWTCTAATCC-3'), via Illumina MiSeq sequencing. Finally, PCR amplification, sequencing and taxonomic identification were carried out as described by Galan *et al.* (48). From the clusters of microorganism DNA detected, Pputative pathogenic OTUss were filtered and selected based on their potential zoonotic risk and pathogen impact on rodents (51). First, we discarded putative false positive results associated with sequence counts below two OTU-specific thresholds, which checked respectively for putative cross-contamination between samples during DNA extraction and PCR steps on the one hand, and incorrect assignment due to the generation of mixed clusters on the flowcell during Illumina sequencing on the other hand (52). Then, for each sample, and each OTU, we kept only the occurrences confirmed by the two PCR replicates of the same sample. Finally, the putative pathogenic OTUs were selected using their taxonomic affiliations and based on their potential zoonotic risk and pathogen impact on rodents, to separate them from commensal species (51).

Statistical analyses

Formatted: Font: 12 pt, Bold

Formatted: Font: Not Italic

Formatted: Font: Not Italic

Formatted: Font: Not Italic

Formatted: Font: Not Italic

Formatted: Font: Not Italic

Formatted: Font: Not Italic

Formatted: Font: Not Italic

Calculation of prevalence and species richness

Data were analysed using the R version 4.3.1 working under the RStudio software hoods (2023.06.1 Build 524). The packages Plyr (53) and ggplot2 (54) were used to create tables and figures. Separate putative pathogenic OTUs of the same pathogen species were combined into potentially pathogenic taxa for analysis. For each putative pathogenic taxa, prevalence was calculated by the total number of infected individuals divided by the total population sampled. Confidence intervals (maximum and minimum) were calculated to 95% using the R package PropCIs (55). Pathogen species richness was calculated by counting the total number of putative pathogenic taxa recorded in an individual.

Examining patterns of the enemy release hypothesis

Formatted: Font: 12 pt, Bold

Firstly, bacterial data collected from *C. glareolus* and *A. sylvaticus* in Ireland from 2021 was compared to data collected from rodents trapped in forest sites in France, sampled in 2021 by Pradel *et al.* (56), using the same 16S metabarcoding approach (48). These data were examined by comparing prevalences of potentially pathogenic bacterial taxa in rodents from Ireland and France, to examine if less species were detected and at a lower prevalence in Ireland, related to the enemy release hypothesis. However, ~~D~~due to differences in assigning age and maturity to animals in each country, ~~further~~ statistical analysis could not be conducted. Likewise, while the bacterial pathogens were filtered using the same methodology, two extra species of potentially pathogenic Sarcocystidae were included in the Irish data, and consequently meant that these pathogen species could not be included in the comparison.

Examining patterns of infection in Irish rodents

Formatted: Font: 12 pt, Bold

To investigate patterns of infection in each host species in Ireland, pathogens with prevalence above 5% in the overall dataset (all sampling dates and sites) were selected for statistical analysis using generalised linear mixed models (GLMM's) and a binomial distribution (24). The threshold of 5% prevalence was selected as variations below this threshold would be too low for appropriate statistical testing. GLMM's from the package glmmTMB were used (57). GLMMs were selected for these analyses to allow the inclusion of both fixed and random effects in the models, and the appropriate distribution for each type of data being analysed. ~~Pathogen species~~

~~richness was calculated by counting the total number of putative pathogenic taxa recorded in an individual.~~ Due to the depauperate pathogen communities in Ireland, zero-inflated negative binomial distributions were used to investigate patterns in pathogen species richness instead of Poisson distributions used by Diagne *et al.* (24).

Two strategies were used to analyse these data ~~results~~;

1. **The invasion gradient analysis**: only data from 2016 (and all nine sites) were included to test the influence of the invasion gradient on infection patterns in both *A. sylvaticus* and *C. glareolus,* as this year had a very clear "core", "front" (front 2016) and "uninvaded" (front 2021) sites as per the study by Stuart *et al.* (39) (see Figure 1). An additional ~~overall~~ model examining the difference in pathogen species richness between both rodent species in Ireland, and a separate model focusing on *A. sylvaticus* only, was conducted using these data from 2016.

2. **The longitudinal analysis:** data from all four years were used, but only the six sites that were sampled each year were selected, excluding Foynes 2, Carnpark and Loughkey, to keep the data comparable (Table 1). In this approach, time was included in the models as a numerical variable counting years from baseline time, i.e. 2016 = 1.

Formatted: Font: Bold

Formatted: Font: Bold

~~The percentage of *A. sylvaticus* relative to *C. glareolus* was tested against region to justify the classification of these sampling sites in the analyses. For these analyses, the total number *A. sylvaticus* relative to *C. glareolus* trapped in each site in each season within a given year was calculated, generating a percentage of *A. sylvaticus* relative to *C. glareolus,* and assigned to each sample corresponding to trapping site, season and year. The percentage of *A. sylvaticus* relative to *C. glareolus* in the dataset was tested against region using a Kruskal-Wallis test, and a post-hoc Dunn's test, through the dunn.test R package~~ (58)~~, to check differences between regions in both 2016 only and the longitudinal dataset, as the data followed a non-normal distribution.~~

~~Following the justification of the criteria in this factor, GLMMs were used for both analysis strategies.~~ Factors used in each model included season, region (invasion region; core, front 2016, front 2021), sex, age class (juvenile, young adult and mature) as fixed effects, with site included as a random effect variable (Table 1). The longitudinal analysis included the additional fixed effects of time, as years since baseline (2016), and an interaction between season and time. An additional fixed factor, rodent species, was used for the ~~overall~~ model investigating the variance in pathogen species richness between Irish populations of *C. glareolus* and *A. sylvaticus* in 2016. The MuMIn package was used to refine and find the best model based on lowest Akaike information criterion corrected for small sample size (AICc) value (59). The DHARMa package was then used

to check model fit and the performance package to test for multicollinearity and overdispersion (60, 61). The optimisation function, Optim, using the Broyden, Fletcher, Goldfarb and Shanno (BFGS) method was used in cases where model optimisation was needed. Post-hoc tests using Tukey Kramer analysis were used for factors with three or more modalities, while post-hoc Pearson's correlation tests were used to analyse changes in prevalence with time. In ~~the case~~cases where no ~~model~~ GLMM could be fitted, non-parametric tests were used, such as a Wilcoxon Rank Sum test. ~~Post-hoc tests using Tukey Kramer analysis were used for factors with three or more modalities, while post-hoc Pearson's correlation tests were used to analyse changes in prevalence with time.~~

These analyses used the categorical variable of region as a proxy for invasion levels of *C. glareolus* relative to *A. sylvaticus* to aid in the interpretation of the results and increase statistical power through increased number of samples per variable. In order to justify our classification of invasion region in the analysis, the percentage of *A. sylvaticus* relative to *C. glareolus* was tested against invasion region. For these analyses, the total number *A. sylvaticus* relative to *C. glareolus* trapped in each site in each season within a given year was calculated, generating a percentage of *A. sylvaticus* relative to *C. glareolus*. These numbers were then assigned to each sample corresponding to their trapping site, season and year. Finally, to justify the classification of regions, this assigned percentage of *A. sylvaticus* relative to *C. glareolus* in the dataset was tested against region using a Kruskal-Wallis test, and a post-hoc Dunn's test, through the dunn.test R package (58), to check differences between regions in both 2016 only, and the longitudinal dataset, as the data followed a non-normal distribution.

Table 1. Models and data used for the two approaches of this study, the invasion gradient analysis and the longitudinal analysis. Time:Season indicates the interaction that was used between time and season. Time is defined as years since 2016 baseline, with 2016 = 1.

	**Invasion gradient analysis**	**Longitudinal analysis**
**Fixed variables**	Season, Region, Age Class, Sex	Time, Season, Region, Age Class, Sex, Time:Season
**Random variables**	Site	Site
**Years**	2016 only	All (2016, 2017, 2021, 2022)

Sites	All (Figure 1.)	6 repeated sites; Foynes 1 & 3, Killinthomas, Donore, Moore Abbey and St. John's.
Clethrionomys glareolus	n = 148	n = 411
Apodemus sylvaticus	n = 203	n = 500

Results

Pathogens detected in Irish rodent spleen samples

The 16S analyses of 584 *A. sylvaticus* and 498 *C. glareolus* trapped over the four years of sampling in Ireland revealed 10 putative pathogenic OTUs corresponding to five potentially pathogenic taxa (Table 3; Sequencing information, raw sequence reads and prevalence tables are available to the following link: https://zenodo.org/records/14883529). These included one OTU of the genus *Bartonella*, a bacterial genus potentially including zoonotic species (e.g. *B. henselae*) (62), three OTUs of *Mycoplasma haemomuris* (combined for analysis), one OTU of *Mycoplasma coccoides* and one OTU of *Mycoplasma penetrans* (known to be zoonotic). These three *Mycoplasma* species have traditionally been associated with pathology in rodents (63-65). While more recent studies suggest that *Haemoplasma* may be largely subclinical in wildlife they have also highlighted the need for further studies increasing their geographic range and elucidating transmission (66). Finally, we detected four OTUs of Sarcocystidae (combined for analysis, a family of protozoans, with some species that are zoonotic, and others that are pathogenic for rodents) (67, 68). All five taxa were detected in *C. glareolus*, all of which had a 1% prevalence or below, except for Sarcocystidae, which had a prevalence of 37.15%. All potentially pathogenic taxa were also found in *A. sylvaticus* (Table 2; the prevalence for each region per year available in Supplementary Table 2), except *M. penetrans*. However, it should be noted that only one individual *C. glareolus* was found to be positive for this pathogen out of 498 tested.

Formatted: Font: (Default) Times New Roman

Table 2. Prevalence (%) of potentially pathogenic taxa in all *Apodemus sylvaticus* and *Clethrionomys glareolus* sampled in Ireland from 2016 - 2022. The 95% confidence interval (maximum and minimum value) is detailed in brackets.

**Pathogen**	**Transmission**	***Apodemus sylvaticus*** ***n = 584***	***Clethrionomys glareolus*** ***n = 498***
Bartonella	Vector (flea)	51.54 (47.40 - 55.66)	0.2 (0.01 - 1.11)
Mycoplasma haemomuris	Vector (lice)	44.01 (39.93 - 48.14)	1 (0.33 - 2.33)
M. coccoides	Vector (lice)	44.18 (40.10 - 48.31)	0.2 (0.01 - 1.11)
M. penetrans	Sexual	0.00	0.2 (0.01 - 1.11)
Sarcocystidae	Dependent on species, *i.e.* Environmental (Direct life cycle), or intermediate host (indirect life cycle) in some cases.	1.71 (0.82 - 3.13)	37.15 (32.89 - 41.56)

Mycoplasma transmission has been linked to ectoparasites such as lice, based on early observations and PCR detection in arthropod vectors, although more experimental evidence for biological transmission is required (69).

Formatted: Font: Not Bold, Italic, English (Ireland)

Formatted: Font: Not Bold, English (Ireland)

Formatted: Font: Not Bold, English (Ireland)

Formatted: Font: Not Bold, English (Ireland)

Table 3. Summary of significant factors in pathogen species richness and pathogens prevalence detected.

	**Statistically significant factors observed in invasion gradient analysis (2016)**	**Statistically significant factors observed in longitudinal analysis (2016 - 2022)**
**Both Rodent Species**		
**Species Richness**	Species (Voles)***	NA
Apodemus sylvaticus	**n = 203**	**n = 500**
**Species Richness**	Sex (M)*	NA
Bartonella	Season (Spring)**, Sex (M)*	**Full:** Season(Spring)***, Sex(M)*<br>**Sp:** Time*
Mycoplasma haemomuris	Age class (Mature)***	Season(Spring)**, Region(Front 2016)**, Region(Front2021)**, Age class(Juvenile)***, Age class (Mature)*
Mycoplasma coccoides	Age class (Mature)*	Time*, Age class(Mature)**
Clethrionomys glareolus	**n = 148**	**n = 411**
**Sarcocystidae**	Region (Front 2016)**	**Full:** Time***, Season(Spring)***, Region(Front 2021)*, Sex(M)*, Age class(Mature)*<br>**Sp**: Region(Front 2016)*, Region(Front 2021)***, Age class(Mature)*<br>**Au:** Time***, Sex(M)*

Full = full model including all years and seasons. In some instances, per season models were ran due to multicollinearity, this is indicated by Sp = spring only model, Au = autumn only model. Time indicates years as a numerical value since baseline, with 2016 = 1. n represents sample sizes in the full model for each analysis. Statistically significant factors include the associated variable (level) in brackets. Asterisks indicate level of significance, with * = $p < 0.5$, ** = $p < 0.01$, and *** = $p < 0.001$.

Patterns of infection relating to the enemy release hypothesis

Based on data from 2021, *C. glareolus* in Ireland (n = 189) exhibited fewer potentially pathogenic bacterial taxa than *C. glareolus* sampled in forest sites in France (n=221). Most notably, *Anaplasma, Borrelia, Candidatus Neoehrlichia, Francisella and Orientia* were absent from the Irish samples. In addition, lower prevalences of *Bartonella* and *M. haemomuris* were also observed in the Irish *C. glareolus* specimens (Figure 2a). Moreover, *A. sylvaticus* individuals trapped in Ireland (*n* = 193) had a similar prevalence of *Bartonella* compared to forest site populations in France in 2021 (*n* = 95), but higher prevalences of *M. haemomuris and M. coccoides*. As above,

Formatted Table

Formatted: Font: Bold

Formatted: Font: Bold

Formatted: Font: Bold

Formatted: Font: Bold

Formatted: Font: Not Italic

Formatted: Font: 12 pt

Formatted: Font: 12 pt, Bold

Anaplasma, Borrelia, Candidatus Neoehrlichia, Francisella and Orientia were not detected in the Irish *A. sylvaticus* samples (Figure 2b).

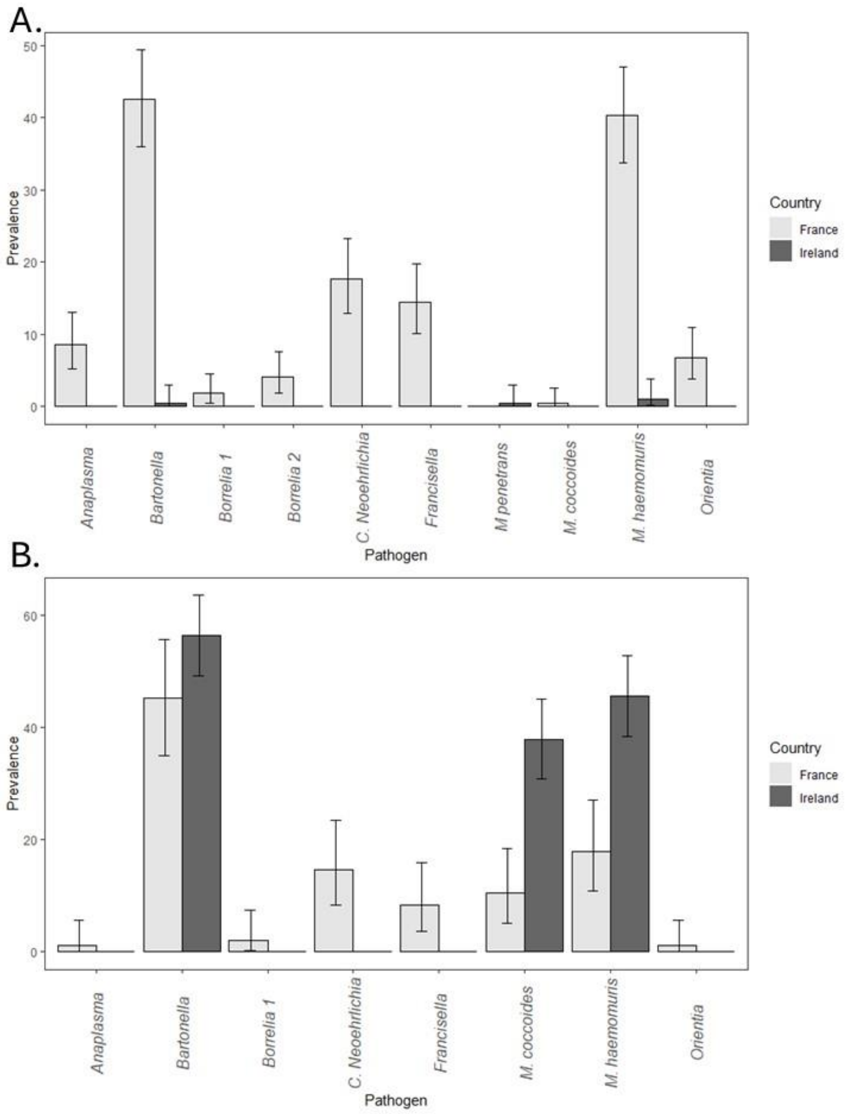

Figure 2. Prevalence of potentially pathogenic bacterial taxa observed in 2021 in both France (*Clethrionomys glareolus* native range, light grey) and Ireland (*C. glareolus* invasive range, dark grey) in **A.** *C. glareolus* and **B.**

Apodemus sylvaticus. Error bars represent 95% confidence intervals. *C. Neoehrlichia* represents *Candidatus Neoehrlichia.*

Validation of invasion region criteria for statistical models &~~and~~ progress of *Clethrionomys glareolus* invasion

In the 2016 data for the invasion gradient analysis, the percentage of *A. sylvaticus* relative to *C. glareolus* was directly correlated with the region of invasion. The core had the lowest percentage of *A. sylvaticus* relative to *C. glareolus*, followed by front 2016, and finally front 2021 was completely uninvaded, with 100% *A. sylvaticus* (Kruskal Wallis, $chi^2 = 271.99$, df = 2, $p < 0.001$; Dunn's test post-hoc analysis, All regions, $p < 0.001$) (Figure 3~~2~~). Our capture data thus confirms the invasion categories initially designed.

In the longitudinal data, the core had a lower percentage of *A. sylvaticus* relative to *C. glareolus* when compared to both invasion front regions, front 2016 and front 2021. However, the front 2021 was rapidly invaded in 2021 and 2022 and consequently no difference was observed between the percentage of *A. sylvaticus* in front 2016 and front 2021 (Kruskal Wallis, $chi^2 = 606.57$, df = 2, $p < 0.001$; Dunn's test post-hoc analysis, Core – Front 2016, $p < 0.001$; Core – Front 2021, $p < 0.001$; Front 2016 – Front 2021, *p* =0.1039~~> 0.05~~) (Figure 3~~2~~).

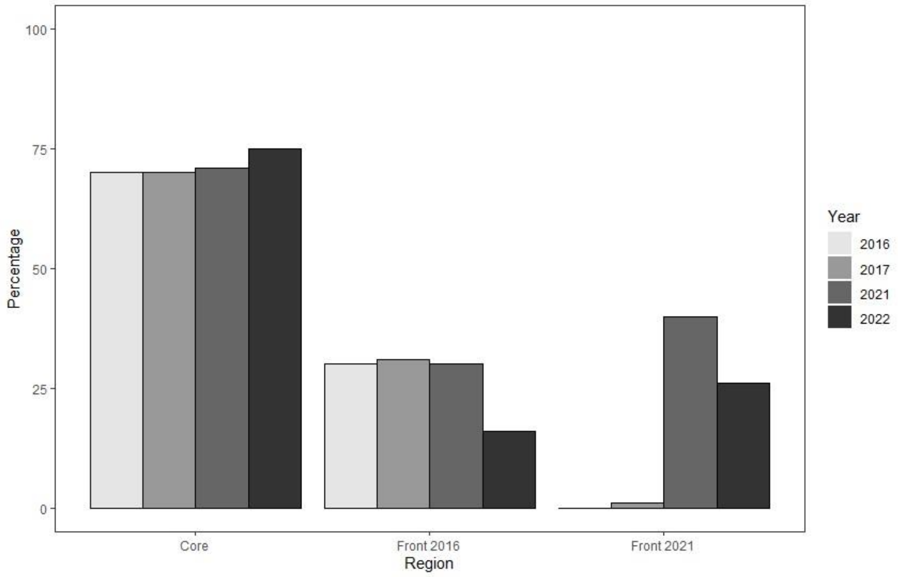

Figure 32. Percentage *Clethrionomys glareolus* relative to *Apodemus sylvaticus* in each region for each year. Data for 2016 and 2017 includes all 9 sites.

Pathogens detected in Irish rodent spleen samples

The 16S analyses of 584 *A. sylvaticus* and 498 *C. glareolus* trapped over the four years of sampling in Ireland revealed 10 putative pathogenic OTUs corresponding to five potentially pathogenic taxa (Table 3; Sequencing information, raw sequence reads and prevalence tables are available to the following link: https://zenodo.org/records/14883529). These included one OTU of the genus *Bartonella*, a bacterial genus potentially including zoonotic species (e.g. *B. henselae*), three OTUs of *Mycoplasma haemomuris* (combined for analysis, and known to be pathogenic for rodents), one OTU of *Mycoplasma coccoides* (known to be pathogenic for rodents), one OTU of *Mycoplasma penetrans* (known to be zoonotic), and four OTUs of Sarcocystidae (combined for analysis, a family of protozoans, with some species that are zoonotic, and others that are pathogenic for rodents). All five taxa were detected in *C. glareolus*, all of which had a 1% prevalence or below, except for Sarcocystidae, which had a prevalence of 37.15%. All potentially pathogenic taxa were also found in *A. sylvaticus* (Table 2), except *M. penetrans*. However, it should be noted that only one individual *C. glareolus*

Formatted: Font: (Default) Times New Roman

was found to be positive for this pathogen out of 498 tested.

Table 2. Prevalence (%) of potentially pathogenic taxa in all *Apodemus sylvaticus* and *Clethrionomys glareolus* sampled in Ireland from 2016 - 2022. The 95% confidence interval (maximum and minimum value) is detailed in brackets.

-	**Transmission**	***Apodemus sylvaticus***	***Clethrionomys glareolus***
**Pathogen**		***n* = 584**	***n* = 498**
Bartonella	Vector (flea)	51.54 (47.40 - 55.66)	0.2 (0.01 - 1.11)
Mycoplasma haemomuris	Vector (lice)	44.01 (39.93 - 48.14)	1 (0.33 - 2.33)
M. coccoides	Vector (lice)	44.18 (40.10 - 48.31)	0.2 (0.01 - 1.11)
M. penetrans	Sexual	0.00	0.2 (0.01 - 1.11)
Sarcocystidae	Dependent on species, *i.e.* Environmental (Direct life cycle), or intermediate host (indirect life cycle) in some cases.	1.71 (0.82 - 3.13)	37.15 (32.89 - 41.56)

Formatted: Line spacing: Multiple 1.08 li

Table 3. Summary of significant factors in pathogen species richness and pathogens prevalence detected.

	**Statistically significant factors observed in invasion gradient analysis (2016)**	**Statistically significant factors observed in longitudinal analysis (2016 - 2022)**
**Both Rodent Species**		
**Species Richness**	Species (Voles)***	NA
Apodemus sylvaticus		
**Species Richness**	Sex (M)*	NA
Bartonella	Season (Spring)**, Sex (M)*	**Full:** Season(Spring)***, Sex(M)*<br>**Sp:** Time*
Mycoplasma haemomuris	Age class (Mature)***	Season(Spring)**, Region(Front 2016)**, Region(Front2021)**, Age class(Juvenile)***, Age class (Mature)*
Mycoplasma coccoides	Age class (Mature)*	Time*, Age class(Mature)**
Clethrionomys glareolus		
**Sarcocystidae**	Region (Front 2016)**	**Full:** Time***, Season(Spring)***, Region(Front 2021)*, Sex(M)*, Age class(Mature)*<br>**Sp:** Region(Front 2016)*, Region(Front 2021)***, Age class(Mature)*<br>**Au:** Time***, Sex(M)*

Full = full model including all years and seasons. In some instances, per season models were ran due to multicollinearity, this is indicated by Sp = spring only model, Au = autumn only model. Time indicates years as a numerical value since baseline, with 2016 = 1. Statistically significant factors include variables in brackets. Asterisks indicate level of significance, with * = $p < 0.5$, ** = $p < 0.01$, and *** = $p < 0.001$.

Based on data from 2021, *C. glareolus* in Ireland (n = 189) exhibited fewer potentially pathogenic bacterial taxa than *C. glareolus* sampled in forest sites in France (n=221). Most notably, *Anaplasma, Borrelia, Candidatus Neoehrlichia, Francisella and Orientia* were absent from the Irish samples. In addition, lower prevalences of *Bartonella* and *M. haemomuris* were also observed in the Irish *C. glareolus* specimens (Figure 3a). Moreover, *A. sylvaticus* individuals trapped in Ireland (*n* = 193) had a similar prevalence of *Bartonella* compared to forest site populations in France in 2021 (*n* = 95), but higher prevalences of *M. haemomuris and M. coccoides*. As above,

Anaplasma, Borrelia, Candidatus Neoehrlichia, Francisella and Orientia were not detected in the Irish *A. sylvaticus* samples (Figure 3b).

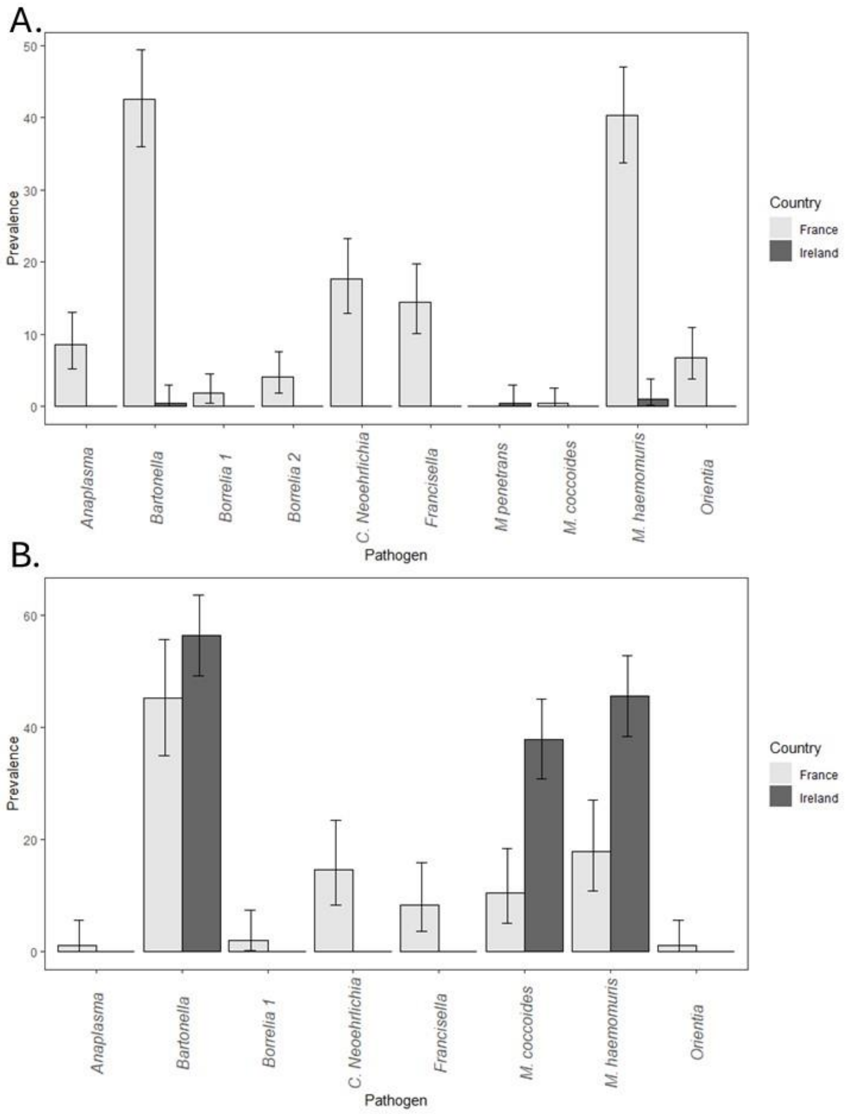

Figure 3. Prevalence of potentially pathogenic bacterial taxa observed in 2021 in both France (*Clethrionomys glareolus* native range, light grey) and Ireland (*C. glareolus* invasive range, dark grey) in **A.** *C. glareolus* and **B.**

Apodemus sylvaticus. Error bars represent 95% confidence intervals (Maximum and Minimum). *C. Neoehrlichia* represents *Candidatus Neoehrlichia*.

Invasion gradient analysis:

Formatted: Font: 12 pt

Formatted: Font: 12 pt, Bold

Pathogen richness

The best model (lowest AICc value; 813.979) explaining variations in the species richness of potentially pathogenic taxa between *C. glareolus* and *A. sylvaticus* in 2016 included only rodent species (Table 3). *C. glareolus* ($\bar{x}$ = 0.46) was shown to have a lower mean number of pathogen species compared to *A. sylvaticus* ($\bar{x}$ = 1.37) (GLMM, family = Zero inflated negative binomial, z = -8.068, p < 0.001). However, poor model fit was still observed when examining residuals, hence nonparametric tests were carried out to confirm these findings. Using a Wilcoxon Rank Sum test, a significant difference in species richness between *C. glareolus* and *A. sylvaticus* was confirmed, with the invasive *C. glareolus* (med = 0) having a lower median number of pathogens compared to *A. sylvaticus* (med = 1) (W = 23262, p < 0.001).

Formatted: Not Highlight

When focusing on *A.* sylvaticus only, using the invasion gradient analysis, no effect for the *C. glareolus* invasion was observed on the species richness of potentially pathogenic taxa. The best model (lowest AICc value; 565.089) included only sex (Table 3). Sex was shown to be statistically significant with males ($\bar{x}$ = 1.54) having a higher mean pathogen species richness compared to females ($\bar{x}$ = 1.14) (GLMM, family = Zero inflated negative binomial, z = 2.391, p = 0.017). As before, due to poor model fit, these findings were confirmed using a Wilcoxon Rank Sum test, showing males (med = 2) have a higher median species richness compared to females (med = 1) (W = 3830.5, p < 0.01).

Apodemus sylvaticus

The patterns of infections observed in the invasion gradient and longitudinal analyses for the pathogen species *Bartonella, M. haemomuris* and *M. coccoides* were examined in *A. sylvaticus* sampled in Ireland.

Bartonella

Considering the invasion gradient analysis, the best model (lowest AICc value; 271.876) included season and sex (Table 3). *Bartonella* prevalences were higher in autumn compared to spring (GLMM, family = Binomial, z = -2.741, p = 0.00612), while males were more infected than females (GLMM, family = Binomial, z = 2.404, p < 0.05).

In the longitudinal analysis, the best model (lowest AICc value) included time, season, region, and sex, with an interaction between time and season. The interaction between time and season was shown to have a high variance inflation factor (VIF), meaning the covariates were correlated and potentially affecting the model through multicollinearity. To account for this, the interaction was removed in the overall model, and separate GLMM's were conducted for each season. In the overall model, no effect for time or region was observed (p > 0.05). *A. sylvaticus* in autumn showed higher prevalences of *Bartonella* than in spring (GLMM, family = Binomial, z = -3.708, p < 0.001). Alongside this, males exhibited higher prevalences compared to females (GLMM, family = Binomial, z = 2.241, p < 0.05).

In the seasonal models, the best model (lowest AICc value) for spring included time and sex. No effect for region was observed. Prevalence was positively correlated (0.15) with time (GLMM, family = Binomial, z = 2.344, p = 0.019; Pearsons's correlation test, t = 2.4751, p < 0.05). Sex did not significantly influence the prevalence of *Bartonella* in *A. sylvaticus* in the spring only model (p > 0.05). In the autumn only analysis, no factor significantly influenced *Bartonella* prevalence.

Mycoplasma haemomuris

Considering the invasion gradient analysis, the best model (lowest AICc value; 268.721) included only age class (Table 3). No effect for region was observed. Age class was statistically significant (Age class: Mature, GLMM, family = Binomial, z = 3.405, p < 0.001). Post-hoc analysis showed that mature adults had significantly higher prevalences compared to young adults and juveniles. No difference between young adults and juveniles was observed, possibly due to the low number of juveniles in the dataset (n = 9) (Tukey Kramer analysis; Mature – Young adults, p < 0.01; Mature – Juveniles, p < 0.05; Young adults – Juveniles, p => 0.06255).

In the longitudinal analysis, no effect for time was observed, and the best model selected (lowest AICc value) included the factors season, region, and age class. Region was shown to have a significant effect on the

prevalence of *M. haemomuris* (Region: Front 2016, GLMM, family = Binomial, *z* = 2.744, $p < 0.01$; Region: Front 2021, GLMM, family = Binomial, *z* = 2.874, $p < 0.01$). Post-hoc analysis demonstrated that the core had a lower prevalence of *M. haemomuris* compared to front 2016 and front 2021, with no difference observed between front 2016 and front 2021 (Figure 4) (Tukey Kramer analysis; Core – Front 2016, $p < 0.001$; Core – Front 2021, $p < 0.001$; Front 2016 – Front 2021, $p > 0.05$). Secondly, spring had higher prevalences compared to autumn (GLMM, family = Binomial, *z* = 2.987, $p < 0.01$). Finally, age class was shown to be a statistically significant factor (Age class: Juvenile, GLMM, family = Binomial, *z* = -3.414, $p < 0.001$; Age class: Mature, GLMM, family = Binomial, *z* = 2.146, $p < 0.05$). Post-hoc analysis showed mature adults were the most infected, followed by subadults, with juveniles least infected (Tukey Kramer analysis; Mature - Young adults, $p < 0.01$; Mature – Juveniles, $p < 0.001$; Juveniles – Young adults, $p < 0.001$).

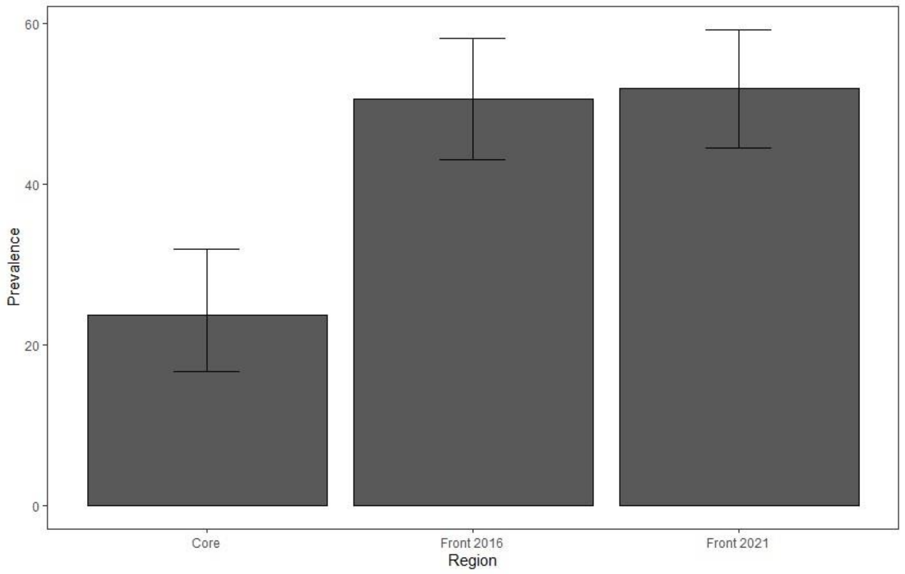

Figure 4. Prevalence of *Mycoplasma haemomuris* in *Apodemus sylvaticus* per region in the longitudinal analysis (all years). Error bars represent 95% confidence intervals (Maximum and Minimum).

Mycoplasma coccoides

For the invasion gradient analysis, the best model (lowest AICc value from MuMIn; 285.4) included only the

null model, therefore the second lowest AICc model (285.486) was used, including only age class and sex (Table 3). Age class was the only statistically significant variable (Age class: Mature, GLMM, family = Binomial, $z = -2.115$, $p < 0.05$). However, during post-hoc analysis no statistical differences was observed between pairs of age class (Tukey Kramer analysis; Mature – Young adults, $p = 0.24$; Mature – Juveniles, $p = 0.92$; Young adults – Juveniles, $p = 0.89$). ~~(Tukey Kramer analysis, $p > 0.05$).~~ No effect of the *C. glareolus* invasion gradient on *M. coccoides* prevalence was observed, as region was excluded from the lowest AICc model.

~~The longitudinal analysis showed that the best model (lowest AICc value) included time, sex and age class. Time was shown to be a significant factor (GLMM, family = Binomial, $z = -2.175$, $p < 0.05$), with a negative correlation (-0.1) between prevalence and time (years) (Pearson's correlation test, t=-2.2929, p < 0.05). This can be observed in Figure 5, where a reduction in prevalence is seen over time. Age class was also shown to affect prevalence (Age class: Mature, GLMM, family = Binomial, $z = -3.04$, $p < 0.01$). Post-hoc analysis demonstrated young adults being more infected than mature adults, however, no statistical differences were observed between mature adults and juveniles and between juveniles and young adults (Tukey Kramer analysis; Young adults – Mature, $p < 0.05$; Mature – Juveniles, $p > 0.05$; Young adults – Juveniles, $p > 0.05$).~~

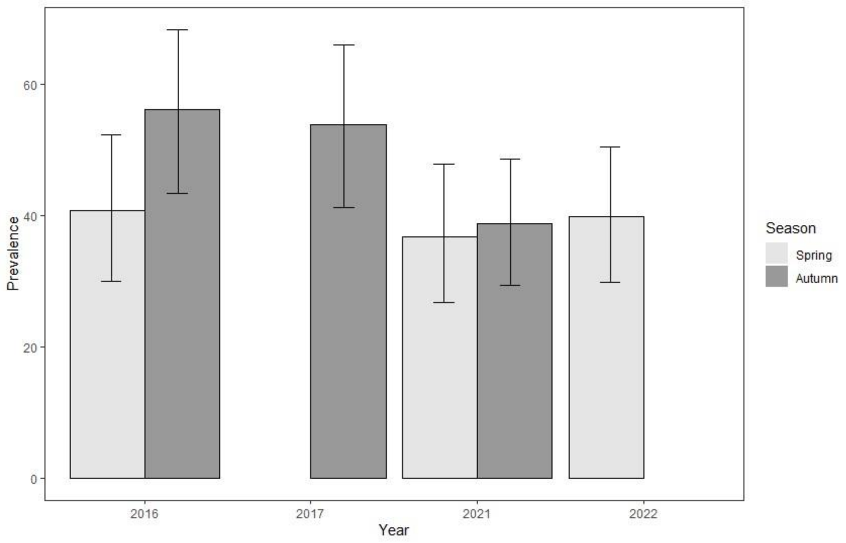

~~**Figure 5.** Prevalence of *Mycoplasma coccoides* in *Apodemus sylvaticus* for each season and year sampled. Error bars represent 95% confidence intervals (Maximum and Minimum).~~

Clethrionomys glareolus

Regarding *C. glareolus* in Ireland, the patterns of infection with Sarcocystidae observed during the invasion gradient ~~and longitudinal analyses~~ were examined.

Sarcocystidae

Considering the invasion gradient analysis on the prevalence of Sarcocystidae in *C. glareolus,* the best model (lowest AICc value; 202.816) included only region (Table 3). The invasion front (front 2016) showed a significantly higher prevalence when compared to the core (Region: Front 2016, GLMM, family = Binomial, z = 2.652, p < 0.01) (Figure 4~~6~~). Similarly, the same invasion gradient analysis was performed on a single OTU of Sarcocystidae specific to *C. glareolus*. The best model (lowest AICc value; 201.422) included region and age class. Region showed the same pattern as the analysis on all Sarcocystidae, with higher prevalence of this Sarcocystidae OTU in the front 2016 compared to the core (Region: Front 2016, GLMM, family = Binomial, z =

2.187, $p < 0.05$). Age class was not statistically significant (Age class: Mature, GLMM, family = Binomial, z = -1;871, $p = 0.0613$; Age class: Juvenile, GLMM, family = Binomial, z = 0.453, p = 0.65~~$p > 0.05$~~).

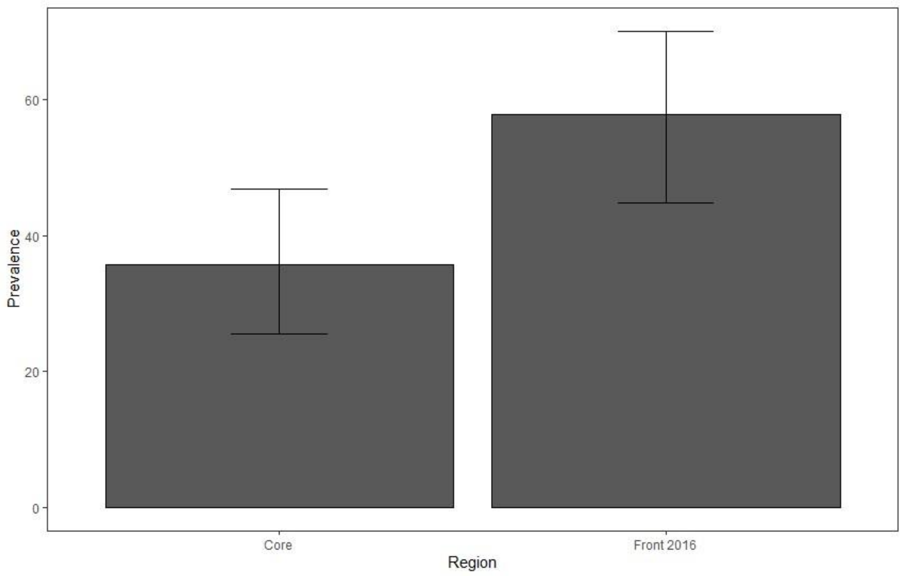

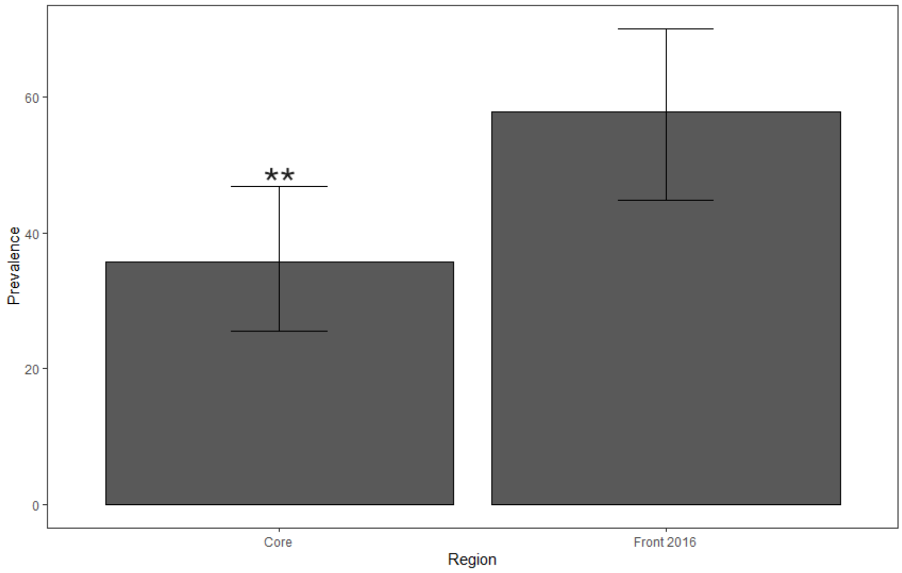

Figure 46. Prevalence of Sarcocystidae in *Clethrionomys glareolus* per region in 2016. Error bars represent 95% confidence intervals (Minimum and Maximum). Asterisks indicate level of statistical significant (** = p <0.01)

The longitudinal analysis of the prevalence of Sarcocystidae in *C. glareolus* found the best model (lowest AICc value) included time, season, region, sex, age class and an interaction between time and season. A high VIF was observed for the interaction and hence it was removed from the final model. Time was shown to be statistically significant (GLMM, family = Binomial, z = -4.849, p < 0.001). Post-hoc analysis found that prevalence is negatively correlated (-0.22) with time (years since baseline) (Pearson's correlation test; t = -4.6122, p < 0.001, Figure 7). Higher prevalence was observed in spring compared to autumn (GLMM, family = Binomial, z = 3.547, p < 0.001). Region was shown to be statistically significant (Region: Front 2021, GLMM, family = Binomial, z = 2.143, p < 0.05). Post-hoc analysis demonstrates that the core had a lower prevalence compared to front 2016 but not statistically different from front 2021, while no statistical difference was observed between front 2016 and front 2021 (Tukey Kramer analysis; Core – Front 2016, p < 0.05; Core – Front 2021, p > 0.05; Front 2016 – Front 2021, p > 0.05). Females had higher prevalences than males (GLMM, family = Binomial, z = -2.216, p < 0.05). Finally, age class was shown to be a statistically significant factor (Age class: Mature, GLMM, family = Binomial, z = -2.322, p < 0.05). Post-hoc analysis showed that juveniles were more infected than mature adults. No statistical difference was observed between mature adults and young adults, or young adults and juveniles (Tukey Kramer analysis; Juveniles – Mature, p < 0.01; Juveniles – Young adults, p > 0.05; Mature – Young adults, p > 0.05).

Separate binomial GLMMs for each season were run on the longitudinal models to account for the removed interaction between season and time. In spring, the best model (lowest AICc value) included region and age class. Region was shown to be statistically significant (Region: Front 2016, GLMM, family = Binomial, z = 2.188, p < 0.05; Region: Front 2021, GLMM, family = Binomial, z = 3.769, p < 0.001). Post-hoc analysis demonstrated that the core had a lower prevalence when compared to both front 2016 and front 2021, while no difference was observed in the post hoc analysis between the two front regions identified in 2016 and 2021 (Tukey Kramer analysis; Core – Front 2016 p < 0.01; Core – Front 2021 p < 0.001; Front 2016 – Front 2021, p > 0.05). Age class was also statistically significant (Age class: Mature, GLMM, family = Binomial, z = -2.112, p < 0.05). Post-hoc analysis showed juveniles and young adults more likely to be infected compared to mature

adults, but no statistical difference was observed between juveniles and young adults (Tukey Kramer analysis; Mature – Juveniles, $p < 0.01$; Mature – Young adults, $p < 0.05$; Young adults – Juveniles, $p > 0.05$).

Finally, the best model (lowest AICc value) for autumn included time, sex, and age class. No effect for region was observed. Time was shown to be statistically significant (GLMM, family = Binomial, $z = -6.766$, $p < 0.001$) with time being negatively correlated (-0.5) with prevalence (Pearsons's correlation test, $t = -8.5352$, $p < 0.001$, Figure 7). Females had higher prevalences compared to males (GLMM, family = Binomial, $z = -2.231$, $p < 0.05$). Age class did not significantly influence the prevalence of Sarcocystidae in this analysis.

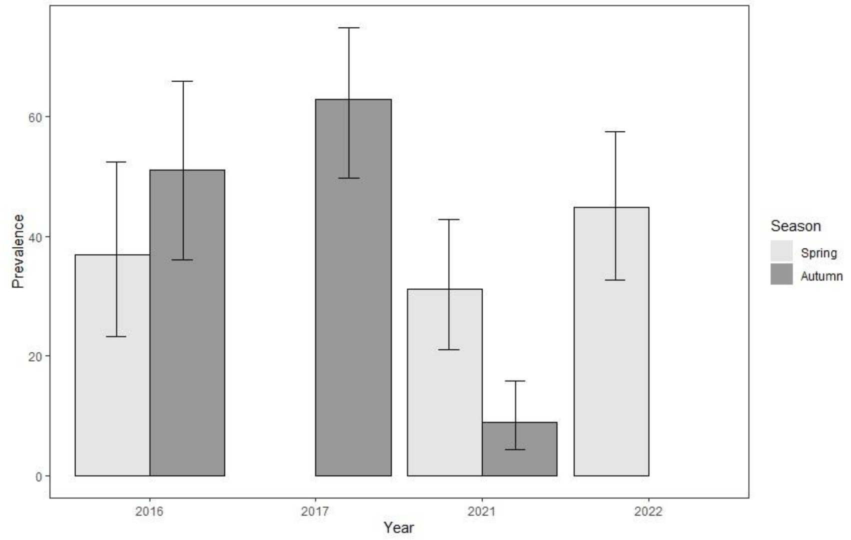

Figure 7. Prevalence of Sarcocystidae in *Clethrionomys glareolus* between each season per year. Error bars represent 95% confidence intervals (Maximum and Minimum).

Longitudinal analyses

Apodemus sylvaticus

The patterns of infections observed in the invasion gradient analyses for the pathogen species *Bartonella, M. haemomuris* and *M. coccoides* were examined in *A. sylvaticus* sampled in Ireland.

Formatted: Font: 12 pt

Formatted: Font: (Default) Times New Roman

Formatted: Normal

Formatted: Font: 10 pt, Not Bold

Bartonella

In the longitudinal analysis, the best model (lowest AICc value; 672.6) included time, season, region, and sex, with an interaction between time and season (Table 3). The interaction between time and season was shown to have a high variance inflation factor (VIF), meaning the covariates were correlated and potentially affecting the model through multicollinearity. To account for this, the interaction was removed in the overall model (AICc 674.665), and separate GLMM's were conducted for each season. In the overall model, no effect for time or region was observed (GLMM, family = Binomial, z = 1.904, $p = 0.057$). *A. sylvaticus* in autumn showed higher prevalences of *Bartonella* than in spring (GLMM, family = Binomial, $z = -3.708$, $p < 0.001$). Alongside this, males exhibited higher prevalences compared to females (GLMM, family = Binomial, $z = 2.241$, $p < 0.05$).

In the seasonal models, the best model (lowest AICc value; 354.832) for spring included time and sex. No effect for region was observed (Table 3). Prevalence was positively correlated (correlation coefficient; 0.15) with time (GLMM, family = Binomial, $z = 2.344$, $p = 0.019$; Pearsons's correlation test, $t = 2.4751$, $p < 0.05$). Sex did not significantly influence the prevalence of *Bartonella* in *A. sylvaticus* in the spring only model (GLMM, family = Binomial, z = 1.942, $p = 0.052$). In the autumn only analysis, the best model included only region (lowest AICc value; 322.788), however, no factor significantly influenced *Bartonella* prevalence.

Formatted: Not Highlight

Formatted: Not Highlight

Formatted: Not Highlight

Formatted: Not Highlight

Mycoplasma haemomuris

In the longitudinal analysis, no effect for time was observed, and the best model selected (lowest AICc value; 626.339) included the factors season, region, and age class (Table 3). Region was shown to have a significant effect on the prevalence of *M. haemomuris* (Region: Front 2016, GLMM, family = Binomial, $z = 2.744$, $p < 0.01$; Region: Front 2021, GLMM, family = Binomial, $z = 2.874$, $p < 0.01$). Post-hoc analysis demonstrated that the core had a lower prevalence of *M. haemomuris* compared to front 2016 and front 2021, with no difference observed between front 2016 and front 2021 (Figure 5) (Tukey Kramer analysis; Core – Front 2016, $p < 0.001$; Core – Front 2021, $p < 0.001$; Front 2016 – Front 2021, $p = 0.96$). Secondly, spring had higher prevalences compared to autumn (GLMM, family = Binomial, $z = 2.987$, $p < 0.01$). Finally, age class was shown to be a statistically significant factor (Age class: Juvenile, GLMM, family = Binomial, $z = -3.414$, $p < 0.001$; Age class: Mature, GLMM, family = Binomial, $z = 2.146$, $p < 0.05$). Post-hoc analysis showed mature adults were the most

Formatted: Not Highlight

Formatted: Not Highlight

infected, followed by subadults, with juveniles least infected (Tukey Kramer analysis; Mature - Young adults, *p* < 0.01; Mature – Juveniles, *p* < 0.001; Juveniles – Young adults, *p* < 0.001).

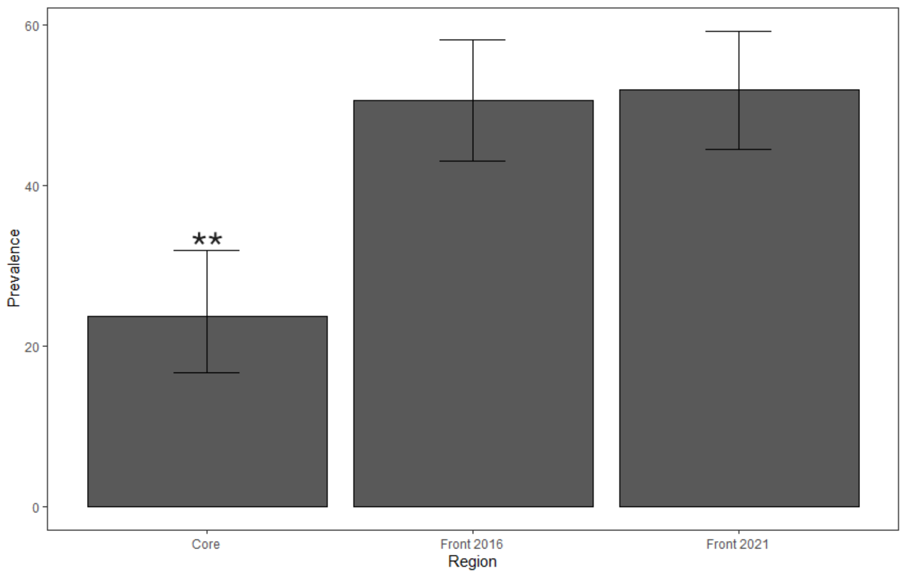

Figure 5. Prevalence of *Mycoplasma haemomuris* in *Apodemus sylvaticus* per region in the longitudinal analysis (all years). Error bars represent 95% confidence intervals. Asterisks indicate level of statistical significant (** = p <0.01)

Formatted: Font: (Default) Times New Roman

Mycoplasma coccoides

The longitudinal analysis showed that the best model (lowest AICc value; 677.786) included time, sex and age class (Table 3). Time was shown to be a significant factor (GLMM, family = Binomial, $z = -2.175$, $p < 0.05$), with a negative correlation (-0.1) between prevalence and time (years) (Pearson's correlation test, t=-2.2929, p < 0.05). This can be observed in Figure 6, where a reduction in prevalence is seen over time. Age class was also shown to affect prevalence (Age class: Mature, GLMM, family = Binomial, $z = -3.04$, $p < 0.01$). Post-hoc analysis demonstrated young adults being more infected than mature adults, however, no statistical differences

were observed between mature adults and juveniles and between juveniles and young adults (Tukey Kramer analysis; Young adults – Mature, $p < 0.05$; Mature – Juveniles, $p = 0.97$; Young adults – Juveniles, $p = 0.097$).

Formatted: Not Highlight

Formatted: Not Highlight

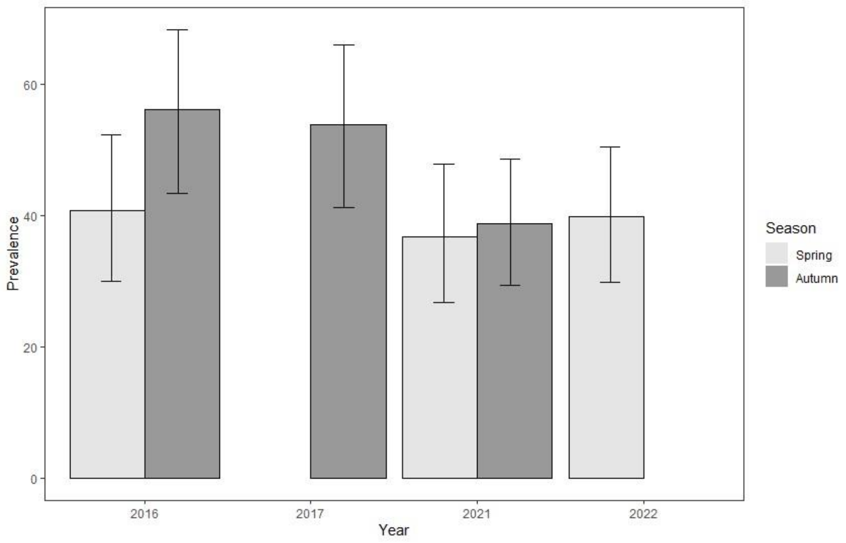

Figure 6. Prevalence of *Mycoplasma coccoides* in *Apodemus sylvaticus* for each season and year sampled. Error bars represent 95% confidence intervals.

Clethrionomys glareolus

Regarding *C. glareolus* in Ireland, the patterns of infection with Sarcocystidae observed during longitudinal analyses were examined.

Formatted: Font: (Default) Times New Roman

Formatted: Normal

Formatted: Font: 10 pt, Not Bold, English (Ireland)

Formatted: Font: Not Italic

Sarcocystidae

The longitudinal analysis of the prevalence of Sarcocystidae in *C. glareolus* found the best model (lowest AICc value; 460.593) included time, season, region, sex, age class and an interaction between time and season. A high VIF was observed for the interaction and hence it was removed from the final model (AICc value; 499.807 (Table 3). Time was shown to be statistically significant (GLMM, family = Binomial, $z = -4.849$, $p < 0.001$).

Post-hoc analysis found that prevalence is negatively correlated (-0.22) with time (years since baseline) (Pearson's correlation test; $t = -4.6122$, $p < 0.001$). Higher prevalence was observed in spring compared to autumn (GLMM, family = Binomial, $z = 3.547$, $p < 0.001$). Region was shown to be statistically significant (Region: Front 2021, GLMM, family = Binomial, $z = 2.143$, $p < 0.05$). Post-hoc analysis demonstrates that the core had a lower prevalence compared to front 2016 but not statistically different from front 2021, while no statistical difference was observed between front 2016 and front 2021 (Tukey Kramer analysis; Core – Front 2016, $p < 0.05$; Core – Front 2021, $p = 0.5$; Front 2016 – Front 2021, $p = 0.53$). Females had higher prevalences than males (GLMM, family = Binomial, $z = -2.216$, $p < 0.05$). Finally, age class was shown to be a statistically significant factor (Age class: Mature, GLMM, family = Binomial, $z = -2.322$, $p < 0.05$). Post-hoc analysis showed that juveniles were more infected than mature adults. No statistical difference was observed between mature adults and young adults, or young adults and juveniles (Tukey Kramer analysis; Juveniles – Mature, $p < 0.01$; Juveniles – Young adults, $p = 0.14$; Mature – Young adults, $p = 0.15$).

Separate binomial GLMMs for each season were run on the longitudinal models to account for the removed interaction between season and time (Table 3). In spring, the best model (lowest AICc value; 232.189) included region and age class. Region was shown to be statistically significant (Region: Front 2016, GLMM, family = Binomial, $z = 2.188$, $p < 0.05$; Region: Front 2021, GLMM, family = Binomial, $z = 3.769$, $p < 0.001$). Post-hoc analysis demonstrated that the core had a lower prevalence when compared to both front 2016 and front 2021, while no difference was observed in the post hoc analysis between the two front regions identified in 2016 and 2021 (Tukey Kramer analysis; Core – Front 2016 $p < 0.01$; Core – Front 2021 $p < 0.001$; Front 2016 – Front 2021, $p = 0.92$). Age class was also statistically significant (Age class: Mature, GLMM, family = Binomial, $z = -2.112$, $p < 0.05$). Post-hoc analysis showed juveniles and young adults more likely to be infected compared to mature adults, but no statistical difference was observed between juveniles and young adults (Tukey Kramer analysis; Mature – Juveniles, $p < 0.01$; Mature – Young adults, $p < 0.05$; Young adults – Juveniles, $p = 0.14$).

Finally, the best model (lowest AICc value; 224.497) for autumn included time, sex, and age class. No effect for region was observed. Time was shown to be statistically significant (GLMM, family = Binomial, $z = -6.766$, $p < 0.001$) with time being negatively correlated (-0.5) with prevalence (Pearsons's correlation test, $t = -8.5352$, $p < 0.001$). Females had higher prevalences compared to males (GLMM, family = Binomial, $z = -2.231$, $p < 0.05$). Age class did not significantly influence the prevalence of Sarcocystidae in this analysis (Age class: Mature, GLMM, family = Binomial, z = -0.848, $p = 0.4$; Age class; Juvenile, GLMM, family = Binomial, z = 1.727, $p = 0.084$).

Formatted: Not Highlight

Formatted: Not Highlight

Formatted: Not Highlight

Formatted: Not Highlight

Formatted: Font: 12 pt, Bold, Italic

Discussion

The aim of this study was to investigate the effect an invasive species may have on native pathogen communities, including zoonotic species~~s~~, using the bio-invasion of Ireland by *C. glareolus* as a model system. While previous studies on rodent-borne microparasites in Ireland have focused on single bacterial ~~pathogens in rodents before~~species, such as Bartonella or Borrelia (70-72), this is the first time 16SrRNA ~~screening~~ metabarcoding techniques have been used to identify the full communities of potentially zoonotic pathogens ~~of zoonotic risk~~, and their prevalence levels, that are circulating in Irish populations of *C. glareolus and A. sylvaticus*. Furthermore, this study has created~~creating~~ an important baseline for rodent-borne disease studies in Ireland, and highlights~~ing~~ new important areas of research to guide future research in Ireland, and potentially other invasion systems (e.g ~~Zintl *et al.,* 2023~~ (73)).

Rodent-borne ~~infectious risk~~pathogens observed in Ireland

Firstly, we discovered that Irish rodents exhibit a depauperate pathogen richness, with OTUs relating to only four pathogenic bacterial species and one family of protozoans in sampled rodent populations. Although the species in the Sarcocystidae OTUs were not identified in the present study, comparison with published sequences using NCBI BLAST indicated these may contain pathogens of veterinary and medical importance, including *Toxoplasma, Eimeria* or *Neospora*. ~~Furthermore, we observed that the *C. glareolus* population in Ireland expresses patterns consistent with the enemy release hypothesis, and potentially disrupts the transmission of native pathogen species.~~ Finally, this study examined the importance of considering time when examining the effect biological invaders have on pathogen communities. For example, in contrast to a previous study by Telfer *et al.,* (72) we detected *Bartonella* in *C. glareolus* in Ireland, albeit at a low prevalence rate. This result was surprising as the previous study had postulated that *C. glareolus* in Ireland might be resistant to native strains (72). Further molecular work is required to determine if the *Bartonella* observed during this study is the same species of *Bartonella* observed in mice during the study by Telfer *et al.,* (72).

It is also worth noting that despite being native, *A. sylvaticus* also exhibited a depauperate community of potentially pathogenic bacterial taxa when compared to French populations, with no *Anaplasma, Candidatus Neoehrlichia, Francisella* or *Orientia* observed in the Irish population. This is consistent with island

biogeography theory, which states that species diversity is limited by their size and isolation from the mainland (74, 75). The absence of certain vector-borne pathogens might also be explained by the low vector diversity in Ireland. For example, reviews in Ireland and France have shown Ireland to have 13 species of ticks, compared to approximately 40 tick species in France (76, 77). However, assays specifically targeting these pathogens would need to be carried out to confirm their absence. Furthermore, *Borrelia* was also notably absent from Irish rodents in this study, although it had been detected, albeit at low prevalences, in Irish rodents by previous studies (70, 71). This discrepancy may again be a result of the methods and target organ used in the present study, with previous studies using ear biopsies and species specific PCR (70, 71). Also, while *Apodemus* species and *C. glareolus* are considered important aspects of the *Borrelia* life cycle in Europe, these rodents may play a limited role as reservoir in Ireland, with the rodent-associated, *Borrelia afzelii*, being less common in Ireland (70, 71, 78, 79). Additionally, the absence of *Anaplasma* from rodents sampled in the present study agree with findings reported by Flattery *et al.* (80), who screened questing nymphal *Ixodes ricinus* ticks collected across Ireland for the presence of *Anaplasma phagocytophilum* and only found groEL ecotype 1, a strain that is not thought to be associated with rodents.

Enemy release in the invasive *Clethrionomys glareolus*

Two patterns consistent with the enemy release hypothesis were observed for bacterial pathogens in the invasive *C. glareolus* population in Ireland for the first time~~, at both an international intraspecific scale and interspecific scale~~. Firstly, a comparison of~~by comparing~~ the number of bacterial taxa in Irish and French *C. glareolus* populations indicated that~~,~~ the population of *C. glareolus* sampled in Ireland had fewer potentially pathogenic bacterial taxa ~~when compared to~~than conspecifics in their native range. This can be seen particularly in the 2021 data, where the Irish *C. glareolus* individuals had fewer potentially pathogenic bacterial taxa and lower prevalences compared to *C. glareolus* populations in France. In particular, a number of potentially pathogenic taxa were completely absent from *C. glareolus* populations in Ireland including: *Anaplasma, Candidatus Neoehrlichia, Francisella and Orientia*. Further studies should be conducted to confirm this and incorporate consistent aging techniques in both countries allowing for more in-depth statistical analysis between samples from Ireland and France. Secondly, *C. glareolus* exhibited enemy release from potentially pathogenic bacterial taxa in comparison to the native *A. sylvaticus* in Ireland. While *C. glareolus* was found to have one more

bacterial taxon when compared to *A. sylvaticus, C. glareolus* has lower prevalences of these bacterial taxa in the overall data, and a significantly lower pathogen species richness compared to *A. sylvaticus* in the invasion gradient analysis (2016 only).

These findings are comparable to studies investigating the helminth communities in the invasive *C. glareolus* in Ireland (23, 39). These authors found the same two examples of enemy release, i.e. *C. glareolus* had a lower helminth species richness compared to its native range, and to the native *A. sylvaticus* in Ireland (23, 39). Likewise, similar patterns have been observed in other invasion systems. For example in the Senegal invasion system, Diagne *et al.* (24) demonstrated that native rodents (*Mastomys erythroleucus* and *Mastomys natalensis*) had a greater species richness of pathogenic bacterial OTUs when compared to non-native rodents (*Mus musculus domesticus* and *Rattus rattus*). These findings from investigating invasion systems in different countries and invasive species can provide insights into the underlying mechanisms contributing to how these species survive, establish and outcompete native fauna when colonising new environments. For example, this pattern of enemy release could allow the invasive species to direct more energy from immune responses into growth or fecundity, potentially giving invasive species a competitive advantage over native species (81).

Stuart *et al.* (39) described a third example of enemy release in the Irish population of *C. glareolus,* namely the reduced abundance of the directly transmitted nematode *A. tianjinsensis* in *C. glareolus* in the invasion front compared to the invasion core in spring and autumn in 2016 (39). A similar example of enemy release at the invasion front was not found during this present study. In contrast, in the invasion gradient analysis, *C. glareolus* was more likely to be infected in the invasion front, compared to the more established invasion core, with significantly higher prevalences of Sarcocystidae in front 2016 compared to the core. This pattern was also observed in 2016 for the single Sarcocystidae OTU which seems to be specific to *C. glareolus*, with no *A. sylvaticus* found infected by this OTU in this study. These findings are in direct contrast to the enemy release hypothesis, as the more recently invaded populations exhibited higher infection rates, highlighting that infection patterns may be highly pathogen specific.

This result was all the more surprising given that male *C. glareolus* at the invasion front are thought to be more timid and less exploratory than their conspecifics at core sites (82), characteristics which would be expected to reduce their exposure to pathogens. On the other hand, there also seems to be a strong link between spatial behaviour and behaviour traits, with shy but thorough explorers using larger areas (82). If animals are exploring larger areas at the expansion front, this could potentially result in greater exposures to pathogens.

Finally, a possible explanation for the increased infection rate at the invasion front might lie in the immunology of *C. glareolus* during its invasion of Ireland. Pathogens may lag behind biological invasions, as the population density of the invading species at the front is too low to maintain sufficient transmission. Consequently, the reallocation of resources from immune responses, known as the evolution of increased competitive ability hypothesis and enemy release from pathogens, could be favoured in invasive populations (22, 47). This could potentially leave *C. glareolus* in the invasion front relatively immunologically naïve and hence, susceptible to increased Sarcocystidae infection when the population increases over a certain threshold. This would be further supported by the findings in the longitudinal analysis, that juveniles have a higher prevalence of Sarcocystidae compared to mature adults, as juveniles would potentially be more immunologically naïve. In a study of the population genomics of *C. glareolus* in Ireland, White *et al.* (83) identified the selection of genes with immunological function in *C. glareolus* furthest from the point of introduction. However, to fully understand these mechanisms further investigation of the eco-immunology of invasive *C. glareolus* populations, and identification of the species present within these Sarcocystidae OTUs is required.

Formatted: Font: (Default) Times New Roman

Effect of *Clethrionomys glareolus* invasion on pathogen communities in the native *Apodemus sylvaticus*

The effect of the *C. glareolus* invasion on the pathogen communities in the native *A. sylvaticus* was also investigated. It was hypothesised that the presence of *C. glareolus* might affect the pathogen communities in the native *A. sylvaticus*. However, no dilution or amplification effect resulting from the *C. glareolus* invasion gradient on pathogen species richness in *A. sylvaticus* was observed in the present study. Likewise, in the invasion gradient analysis (2016 data only), no effect for invasion region was found on the prevalence of *A. sylvaticus'* pathogens. However, using the longitudinal data, the increased relative abundance of *C. glareolus* at the site of introduction apparently caused a dilution effect for *M. haemomuris* in *A. sylvaticus*. In this instance the core showed significantly reduced prevalences of *M. haemomuris* in *A. sylvaticus* compared to conspecifics from the invasion front regions (front 2016 and front 2021). No difference was observed between front 2016 and front 2021 indicating that the reduction in *M. haemomuris* prevalence may be directly attributable to the higher *C. glareolus* abundance at the core. The potential reason for a dilution effect being observed in the longitudinal data and not in the 2016 only data is likely due to the increased statistical power in the longitudinal analysis, as a result of the increased sample size.

In a previous study investigating the impact of forest anthropization on small mammal pathogens, Bouilloud (84) detected a dilution effect for *M. haemomuris* (Strain 2) associated with increased small mammal diversity in France. The author argued that the declining abundance of its most competent host, *C. glareolus,* as host species diversity increased, might explain the dilution of *M. haemomuris* at these sites. The dilution of *M. haemomuris,* in *A. sylvaticus* in Ireland due to the presence of *C. glareolus,* and the dilution of the strain 2 in France, presumably due to increased small mammal diversity, demonstrate that despite the multiple factors that can alter rodent populations (e.g. climate change, invasive species, deforestation and urbanisation), that understanding the underlying mechanisms behind rodent-borne disease can allow for better predictions of disease transmission and improved disease management. Interestingly, the dilution pattern identified for *M. haemomuris* was not observed for *M. coccoides*. The driver behind the different patterns of these *Mycoplasma* spp. may possibly be a result of the specific behaviour of the respective vector species, *Polyplax serrata* in the case of *M. coccoides* and *Polyplax spinulosa* in the case of *M. haemomuris* (85, 86). However, studies on these pathogens vector species are based on mice and rats' laboratory experiments only. Therefore, field-based studies are needed to confirm the possibility that *C. glareolus* might be a less favourable host for the vector transmitting *M. haemomuris*, or the bacteria itself, in Ireland, which could result in a dilution effect in the native *A. sylvaticus.*

Surprisingly, we detected no dilution effect for *Bartonella* in *A. sylvaticus* in this present study. *Bartonella birtlesii* and *Bartonella taylorii* have previously been recorded in Ireland, with prevalences in *A. sylvaticus* decreasing with increased *C. glareolus* densities (72). These seemingly contradictory observations may be due to the time that has elapsed since the study by Telfer *et al.* (72) and the present study. It is possible for example, that the initial invasion of *C. glareolus* disrupted these pathogens, but they have recovered since. On the other hand, only a single *C. glareolus* individual was found to be infected with *Bartonella* here, corroborating the suggestion that *C. glareolus* in Ireland is not a competent host for this pathogen species. Hence, the observed difference may stem from the detection techniques employed, as Telfer *et al.* (72) utilized bacterial cultivation and genus-specific PCR assays, while this present study only found one OTU of *Bartonella,* using 16SrRNA amplification and illumina sequencing. Further work, using genus specific primers, such as those used by Telfer *et al.* (72) would be needed to examine *Bartonella* positive rodents to determine species identity, and hence identify whether dilution does still occur in the Irish *A. sylvaticus* population.

Changes in rodent pathogen communities over time

Finally, the effect of time on the prevalence of the various pathogenic taxa, as the *C. glareolus* invasion progressed was examined. Prevalence decreased between 2016 and 2022, for both *M. coccoides* in *A. sylvaticus* and Sarcocystidae in *C. glareolus*. However, examining the spring longitudinal data only, *Bartonella* prevalence in *A. sylvaticus* increased between 2016 and 2022. These patterns show that pathogen communities are dynamic, and longitudinal studies are essential for fully understanding the changes that occur in pathogen communities. Cornet *et al.* (22), expressed the need for longitudinal studies to examine the time-lag for invasive species between invasion and adapting to the invasion front. A recent review by Brian and Catford (87) highlighted that time since introduction is a significant factor when investigating the enemy release hypothesis, as enemy impact on the invader will potentially increase with time, as pathogens adapt to the invasive species. In our case, the dilution effect for *M. haemomuris* in *A. sylvaticus* was only observed in the longitudinal data, an observation that could potentially have been missed in a single timepoint study. Moreover, *M. haemomuris, M. penetrans* and *Bartonella* were not detected in *C. glareolus* in the baseline 2016 data. While this could be attributed to the increased sample size afforded by multiple years of sampling, focusing on a single year, could have resulted in missing the dilution for *M. haemomuris* in *A. sylvaticus* and the assumption that *C. glareolus* is not infected by these pathogens in Ireland. Similarly, the recently reported *Babesia microti* 'Munich' strain in Ireland was detected exclusively in *C. glareolus* located in the eastern part of the country, which corresponds to the *C. glareolus* invasion front (73). The primary vector of this strain, *Ixodes trianguliceps*, has also been mainly reported in eastern Ireland (88). If the assumption that *B. microti* is confined to the eastern regions of Ireland is correct, this would further demonstrate the importance of long-term studies on the pathogens of invasive species, to monitor the emergence of new pathogens in newly colonised areas. Finally, more long-term data is needed to fully understand if rodents and their pathogens fluctuate through multi-annual cycles, which is seen in other countries where long-term data is gathered, such as Finland (89-91).

Conclusion

In conclusion, patterns consistent with the enemy release hypothesis were observed in the Irish *C. glareolus* population. Despite exhibiting higher prevalences of Sarcocystidae, *C. glareolus* in Ireland shows reduced pathogen species richness, and lower prevalences of bacterial ~~prevalences~~taxa, compared to the native *A. sylvaticus* and fewer bacterial taxa compared to the native range of the species. These findings increase our

understanding of the mechanism of how invasive species thrive in new environments and outcompete native fauna, however, eco-immunological data are required to fully understand these patterns of enemy release. Secondly, the present study showed the disruptive effect of non-native host species on native pathogen systems, with the example of a dilution effect and reduced prevalence of *M. haemomuris* in *A. sylvaticus* at the invasion core compared to invasion fronts, where *C. glareolus* populations exhibit higher abundance. These findings show how changes in biodiversity, such as the addition of new hosts with varying levels of competencies for a given pathogen, may disrupt pathogen communities and host-pathogen dynamics. Further, work is also needed to identify the Sarcocystidae and *Bartonella* species detected in this study in order to fully understand the pathogen community dynamics, as genetic differences may reveal different levels of host specificity and help explain host-pathogen interactions. Finally, time, as the *C. glareolus* invasion progresses, was shown to be correlated, both positively and negatively, with three pathogen taxa during this study, illustrating that invasion systems are dynamic, and pathogen communities can fluctuate as the invader becomes more established. By conducting a longitudinal investigation, we demonstrate that long-term data are essential to fully understand eco-evolutionary processes driving invasion dynamics and success, preventing conclusions drawn from single timepoint studies being assumed as long-term changes in pathogen communities.

Acknowledgements

We would like to thank Ian Montgomery, Colin Lawton and John Rochford for loaning their traps. We would also like to thank Breda Fitzpatrick, Patrick Dalton, Hugh O'Connor and the other technical staff at MTU for their help and loan of equipment. Finally, we would like to thank TCD students, volunteers and interns, Laura McCarthy, Lucie Veitová and Paula Curtin for their help in the field and lab processing the Irish samples. We are grateful to Audrey Weber (AGAP laboratory) and the GenSeq facilities for the Illumina MiSeq sequencing, and the Genotoul bioinformatics platform Toulouse Midi-Pyrénées (Bioinfo Genotoul). Thank you to all the BioRodDis team members who have been vital to this study through the sharing of data, discussion of findings and statistical analyses. We would also like to thank Niamh Reilly for helping proofread the manuscript.

Statements & Declarations

Funding

This research was funded through the 2018–2019 BiodivERsA joint call for research proposals, under the BiodivErsA3 ERA-Net COFUND programme, and with the funding organisations EPA Research Programme, Agence Nationale de la Recherche (ANR), Research Foundation–Flanders (FWO), National Science Centre, Poland (NCN) and Deutsche Forschungsgemeinschaft (DFG). This project is funded under the EPA Research Programme 2021–2030 and cofunded by Agence Nationale de la Recherche (ANR), Research Foundation–Flanders (FWO), National Science Centre, Poland (NCN) and Deutsche Forschungsgemeinschaft (DFG).

This project is funded under the EPA Research Programme 2021-2030. The EPA Research Programme is a Government of Ireland initiative funded by the Department of the Environment, Climate and Communications (project number 2019-NC-MS-10).

2016 and 2017 sampling was funded by the Irish Research Council, Government of Ireland Postdoctoral Fellowship Programme and by the Marie Curie Research Grants Scheme, European Union, grant (749312, INVASIMMUN).

The funders had no role in study design, data collection and analysis, decision to publish, or preparation of the manuscript.

The author, Andrew McManus, received a stipend to complete this research from the EPA Research Programme as part of their PhD studies.

Ethics statement:

This study was carried out under ethical approval from the universities Trinity College Dublin animal research ethics committee and Munster Technological University ethics board, in compliance with the Health Products Regulatory Association (HPRA) of Ireland for euthanasia of the rodents sampled (Authorisation Number: AE22171/I004).

Competing Interests:

The authors declare no competing or financial interests.

Author contributions.

~~Andrew McManus, Celia Holland, Nathalie Charbonnel, Maxime Galan and Peter Stuart contributed to the study conception and design. Material preparation, data collection and analysis were performed by Andrew McManus, Nathalie Charbonnel, Maxime Galan, Peter Stuart and Vincent Sluydst. The first draft of the manuscript was prepared by Andrew McManus and all authors commented on previous version of the manuscript. All authors have read and approved the final manuscript.~~

Author contributions

Conceptualization: Andrew McManus, Celia Holland, Nathalie Charbonnel, Maxime Galan, Peter Stuart

Data Curation: Andrew McManus, Maxime Galan, Peter Stuart

Formal Analysis: Andrew McManus, Nathalie Charbonnel, Maxime Galan, Vincent Sluydst, Peter Stuart

Funding Acquisition: Nathalie Charbonnel, Peter Stuart

Investigation: Andrew McManus, Celia Holland, Nathalie Charbonnel, Maxime Galan, Vincent Sluydst, Peter Stuart

Methodology: Andrew McManus, Celia Holland, Nathalie Charbonnel, Maxime Galan, Vincent Sluydst, Peter Stuart

Project Administration: Nathalie Charbonnel, Peter Stuart

Resources: Andrew McManus, Nathalie Charbonnel, Maxime Galan, Peter Stuart

Software: Andrew McManus, Nathalie Charbonnel, Maxime Galan, Vincent Sluydst

Supervision: Celia Holland, Nathalie Charbonnel, Peter Stuart

Validation: Andrew McManus, Nathalie Charbonnel, Maxime Galan, Vincent Sluydst

Visualization: Andrew McManus

Writing – Original Draft Preparation: Andrew McManus, Peter Stuart

Writing – Review & Editing: Andrew McManus, Celia Holland, Nathalie Charbonnel, Maxime Galan, Vincent Sluydst, Peter Stuart, Annetta Zintl

References

1. Jones KE, Patel NG, Levy MA, Storeygard A, Balk D, Gittleman JL, et al. Global trends in emerging infectious diseases. Nature. 2008;451(7181):990-3. doi: 10.1038/nature06536
2. Brown K, Leggat PA. Human monkeypox: current state of knowledge and implications for the future. Tropical medicine and infectious disease. 2016;1(1):8. doi: 10.3390/tropicalmed1010008
3. Mackenzie JS, Smith DW. COVID-19: a novel zoonotic disease caused by a coronavirus from China: what we know and what we don't. Microbiology Australia. 2020;41(1):45-50. doi: 10.1071/MA20013
4. Roche B, Garchitorena A, Guégan JF, Arnal A, Roiz D, Morand S, et al. Was the COVID-19 pandemic avoidable? A call for a "solution-oriented" approach in pathogen evolutionary ecology to prevent future outbreaks. Ecology letters. 2020. doi: 10.1111/ele.13586
5. Heinrich ML, Boisen ML, Nelson DK, Bush DJ, Cross RW, Koval AP, et al. Antibodies from Sierra Leonean and Nigerian Lassa fever survivors cross-react with recombinant proteins representing Lassa viruses of divergent lineages. Scientific Reports. 2020;10(1):1-19. doi: 10.1038/s41598-020-72539-w
6. Dharmarajan G, Li R, Chanda E, Dean KR, Dirzo R, Jakobsen KS, et al. The animal origin of major human infectious diseases: what can past epidemics teach us about preventing the next pandemic? Zoonoses. 2022;2(1).
7. Dhawan M, Choudhary OP. Emergence of monkeypox: risk assessment and containment measures. Travel Medicine and Infectious Disease. 2022;49:102392. doi: 10.1016/j.tmaid.2022.102392
8. Daszak P, Cunningham AA, Hyatt AD. Emerging infectious diseases of wildlife--threats to biodiversity and human health. science. 2000;287(5452):443-9. doi: 10.1126/science.287.5452.443
9. Wikelski M, Foufopoulos J, Vargas H, Snell H. Galápagos birds and diseases: invasive pathogens as threats for island species. Ecology and Society. 2004;9(1).
10. Wilcox BA, Gubler DJ. Disease ecology and the global emergence of zoonotic pathogens. Environmental Health and Preventive Medicine. 2005;10(5):263-72. doi: 10.1265/ehpm.10.263
11. Hatcher MJ, Dick JT, Dunn AM. Disease emergence and invasions. Functional Ecology. 2012;26(6):1275-87. doi: 10.1111/j.1365-2435.2012.02031.x
12. Becker DJ, Albery GF, Kessler MK, Lunn TJ, Falvo CA, Czirják GÁ, et al. Macroimmunology: The drivers and consequences of spatial patterns in wildlife immune defence. Journal of Animal Ecology. 2020;89(4):972-95. doi: 10.1111/1365-2656.13166
13. IPBES. Global assessment report on biodiversity and ecosystem services of the Intergovernmental Science-Policy Platform on Biodiversity and Ecosystem Services. In: Brondízio ES, Settele J, Díaz S, Ngo HT, (Editors), editors.: IPBES secretariat, Bonn, Germany; 2019. doi: 10.5281/zenodo.3831673~~p. 1144.~~
14. Schreiner CL, Nuismer SL, Basinski AJ. When to vaccinate a fluctuating wildlife population: Is timing everything? Journal of Applied Ecology. 2020;57(2):307-19. doi: 10.1111/1365-2664.13539
15. Bezerra-Santos MA, Dantas-Torres F, Mendoza-Roldan JA, Thompson RA, Modry D, Otranto D. Invasive mammalian wildlife and the risk of zoonotic parasites. Trends in parasitology. 2023;39(9):786-98. DOI: 10.1016/j.pt.2023.06.004~~.~~

Formatted: Indent: Left: 0", Hanging: 0.5"

Formatted: Indent: First line: 0.5"

Formatted: Indent: Left: 0.5"

Formatted: Indent: Left: 0", Hanging: 0.5"

16. Eby P, Peel AJ, Hoegh A, Madden W, Giles JR, Hudson PJ, et al. Pathogen spillover driven by rapid changes in bat ecology. Nature. 2023;613(7943):340-4. doi: 10.1038/s41586-022-05506-2
17. Bonesi L, Palazon S. The American mink in Europe: status, impacts, and control. Biological conservation. 2007;134(4):470-83. doi: 10.1016/j.biocon.2006.09.006
18. Salgado I. Is the raccoon (*Procyon lotor*) out of control in Europe? Biodiversity and Conservation. 2018;27(9):2243-56. doi: 10.1007/s10531-018-1535-9
19. Keane RM, Crawley MJ. Exotic plant invasions and the enemy release hypothesis. Trends in Ecology & Evolution. 2002;17(4):164-70. DOI: 10.1016/S0169-5347(02)02499-0
20. Torchin ME, Lafferty KD, Dobson AP, McKenzie VJ, Kuris AM. Introduced species and their missing parasites. Nature. 2003;421(6923):628-30. doi: https://doi.org/10.1038/nature01346
21. Lee KA, Klasing KC. A role for immunology in invasion biology. Trends in Ecology & Evolution. 2004;19(10):523-9. DOI: 10.1016/j.tree.2004.07.012
22. Cornet S, Brouat C, Diagne C, Charbonnel N. Eco-immunology and bioinvasion: Revisiting the evolution of increased competitive ability hypotheses. Evolutionary Applications. 2016;9(8):952-62. doi: 10.1111/eva.12406
23. Loxton KC, Lawton C, Stafford P, Holland CV. Reduced helminth parasitism in the introduced bank vole (*Myodes glareolus*): More parasites lost than gained. International Journal for Parasitology: Parasites and Wildlife. 2016;5(2):175-83. doi: 10.1016/j.ijppaw.2016.05.005
24. Diagne C, Galan M, Tamisier L, d'Ambrosio J, Dalecky A, Bâ K, et al. Ecological and sanitary impacts of bacterial communities associated to biological invasions in African commensal rodent communities. Scientific reports. 2017;7(1): 14995. doi: 10.1038/s41598-017-14880-1~~:1-11.~~
25. Chalkowski K, Lepczyk CA, Zohdy S. Parasite ecology of invasive species: conceptual framework and new hypotheses. Trends in parasitology. 2018;34(8):655-63. DOI: 10.1016/j.pt.2018.05.008
26. Dunn AM, Hatcher MJ. Parasites and biological invasions: parallels, interactions, and control. Trends in Parasitology. 2015;31(5):189-99. DOI: 10.1016/j.pt.2014.12.003
27. Schmidt KA, Ostfeld RS. Biodiversity and the dilution effect in disease ecology. Ecology. 2001;82(3):609-19. doi: 10.1890/0012-9658(2001)082[0609:BATDEI]2.0.CO;2
28. Tierney PA, Caffrey JM, Vogel S, Matthews SM, Costantini E, Holland CV. Invasive freshwater fish (*Leuciscus leuciscus*) acts as a sink for a parasite of native brown trout Salmo trutta. Biological Invasions. 2020;22(7):2235-50. doi: 10.1007/s10530-020-02253-1
29. Keesing F, Ostfeld RS. Dilution effects in disease ecology. Ecology Letters. 2021;24(11):2490-505. doi: 10.1111/ele.13875
30. Kelly D, Paterson R, Townsend C, Poulin R, Tompkins D. Parasite spillback: a neglected concept in invasion ecology? Ecology. 2009;90(8):2047-56. doi: 10.1890/08-1085.1
31. Loxton KC, Lawton C, Stafford P, Holland CV. Parasite dynamics in an invaded ecosystem: helminth communities of native wood mice are impacted by the invasive bank vole. Parasitology. 2017;144(11):1476-89. doi: 10.1017/S0031182017000981
32. Diagne C, Ballesteros-Mejia L, Cuthbert RN, Bodey TW, Fantle-Lepczyk J, Angulo E, et al. Economic costs of invasive rodents worldwide: the tip of the iceberg. PeerJ. 2023;11:e14935. doi: 10.7717/peerj.14935
33. Meerburg BG, Singleton GR, Kijlstra A. Rodent-borne diseases and their risks for public health. Critical Reviews in Microbiology. 2009;35(3):221-70. doi: 10.1080/10408410902989837
34. Pedersen AB, Babayan SA. Wild immunology. Molecular ecology. 2011;20(5):872-80. doi: 10.1111/j.1365-294X.2010.04938.x
35. Han BA, Schmidt JP, Bowden SE, Drake JM. Rodent reservoirs of future zoonotic diseases. Proceedings of the National Academy of Sciences. 2015;112(22):7039-44. doi: 10.1073/pnas.1501598112
36. Jackson J. Immunology in wild nonmodel rodents: an ecological context for studies of health and disease. Parasite Immunology. 2015;37(5):220-32. doi: 10.1111/pim.12180

Formatted: Font: Italic

Formatted: Font: Italic

Formatted: Font: Italic

37. McManus A, Holland CV, Henttonen H, Stuart P. The Invasive Bank Vole (*Myodes glareolus*): A Model System for Studying Parasites and Ecoimmunology during a Biological Invasion. Animals. 2021;11(9):2529. doi: 10.3390/ani11092529
38. Diagne C, Granjon L, Gueye MS, Kane M, Niang Y, Tatard C, et al. Association between temporal patterns in helminth assemblages and successful range expansion of exotic *Mus musculus domesticus* in Senegal. Biological Invasions. 2020;22(10):3003-16. doi: 10.1007/s10530-020-02304-7
39. Stuart P, Paredis L, Henttonen H, Lawton C, Torres CAO, Holland CV. The hidden faces of a biological invasion: parasite dynamics of invaders and natives. International Journal for Parasitology. 2020;50(2):111-123. doi: 10.1016/j.ijpara.2019.11.003.
40. Marnell F, Looney D, Lawton C. Ireland Red List No. 12: Terrestrial Mammals. National Parks and Wildlife Service, Department of the Culture, Heritage and the Gaeltacht, Dublin, Ireland; 2019.
41. Stuart P, Mirimin L, Cross T, Sleeman D, Buckley NJ, Telfer S, et al. The origin of Irish bank voles *Clethrionomys glareolus* assessed by mitochondrial DNA analysis. The Irish Naturalists' Journal. 2007:440-6.
42. White TA, Lundy MG, Montgomery WI, Montgomery S, Perkins SE, Lawton C, et al. Range expansion in an invasive small mammal: influence of life-history and habitat quality. Biological Invasions. 2012;14(10):2203-15. doi: 10.1007/s10530-012-0225-x
43. Montgomery WI, Lundy MG, Reid N. 'Invasional meltdown': evidence for unexpected consequences and cumulative impacts of multispecies invasions. Biological Invasions. 2012;14(6):1111-25. doi: https://doi.org/10.1007/s10530-011-0142-4
44. Razzauti M, Galan M, Bernard M, Maman S, Klopp C, Charbonnel N, et al. A comparison between transcriptome sequencing and 16S metagenomics for detection of bacterial pathogens in wildlife. PLoS Neglected Tropical Diseases. 2015;9(8):e0003929. doi: 10.1371/journal.pntd.0003929
45. Hazel S, Bennett M, Chantrey J, Bown K, Cavanagh R, Jones T, et al. A longitudinal study of an endemic disease in its wildlife reservoir: cowpox and wild rodents. Epidemiology & Infection. 2000;124(3):551-62. doi: 10.1017/S0950268899003799
46. Davis S, Calvet E, Leirs H. Fluctuating Rodent Populations and Risk to Humans from Rodent-Borne Zoonoses. Vector-Borne and Zoonotic Diseases. 2005;5(4):305-14. doi: 10.1089/vbz.2005.5.305
47. White TA, Perkins SE. The ecoimmunology of invasive species. Functional Ecology. 2012;26(6):1313-23. doi: 10.1111/1365-2435.12012
48. Galan M, Razzauti M, Bard E, Bernard M, Brouat C, Charbonnel N, et al. 16S rRNA amplicon sequencing for epidemiological surveys of bacteria in wildlife. MSystems. 2016;1(4):e00032-16. doi: 10.1128/msystems.00032
49. Bouilloud M, Galan M, Dubois A, Diagne C, Marianneau P, Roche B, et al. Three-way relationships between gut microbiota, helminth assemblages and bacterial infections in wild rodent populations. Peer Community Journal. 2023;3. doi: 10.24072/pcjournal.243.
50. Behnke J, Barnard C, Bajer A, Bray D, Dinmore J, Frake K, et al. Variation in the helminth community structure in bank voles (*Clethrionomys glareolus*) from three comparable localities in the Mazury Lake District region of Poland. Parasitology. 2001;123(4):401-14. doi: 10.1017/S0031182001008605
51. Abbate JL, Galan M, Razzauti M, Sironen T, Voutilainen L, Henttonen H, et al. Pathogen community composition and co-infection patterns in a wild community of rodents. Peer Community Journal; 2024. doi: 10.24072/pcjournal.370.
52. Kircher M, Sawyer S, Meyer M. Double indexing overcomes inaccuracies in multiplex sequencing on the Illumina platform. Nucleic Acids Research. 2011;40(1):e3-e. doi: https://doi.org/10.1093/nar/gkr771
53. Wickham H. Plyr 1.8.8 R package 2022 [Available from: https://CRAN.R-project.org/package=plyr].

Formatted: Font: Italic

Formatted: Font: Italic

Formatted: Font: Italic

Formatted: Font: Italic

Formatted: Default Paragraph Font

54. Wickham H, Chang W, Henry L, Pedersen TL, Takahashi K, Wilke C, et al. GGplot2 3.4.3 R package 2023 [Available from: https://CRAN.R-project.org/package=ggplot2].
55. Scherer R. PropCIs R package 2018 [Available from: https://CRAN.R-project.org/package=PropCIs].
56. Pradel J, Bouilloud M, Loiseau A, Piry S, Galan M, Artige E, et al. Small terrestrial mammals (Rodentia and Soricomorpha) along a gradient of forest anthropisation (reserves, managed forests, urban parks) in France. Biodiversity Data Journal. 2022;10. doi: 10.3897/BDJ.10.e95214
57. Brooks M, Bolker B, Kristensen K, Maechler M, Magnusson A, McGillycuddy M, et al. glmmTMB 1.1.7 R package 2023 [Available from: https://CRAN.R-project.org/package=glmmTMB].
58. Dinno A. dunn.test: Dunn's Test of Multiple Comparisons Using Rank Sums 2024 [Available from: https://cran.r-project.org/package=dunn.test].
59. Bartoń K. MuMIn: Multi-Model Inference, R package 2023 [Available from: https://CRAN.R-project.org/package=MuMIn].
60. Hartig F, Lohse L. DHARMa 0.4.6 R package 2022 [Available from: https://CRAN.R-project.org/package=DHARMa].
61. Lüdecke D, Makowski D, Ben-Shachar MS, Patil I, Waggoner P, Wiernik BM, et al. Performance 0.10.4 R package 2023 [Available from: https://CRAN.R-project.org/package=performance].
62. Engbæk K, Lawson PA. Identification of *Bartonella* species in rodents, shrews and cats in Denmark: detection of two *B. henselae* variants, one in cats and the other in the long-tailed field mouse. APMIS. 2004;112(6):336-41. doi: https://doi.org/10.1111/j.1600-0463.2004.apm1120603.x
63. Voller A, Bidwell DE. The effect of *Eperythrozoon coccoides* infection in mice on superimposed *Plasmodium chabaudi* and Semliki Forest Virus infections. Annals of Tropical Medicine & Parasitology. 1968;62(3):342-8. doi: https://doi.org/10.1080/00034983.1968.11686569
64. Feng SH, Lo SC. Induced mouse spleen B-cell proliferation and secretion of immunoglobulin by lipid-associated membrane proteins of *Mycoplasma fermentans* incognitus and *Mycoplasma penetrans*. Infection and Immunity. 1994;62(9):3916-21. doi: https://doi.org/10.1128/iai.62.9.3916-3921.1994
65. Harasawa R, Fujita H, Kadosaka T, Ando S, Rikihisa Y. Proposal for '*Candidatus* Mycoplasma haemomuris subsp. musculi' in mice, and '*Candidatus* Mycoplasma haemomuris subsp. ratti' in rats. International Journal of Systematic and Evolutionary Microbiology. 2015;65(Pt_2):734-7. doi: https://doi.org/10.1099/ijs.0.069856-0
66. Millán J, Di Cataldo S, Volokhov DV, Becker DJ. Worldwide occurrence of haemoplasmas in wildlife: Insights into the patterns of infection, transmission, pathology and zoonotic potential. Transboundary and Emerging Diseases. 2021;68(6):3236-56. doi: https://doi.org/10.1111/tbed.13932
67. Smith DD. The Sarcocystidae: *Sarcocystis, Frenkelia, Toxoplasma, Besnoitia, Hammondia*, and *Cystoisospora*. The Journal of Protozoology. 1981;28(2):262-6. doi: https://doi.org/10.1111/j.1550-7408.1981.tb02848.x
68. Sanchez SG, Besteiro S. The pathogenicity and virulence of *Toxoplasma gondii*. Virulence. 2021;12(1):3095-114.
69. Arendt M, Stadler J, Ritzmann M, Ade J, Hoelzle K, Hoelzle LE. Hemotrophic Mycoplasmas—Vector Transmission in Livestock. Microorganisms. 2024;12(7):1278. doi: https://doi.org/10.3390/microorganisms12071278
70. Gray J, Kirstein F, Robertson J, Stein J, Kahl O. *Borrelia burgdorferi* sensu lato in *Ixodes ricinus* ticks and rodents in a recreational park in south-western Ireland. Experimental & applied acarology. 1999;23:717-29. doi: 10.1023/A:1006233700194

71. Gray J, Robertson J, Key S. Limited role of rodents as reservoirs of *Borrelia burgdorferi* sensu lato in Ireland. European Journal of Epidemiology. 2000;16:101-3. doi: 10.1023/A:1007642908546
72. Telfer S, Bown KJ, Sekules R, Begon M, Hayden T, Birtles R. Disruption of a host-parasite system following the introduction of an exotic host species. Parasitology. 2005;130(6):661-8. doi: 10.1017/S0031182005007250
73. Zintl A, McManus A, Galan M, Diquattro M, Giuffredi L, Charbonnel N, et al. Presence and Identity Of *Babesia mMicroti* In Ireland. Available at SSRN 4373187. 2023. doi: 10.1016/j.ttbdis.2023.102221
74. Poulin R, Morand S. The diversity of parasites. The quarterly review of biology. 2000;75(3):277-93. doi: 10.1086/393500
75. Chisholm RA, Fung T, Chimalakonda D, O'Dwyer JP. Maintenance of biodiversity on islands. Proceedings of the Royal Society B: Biological Sciences. 2016;283(1829):20160102. doi: 10.1098/rspb.2016.0102
76. Zintl A, Moutailler S, Stuart P, Paredis L, Dutraive J, Gonzalez E, et al. Ticks and Tick-borne diseases in Ireland. Irish Veterinary Journal. 2017;70(1):1-10. doi: 10.1186/s13620-017-0084-y
77. Boulanger N, Boyer P, Talagrand-Reboul E, Hansmann Y. Ticks and tick-borne diseases. Medecine et maladies infectieuses. 2019;49(2):87-97. doi: 10.1016/j.medmal.2019.01.007
78. Kirstein F, Rijpkema S, Molkenboer M, Gray J. The distribution and prevalence of B. *burgdorferi* genomospecies in *Ixodes ricinus* ticks in Ireland. European journal of epidemiology. 1997;13:67-72. doi: 10.1023/A:1007360422975
79. Zintl A, Zaid T, McKiernan F, Naranjo-Lucena A, Gray J, Brosnan S, et al. Update on the presence of *Ixodes ricinus* at the western limit of its range and the prevalence of B*orrelia burgdorferi* sensu lato. Ticks and Tick-borne Diseases. 2020;11(6):101518. doi: 10.1016/j.ttbdis.2020.101518
80. Flattery A, McKiernan F, Browne J, Gray J, Zaid T, O'Connor J, et al. The prevalence and distribution of *Anaplasma phagocytophilum* genotypes in *Ixodes ricinus* nymphs collected from farm- and woodland sites in Ireland. Ticks and Tick-borne Diseases. 2022;13(3):101928. doi: 10.1016/j.ttbdis.2022.101928
81. Colautti RI, Ricciardi A, Grigorovich IA, MacIsaac HJ. Is invasion success explained by the enemy release hypothesis? Ecology letters. 2004;7(8):721-33. doi: 10.1111/j.1461-0248.2004.00616.x
82. Eccard JA, Mazza V, Holland C, Stuart P. The timid invasion: behavioural adjustments and range expansion in a non-native rodent. Proceedings of the Royal Society B. 2023;290(2003):20230823. doi: 10.1098/rspb.2023.0823
83. White TA, Perkins SE, Heckel G, Searle JB. Adaptive evolution during an ongoing range expansion: the invasive bank vole (*Myodes glareolus*) in Ireland. Molecular Ecology. 2013;22(11):2971-85. doi: 10.1111/mec.12343
84. Bouilloud M. Impact De L'anthropisation Sur Les Liens Entre Les Communautés De Petits Mammifères, Leur Microbiote Et Les Dangers Zoonotiques: PhD Thesis, Institut agro, Montpellier; 2023.
85. Eliot CP. The insect vector for the natural transmission of *Eperythrozoon coccoides* in mice. Science. 1936;84(2183):397-. DOI:10.1126/science.84.2183.397.a
86. Crystal MM. The mechanism of transmission of *Haemobartonella muris* (Mayer) of rats by the spined rat louse, *Polyplax spinulosa* (Burmeister). The Journal of parasitology. 1958:603-6. doi: 10.2307/3274543
87. Brian JI, Catford JA. A mechanistic framework of enemy release. Ecology Letters. 2023.;26(12):2147-66. doi : 10.1111/ele.14329
88. Kelly TC, Sleeman DP, Fennessy GJ, Dillon A, Walton GA, Gray J. The Mammal Ticks (Acari: Ixodoidea) of Ireland: Their Distribution and Hosts. The Irish Naturalists' Journal. 2001;26(10):363-70.
89. Haukisalmi V, Henttonen H, Tenora F. Population dynamics of common and rare helminths in cyclic vole populations. The Journal of Animal Ecology. 1988:807-25. doi: 10.2307/5094

Formatted: Font: Italic

Formatted: Font: Italic

Formatted: Font: Italic

Formatted: Font: Italic

Formatted: Font: Italic

Formatted: Font: Italic

Formatted: Font: Italic

Formatted: Font: Italic

Formatted: Font: Italic

Formatted: Font: Italic

Formatted: Font: Italic

Formatted: Font: Italic

Formatted: Font: Italic

90. Laakkonen J, Oksanen A, Soveri T, Henttonen H. Dynamics of intestinal coccidia in peak density *Microtus agrestis, Microtus oeconomus* and *Clethrionomus glareolus* populations in Finland. Ecography. 1998;21(2):135-9. doi: 10.1111/j.1600-0587.1998.tb00667.x

91. Voutilainen L, Kallio ER, Niemimaa J, Vapalahti O, Henttonen H. Temporal dynamics of Puumala hantavirus infection in cyclic populations of bank voles. Scientific Reports. 2016;6(1):1-15. doi: 10.1038/srep21323

Supporting Information

Supplementary Table 1 – Field work dates. This details the date in which field work was conducted

Supplementary Table 2 – Prevalence summary region and year. This provides a summary of the pathogen prevalences per host species for each invasion region and year during this study.

Formatted: Font: Italic

Formatted: Font: Italic

Formatted: Font: (Default) Times New Roman

Formatted: Font: (Default) Times New Roman

Data Review URL

Click here to download Data Review URL
http://zenodo.org/records/14883529
