# Supplementary material for: Longitudinal study of a rodent invasion reveals shifts in host pathogen interactions
Source: PLoS Pathog. 2026 Jul 23;22(7):e1014349. doi: 10.1371/journal.ppat.1014349 (PMC13395368; doi:10.1371/journal.ppat.1014349)
Supplement: Attachment — Submitted filename: Authors reply to reviewers.docx [file ppat.1014349.s005.docx]

Dear Editor and Reviewers,

We would like to thank you for taking the time to review our manuscript. All three reviewers comments have been consolidated below, with a numbering system attached in order to aid clarity. We have attached how we addressed your comments, and our replies in red below. Line numbers mentioned in our replies reflect the “tracked changes” document.

Yours faithfully,

The Authors

**Reply to Reviewers' Comments:**

Reviewer's Responses to Questions

**Part I - Summary**
Please use this section to discuss strengths/weaknesses of study, novelty/significance, general execution and scholarship.

Reviewer #1: Dear authors,

**1.** I was pleased to review this manuscript on the hidden dynamics of pathogen transmission in a biological invasion context. I think that the longitudinal study is extremely interesting and certainly deserves publication after a few issues are resolved.
My concerns are mainly about the statistical analyses, I have left detailed comments in the pdf attached, but I think that the methods are slightly confusing and lack of an input form an expert in statistics, which could improve the explanation and the rationale behind the chosen methodology and the reporting of the results. That said, I do not think the methodology is wrong, just needs to be reported in a better way, and there are some things to be clarified (see the comments in the document attached).

Thank you for taking the time to thoroughly review the manuscript and we are glad you agree it is worthy of publication. Thanks also for your useful suggestion on how to improve the manuscript. The statistical analysis section has been reworked to improve clarity as per comments 53, 57, 59 and 70. We have addressed these comments you and other reviewers have left on the manuscript here, please see the list below below:

**2.** Line 27: “were sampled” – comment “what do you mean sampled? removal trapping? which samples were collected?”

Replaced with: “were sampled, via removal trapping,” on line 28.

**3.** Comment: “specify the matrix otherwise 10 OTUs seem reductive”

The sequencing of spleens from the 1,082 rodents from Ireland presented in this study was carried out between 2016 and 2022, sometimes including samples from other countries outside the scope of this study, and the sequencing data were analysed in three stages over different periods. Details of the sequencing process are presented in the file ‘Sequencing_informations_spleen_samples.xlsx’ on Zenodo (<https://zenodo.org/records/14883529>). Thus, for the three batches of samples, we obtained:
-    2016, Batch No. 1 (379 samples): total of 761 OTUs representing 5,212,747 reads, including 10 putative pathogenic OTUs representing 4,604,340 reads (88% of the reads)
-    2017, Batch No. 2 (189 samples): total of 401 OTUs representing 5,099,867 reads, including 8 putative pathogenic OTUs representing 4,774,704 reads (93% of reads)
-    2022, Lot No. 3 (672 samples): total of 1,272 OTUs representing 10,566,359 reads, including 10 putative pathogenic OTUs representing 8,442,958 reads (80% of reads)
We believe that these details would considerably weigh down the description of the results, so we have decided not to include these numbers in the manuscript.

**4.** Line 31: “Several epidemiological processes were found to be correlated with the *C. glareolus* invasion.” – Comment “example? it is too vague even for an abstract”

This was rephrased so it links to the epidemiological processes that are described subsequently, with “firstly” on line 32 and “secondly” on line 34.

**5.** Strikethrough removing the second full stop on line 52.

This has been removed.

**6.** Line 66: “This can happen because the pathogen, or their vector, is absent from the founder population, or because the environment in the new range is unsuitable (19-23). This, in turn, may give invaders a competitive advantage over native species.”

Comment: “I think this should be slightly rephrased for clarity and expanded for readers who are not familiar with epidemiology. Also, maybe would be nice to add the publication where this theory was developed for the first time”

This was subsequently rephrased and and the Keane and Crawley (2002) and Torchin et al., 2003 references were added on lines 64-69, now reading: “However, non-native species can also benefit from a reduction in natural enemies, such as predators or pathogens, compared to conspecifics in their native range (and often compared to the native species), through a process known as “enemy release”(19, 20). In relation to pathogens, this can happen because the pathogen, or the vector responsible for its transmission, is absent from the founder population, or because the environment in the new range is unsuitable to sustain the pathogen or vector (21-25).”

**7.** Strikethrough removing “including among others” and replacing with “such as” on line 80.

This was accepted and now reads “such as” instead on line 82 of the tracked changes document.

**8.** Line 82: “this is a little awkward, please rephrase. maybe expand just a liitle bit”

This was expanded to provide extra clarification, and now reads as: “Finally, studies on rodent invasions have demonstrated the interplay between rodent pathogen interactions and biological invasions, for example, helminth studies in both Ireland and Senegal have observed patterns of enemy release in invasive rodents when comparing sites of long established populations to more recently invaded sites (24, 31, 38, 39).” On lines 83-86.

**9.** Strikethrough removing “this is and replacing with a comma on line 88.

Line 86: insert “only” in this sentence.

These suggestions were accepted and inserted on lines 88/89, now reading: “(37), particularly due to the depauperate woodland rodent community in Ireland, that includes only the invasive *C. glareolus* and the native woodmouse, *Apodemus sylvaticus* (40).”

**10.** Line 91, comma inserted after Co. Limerick

Comma was inserted in the text here on line 94.

**11.** Line 99: “this paragraph is slightly clunky, maybe try to improve the connection among sentences; although, I understand what you are saying and it is correct”

This paragraph has been rephrased to improve connection among sentences starting on line 104, now reading as:

“The development of high-throughput sequencing, and in particular metabarcoding techniques, facilitates a more holistic approach to studying the rodent pathobiome (44). These methodologies provide a global picture of pathogen diversity within host species, improving our understanding of the relationship between host-parasite interactions and biological invasions. In the Irish context, rodent helminth parasite communities have been explored previously by Loxton et al. (23, 31) and Stuart et al. (39). However, no such data exists on pathogenic microparasites in this model system. Furthermore, there remains a knowledge gap concerning longitudinal studies focusing on changes in pathogen communities during biological invasions. Such studies are essential for fully exploring changes in host-pathogen interactions, such as pathogen loss in invasive species, as cross-sectional studies only capture a single timepoint of this dynamic process (37, 39, 45-47).

In this study, we aim to address these knowledge gaps by describing the community of microparasites that could be zoonotic or pathogenic for rodents, including bacteria and protozoa, of *C. glareolus* and *A. sylvaticus* in Ireland, through a longitudinal study.”

**12.** Line 111: the subject is rodents? if so, and rodents sampled....were subsequently sampled in 2017, 2021, and 2022.

Rephrased to acknowledge this on lines 116-118: “We performed a spatiotemporal analysis of this community along the invasion gradient described above, using rodents removed in 2016 from a previous study by Stuart et al. (39), with subsequent removal sampling at these sites in 2017, 2021 and 2022.”

**13.** Line 111, comma after “in this study” and line 112 “recently used by”

Comma and wording suggestions were inserted on lines 118-120, now reading: “In this study, we use the 16S rRNA amplicon metabarcoding approach developed by Galan et al. (48), and recently used by Bouilloud et al. (49).”

**14.** Line 114-119: “non need to capitalise firstly. I would use semicolon to separate the hypotheses with some sort or numbering (e.g. i, ii, iii, or any of your choice)” and “in the first hypothesis, pathogenic for the vole? or zoonotic?”

The hypotheses have been restructured as you suggested on lines 121, 123 and 125. Furthermore, the first hypothesis relates to all pathogenic species, hence the terminology “patterns consistent with the enemy release hypothesis”, the pathogenicity of pathogens found are later described in the results with improved references as per your comments.

Now reads as: “Focusing on putative pathogenic taxa recorded in samples collected between 2016 and 2022, this present study addresses three main hypotheses: (i) whether *C. glareolus* exhibits patterns consistent with the enemy release hypothesis, including a lower pathogen species richness of potentially pathogenic taxa when compared to its native range and the native *A. sylvaticus*; (ii) whether the presence of *C. glareolus* alters the pathogen communities present in the native *A. sylvaticus* as expected under the dilution or spillback hypotheses; finally (iii) whether pathogen prevalence changes over time in both rodent species, as the invader becomes established.”

**15.** Line 121: “this section might benefit a reorganization of the sections, splitting it into defined data/sample collection, molecular data analysis, statistical analysis

also, it is less well written than the introduction. it is difficult to follow at times, and there are redundant sentences, especially the stat section need same polishing”

Rephrased and reorganised the methods section entirely (lines 128-261), see your comment below for details.

**16.** Line 132: “it needs to be clear that is removal trapping, and all ethical and technical aspects related to removal should be mentioned (or refer to the methods in another publication)”

Rephrased on line 141 to read: “A maximum of 24 animals from each species were removed from each site per season and year of sampling.”

As for the ethics, this is stated clearly in the designated section as per the publishers formatting on lines 820-824 on the tracked changes document.

**17.** Line 165: I know that there are references, but it be worth expanding this a little bit to specify how and why you filter which (presumed) pathogens

This has been rephrased to clarify they were identified based on their taxonomic affiliations on lines 169-179, now reading: “From the clusters of microorganism DNA detected, putative pathogenic OTUs were filtered and selected (51). First, we discarded putative false positive results associated with sequence counts below two OTU-specific thresholds, which checked respectively for putative cross-contamination between samples during DNA extraction and PCR steps on the one hand, and incorrect assignment due to the generation of mixed clusters on the flowcell during Illumina sequencing on the other hand (52). Then, for each sample, and each OTU, we kept only the occurrences confirmed by the two PCR replicates of the same sample. Finally, the putative pathogenic OTUs were selected using their taxonomic affiliations and based on their potential zoonotic risk and pathogen impact on rodents, to separate them from commensal species (51).”

**18.** Line 165: so, what did you compare? the overall OTUs? which test? did you have the raw data from France?

This has been clarified that no statistical analysis could be conducted due to differences in how the data and rodents were processed on lines 193-201, now reading: “Firstly, bacterial data collected from *C. glareolus* and *A. sylvaticus* in Ireland from 2021 was compared to data collected from rodents trapped in forest sites in France, sampled in 2021 by Pradel et al. (56), using the same 16S metabarcoding approach (48). These data were examined by comparing prevalences of potentially pathogenic bacterial taxa in rodents from Ireland and France, to examine if less species were detected and at a lower prevalence in Ireland, related to the enemy release hypothesis. However, due to differences in assigning age and maturity to animals in each country, statistical analysis could not be conducted. Likewise, while the bacterial pathogens were filtered using the same methodology, two extra species of potentially pathogenic Sarcocystidae were included in the Irish data, and consequently meant that these species could not be included in the comparison.”

**19.** Line 172: is that reported elsewhere? do you think you might have missed something interesting or important?”

This has been replicated in studies by Diagne et al. – The logic in this is that prevalences below 5% would fall into the margin of error and proper statistical analysis was not feasible. In this instance, the prevalences below 5% are a handful of individual positives out of the entire dataset and statistical analyses would be extremely zero inflated. In this study, 498 *C. glareolus* and 584 *A. sylvaticus* were sampled across four years, therefore, we are confident that all patterns of infection were explored.

**20.** Line 183: “is this the same analysis as the comparison with French data?”

Clarified above that no statistical analysis was conducted. This section has also been rephrased to improve clarity as per comment 53 below.

**21.** Line 187: “baseline time should be T0, but 1 is also fine, bu it would be clearer to keep the variable name year and then use T1, T2, etc..”

This has no impact on the analysis or interpretation, however baseline as year 1 was chosen to avoid any complications regarding zero inflation should it have arisen.

**22.** Line 189: “I think I get it but it is a little awkward and also, as this percentage is not included in the models it is confusing here. What was the rationale behind this analysis?” and line 196: “What do you mean?”

Invasion region as a category was used as a proxy for the “percentage of *Apodemus* relative to C. glareolus” in order to quantify the effects of the invasion gradient, as the bank vole overtakes the woodmouse in numbers. Hence the assigned value for percentage *Apodemus* to each sample was tested against region to verify if our classification of region holds true for the analysis, this has been rephrased and moved to a better location on lines 246-255 to provide clarity:

“These analyses used the categorical variable of region as a proxy for invasion levels *of C. glareolus* relative to *A. sylvaticus* to aid in the interpretation of the results and increase statistical power through increased number of samples per variable. In order to justify our classification of invasion region in the analysis, the percentage of *A. sylvaticus* relative to *C. glareolus* was tested against invasion region. For these analyses, the total number *A. sylvaticus* relative to *C. glareolus* trapped in each site in each season within a given year was calculated, generating a percentage of *A. sylvaticus* relative to *C. glareolus*. These numbers were then assigned to each sample corresponding to their trapping site, season and year. Finally, to justify the classification of regions, this assigned percentage of *A. sylvaticus* relative to *C. glareolus* in the dataset was tested against region using a Kruskal-Wallis test, and a post-hoc Dunn’s test, through the dunn.test R package (58), to check differences between regions in both 2016 only, and the longitudinal dataset, as the data followed a non-normal distribution.”

**23.** Line 206: “This not clear to me”

In the case where GLMMs could not be fitted, non-parametric tests were conducted, where the data does not follow a normal distribution. This was the case with the pathogen species richness models; hence non-parametric tests were used to verify model findings.

This clarification was added on line 242 and now reads “In cases where no GLMM could be fitted, non-parametric tests were used, such as a Wilcoxon Rank Sum test.”

**24.** Line 210: “It would be good to include the formulation of the model to highlight the response variable and the distribution used (I see they are in the text, but an equation or even the R prompt would be great for clarity)”

Adding these would introduce complexity to table where the intent of this figure was to provide a summary of the factors and portions of the datasets used in each analysis (i.e. a reference for all factors investigated when interpreting the results section) and has been left as such. The model formation is specified in the text itself, as different response variables/distributions are used depending on the data being analysed, e.g. negative binomial for pathogen species richness and binomial for pathogen prevalence. The response variables and distributions used are explained in lines 203-212. While the strategy for each analysis (invasion gradient and longitudinal) are explained from lines 213 to 245. Additionally, the distribution family used for each GLMM is also repeated in the results section to aid the reader.

**25.** Comment on same table: “this is a result”

Sample sizes removed and put with the model summary table 3 on line 289 and explanation added below on line 292: “n represents sample sizes in the full model for each analysis”.

**26.** Line 215: “once the methods are organised please follow the same order for the results

maybe the overall section is a bit too long”

This has been rephrased and restructured, the new section is broken up into each analysis and then the results within it to help break up the text better. A detailed description of the restructuring is provided below in comment 53.

**27.** Line 223: “maybe in the intro you should cite some studies demonstrating that voles tend to overtake mice population to understand this point”

This has been added with appropriate references on lines 97-101: “Furthermore, previous studies have shown a reduced relative abundances of *A. sylvaticus* at sites invaded *by C. glareolus* (39, 43). This has allowed for the establishment of cross-sectional studies with an invasion gradient, containing sites from the invasion core (established from the introduction point, where *C. glareolus* exhibits it’s highest relative abundance), invasion front (sites where *C. glareolus* had most recently invaded), and uninvaded sites (*A. sylvaticus* only) (39).”

**28.** Line 229: “this shows it the other way round compared to the methods. also does 100% is the total population of voles+mice or only mice? this figure is unclear to me”

The data itself was analysed as percentage of *Apodemus* relative to *C. glareolus* as this was the measure we used to identify how invaded a site had become. However, it was chosen to display the progress of the *C. glareolus* invasion using the percentage of *C. glareolus*, relative to *A. sylvaticus* (the direct inverse). This more clearly shows how invaded the regions were as 0% *C. glareolus* is more straightforward than 100% *A. sylvaticus.* Additionally, the 100% represents the total rodent community, i.e. at the core it is dominated by *C. glareolus* and approximately 75% in 2022, the remaining approximate 25% are *A. sylvaticus*. Therefore, the front 2021 in 2016 showing 0% *C. glareolus* means the site is 100% *A. sylvaticus.*

**29.** Line 236: “references? also these adds up to 9”

This has been rechecked, and these add up to 10 OTUs. References have been added for the pathogenicity of the mycoplasmas: “These included one OTU of the genus *Bartonella*, a bacterial genus potentially including zoonotic species (e.g. *B. henselae*) (62), three OTUs of *Mycoplasma haemomuris* (combined for analysis), one OTU of *Mycoplasma coccoides* and one OTU of *Mycoplasma penetrans* (known to be zoonotic). These three *Mycoplasma* species have traditionally been associated with pathology in rodents (63-65). While more recent studies suggest that *Haemoplasma* may be largely subclinical in wildlife they have also highlighted the need for further studies increasing their geographic range and elucidating transmission (66). Finally, we detected four OTUs of Sarcocystidae (combined for analysis, a family of protozoans, with some species that are zoonotic, and others that are pathogenic for rodents) (67, 68).

**30.** Line 247: “this recurrent terminology is very vague and needs to be clarified in the methods (see relative comment)”

The term potentially pathogenic is used due to the small fragments of DNA from the 16S methodology. The terminology itself has been clarified in the methods section and used consistently throughout to help clarity: “described by Galan et al. (48). From the clusters of microorganism DNA detected, putative pathogenic OTUs were filtered and selected” and “Separate putative pathogenic OTUs of the same pathogen species were combined into potentially pathogenic taxa for analysis” on lines 169 and 183.

**31.** Line 253: this table is not the best way to report modelling results. information regarding metrics usually used to assess model fit and variance explanation proportion are missing. also the table is not cited in the text

This summary table is intended as a brief summary of the statistically significant factors in a concise table, allowing the reader to use this as reference for subsequent results and discussion sections. The Z, P and AICc values are added throughout the text to help aid these results, likewise the table is now referenced throughout the results section, including adding “In the autumn only analysis, the best model included only region (lowest AICc value; 322.788), however, no factor significantly influenced *Bartonella* prevalence.” to line 533-534, to further clarify the model.

**32.** Line 260: this section should be put before the modelling results, and the latter need to be further expanded beyond table 3. Also, figure 3 needs asterisks (or another appropriate symbol to visualise significant differences among each of the coupled bars)

The results section has been restructured to bring the pathogens found to the top of the results section, with the comparison with France directly below this. Asterisks cannot be added as statistical analysis was not conducted as per comments on the statistical analysis section. The comparison with the French samples is still left below the modelling table 3 as this is a summary of the overall results found in the Irish samples and is most appropriate at the top of the results section. However, the enemy release section is discussed before the modelling results themselves for pathogen species richness and pathogen prevalence in each host spp.

**33.** Line 277: maybe this section would be appropriate for a restructured table 3

The entirety of the results section has now been restructured to reflect the statistical analysis section. Table 3 has been left as is, so as to provide a simple summary for readers as per above comment.

**34.** Lines 278 and 286: “it is redundant to present both” and “again showing both is redundant, and maybe, if there is poot fit, the problem lies in the structure and type of data, you do not solve it changing test.”

Both were kept as the final models were observed to show poor fit, therefore non-parametric tests were used to confirm our findings (following tests of normality for the data). See comment above regarding non-parametric tests.

**35.** Line 294: “these results (all sections regarding statistical analyses) might have been better displayed using a table like table 3 (which should be mentioned here I guess), but with AIC, z and p values for each model and each factor” and “the reporting of statistical results is confusing and difficult to follow, I am not sure the methodology fully matches the reported results. the methods and results section regarding statistical testing, although might not be entirely wrong might benefit from a review from an expert in statistics. I think some tests are redundant and the result section might be streamlined with better tables and figures”

These have been explained as text in order to help understand the differences between individual groups, i.e. it can be explained if juveniles are more infected, or which region etc. The table only provides a summary of the statistically significant factors to aid as a reference for the discussion, as the results are quite long. The statistical analysis section has been reorganised alongside the results section to reflect the same order and layout to improve clarity.

For example, the section on *Bartonella* during the invasion gradient now reads as: “Considering the invasion gradient analysis, the best model (lowest AICc value; 271.876) included season and sex (Table 3). *Bartonella* prevalences were higher in autumn compared to spring (GLMM, family = Binomial, z = -2.741, p = 0.00612), while males were more infected than females (GLMM, family = Binomial, z = 2.404, p < 0.05).”

**36.** Line 303: Why not keep just one of the two?

Both models were kept due to the nature of these data being analysed; 2017 and 2022 only have a single season, consequently these variables were inherently linked. Consequently, a high VIF was observed with the interaction between the two variables. Therefore, separate models were run on each season so the effect of time during spring and the effect of time on autumn data could be explored, which did show different patterns of infection, based on the season being focused upon.

**37.** Line 337/338: “please place significance letters oor symbols on top of the bars” and strikethrough on “(Maximum and Minimum)”

The asterisks have been added to the factor that is statistically significant, on line 551 of the tracked changes document. Likewise, the maximum and minimum has been removed on each figure but a single clarification added to the statistical analysis section instead to make up for this, on line 186: “Confidence intervals (maximum and minimum) were calculated to 95% using the R package PropCIs (55).” This has also been done for the Sarcocystidae figure 4 on line 476.

**38.** Line 340: “same comments apply here, also for the figure” and “I do not understand why you did GLMM, correlation, non parametric tests, post-hoc etc. all together”

The figure has been kept as described below relating to seasonal figures. Asterisks weren’t added as the significant factor relates to time, which is shown per year and season here, to reflect that some years only contain one season’s data.

Post-hoc tests were used to determine the differences between groups with more than two variables. In the case of the correlation test, this was done post-hoc to show the correlations of the levels of certain pathogens with time.

**39.** Line 360: “and the other pathogens? table 2 showed some prevalence”

Prevalences are below 5% and in the case of this dataset, that is only a handful of individuals, therefore it would be inappropriate to conduct statistical analyses on these pathogens (as per comment 19.

**40.** Line 364: “overall the section is difficult to follow, maybe too much was tried to convey, while more target results, functional to the hypotheses would have been better. Another option would be to summarise the results in some tables”

The section has been split up based on the new results section structure as per comments below into invasion gradient and longitudinal analysis. Splitting this up should break up the results to allow for better understanding. Likewise, the statistically significant results are summarised in Table 3.

As with previous comments, the statistical analysis was chosen based on the hypotheses, while confounding factors still need to be considered, as season, sex, age etc can all have effects on pathogen prevalence and need to be fully explored to determine if the hypotheses hold true.

**41.** Line 415: interesting, good references, maybe need a little bit of restructuring and streamlining to convey the main messages more effectively.

Rephrasing and streamlining of text in the discussion section from lines 608 to793 to improve flow. Your specific comments on this section have been addressed through the comments directly below:

**42.** Line 416: a bit clunky, can be rephrased to improve flow and Line 422: the number should suffice

This has been rephrased, now on line 609-617 on the tracked changes document, to improve clarity and reduce clunkiness. The text and date for Zintl et al. (2023) was removed and reference number kept also:

“The aim of this study was to investigate the effect an invasive species may have on native pathogen communities, including zoonotic species, using the bio-invasion of Ireland by C. glareolus as a model system. While previous studies on rodent-borne microparasites in Ireland have focused on single bacterial species, such as Bartonella or Borrelia (70-72), this is the first time 16SrRNA metabarcoding techniques have been used to identify the full communities of potentially zoonotic pathogens, and their prevalence levels, that are circulating in Irish populations of C. glareolus and A. sylvaticus. Furthermore, this study has created an important baseline for rodent-borne disease studies in Ireland, and highlights new important areas of research to guide future research in Ireland, and potentially other invasion systems (e.g (73)).”

**43.** Line 424: you keep changing names of sub-sections and combinations of words such as pathogen risk, zoonotic risk, infectious risk, etc. this is confusing. it is especially confusing whehter your focus is on pathogens pathogenic for rodents or zoonotic pathogen considered a human health risk. I suggest keep a coherent nomenclature throughout the manuscript and restructuring the sub-section headlines so that they match as much as possible in the methods, results, discussion, etc

The subsection here has been renamed to “Rodent-borne pathogens observed in Ireland” to clear up confusion on line 619. Additionally, this sub section has been kept in this location, as this is the logical first step to discuss what pathogens are observed in irish rodents. Followed by subheadings specific to the topics hypothesised, i.e. patterns of enemy release, whether the bank vole has an effect on the wood mouse pathogens, and finally whether time (as the bank vole becomes more established in Ireland) has an impact.

**44.** Line 426: do you have a reference? if you are mentioning France, then add the reference

This is a general statement based on the findings of the paper, we only detected 5 pathogen species in Irish rodents, therefore no reference is provided. In the next paragraph the comparison with the French samples is discussed.

**45.** Line 429: this is in the next section, why also here?

This sentence reading “Furthermore, we observed that the *C. glareolus* population in Ireland expressed patterns consistent with the enemy release hypothesis, and potentially disrupts the transmission of native pathogen species.” has since been removed to avoid repetition on lines 624-626.

**46.** Line 439: so not the effect of invasive voles?

Yes, while the bank vole was shown to potentially impact the prevalence of *M. haemomuris,* the depauperate community already present in the native wood mouse, would most likely be due to Ireland being an Island. Likewise, this would also possibly explain the lack of pathogens picked up by the bank vole.

**47.** Line 443: I think this is a missed opportunity for a greater pathogen diversity assessment

While we accept this is a limitation of the study, the data for this paper serves as a baseline for future work outlined in the discussion, and as discussed in papers like Zintl et al., 2023. The 16S methology was chosen in order to get a broad picture of all the potential pathogen species infecting the rodents involved in this invasion system, and to fully understand the patterns in these pathogen communities caused by the bank vole invasion (see comment 57 below for detailed explanation).

**48.** Line 458: please rephrase, I am not sure I understand

This was meant to explain that patterns consistent with the enemy release hypothesis were observed between the bank vole and wood mouse in Ireland, and between the bank vole in Ireland and its native range (France). This has been clarified by removing the phrase altogether and explaining the different patterns of enemy release, now reading as; “Two patterns consistent with the enemy release hypothesis were observed for bacterial pathogens in the invasive *C. glareolus* population in Ireland for the first time. Firstly, a comparison of the number of bacterial taxa in Irish and French *C. glareolus* populations indicated that the population of *C. glareolus* sampled in Ireland had fewer potentially pathogenic bacterial taxa than conspecifics in their native range.” on line 653-656 on the tracked changes document.

**49.** Line 514: the main verb is missing, do you meant resulted instead of resulting?

This has been fixed by adding “was observed” on line 711, now reading: “However, no dilution or amplification effect resulting from the *C. glareolus* invasion gradient on pathogen species richness in *A. sylvaticus* was observed in the present study.”

**50.** Line 554: this is the best section of the discussion and the most interesting, I would expand a bit. To make it more relevant I would very briefly mention the significance of each pathogen (effect on rodent survival, zoonotic potential, etc.)

While we appreciate that these are interesting topics, we worry that expanding this discussion would take away from the main messages/hypotheses of the paper, which is how invasive species, and time since invasion can have significant impacts on pathogen communities. Furthermore, we think that cross-sectional studies are not always sufficient to fully tease out the complex epidemiological processes during these invasions.

**51.** Line 580: careful that Sarcocystidae are not bacteria

This has been clarified on lines 776-778 that Sarcocystidae were not included in the comment regarding bacteria by changing the phrasing, now reading as: “Despite exhibiting higher prevalences of Sarcocystidae, *C. glareolus* in Ireland shows reduced pathogen species richness, and lower prevalences of bacterial taxa, compared to the native *A. sylvaticus* and fewer bacterial taxa compared to the native range of the species.”

**52.** Line 639: please double check the formatting, as there are some typos and minor errors, in particular most species names are not italicised (I highlighted some but not all), as well as capitalisation where is not needed and other minor mistakes

Thank you for pointing out these formatting errors. We have rectified these in the reference list through lines 836 to 1091. This included lowering sentence case where all caps had been inserted, making species names italic and the end bracket from the links to R packages (“]”).

In the case of reference 8, Daszak et al. the double hyphen is how the paper is titled, and the reference is correct.

The tracked changes include some details that were in the original submission (missing DOIs, some incorrect page numbers for references 24, 39 and 87, a missing “PhD Thesis” from 84, and formatting the indent to tidy up the reference block), that were added back in, as the endnote reference manager reformats every time new references were added.

**53.** Then, I think that the structure of methods, results, and consequently discussion should be improve to streamline the main message, and the sub-sections need to be restructured/reordered so that the reader can follow the different aspects through the various section of the manuscript.

Sub sections have been added and structured to provide clarity, including rephrasing of sentences to provide clarity (see lines128 to 179 for methods, line 180 to 261 for statistical analysis). Additionally, the results section has been restructured to follow the order of the statistical analysis section and break up the results into an easier format (lines 262 - 607), as follows:

**Methods:**

Field work & sample collection

Laboratory work & analysis

**Statistical analysis**

Calculation of prevalence and species richness

Examining patterns of the enemy release hypothesis

Examining patterns of infection in Irish rodents

**Results**

Pathogens detected in Irish rodent spleen samples

Patterns of infection relating to the enemy release hypothesis

Validation of invasion region criteria for statistical models & progresss of *Clethrionomys glareolus* invasion

**Invasion gradient analysis**

(includes pathogen richness and host specific model results)

**Longitudinal analysis**

(includes host specific model results)

**54.** Hence, I also left comments for the images that sometimes can be improved to be clearer and more immediate.

Seasonal figure 7 have been removed, however, figure 5 was kept (now figure 6) as it shows the change in prevalence over time, while acknowledging the seasonal limitations of the 2017 and 2022 data. Grayscale has been kept for accessibility reasons (addressed in a comment 78 below).

**55.** Minor issues regard the precision of some statement, the formatting of the reference list and other minor comments that can be found in the pdf attached.

These formatting issues in the reference section have been addressed in the above comment 52.

All the details are reported in the comments in the document attached.
I hope that my comments will improve the quality of the manuscript and will give it the relevance it deserves.

**56.** Reviewer #2: This interesting manuscript investigates pathogen dynamics in an invasive rodent through a longitudinal study conducted in both core invasion zones and the invasion front. It also examines the relationships between the invasive species, its pathogens, and a native rodent species.

The article provides valuable insights into invasive species and their associated parasites/pathogens within the framework of the enemy release hypothesis. The analysis is robust, supported by a solid sample size and state-of-the-art pathogen detection methods. The study is also well contextualized through comparisons with data from France. The authors appropriately acknowledge the study’s limitations and outline relevant avenues for future research.

Thank you for taking the time to review out manuscript and we are glad you consider it a valuable contribution

**57.** Reviewer #3: Invasive species can influence epidemiological processes through a range of direct and indirect mechanisms, and gaining a better understanding of these dynamics is of clear value both theoretically and practically. McManus et al. have taken advantage of a unique "natural" experiment involving the recent spread of the bank vole (Clethrionomys glareolus) in Ireland to explore the epidemiological consequences of host invasion. Their study compares rodent-borne microbial taxa across a geographic gradient, including sites in France (the native range), as well as core and front areas of the invasion in Ireland. By incorporating both cross-sectional and longitudinal data, and by comparing patterns in a second rodent species (Apodemus sylvaticus) that is native to both regions, the authors aim to disentangle potential effects of invasion history on microbial communities. Their findings suggest patterns that may be consistent with enemy release and dilution effects, and they also highlight temporal dynamics in pathogen prevalence along the invasion gradient.

I commend the authors for their creative and ambitious study design, the integration of multiple data types, and the rigorous statistical approach. However, I have concerns about whether the manuscript meets the criteria of originality, impact, and scientific depth expected for publication in PLOS Pathogens, which emphasizes advances in pathogen biology. In my view, the primary limitations stem from the reliance on 16S rRNA metabarcoding in a relatively small and well-characterized host–microbe system. Although this method is valuable in more complex microbial communities and could serve as a preliminary tool to identify candidate species in this context, its application here leads to limited taxonomic resolution. As a result, it constrains the ability to draw confident conclusions regarding microbial identity, pathogenic potential, and ecological significance, particularly given the lower sensitivity of this approach. Additionally, the statistical analyses, though comprehensive, feel exploratory rather than hypothesis-driven, which makes it difficult to pinpoint key results and interpret them with confidence. As such, I am not fully convinced that the study yields novel or robust insights beyond those already offered by prior work focused on specific pathogens within this system. For these reasons, I recommend that the authors consider submitting their manuscript to a journal with a broader ecological or invasion biology focus. I offer detailed comments below in the hope that they will help strengthen the manuscript in its next iteration.

We understand that the 16S rRNA metabarcoding has its limitations, and that it is well characterised in rodent systems. However, in the case of the Irish rodent invasion system, this is the first time any large-scale pathogen screening has been done, aside from studies on helminth systems or smaller studies focusing on specific pathogens. The 16S approach has been successfully applied when investigating pathogen communities in other invasion systems, such as the studies by Diagne *et al.,* in Senegal. We chose 16S rRNA metabarcoding over other species-specific assays for individual pathogens, as this helps identify the potential pathogenic taxa in this current invasion system, while also allowing for analysis of pathogen communities as a whole, rather than focusing on a single pathogen species at a time. It is clear from our investigation that in a longitudinal study of an invasion, there will be winners and losers amongst the pathogens and a 16S approach allows for these simultaneous patterns to be detected. We believe this longitudinal survey during an invasion was a knowledge gap that needed to be filled. The knowledge generated will be useful to future studies of pathogens in invasional systems by providing valuable information helping narrow down which taxonomic groups to further investigate specific pathogens in. This has already happened as a result of the present study (e.g Zintl et al. 2023 study on *Babesia microti*)

Relating to the statistical analysis, while it may appear to be exploratory in nature, the statistical analysis was designed with a focus of describing the effect of the bank vole invasion on pathogen communities in the native wood mouse, and the effect of the invasion on the bank vole’s own pathogen species. We aimed to investigate both the effect of the *C. glareolus* invasion gradient, and the effect of time, as the invader becomes more established. Hence, we used separate analyses for these hypotheses; i.e. one using a single year where the invasion gradient was most prominent, with uninvaded *Apodemus* only sites; and a longitudinal study focusing on multiple years data but only the sites which had been repeated in 2021 and 2022. Finally, the statistical analysis also includes other confounding factors such as age, sex, seasonality etc. which have all been proven to influence pathogen prevalence in other systems and hence need to be included in order to fully understand the ongoing epidemiological processes in the Irish rodent system.

**Part II – Major Issues: Key Experiments Required for Acceptance**
Please use this section to detail the key new experiments or modifications of existing experiments that should be absolutely required to validate study conclusions.

Generally, there should be no more than 3 such required experiments or major modifications for a "Major Revision" recommendation. If more than 3 experiments are necessary to validate the study conclusions, then you are encouraged to recommend "Reject".

**58.** Reviewer #1: The methods section might benefit a reorganisation of the sub-sections, splitting it into defined data/sample collection, molecular data analysis, statistical analysis. Also, it is less well written than the introduction and it is difficult to follow at times, e.g. there are redundant sentences.

Addressed in points above.

**59.** The statistical analyses need to be reviewed, and so the relative results section. See the general comments and the comments in the document attached. The reporting of statistical results is confusing and difficult to follow, I am not sure the methodology fully matches the reported results. The methods and results section regarding statistical testing, although might not be entirely wrong might benefit from a review from an expert in statistics. I think some tests are redundant and the result section might be streamlined with better tables and figures.

We have attempted to address this by improving the clarity of the statistical analysis section, and streamlining the results to follow the same sequence (see above comments).

Additionally, in some instances, the analyses required splitting into “seasonal models” (where a high VIF was observed in the interaction between season and time). These were kept due to the differing patterns observed in the analyses of each season in isolation. Hence just removing the interaction and not acknowledging this would be inappropriate to describe all the ongoing epidemiological processes in this system.

Comments to the pdf have also been addressed individually above.

Reviewer #2: --

Reviewer #3: 1. Taxonomic identification and interpretation of the biology of microbes
**60.** o 1a. The focus on genus and family levels limits the capacity to draw meaningful conclusions about pathogenicity and the biology of the microbes. For instance, while all members of Sarcocystidae are parasitic, their clinical relevance varies. Some may cause subclinical or negligible effects, particularly in well-adapted hosts.

Due to the methods used, it is not possible to obtain species level taxonomic resolution for the *Bartonella* or Sarcocystidae species present. Consequently, the limited ability to draw conclusions from the taxonomic resolution has been addressed throughout the discussion, furthermore we have identified future work which would be required to verify the drivers behind these patterns observed in this study. As noted previously, while the 16S approach does not always provide species-level identification, it offers a broad overview of putative pathogens present in the samples. This breadth enables the identification of simultaneously occurring processes and helps to narrow down the range of specific pathogens for targeted investigation in future studies.

**61.** o 1b. Some statements regarding pathogenicity may require further support. For example, existing literature suggests limited pathogenic effects of Bartonella and Mycoplasma spp. in rodent hosts. If the authors are aware of studies suggesting otherwise, citing them would provide a more balanced view.

*Bartonella* was described as zoonotic in the manuscript and has been left as such with a reference added to support, likewise for the Sarcocystidae. References for the mycoplasmas, and a caveat have been added. Line 267- 272 now reads as: “These included one OTU of the genus *Bartonella*, a bacterial genus potentially including zoonotic species (e.g. *B. henselae*) (62), three OTUs of *Mycoplasma haemomuris* (combined for analysis), one OTU of *Mycoplasma coccoides* and one OTU of *Mycoplasma penetrans* (known to be zoonotic). These three *Mycoplasma* species have traditionally been associated with pathology in rodents (63-65). While more recent studies suggest that *Haemoplasma* may be largely subclinical in wildlife they have also highlighted the need for further studies increasing their geographic range and elucidating transmission (66).”

**62.** o 1c. It would be helpful to describe the criteria used to classify OTUs as putative pathogens. At the genus level, groups like Rickettsia and Francisella include both endosymbionts and pathogens, so additional clarification is warranted to avoid overinterpretation.

This has been clarified on lines 169 to 179: “From the clusters of microorganism DNA detected, putative pathogenic OTUs were filtered and selected (51). First, we discarded putative false positive results associated with sequence counts below two OTU-specific thresholds, which checked respectively for putative cross-contamination between samples during DNA extraction and PCR steps on the one hand, and incorrect assignment due to the generation of mixed clusters on the flowcell during Illumina sequencing on the other hand (52). Then, for each sample, and each OTU, we kept only the occurrences confirmed by the two PCR replicates of the same sample. Finally, the putative pathogenic OTUs were selected using their taxonomic affiliations and based on their potential zoonotic risk and pathogen impact on rodents, to separate them from commensal species (51).”

**63.** 2. Limitations of the 16S rRNA metabarcoding approach
The chosen method may underrepresent certain taxa and has limited resolution for accurately detecting specific pathogens.

The reason for choosing this method has been clarified in response to comments in Part 1 above.

**64.** 3. Use of statistical analyses, experimental design, and result presentation
**65.** o 3a. The results section could be streamlined, for example by referring readers directly to Table 3 where appropriate.

The results section has been streamlined by improving formatting and structure (see comment regarding methods/results). Furthermore, the AICc values, and references to table 3 have been added throughout the results section in order to provide more detail on the models and clarity to the reader.

**66.** o 3b. While the seasonal breakdown of figures (e.g., Figs. 5, 7) reflects the statistical analysis, its relevance to the main research questions could be reconsidered.

Figure 5 (now figure 6 in new document) was kept as it shows the reduction in prevalence with time, while also acknowledging that certain years have only single seasons data. This is part of the main hypotheses. Figure 7 has since been removed

**67.** o 3c. Given the specific hypotheses about invasion dynamics, the authors may consider targeted model comparisons. For example, comparing the AIC of models with and without the region × year × species interaction Similarly, it may be informative to assess whether species differences are more pronounced in Ireland than in France.

For each GLMM in this present study, the model was run through the package MuMIn, which takes all the variables and interactions fed into the GLMM and tests the models against each other with and without each factor to find the lowest AICc model (which best describe the data). The only interaction included an interaction with season and year due to the 2017 and 2022 data only containing a single season’s data.

**68.** o 3d. To assess a dilution effect, it may be clearer to directly correlate the relative abundance of C. glareolus with pathogen prevalence.

Grouping by region affords more statistical power than using relative abundance, as not all sites within each region contain equal numbers of animals sampled. This would be particularly evident in the invasion gradient analysis which included the 2016 only data, to avoid the influence of time, where due to the single year of sampling each site would exhibit fewer samples. Hence region was correlated with the assigned “percentage *Apodemus*” for each sample to show that region could be used as a proxy in this situation. To further establish whether invasion region was the contributing factor, site was included as a random effect variable. This was to account for differences in pathogen prevalence that may occur between sites in each region, due to climatic, environmental or other variables.

**69.** o 3e. Since the abundance of C. glareolus varied over time in the invasion front regions, summarizing infection rates across regions (e.g., in Fig. 4) may obscure temporal effects. Similarly, time alone may not serve as a reliable proxy for invasion in regions like the core, where host abundance remained stable (e.g., line 350).

The infection rates across regions and years have now been summarised in Supplementary table 2. Furthermore, this study focuses on the temporal effects, as the invasive host becomes more established on the island, to test whether assumptions made from cross-sectional studies hold true when tested over time. Further work would need to be carried out to investigate the changes in specific regions, such as the core where bank vole relative abundance has stayed relatively stable, or the 2021 invasion front which has become rapidly invaded between the 2016 and 2021 period.

**70.** o 3f. For the longitudinal data, it might be useful to analyze year-to-year differences as a dependent variable or apply repeated measures analyses.

This might work on more even datasets, however, as the data here is limited by the two years of full data (both seasons; spring and autumn) and two years of one season only (i.e. 2017 only has autumn, while 2022 only has spring). In this instance we decided to go for a more conservative approach, using GLMMs and fitting a year*season interaction, as our primary interest was in the change in infection patterns over time, while accounting for seasonal effects on pathogen prevalence.

**71.** o 3h. Some of the conclusions, such as the reduction in potentially pathogenic taxa in Irish C. glareolus populations, would benefit from supporting figures. For instance, a comparison of pathogen community composition between Irish and French C. glareolus and Apodemus populations would clarify whether community differences are stronger in the invasive host.

This has been included in Figure 3, on line 306 of the tracked changes document.

**72.** o 3i. It is unclear why Table 1 presents separate analyses for each host species rather than including host species as a model factor.

The invasive host, *C. glareolus,* in Ireland exhibits a different pathogen community to the native *A. sylvaticus,* which can be seen in Table 2. Including *C. glareolus* in the analyses for *A. sylvaticus and* vice versa would introduce a significant number of zeroes to the analysis. Likewise, the main focus of the analysis is to provide insights into how the presence of *C. glareolus* effects the pathogens in *A. sylvaticus,* and how its own pathogens are affected, hence host species specific models were chosen as the most appropriate models to investigate this.

**73.** o 3j. Splitting Table 2 by year and region might make patterns more interpretable.

This has been done and included in supplementary table 2, with a reference in the text as follows; “The prevalence for each region per year available in Supplementary Table 2” on line 276-277.

**74.** o 3k. Where results are non-significant, providing exact p-values would improve transparency.

P values have been added throughout the results section. In instances where only the P-value was given for GLMMs, the full Z and P values have been given, such as for the Sarcocystidae on line 471: “Age class was not statistically significant (Age class: Mature, GLMM, family = Binomial, z = -1;871, p = 0.0613; Age class: Juvenile, GLMM, family = Binomial, z = 0.453, p = 0.65).” and lines 604-606: “Age class did not significantly influence the prevalence of Sarcocystidae in this analysis (Age class: Mature, GLMM, family = Binomial, z = -0.848, p = 0.4; Age class; Juvenile, GLMM, family = Binomial, z = 1.727, p = 0.084).”

**Part III – Minor Issues: Editorial and Data Presentation Modifications**
Please use this section for editorial suggestions as well as relatively minor modifications of existing data that would enhance clarity.

**75.** Reviewer #1: The manuscript has great potential and it shows interesting results. More clarity and better presentation of the results are needed to convey the main message that gets a little bit lost. Minor mistakes, and formatting issues are reported in the comments included in the attached document.

Addressed in response to comments from reviewer 1 as described above, results have been restructured to mirror the new statistical analysis section. The summary table 3 is included towards the start of the results section as it provides a key reference point for the reader to come back to when reading the discussion.

**76.** Reviewer #2: I only have few minor comments:

Page 5, lines 133–135: It is unclear how the sample size was determined. It would be helpful to include the formula or method used for the calculation.

Sample size calculation were clarified and a link to the tool that was used is provided as follows: “The maximum sample size was calculated based on the estimated sample required to observe at least one infected individual based on a prevalence of 12%, and at least 1 success, with 95% confidence intervals (i.e. a sample size of 24 should allow detection of all pathogens with a prevalence of 12% and above, allowing detection of rare pathogens, calculated using Sampsize tool available at: <https://sampsize.sourceforge.net/iface/index.html#prev)>.” on lines 141-146.

**77.** Discussion:
Both rodent species exhibit wide geographic distributions and overlap spatially in several areas, including regions where they are both native. I suggest adding more background on the known interactions between these species. Is there evidence of competition, displacement, or other ecological interactions? Since the authors analyze the relative abundances of the native species in relation to the presence of the invasive species, and compare pathogen occurrence and prevalence between them, providing additional information on their interactions in other regions would strengthen the discussion.

This has been added to the introduction, referencing previous studies on the Irish rodent system, now reading: “Furthermore, previous studies have shown a reduced relative abundances of *A. sylvaticus* at sites invaded by *C. glareolus* (39, 43).” on lines 97-98.

**78.** Figures: Figures would be more visually engaging and easier to interpret if presented in color rather than grayscale.

While colour can be essential to understanding some figures, this paper only uses histograms to visualise our findings. In this case grayscale provides a clear contrast for readers with different forms of colour-blindness, making our figures universally accessible. Additionally, it allows for printing without coloured ink or toners. Asterisks have been included to indicate statistical significance where appropriate.

**79.** Reviewer #3: 1. Defining key terms such as “invasion gradient,” “invasion front,” and “invasion core” early in the text would help orient readers.

These are described in the introduction, highlighting that the core would have the highest relative abundance of *C. glareolus*; “This has allowed for the establishment of cross-sectional studies with an invasion gradient, containing sites from the invasion core (established from the introduction point, where *C. glareolus* exhibits it’s highest relative abundance), invasion front (sites where *C. glareolus* had most recently invaded), and uninvaded sites (*A. sylvaticus* only) (39).” on lines 98-101. Furthermore, it has been clarified in the methods section which sites are located in each of these regions.

**80.** 2. Line 118: It may be more appropriate to refer to the “amplification effect hypothesis” rather than the “spillback hypothesis.”

In this context we would argue that any increases in pathogen prevalences directly attributable the *C. glareolus* invasion would be considered spillback, as the amplification in the pathogen would be a result of the presence of the non-native host.

**81.** 3. Consider using a consistent metric for relative abundance throughout (i.e., proportion of C. glareolus vs. percentage of A. Sylvaticus; lines 189 and 219 and Fig. 2).

This reads as “percentage of *A.sylvaticus* relative to *C. glareolus*” in the methods section, now moved to lines 246 – 255. However, the proportion of *C. glareolus* relative to *A. sylvaticus* is used in the figure in order to aid interpretation of how invaded the sites are, as 100% *A. sylvaticus* for the invasion front 2021 would seem more confusing than the levels of *C. glareolus*.

The results section from lines 315 to 324 now mention percentage only and make each paragraph clear its “percentage of *A. sylvaticus* relative to *C. glareolus*” on lines 315-322.

**82.** 4. Line 237: B. henselae is rarely found in rodents (1–2%), and mainly in rats. Most Bartonella strains are not pathogenic to their rodent hosts.

This was intended to provide an example of a *Bartonella* species that this cluster may relate to, it is further described in the discussion section that further work is needed to identify the species of *Bartonell*a that are in this OTU cluster. Additionally, this present study only claimed that *Bartonella* was zoonotic, rather than pathogenic to rodents. Finally, I have added the below reference to this statement, which shows *A. sylvaticus* has the potential to carry *B. henselae*:

Engbæk, K. and Lawson, P.A. (2004), Identification of *Bartonella* species in rodents, shrews and cats in Denmark: detection of two *B. henselae* variants, one in cats and the other in the long-tailed field mouse[^†^](https://onlinelibrary.wiley.com/action/showCitFormats?doi=10.1111%2Fj.1600-0463.2004.apm1120603.x#fn1). APMIS, 112: 336-341. <https://doi.org/10.1111/j.1600-0463.2004.apm1120603.x>

**83.** 5. Table 2: The evidence for M. haemomuris transmission via lice is dated and limited. More recent findings suggest direct host-to-host transmission may be more important.

A caveat acknowledging this has been added to the table 2 legend: “*Mycoplasma* transmission has been linked to ectoparasites such as lice, based on early observations and PCR detection in arthropod vectors, although more experimental evidence for biological transmission is required (69).”

**84.** 6. Table 3: Please clarify what the values in parentheses represent—are they levels with the strongest effect (this does not appear to be the case)?

This was described in the footnote under the table. We have further clarified this by adding the term; “Statistically significant factors include the associated variable (level) in brackets.”

**85.** 7. Lines 260–261: Please clarify whether the authors refer to regional species richness or per-host richness, and consider supporting this with appropriate statistics or a figure.

This was clarified in the statistical analysis section on lines 187-188, now reading: “Pathogen species richness was calculated by counting the total number of putative pathogenic taxa recorded in an individual.”

**86.** 8. Fig. 3: Including Sarcocystidae here would make the figure more representative of the study’s full scope.

The patterns of infection that were consistent with the enemy release hypothesis were observed mainly in the bacterial species. As the Irish vole populations are quite heavily infected with Sarcocystidae, therefore comparing bacterial communities would be appropriate. Furthermore, Sarcocystidae were filtered differently between the French and Irish datasets making the prevalences incomparable as the French only kept 2 OTU’s, while our data includes four OTU’s. This would make comparing these prevalences inappropriate as they stand. Additionally excluding two OTU’s from the Irish data to make this comparable would also increase confusion for the reader.

**87.** 9. Line 311: Please specify whether the value 0.15 is a correlation coefficient.

This has been added in on line 530: “Prevalence was positively correlated (correlation coefficient; 0.15) with time (GLMM, family = Binomial, z = 2.344, p = 0.019; Pearsons’s correlation test, t = 2.4751, p < 0.05).”

**88.** 10. Line 341: If the intercept-only model is the best fit, this may suggest no explanatory variable significantly improves the model. Proceeding to additional models should be justified.

The AICc differs between these models by approximately 0.1, Therefore, it is acceptable as the delta AICc is less than 5, likewise age shows some statistical significance, which means there is some importance here regarding pathogen prevalence, even though the post-hoc analysis shows no difference between age groups. Hence, this has been kept, and the AICc values for each added to the results section, now reading: “For the invasion gradient analysis, the best model (lowest AICc value from MuMIn; 285.4) included only the null model, therefore the second lowest AICc model (285.486) was used, including only age class and sex (Table 3).”

**89.** 11. Lines 463–464: This pattern appears to be shared by A. Sylvaticus as well—clarification would be helpful.
This is explained from lines 632 to 648 prior when discussing rodent-borne pathogens in Ireland.
