# Supplementary material for: Longitudinal study of a rodent invasion reveals shifts in host pathogen interactions
Source: PLoS Pathog. 2026 Jul 23;22(7):e1014349. doi: 10.1371/journal.ppat.1014349 (PMC13395368; doi:10.1371/journal.ppat.1014349)
Supplement: Attachment — Submitted filename: Author response to reviewers R2.docx [file ppat.1014349.s007.docx]

Dear Editor and Reviewers,

Thank you for taking time to thoroughly review this manuscript again and providing valuable comments. We have incorporated the comments into the manuscript where appropriate and have offered our reasoning in cases where not.

We have incorporated all reviewers’ comments into a single list below, with our response, and changes in red text. Line numbers of your comments reflect the original manuscript, while line numbers in our response refer to their current location in the “tracked changes” document.

Yours Faithfully,

The authors

Comments on the manuscript:

1. Line 42: Remove disease and replace with epidemiological patterns.

Accepted: This has been included in the paper and now reads as: “When animals are introduced to new environments, they can disrupt local epidemiological patterns…” on line 42.

2. Line 55: don't they have to be in alphabetical order?

Accepted: This has been changed to alphabetical order, now displayed as “Biological invasions, enemy release, epidemiology, rodents, zoonotic pathogens, 16s rRNA metabarcoding.” on line 55.

3. Line 87: I don't think this is necessary. If you wanto to give this level of detsail then include species authority to clarify

Accepted: “(formerly *Myodes glareolus*)” has been removed on line 89.

4. Line 95: insertion of was and a comma
   Accepted, now reads as “Furthermore*, C. glareolus* in Ireland has a single identifiable introduction point in the south west, near Foynes, Co. Limerick, and the likely source population from Germany, which was identified through mitochondrial DNA (41).” On line 97.
5. Line 113/114: I think this is an overstatement. You certainly cover some gaps but not all the knowledge gaps on the topic. Be focused on what you have done. Also because how you selected the OTUs and the potential pathogens/parasites is very arbitrary at the end of the day

Accepted: Thank you for this comment, This statement now reads as: “In this study, we aim to use 16S rRNA amplicon sequencing to characterise the bacterial community with zoonotic potential or pathogenic potential to rodents associated with *C. glareolus* and *A. sylvaticus* in Ireland within a longitudinal study” on line 115.

6. Line 121: pathogenic for who
   Accepted: see comment 5 above.
7. Line 153: This is a result
   Accepted: Removed
8. Lines 167-175: I understand from your reply that you didn't want to put all those numbers in the methods and result section regarding this, but maybe a supplementary section that details the process as you did in your reply would be beneficial for transparency

Accepted: Now in supplementary material as suggested (Supp. table 3)

9. Line 194/195: Couldn’t just pool the data? or use a permutation technique? So how did you analyse the data?

   Accepted: We thank the reviewer for this suggestion and agree that pooling or permutation‑based community analyses would be appropriate approaches if sufficient infected samples were available across groups. We therefore explored these approaches in detail.

Community‑level analyses using Bray–Curtis dissimilarities were initially conducted in *R* using the packages vegan and cluster. However, these analyses were limited by the very high proportion of pathogen negative voles trapped in Ireland. Removal of pathogen negative individuals resulted in only five Irish vole samples remaining, with pathogens detected in only one or two individuals per taxon (one *Bartonella*, two *M. haemomuris*, one *M. penetrans*). This sample size was insufficient for meaningful multivariate or permutation-based community analyses.

Exploratory prevalence‑based comparisons were nevertheless examined. These suggested marked differences between host species and countries, with pathogen community similarity between France and Ireland being higher for *Apodemus sylvaticus* (Bray–Curtis similarity = 0.612) than for *C. glareolus* (Bray–Curtis similarity = 0.023). Hierarchical clustering based on Bray–Curtis dissimilarities further indicated that French *A. sylvaticus* and *C. glareolus* were more similar to each other than to Irish *C. glareolus*, which was consistently the most distinct population. However, because Bray–Curtis similarity and flexible beta clustering are designed for comparisons based on multiple independent samples per group, we concluded that applying these methods to prevalence‑level data did not provide a statistically sound basis for inference.

Accordingly, and following further consideration, we adopted pathogen‑specific prevalence analyses using direct frequency comparisons, which are more appropriate for sparse and zero‑inflated data. Fisher’s exact tests were used when expected cell counts were low, and Pearson’s chi‑square tests (without continuity correction) were used when expected counts were sufficient. These analyses are now clearly described in the Methods section, and the Results and Discussion have been updated accordingly

Methods (lines 208-213).

Differences between pathogen communities in hosts from France and Ireland were examined using Bray–Curtis dissimilarities calculated in *R* using the package vegan. Where community level analysis was not appropriate due to low numbers of infected hosts, differences in pathogen prevalence between countries were assessed using direct comparisons of infection frequencies. Fisher’s exact tests were used when expected cell counts were low, and Pearson’s chi‑square tests (without continuity correction) were used when expected counts were sufficient.

Results (lines 318-331).

Due to the high proportion of *C. glareolus* trapped in Ireland in which no pathogens were detected, it was not possible to conduct community level similarity analyses for this species. Removal of pathogen negative individuals resulted in only five Irish *C. glareolus* remaining (one positive for *Bartonella*, two for *M. haemomuris*, and one for *M. penetrans*), which was insufficient for appropriate multivariate community analysis. Therefore, pathogen‑specific prevalence comparisons were conducted for the three bacterial taxa detected in both France and Ireland: *Bartonella*, *M. haemomuris*, and *M. coccoides*.

*C. glareolus* from Ireland were found to have significantly lower prevalences of *Bartonella* (Ireland: 0.53%; France: 42.53%; Fisher’s exact test, *p* < 0.01) and *M. haemomuris* (Ireland: 1.06%; France: 40.27%; Fisher’s exact test, *p* < 0.01) than those from France. No significant association was detected between country and prevalence of *M. coccoides* in *C. glareolus* (Ireland: 0%; France: 0.45%; Fisher’s exact test, *p* > 0.05).

In contrast, *A. sylvaticus* from Ireland showed significantly higher prevalences of *M. coccoides* (Ireland: 37.82%; France: 10.52%; χ² = 23.13, *p* < 0.01) and *M. haemomuris* (Ireland: 45.60%; France: 17.89%; χ² = 21.08, *p* < 0.01) than those from France. No significant association was detected between country and prevalence of *Bartonella* in *A. sylvaticus* (Ireland: 56.48%; France: 45.26%; χ² = 3.21, *p* > 0.05).

Discussion (lines 585-587).

Consistent with these findings, both *Bartonella* and *M. haemomuris* occurred at significantly lower prevalences in *C. glareolus* populations in Ireland compared with those in France.

10. Lines 209 – 213: so the first analyse prevalence as a response variable and the "additional" model species richness?
    Accepted: Thank you for this comment, it has been updated to read: “…only prevalence data from 2016” on line 225. Likewise, on line 231, it now reads “…prevalence data from all four years…” to help clarify that the prevalence is the response variable, while the additional model done on the 2016 data included pathogen species richness.
11. Line 215: because they were not revisited?
    Accepted: Thank you for pointing this out, we have clarified this by adding this, now reading: “excluding Foynes 2, Carnpark and Loughkey, to keep the data comparable, as these sites were not revisited in 2021 and 2022.” on line 232.
12. Line 231: core, etc?
    Accepted: This was already clarified on line 236, explaining that the variable “region” is comprised of the invasion regions, reading: “Factors used in each model included season, region (invasion region; core, front 2016, front 2021) …”. It has been repeated in this section for clarity.

Now reads as: These analyses used the categorical variable of region (invasion region; core, front 2016, front 2021) as a proxy. on line 249

13. Line 233: delete underline
    Accepted: This has been removed.
14. Line 244: and species? : and delete text

Accepted: Thank you for this, this has been included in the table by adding “Rodent Species*” to the table, and the caveate “*The fixed variable Rodent species was only used in the model investigating differences in pathogen species richness between *Clethrionomys glareolus* and *Apodemus sylvaticus* in Ireland.” on Line 264.

15. Line 247: this section should follow the same order of methods. It is also a bit difficult to follow at times for the variety of definitions you use. I suggest a bit more consistency in the use of model terms etc.

Accepted: The methods section was restructured for previous reviewers’ comments in order to aid clarification, and follows the methods section as much as possible.

To more closely signpost the link between the order of the methods and results the following changes have been made:

In the methods section “Laboratory work & analysis”

Now reads as 2 sections “Sample collection and host characterisation” and “Pathogen detection in Irish rodent spleen samples” line 158 and 168.

To more closely align with “Pathogens detected in Irish rodent spleen samples” in the results section.

In the methods “Examining patterns of the enemy release hypothesis” now reads as “Examining patterns of infection related to the enemy release hypothesis” line 199

to more closely align with “Patterns of infection relating to the enemy release hypothesis” in the results section.

To further roadmap the alignment of the methods and results sections, this sentence has been added to the start of the results section “Results are presented following the analytical structure described in the Materials and Methods: (i) pathogen detection and prevalence, (ii) comparison with native‑range populations to examine enemy release, (iii) invasion‑gradient analyses (2016), and (iv) longitudinal analyses (2016–2022).” line 269

“Over all” model was changed to “full model” and “best model” to “best supported model” throughout for consistency and clarity

Other specific changes to results see points: Check later and add 16-18, 21-23, 25, 52 and 54.

16. Line 250: numbers of table should be in order of appearance in the text. why table 3 is mentioned before table 2? maybe you meant another table.
    Accepted: Thank you for spotting this, it has been changed to read “table 2” on line 292.
17. Line 256: ????

Not sure what the 4 question marks refer to but have removed italics pluralised and added explanation.

Accepted: Now reads as: “While more recent studies suggest that Haemoplasmas (Hemotropic mycoplasmas that include the species *M. coccoides* and *M. haemomuris*) may be largely subclinical….” on line 281.

18. Line 274: this table is still very confusing and I am sure there is a better way to describe these results. Also, in the methods you are not sufficiently clear to then understand this table
    Accepted: This table shows the statistically significant variables affecting pathogen species richness and pathogen prevalences detected in each host species. It is not intended to be interpreted on it’s own, however, to serve as a summary aide to the reader, while the following sections show the in-detail results.

    However, to increase the clarity of the table, the left column has been changed to read “Pathogen species richness”, grey shading has been added to break up the different host species and the title now reads as “**Table 3.** Summary of significant factors in pathogen species richness and pathogens prevalence detected when looking at both species combined, *Apodemus sylvaticus* independently and pathogen prevalence in *Clethrionomys glareolus* independently.” on line 300.
19. Line 297: why you presented the model results first and then this?
    Addressed in the above comment.
20. Line 314: where is described why you use 3 pathogens out of 5 for model in Apodemus, and only sarcocistidae for voles?

This was clarified in the statistical analyses section, where pathogens of 5% and above were selected, reading: “The threshold of 5% prevalence was selected as variations below this threshold would be too low for appropriate testing.”, and as displayed in table 2, only 3 pathogen species met this threshold for *A. sylvaticus* and only Sarcocystidae for *C. glareolus*. Line 218.

21. Line 333: this is methods
    Accepted: Now reads as “Below, we present the results of the invasion‑gradient analyses for *Bartonella*, *M. haemomuris* and *M. coccoides* in *Apodemus sylvaticus* sampled in Ireland.” Line 376.
22. Line 375: the asterisks are in the wrong position, should be in the middle to highlight that the two distribution are statistically different
    Accepted: Thanks for highlighting this. This figure has been formatted like this to keep consistent with Figure 5. Placing the asterisks between them or using brackets we found meant it could easily be confused that Front 2016 and Front 2021 were significantly different from each other. In order to clarify this is keeping with a similar figure published in plos pathogens (<https://journals.plos.org/plospathogens/article/figure?id=10.1371/journal.ppat.0030005.g003>) we clarified what the asterisks shows in the relevant part of figure title of figure 4 and 5. Which now read as:

Figure 4: "Asterisks indicate level of statistical significance (** = p <0.01), with the core being significantly different from Front 2016. line 422.

Figure 5: " Asterisks indicate level of statistical significance (** = p <0.01), with the core being significantly different from Front 2016 and front 2021, and with no statistical difference between Front 2016 and Front 2021." line 467

23. Line 380: delete? it is methods anyway
    Accepted: Now reads as “Below we present the patterns of infections observed in the invasion gradient analyses for the pathogen species *Bartonella, M.*  *haemomuris* and *M. coccoides* in *A. sylvaticus* sampled in Ireland”. Line 429
24. Line 413: same comment as per figure 4
    Accepted: Please see response to comment 22.
25. Line 432: there is no need of these introductory sentences as you should have stated this in the methods
    Accepted: We believe this signposting makes it easier for the reader, but agree they should read less like methods. Now reads as “Below we present the patterns of infection of *C. glareolus* with Sarcocystidae observed during longitudinal analyses.” Line 489.
26. 471: introduction of “that”
    Accepted: This has been inserted, now reading: “The aim of this study was to investigate the effect that an invasive…” on line 531.
27. Line 474: italic
    Accepted: Thank you for pointing this out, these have been changed to italic on line 534. Now reading, “such as *Bartonella* or *Borrelia …*”
28. Line 485: “and”
    Accepted: Thank you now read as “…including *Toxoplasma, Eimeria* and *Neospora* …” Line 545.
29. Line 500: spleen should retreive higher prevalence than ear biopsies. it is more likely the specificity of the PCR
    While we agree, the specificity of the PCR could account for differences observed, the ear has been shown in some studies to be the most sensitive organ for detecting *Borrelia*, so we think it is also worth mentioning (see Zawada SG, von Fricken ME, Weppelmann TA, Sikaroodi M, Gillevet PM (2020) Optimization of tissue sampling for *Borrelia burgdorferi* in white-footed mice *(Peromyscus leucopus)*. PLOS ONE 15(1): e0226798. [https://doi.org/10.1371/journal.pone.0226798))(](https://doi.org/10.1371/journal.pone.0226798)). However, it is also noted in that the rodent associated *B.* *afzelii* is less common in Ireland.
30. Line 519: do you mean age assignation?
    Accepted: This has been changed to read “Further studies should be conducted to confirm this and incorporate consistent techniques in assigning age classifications in both countries…” on line 580.
31. Line 546: even?
    Accepted: Now reads as “This result was even more surprising given that male *C. glareolus…*”. Line 612.
32. Line 558: of
    Accepted: This has been changed, now reading “This would be further supported by the findings of the longitudinal analysis…” on line 624.
33. Line 562: investigations
    Accepted: This has been updated by adding a comma to mechanisms, and pluralising investigation while adding in the “are” for comment 34 below, to now read “…these mechanisms, further investigations of the eco-immunology of invasive *C. glareolus* populations, and identification of the species present within these Sarcocystidae are required.” on line 628.
34. Line 563: are
    Accepted: Rectified in comment 33 above.
35. Line 566: delete, it is in the title of the section
    Accepted. Thank you, the sentence “The effect of the *C. glareolus* invasion on the pathogen communities in the native *A. sylvaticus* was also investigated.” was deleted to avoid repetition on line 632.
36. Line 576: I don't think this is correct. what do you mean? there should be another explanation, or delete as it does not add anything to the discussion
    Accepted and deleted. Line 642.
37. Lines 582-587: this sentence (lines 582–587) is far too long and difficult to follow. please rephrase so the point you are making is clear
    Accepted: Thank you for pointing this out, the sentence has been broken up and rephrased to add clarity on line 649, now reading as:
    “The dilution of *M. haemomuris,* in *A. sylvticus* in Ireland (associated with the presence of *C. glareolus*), and the dilution of *M. haemomuris* (strain 2) in France (presumed to be due to an increase in small mammal diversity), demonstrate that multiple factors (such as climate change, invasive species, deforestation and urbanisation) can alter rodent populations and consequently pathogen prevalences. Understanding the underlying mechanisms driving these patterns can therefore improve predictions of disease transmission and inform disease management strategies.”
38. Line 603: in this study, you have mentioned this for other pathogens as well, so it should be pointed out better as a limitation/caveat of your study

Accepted: This sentence has been added to the methods so the reader is aware of this caveat earlier “It should be noted that 16S rRNA amplicon sequencing primarily only allows detection at the genus level for some bacterial taxa and may not reliably resolve species or strain level diversity without targeted, genus‑specific genetic markers” line 183.

Also see additions in comment 46.

This limitation has also been discussed throughout the discussion, including the conclusion section where the caveat is referenced for Sarcocystidae also, which has been updated to remove the comma (as per comment 42) on line 723, and now reads”

“Further work is also needed to identify the Sarcocystidae and *Bartonella* species detected in this study in order to fully understand the pathogen community dynamics, as genetic differences may reveal different levels of host specificity and help explain host-pathogen interactions.”

39. Line 611: it seems sometimes you have selected these two pathogens, do you state somewhere that these are specifically associated with the two host species? or is there a reason why you mention selectively only some of the pathogen taxa found in each analyses. are you reporting only the statistically significan results? if so, this needs to be stated
    We thank the reviewer for raising this point. Pathogens included in the statistical analyses were selected based on a minimum prevalence threshold of 5%, as stated in the Methods (line 216), to ensure sufficient power for reliable statistical testing. Consequently, only taxa meeting this criterion are discussed in detail in relation to invasion‑related patterns. The Discussion therefore focuses on those pathogens for which statistically supported patterns could be evaluated in the context of the study hypotheses, namely the effects of biological invasion on pathogen prevalence.
40. Line 620-268: I don't fully understand. I think this should be rephrased and clarified. why "if the assumption... is correct, that would further demonstrate..."???
    Accepted: Now reads as “If the assumption that *B. microti* is confined to eastern regions of Ireland is correct, and if sampling of *C. glareolus* had occurred prior to the invasion reaching this area, it is possible that this pathogen would not have been detected and that *B. microti* would have been assumed to be absent from Ireland. This further demonstrates the importance of long‑term studies of pathogens in invasive species, particularly for detecting the emergence of pathogens in newly colonised areas.” Line 701.
41. Line 635, insertion of a comma.
    Accepted: This has been inserted, now reading “compared to the native *A. sylvaticus*, and fewer bacterial taxa compared …” on line 715.
42. Line 643, removal of a comma.

Accepted: Addressed in comment 38.

43. Line 649: “d”
    Accepted: This has been inserted to now read as “we demonstrated that long-term data are essential …” on line 729.
44. **Additional Editor Comments:**

    Dear Authors,
    The reviewers have completed their evaluation of your manuscript. I encourage you to revise and resubmit your work, ensuring that all reviewer comments are thoroughly addressed. Please incorporate the feedback carefully and provide a detailed, point-by-point response that clearly outlines every change made in response to the reviewers’ suggestions.
    In addition, kindly correct all typographical and grammatical errors, and ensure that the manuscript is prepared in full compliance with the journal’s formatting and submission guidelines.
    We look forward to receiving your revised submission.

We thank the editor for facilitating such thorough evaluation and commentary by the reviewers again. We have followed the instructions as outlined.

**Journal Requirements:**

If the reviewer comments include a recommendation to cite specific previously published works, please review and evaluate these publications to determine whether they are relevant and should be cited. There is no requirement to cite these works unless the editor has indicated otherwise.

1) Please ensure that the CRediT author contributions listed for every co-author are completed accurately and in full.
At this stage, the following Authors/Authors require contributions: Andrew McManus, Maxime Galan, Nathalie Charbonnel, Celia Holland, Vincent Sluydts, Annetta Zintl, and Peter Stuart. Please ensure that the full contributions of each author are acknowledged in the "Add/Edit/Remove Authors" section of our submission form.
The list of CRediT author contributions may be found here: <https://journals.plos.org/plospathogens/s/authorship#loc-author-contributions>

Completed

45. **Reviewers' Comments:**

    Reviewer's Responses to Questions

**Part I - Summary**
Please use this section to discuss strengths/weaknesses of study, novelty/significance, general execution and scholarship.

Reviewer #1: Thank you for implementing all the required changes. The manuscript has improved significantly in clarity, and although a few minor revisions and clarifications are still needed, it is very close to being ready for publication.

Thank you for this comment, we have addressed the above comments on the manuscript left by all three reviewers, and the specific comments (1-43) left in the review below.

46. Reviewer #3: I have carefully reviewed the revised manuscript. The structure is now clearer, and the methods, results, and rationale behind the statistical analyses are easier to follow. The revised presentation also links more directly to the three main study hypotheses, which improves the readability and flow. However, there remain two key issues that, in my view, limit the robustness of the conclusions.

    First, the absence of complementary pathogen-specific assays makes it difficult to fully support some of the central patterns—particularly those that contrast with previously reported trends (e.g., the lack of a dilution effect in Bartonella or the pattern opposite to enemy-release expectations in Sarcocystidae). These findings may reflect limitations of the 16S rRNA metabarcoding approach, such as its lower sensitivity or potential biases toward certain strains.

Accepted: We thank the reviewer for this constructive comment. We agree that the absence of complementary, pathogen‑specific assays represents an important limitation, particularly for taxa such as *Bartonella* and Sarcocystidae. We have therefore clarified in the Materials and Methods that 16S rRNA amplicon sequencing may not reliably resolve fine‑scale diversity for these groups. See comment 38. We have revised the Discussion to explicitly acknowledge that patterns contrasting with previously reported trends, such as the lack of a dilution effect for *Bartonella* or the higher prevalence of Sarcocystidae at the invasion front, should be interpreted with appropriate caution. Importantly, we emphasise that these patterns are presented as biologically meaningful signals warranting further investigation using complementary, pathogen‑specific approaches, rather than as definitive evidence contradicting earlier studies.

Line 672 now reads as follows:

Hence, the observed difference may stem from the detection techniques employed, as Telfer *et al.* (72) utilized bacterial cultivation and genus-specific PCR assays, while this present study only found one OTU of *Bartonella,* using 16SrRNA amplification and illumina sequencing an approach that may have limited sensitivity for resolving Bartonella species or strains compared with genus‑specific assays, for example using gltA and rpoB gene sequencing. Nevertheless, the consistently low detection of Bartonella in *C glareolus* across sites and years suggests that host competence differences may still contribute to the observed patterns.

Line 609 now reads as follows:

However, interpretation of these patterns should be treated with caution, as 16S rRNA metabarcoding does not allow reliable species level resolution within the Sarcocystidae, and different species may vary in transmission dynamics

However, we also agree with point 47 (below) that invasion outcomes can depend on specific host pathogen associations as well as the timing of invasion. This was why we utilised the 16S rRNA metabarcoding approach. Pathogenic bacteria and protozoa of wild rodents in Ireland remain relatively understudied, and 16S rRNA sequencing allowed us to undertake a broad survey to identify taxa present, at least at the genus level. Importantly, this approach enabled simultaneous assessment of multiple potential pathogens and comparison of patterns across different taxa, which would not have been feasible had we focused our resources exclusively on a single pathogen.

47. Second, while the manuscript concludes that C. glareolus invasion shows signatures consistent with enemy release, a potential dilution effect, and temporal changes in pathogen patterns along the invasion gradient, these patterns are evident in only a small subset of pathogens. In contrast, several other pathogens do not follow these trends. In this context, I think the main take-home message that could be emphasized more strongly is that invasion outcomes appear to depend on the specific host–pathogen association and the timing of the invasion, and therefore cannot be generalized or predicted in advance. Highlighting this point would, in my opinion, provide a balanced and more robust interpretation of the findings.

Accepted: We thank you for this comment and agree it is a key point we wish to get across. We agree with the reviewer that it should be the main take home message. Therefore, we have added it as the final sentence to the conclusion. Therefore, this now reads as “Disease dynamics during biological invasions cannot be generalised across all host–pathogen associations, nor reliably predicted from a single stage of invasion. Instead, long‑term investigations employing broad, multi‑pathogen approaches are required to capture the dynamic and pathogen‑specific nature of disease processes as invaders establish and spread.” Line 731.

48. **Part II – Major Issues: Key Experiments Required for Acceptance**
    Please use this section to detail the key new experiments or modifications of existing experiments that should be absolutely required to validate study conclusions.

    Generally, there should be no more than 3 such required experiments or major modifications for a "Major Revision" recommendation. If more than 3 experiments are necessary to validate the study conclusions, then you are encouraged to recommend "Reject".

Reviewer #1: n/a

Reviewer #3: In my view, three major issues currently limit the robustness of the conclusions:
1. Lack of complementary pathogen-specific assays (as detailed above).

Accepted: See above reply to comment 46.

2. Segregation of bioinformatic analyses into four separate batches (France, 2016, 2017, and 2022). I understand that the sequencing itself was performed in different runs, yet I wonder whether it would be possible to integrate all datasets and cluster them jointly into OTUs. Such an approach could strengthen the comparative framework of the study, particularly for contrasts between regions (e.g., Ireland vs. France) and across time.

Accepted: Currently the analysis is divided up to help with the understanding of complex issues. The French data is separated due to differences in filtering for Sarcocystidae.

The Irish data was homogenised so it could be run longitudinally (2016 – 2022). However, a single year of data (2016 – where the “invasion gradient” was most prominent, with uninvaded *A. sylvaticus* only sites) was chosen to analyse separately, as a cross-sectional study. This was done in order to focus on the effect of the invasion gradient on pathogen communities, while contrasting these findings to the longitudinal analyses to understand the effect of time on this invasion.

Data was pooled for analysis but problems were encountered due to the high number of negative vole samples from Ireland, see comment 9 for new sections in methods and results.

3. Emphasis of the take-home messages on the three study hypotheses, rather than highlighting what the data appear to support more strongly—that invasion outcomes depend on the specific host–pathogen associations and the timing of the invasion (as outlined above).

Accepted: see comment 47

49. **Part III – Minor Issues: Editorial and Data Presentation Modifications**
    Please use this section for editorial suggestions as well as relatively minor modifications of existing data that would enhance clarity.

Reviewer #1: Minor edits, formatting issues, and comments are listed in the attached PDF, along with a few questions that still require clarification to ensure full clarity for the reviewer and the readers. In addition, a further round of language editing would be beneficial to ensure that the manuscript flows smoothly.

Thank you for these comments, these have been addressed in the above comments.

50. Reviewer #3: • Line 250: Should this refer to Table 2 rather than the current table number?
    Accepted: This has been addressed in comment 16 above.
51. • Table 2 (M. penetrans): How was the confidence interval calculated when only a single host was infected with this parasite?

The RStudio package propCIs calculated this to 95% confidence by using the number of positives and the total sample size to generate a maximum and minimum figure (i.e. the 95% confidence interval around this 1 positive relative to the total host species population sampled).

52. • Line 285: You may consider replacing “moreover” with “in contrast,” which might better reflect the relationship between the statements.

Accepted: This has been changed to read as: “In contrast, *A. sylvaticus* individuals trapped in Ireland …” on line 313.

53. • Lines 516–518: Using the absence of potentially pathogenic taxa in C. glareolus populations in Ireland as evidence for the enemy release hypothesis may be misleading, given that the same pattern was observed in A. sylvaticus populations.

We do agree that *C. glareolus,* may have less pathogens in Ireland due to Island biogeography, similar to *A. sylvaticus.* However, we believe the further analysis suggested by reviewers (see comment 9 and 48) shows the distinct pathogen community of *C. glareolus* is quite unique reflecting its relatively recent arrival to Ireland. As noted in the paper, this is consistent with previous studies of *C. glareolus* pathogens in Ireland such as those by Loxton et al. 2016 and Stuart et al. 2020.

54. • References 63 and 64 do not provide evidence that Mycoplasma species have traditionally been considered pathogenic in rodents.

To avoid overstatement that sentence was written as “traditionally been associated” rather than “considered pathogenic”. It read: These three *Mycoplasma* species have traditionally been associated with pathology in rodents (63-65).

Reference 63 does state that in their experiment infection of mice with *E. coccoides* did result in slight normocytic anaemia.

Also, “Niven et al. (1952) and Gledhill (1956) showed that the pathogenic effects of mouse hepatitis virus were greatly increased by intercurrent infections with *E. coccoides*. Furthermore, Seamer, Gledhill, Barlow and Hotchin (1961) obtained a similar result with lymphocytic choriomeningitis virus”.

Reference 64 states “Different *Mycoplasma* species appear to have different effects on B cells. For instance, *M. arginini* acts only on LPS preactivated B-cell blasts, resulting in an increase in cell numbers but inducing no maturation to Ig secretion by itself (19). *M. orale* induced a significant proliferation of murine B cells but no concomitant Ig secretion (15). *M. hyorhinis, M. neurolyticum, M. pneumoniae*, and *M. pulmonis* are capable of inducing proliferation and maturation to Ig secretion of murine B cells (19). It is interesting that the last four mycoplasma species are believed to be pathogenic organisms associated with various diseases of animals and humans.”

Reference 64 concludes ; “In conclusion, our study and the studies of others have revealed that mycoplasmas possess a component(s) in membranes that acts as a potent B-cell mitogen. The B-cell mitogens in different *Mycoplasma* species, however, have different effects on murine B cells, which may be caused by components with different biochemical structures. Whether this interaction between mycoplasmas and B cells can be implicated in disease pathogenesis in vivo, leading to autoimmune reactions, modulation of immunity, and/or promotion of abnormal B-cell proliferation, needs further investigation.”

We believe these studies show that mycoplasmas may modulate host immune responses, even in the absence of overt disease, and may thereby influence susceptibility to co‑infecting pathogens.

For further clarity on this, the statement now reads as follows:

These three *Mycoplasma* species have previously been associated with immunological and pathological effects in rodents, particularly through modulation of host immune responses to co‑infecting pathogens (63-65). Line 277

55. double check reference list font and font size, as well as line spaces, as there seem to be some inconsistencies

Accepted, removed spacing issues and now all same font and size

PLOS authors have the option to publish the peer review history of their article ([what does this mean?](https://track.editorialmanager.com/CL0/https:%2F%2Fjournals.plos.org%2Fplospathogens%2Fs%2Feditorial-and-peer-review-process%23loc-peer-review-history/1/010f019be7372956-f72a43a6-3bd7-4346-bf75-980bfe6320c5-000000/7ymODmhCMxqnRdR4hwiBiVEqpWa2hZLWDhF_Vz7AkQk=247)). If published, this will include your full peer review and any attached files.


If you choose “no”, your identity will remain anonymous but your review may still be made public.


**Do you want your identity to be public for this peer review?** For information about this choice, including consent withdrawal, please see our [Privacy Policy](https://track.editorialmanager.com/CL0/https:%2F%2Fwww.plos.org%2Fprivacy-policy/1/010f019be7372956-f72a43a6-3bd7-4346-bf75-980bfe6320c5-000000/G4-IL8KE1fQLSyeDJbwDeIiUNoLK9V2dUUTgVVpQ4IA=247).

Reviewer #1: No

Reviewer #3: No
