# Supplementary material for: Longitudinal study of a rodent invasion reveals shifts in host pathogen interactions
Source: PLoS Pathog. 2026 Jul 23;22(7):e1014349. doi: 10.1371/journal.ppat.1014349 (PMC13395368; doi:10.1371/journal.ppat.1014349)
Supplement: Peer review history

# Peer review: Decision Letter 0

**DOI:** 10.1371/journal.ppat.1014349.r001

**Authors:** Debra Bessen, Bersissa Kumsa

7 Jul 2025

-->PPATHOGENS-D-25-01038

Longitudinal study of a rodent invasion reveals shifts in host pathogen interactions.

PLOS Pathogens

Dear Dr. Stuart,

Thank you for submitting your manuscript to PLOS Pathogens. After careful consideration, we feel that it has merit but does not fully meet PLOS Pathogens's publication criteria as it currently stands. Therefore, we invite you to submit a revised version of the manuscript that addresses the points raised during the review process.

Please submit your revised manuscript within 60 days Sep 05 2025 11:59PM. If you will need more time than this to complete your revisions, please reply to this message or contact the journal office at plospathogens@plos.org. When you're ready to submit your revision, log on to https://www.editorialmanager.com/ppathogens/ and select the 'Submissions Needing Revision' folder to locate your manuscript file.

Please include the following items when submitting your revised manuscript:

* A rebuttal letter that responds to each point raised by the editor and reviewer(s). You should upload this letter as a separate file labeled 'Response to Reviewers'. This file does not need to include responses to any formatting updates and technical items listed in the 'Journal Requirements' section below.

* A marked-up copy of your manuscript that highlights changes made to the original version. You should upload this as a separate file labeled 'Revised Manuscript with Track Changes'.

* An unmarked version of your revised paper without tracked changes. You should upload this as a separate file labeled 'Manuscript'.

If you would like to make changes to your financial disclosure, competing interests statement, or data availability statement, please make these updates within the submission form at the time of resubmission. Guidelines for resubmitting your figure files are available below the reviewer comments at the end of this letter.

We look forward to receiving your revised manuscript.

Kind regards,

Bersissa Kumsa, DVM, MSc, PhD

Academic Editor

PLOS Pathogens

Debra Bessen

Section Editor

PLOS Pathogens

Sumita Bhaduri-McIntosh

Editor-in-Chief

PLOS Pathogens

orcid.org/0000-0003-2946-9497

Michael Malim

Editor-in-Chief

PLOS Pathogens

orcid.org/0000-0002-7699-2064

**Additional Editor Comments:**

Dear Authors,

The reviewers have completed their evaluation of your manuscript. I encourage you to revise and resubmit your work, ensuring that all reviewer comments are thoroughly addressed. Please incorporate the feedback carefully and provide a detailed, point-by-point response that clearly outlines every change made in response to the reviewers’ suggestions.

In addition, kindly correct all typographical and grammatical errors, and ensure that the manuscript is prepared in full compliance with the journal’s formatting and submission guidelines.

We look forward to receiving your revised submission.

**Journal Requirements:**

1) Please ensure that the CRediT author contributions listed for every co-author are completed accurately and in full.

At this stage, the following Authors/Authors require contributions: Andrew McManus, Maxime Galan, Nathalie Charbonnel, Celia Holland, Vincent Sluydts, Annetta Zintl, and Peter Stuart. Please ensure that the full contributions of each author are acknowledged in the "Add/Edit/Remove Authors" section of our submission form.

The list of CRediT author contributions may be found here: https://journals.plos.org/plospathogens/s/authorship#loc-author-contributions

2) Please upload all main figures as separate Figure files in .tif or .eps format. For more information about how to convert and format your figure files please see our guidelines:

https://journals.plos.org/plospathogens/s/figures

3) We have noticed that you have uploaded Supporting Information files, but you have not included a list of legends. Please add a full list of legends for your Supporting Information files after the references list.

4) Some material included in your submission may be copyrighted. According to PLOSu2019s copyright policy, authors who use figures or other material (e.g., graphics, clipart, maps) from another author or copyright holder must demonstrate or obtain permission to publish this material under the Creative Commons Attribution 4.0 International (CC BY 4.0) License used by PLOS journals. Please closely review the details of PLOSu2019s copyright requirements here: PLOS Licenses and Copyright. If you need to request permissions from a copyright holder, you may use PLOS's Copyright Content Permission form.

Please respond directly to this email and provide any known details concerning your material's license terms and permissions required for reuse, even if you have not yet obtained copyright permissions or are unsure of your material's copyright compatibility. Once you have responded and addressed all other outstanding technical requirements, you may resubmit your manuscript within Editorial Manager.

Potential Copyright Issues:

i) Figure 1. Please (a) provide a direct link to the base layer of the map (i.e., the country or region border shape) and ensure this is also included in the figure legend; and (b) provide a link to the terms of use / license information for the base layer image or shapefile. We cannot publish proprietary or copyrighted maps (e.g. Google Maps, Mapquest) and the terms of use for your map base layer must be compatible with our CC BY 4.0 license.

Note: if you created the map in a software program like R or ArcGIS, please locate and indicate the source of the basemap shapefile onto which data has been plotted.

If your map was obtained from a copyrighted source please amend the figure so that the base map used is from an openly available source. Alternatively, please provide explicit written permission from the copyright holder granting you the right to publish the material under our CC BY 4.0 license.

If you are unsure whether you can use a map or not, please do reach out and we will be able to help you. The following websites are good examples of where you can source open access or public domain maps:

* U.S. Geological Survey (USGS) - All maps are in the public domain. (http://www.usgs.gov)

* PlaniGlobe - All maps are published under a Creative Commons license so please cite u201cPlaniGlobe, http://www.planiglobe.com, CC BY 2.0u201d in the image credit after the caption. (http://www.planiglobe.com/?lang=enl)

* Natural Earth - All maps are public domain. (http://www.naturalearthdata.com/about/terms-of-use/).

5) In the online submission form, you indicated that your data will be submitted to a repository upon acceptance. We strongly recommend all authors deposit their data before acceptance, as the process can be lengthy and hold up publication timelines. Please note that, though access restrictions are acceptable now, your entire minimal dataset will need to be made freely accessible if your manuscript is accepted for publication. This policy applies to all data except where public deposition would breach compliance with the protocol approved by your research ethics board. If you are unable to adhere to our open data policy, please kindly revise your statement to explain your reasoning and we will seek the editor's input on an exemption.

6) Please amend your detailed Financial Disclosure statement. This is published with the article. It must therefore be completed in full sentences and contain the exact wording you wish to be published.

1) State the initials, alongside each funding source, of each author to receive each grant. For example: "This work was supported by the National Institutes of Health (####### to AM; ###### to CJ) and the National Science Foundation (###### to AM)."

2) State what role the funders took in the study. If the funders had no role in your study, please state: "The funders had no role in study design, data collection and analysis, decision to publish, or preparation of the manuscript."

3) If any authors received a salary from any of your funders, please state which authors and which funders..

If you did not receive any funding for this study, please simply state: u201cThe authors received no specific funding for this work.u201d

7) Please ensure that the funders and grant numbers match between the Financial Disclosure field and the Funding Information tab in your submission form. Note that the funders must be provided in the same order in both places as well. Currently, the Financial Disclosure states there was no funding received.

**Reviewers' Comments:**

Reviewer's Responses to Questions

**Part I - Summary**

Please use this section to discuss strengths/weaknesses of study, novelty/significance, general execution and scholarship.

Reviewer #1: Dear authors,

I was pleased to review this manuscript on the hidden dynamics of pathogen transmission in a biological invasion context. I think that the longitudinal study is extremely interesting and certainly deserves publication after a few issues are resolved.

My concerns are mainly about the statistical analyses, I have left detailed comments in the pdf attached, but I think that the methods are slightly confusing and lack of an input form an expert in statistics, which could improve the explanation and the rationale behind the chosen methodology and the reporting of the results. That said, I do not think the methodology is wrong, just needs to be reported in a better way, and there are some things to be clarified (see the comments in the document attached).

Then, I think that the structure of methods, results, and consequently discussion should be improve to streamline the main message, and the sub-sections need to be restructured/reordered so that the reader can follow the different aspects through the various section of the manuscript.

Hence, I also left comments for the images that sometimes can be improved to be clearer and more immediate.

Minor issues regard the precision of some statement, the formatting of the reference list and other minor comments that can be found in the pdf attached.

All the details are reported in the comments in the document attached.

I hope that my comments will improve the quality of the manuscript and will give it the relevance it deserves.

Reviewer #2: This interesting manuscript investigates pathogen dynamics in an invasive rodent through a longitudinal study conducted in both core invasion zones and the invasion front. It also examines the relationships between the invasive species, its pathogens, and a native rodent species.

The article provides valuable insights into invasive species and their associated parasites/pathogens within the framework of the enemy release hypothesis. The analysis is robust, supported by a solid sample size and state-of-the-art pathogen detection methods. The study is also well contextualized through comparisons with data from France. The authors appropriately acknowledge the study’s limitations and outline relevant avenues for future research.

Reviewer #3: Invasive species can influence epidemiological processes through a range of direct and indirect mechanisms, and gaining a better understanding of these dynamics is of clear value both theoretically and practically. McManus et al. have taken advantage of a unique "natural" experiment involving the recent spread of the bank vole (Clethrionomys glareolus) in Ireland to explore the epidemiological consequences of host invasion. Their study compares rodent-borne microbial taxa across a geographic gradient, including sites in France (the native range), as well as core and front areas of the invasion in Ireland. By incorporating both cross-sectional and longitudinal data, and by comparing patterns in a second rodent species (Apodemus sylvaticus) that is native to both regions, the authors aim to disentangle potential effects of invasion history on microbial communities. Their findings suggest patterns that may be consistent with enemy release and dilution effects, and they also highlight temporal dynamics in pathogen prevalence along the invasion gradient.

I commend the authors for their creative and ambitious study design, the integration of multiple data types, and the rigorous statistical approach. However, I have concerns about whether the manuscript meets the criteria of originality, impact, and scientific depth expected for publication in PLOS Pathogens, which emphasizes advances in pathogen biology. In my view, the primary limitations stem from the reliance on 16S rRNA metabarcoding in a relatively small and well-characterized host–microbe system. Although this method is valuable in more complex microbial communities and could serve as a preliminary tool to identify candidate species in this context, its application here leads to limited taxonomic resolution. As a result, it constrains the ability to draw confident conclusions regarding microbial identity, pathogenic potential, and ecological significance, particularly given the lower sensitivity of this approach. Additionally, the statistical analyses, though comprehensive, feel exploratory rather than hypothesis-driven, which makes it difficult to pinpoint key results and interpret them with confidence. As such, I am not fully convinced that the study yields novel or robust insights beyond those already offered by prior work focused on specific pathogens within this system. For these reasons, I recommend that the authors consider submitting their manuscript to a journal with a broader ecological or invasion biology focus. I offer detailed comments below in the hope that they will help strengthen the manuscript in its next iteration.

**********

**Part II – Major Issues: Key Experiments Required for Acceptance**

Please use this section to detail the key new experiments or modifications of existing experiments that should be absolutely required to validate study conclusions.

Generally, there should be no more than 3 such required experiments or major modifications for a "Major Revision" recommendation. If more than 3 experiments are necessary to validate the study conclusions, then you are encouraged to recommend "Reject".

Reviewer #1: The methods section might benefit a reorganisation of the sub-sections, splitting it into defined data/sample collection, molecular data analysis, statistical analysis. Also, it is less well written than the introduction and it is difficult to follow at times, e.g. there are redundant sentences.

The statistical analyses need to be reviewed, and so the relative results section. See the general comments and the comments in the document attached. The reporting of statistical results is confusing and difficult to follow, I am not sure the methodology fully matches the reported results. The methods and results section regarding statistical testing, although might not be entirely wrong might benefit from a review from an expert in statistics. I think some tests are redundant and the result section might be streamlined with better tables and figures.

Reviewer #2: --

Reviewer #3: 1. Taxonomic identification and interpretation of the biology of microbes

o 1a. The focus on genus and family levels limits the capacity to draw meaningful conclusions about pathogenicity and the biology of the microbes. For instance, while all members of Sarcocystidae are parasitic, their clinical relevance varies. Some may cause subclinical or negligible effects, particularly in well-adapted hosts.

o 1b. Some statements regarding pathogenicity may require further support. For example, existing literature suggests limited pathogenic effects of Bartonella and Mycoplasma spp. in rodent hosts. If the authors are aware of studies suggesting otherwise, citing them would provide a more balanced view.

o 1c. It would be helpful to describe the criteria used to classify OTUs as putative pathogens. At the genus level, groups like Rickettsia and Francisella include both endosymbionts and pathogens, so additional clarification is warranted to avoid overinterpretation.

2. Limitations of the 16S rRNA metabarcoding approach

The chosen method may underrepresent certain taxa and has limited resolution for accurately detecting specific pathogens.

3. Use of statistical analyses, experimental design, and result presentation

o 3a. The results section could be streamlined, for example by referring readers directly to Table 3 where appropriate.

o 3b. While the seasonal breakdown of figures (e.g., Figs. 5, 7) reflects the statistical analysis, its relevance to the main research questions could be reconsidered.

o 3c. Given the specific hypotheses about invasion dynamics, the authors may consider targeted model comparisons. For example, comparing the AIC of models with and without the region × year × species interaction Similarly, it may be informative to assess whether species differences are more pronounced in Ireland than in France.

o 3d. To assess a dilution effect, it may be clearer to directly correlate the relative abundance of C. glareolus with pathogen prevalence.

o 3e. Since the abundance of C. glareolus varied over time in the invasion front regions, summarizing infection rates across regions (e.g., in Fig. 4) may obscure temporal effects. Similarly, time alone may not serve as a reliable proxy for invasion in regions like the core, where host abundance remained stable (e.g., line 350).

o 3f. For the longitudinal data, it might be useful to analyze year-to-year differences as a dependent variable or apply repeated measures analyses.

o 3h. Some of the conclusions, such as the reduction in potentially pathogenic taxa in Irish C. glareolus populations, would benefit from supporting figures. For instance, a comparison of pathogen community composition between Irish and French C. glareolus and Apodemus populations would clarify whether community differences are stronger in the invasive host.

o 3i. It is unclear why Table 1 presents separate analyses for each host species rather than including host species as a model factor.

o 3j. Splitting Table 2 by year and region might make patterns more interpretable.

o 3k. Where results are non-significant, providing exact p-values would improve transparency.

**********

**Part III – Minor Issues: Editorial and Data Presentation Modifications**

Please use this section for editorial suggestions as well as relatively minor modifications of existing data that would enhance clarity.

Reviewer #1: The manuscript has great potential and it shows interesting results. More clarity and better presentation of the results are needed to convey the main message that gets a little bit lost. Minor mistakes, and formatting issues are reported in the comments included in the attached document.

Reviewer #2: I only have few minor comments:

Page 5, lines 133–135: It is unclear how the sample size was determined. It would be helpful to include the formula or method used for the calculation.

Discussion:

Both rodent species exhibit wide geographic distributions and overlap spatially in several areas, including regions where they are both native. I suggest adding more background on the known interactions between these species. Is there evidence of competition, displacement, or other ecological interactions? Since the authors analyze the relative abundances of the native species in relation to the presence of the invasive species, and compare pathogen occurrence and prevalence between them, providing additional information on their interactions in other regions would strengthen the discussion.

Figures: Figures would be more visually engaging and easier to interpret if presented in color rather than grayscale.

Reviewer #3: 1. Defining key terms such as “invasion gradient,” “invasion front,” and “invasion core” early in the text would help orient readers.

2. Line 118: It may be more appropriate to refer to the “amplification effect hypothesis” rather than the “spillback hypothesis.”

3. Consider using a consistent metric for relative abundance throughout (i.e., proportion of C. glareolus vs. percentage of A. Sylvaticus; lines 189 and 219 and Fig. 2).

4. Line 237: B. henselae is rarely found in rodents (1–2%), and mainly in rats. Most Bartonella strains are not pathogenic to their rodent hosts.

5. Table 2: The evidence for M. haemomuris transmission via lice is dated and limited. More recent findings suggest direct host-to-host transmission may be more important.

6. Table 3: Please clarify what the values in parentheses represent—are they levels with the strongest effect (this does not appear to be the case)?

7. Lines 260–261: Please clarify whether the authors refer to regional species richness or per-host richness, and consider supporting this with appropriate statistics or a figure.

8. Fig. 3: Including Sarcocystidae here would make the figure more representative of the study’s full scope.

9. Line 311: Please specify whether the value 0.15 is a correlation coefficient.

10. Line 341: If the intercept-only model is the best fit, this may suggest no explanatory variable significantly improves the model. Proceeding to additional models should be justified.

11. Lines 463–464: This pattern appears to be shared by A. Sylvaticus as well—clarification would be helpful.

**********

PLOS authors have the option to publish the peer review history of their article (what does this mean?). If published, this will include your full peer review and any attached files.

If you choose “no”, your identity will remain anonymous but your review may still be made public.

**Do you want your identity to be public for this peer review?**  For information about this choice, including consent withdrawal, please see our Privacy Policy.

Reviewer #1: No

Reviewer #2: No

Reviewer #3: No

[NOTE: If reviewer comments were submitted as an attachment file, they will be attached to this email and accessible via the submission site. Please log into your account, locate the manuscript record, and check for the action link "View Attachments". If this link does not appear, there are no attachment files.]

**Figure resubmission:**

While revising your submission, please upload your figure files to the Preflight Analysis and Conversion Engine (PACE) digital diagnostic tool, https://pacev2.apexcovantage.com/. PACE helps ensure that figures meet PLOS requirements. To use PACE, you must first register as a user. Registration is free. Then, login and navigate to the UPLOAD tab, where you will find detailed instructions on how to use the tool. If you encounter any issues or have any questions when using PACE, please email PLOS at figures@plos.org. Please note that Supporting Information files do not need this step. If there are other versions of figure files still present in your submission file inventory at resubmission, please replace them with the PACE-processed versions.

**Reproducibility:**

To enhance the reproducibility of your results, we recommend that authors of applicable studies deposit laboratory protocols in protocols.io, where a protocol can be assigned its own identifier (DOI) such that it can be cited independently in the future. Additionally, PLOS ONE offers an option to publish peer-reviewed clinical study protocols. Read more information on sharing protocols at https://plos.org/protocols?utm_medium=editorial-email&utm_source=authorletters&utm_campaign=protocols

Attachment Submitted filename: PPATHOGENS-D-25-01038.pdf

# Author comment: Author response to Decision Letter 1

**DOI:** 10.1371/journal.ppat.1014349.r002

13 Oct 2025

Attachment Submitted filename: Authors reply to reviewers.docx

# Peer review: Decision Letter 1

**DOI:** 10.1371/journal.ppat.1014349.r003

**Authors:** Debra Bessen, Bersissa Kumsa, Debra Bessen, Bersissa Kumsa

22 Jan 2026

-->PPATHOGENS-D-25-01038R1

Longitudinal study of a rodent invasion reveals shifts in host pathogen interactions.

PLOS Pathogens

Dear Dr. Stuart,

Thank you for submitting your manuscript to PLOS Pathogens. After careful consideration, we feel that it has merit but does not fully meet PLOS Pathogens's publication criteria as it currently stands. Therefore, we invite you to submit a revised version of the manuscript that addresses the points raised during the review process.

Please submit your revised manuscript by Mar 23 2026 11:59PM. If you will need more time than this to complete your revisions, please reply to this message or contact the journal office at plospathogens@plos.org. When you're ready to submit your revision, log on to https://www.editorialmanager.com/ppathogens/ and select the 'Submissions Needing Revision' folder to locate your manuscript file.

Please include the following items when submitting your revised manuscript:

* A letter that responds to each point raised by the editor and reviewer(s). You should upload this letter as a separate file labeled 'Response to Reviewers'. This file does not need to include responses to any formatting updates and technical items listed in the 'Journal Requirements' section below.

* A marked-up copy of your manuscript that highlights changes made to the original version. You should upload this as a separate file labeled 'Revised Manuscript with Track Changes'.

* An unmarked version of your revised paper without tracked changes. You should upload this as a separate file labeled 'Manuscript'.

If you would like to make changes to your financial disclosure, competing interests statement, or data availability statement, please make these updates within the submission form at the time of resubmission. Guidelines for resubmitting your figure files are available below the reviewer comments at the end of this letter.

We look forward to receiving your revised manuscript.

Kind regards,

Bersissa Kumsa, DVM, MSc, PhD

Academic Editor

PLOS Pathogens

Debra Bessen

Section Editor

PLOS Pathogens

Sumita Bhaduri-McIntosh

Editor-in-Chief

PLOS Pathogens

orcid.org/0000-0003-2946-9497

Michael Malim

Editor-in-Chief

PLOS Pathogens

orcid.org/0000-0002-7699-2064

**Additional Editor Comments:**

Dear Authors,

The reviewers have completed their evaluation of your manuscript. I encourage you to revise and resubmit your work, ensuring that all reviewer comments are thoroughly addressed. Please incorporate the feedback carefully and provide a detailed, point-by-point response that clearly outlines every change made in response to the reviewers’ suggestions.

In addition, kindly correct all typographical and grammatical errors, and ensure that the manuscript is prepared in full compliance with the journal’s formatting and submission guidelines.

We look forward to receiving your revised submission.

**Journal Requirements:**

If the reviewer comments include a recommendation to cite specific previously published works, please review and evaluate these publications to determine whether they are relevant and should be cited. There is no requirement to cite these works unless the editor has indicated otherwise.

1) Please ensure that the CRediT author contributions listed for every co-author are completed accurately and in full.

At this stage, the following Authors/Authors require contributions: Andrew McManus, Maxime Galan, Nathalie Charbonnel, Celia Holland, Vincent Sluydts, Annetta Zintl, and Peter Stuart. Please ensure that the full contributions of each author are acknowledged in the "Add/Edit/Remove Authors" section of our submission form.

The list of CRediT author contributions may be found here: https://journals.plos.org/plospathogens/s/authorship#loc-author-contributions

**Reviewers' Comments:**

Reviewer's Responses to Questions

**Part I - Summary**

Please use this section to discuss strengths/weaknesses of study, novelty/significance, general execution and scholarship.

Reviewer #1: Thank you for implementing all the required changes. The manuscript has improved significantly in clarity, and although a few minor revisions and clarifications are still needed, it is very close to being ready for publication.

Reviewer #3: I have carefully reviewed the revised manuscript. The structure is now clearer, and the methods, results, and rationale behind the statistical analyses are easier to follow. The revised presentation also links more directly to the three main study hypotheses, which improves the readability and flow. However, there remain two key issues that, in my view, limit the robustness of the conclusions.

First, the absence of complementary pathogen-specific assays makes it difficult to fully support some of the central patterns—particularly those that contrast with previously reported trends (e.g., the lack of a dilution effect in Bartonella or the pattern opposite to enemy-release expectations in Sarcocystidae). These findings may reflect limitations of the 16S rRNA metabarcoding approach, such as its lower sensitivity or potential biases toward certain strains. Second, while the manuscript concludes that C. glareolus invasion shows signatures consistent with enemy release, a potential dilution effect, and temporal changes in pathogen patterns along the invasion gradient, these patterns are evident in only a small subset of pathogens. In contrast, several other pathogens do not follow these trends. In this context, I think the main take-home message that could be emphasized more strongly is that invasion outcomes appear to depend on the specific host–pathogen association and the timing of the invasion, and therefore cannot be generalized or predicted in advance. Highlighting this point would, in my opinion, provide a balanced and more robust interpretation of the findings.

**********

**Part II – Major Issues: Key Experiments Required for Acceptance**

Please use this section to detail the key new experiments or modifications of existing experiments that should be absolutely required to validate study conclusions.

Generally, there should be no more than 3 such required experiments or major modifications for a "Major Revision" recommendation. If more than 3 experiments are necessary to validate the study conclusions, then you are encouraged to recommend "Reject".

Reviewer #1: n/a

Reviewer #3: In my view, three major issues currently limit the robustness of the conclusions:

1. Lack of complementary pathogen-specific assays (as detailed above).

2. Segregation of bioinformatic analyses into four separate batches (France, 2016, 2017, and 2022). I understand that the sequencing itself was performed in different runs, yet I wonder whether it would be possible to integrate all datasets and cluster them jointly into OTUs. Such an approach could strengthen the comparative framework of the study, particularly for contrasts between regions (e.g., Ireland vs. France) and across time.

3. Emphasis of the take-home messages on the three study hypotheses, rather than highlighting what the data appear to support more strongly—that invasion outcomes depend on the specific host–pathogen associations and the timing of the invasion (as outlined above).

**********

**Part III – Minor Issues: Editorial and Data Presentation Modifications**

Please use this section for editorial suggestions as well as relatively minor modifications of existing data that would enhance clarity.

Reviewer #1: Minor edits, formatting issues, and comments are listed in the attached PDF, along with a few questions that still require clarification to ensure full clarity for the reviewer and the readers. In addition, a further round of language editing would be beneficial to ensure that the manuscript flows smoothly.

Reviewer #3: • Line 250: Should this refer to Table 2 rather than the current table number?

• Table 2 (M. penetrans): How was the confidence interval calculated when only a single host was infected with this parasite?

• Line 285: You may consider replacing “moreover” with “in contrast,” which might better reflect the relationship between the statements.

• Lines 516–518: Using the absence of potentially pathogenic taxa in C. glareolus populations in Ireland as evidence for the enemy release hypothesis may be misleading, given that the same pattern was observed in A. sylvaticus populations.

• References 63 and 64 do not provide evidence that Mycoplasma species have traditionally been considered pathogenic in rodents.

**********

PLOS authors have the option to publish the peer review history of their article (what does this mean?). If published, this will include your full peer review and any attached files.

If you choose “no”, your identity will remain anonymous but your review may still be made public.

**Do you want your identity to be public for this peer review?**  For information about this choice, including consent withdrawal, please see our Privacy Policy.

Reviewer #1: No

Reviewer #3: No

[NOTE: If reviewer comments were submitted as an attachment file, they will be attached to this email and accessible via the submission site. Please log into your account, locate the manuscript record, and check for the action link "View Attachments". If this link does not appear, there are no attachment files.]

**Figure resubmission:**

-->While revising your submission, we strongly recommend that you use PLOS’s NAAS tool (https://ngplosjournals.pagemajik.ai/artanalysis) to test your figure files. NAAS can convert your figure files to the TIFF file type and meet basic requirements (such as print size, resolution), or provide you with a report on issues that do not meet our requirements and that NAAS cannot fix.

After uploading your figures to PLOS’s NAAS tool - https://ngplosjournals.pagemajik.ai/artanalysis, NAAS will process the files provided and display the results in the "Uploaded Files" section of the page as the processing is complete. If the uploaded figures meet our requirements (or NAAS is able to fix the files to meet our requirements), the figure will be marked as "fixed" above. If NAAS is unable to fix the files, a red "failed" label will appear above. When NAAS has confirmed that the figure files meet our requirements, please download the file via the download option, and include these NAAS processed figure files when submitting your revised manuscript.

**Reproducibility:**

To enhance the reproducibility of your results, we recommend that authors of applicable studies deposit laboratory protocols in protocols.io, where a protocol can be assigned its own identifier (DOI) such that it can be cited independently in the future. Additionally, PLOS ONE offers an option to publish peer-reviewed clinical study protocols. Read more information on sharing protocols at https://plos.org/protocols?utm_medium=editorial-email&utm_source=authorletters&utm_campaign=protocols

Attachment Submitted filename: PPATHOGENS-D-25-01038_R1_reviewer.pdf

# Author comment: Author response to Decision Letter 2

**DOI:** 10.1371/journal.ppat.1014349.r004

27 Apr 2026

Attachment Submitted filename: Author response to reviewers R2.docx

# Peer review: Decision Letter 2

**DOI:** 10.1371/journal.ppat.1014349.r005

**Authors:** Debra Bessen, Bersissa Kumsa, Debra Bessen, Bersissa Kumsa, Debra Bessen

3 Jun 2026

Dear Dr Stuart,

We are pleased to inform you that your manuscript 'Longitudinal study of a rodent invasion reveals shifts in host pathogen interactions.' has been provisionally accepted for publication in PLOS Pathogens.

Before your manuscript can be formally accepted you will need to complete some formatting changes, which you will receive in a follow up email. A member of our team will be in touch with a set of requests.

Please note that your manuscript will not be scheduled for publication until you have made the required changes, so a swift response is appreciated.

IMPORTANT: The editorial review process is now complete. PLOS will only permit corrections to spelling, formatting or significant scientific errors from this point onwards. Requests for major changes, or any which affect the scientific understanding of your work, will cause delays to the publication date of your manuscript.

Should you, your institution's press office or the journal office choose to press release your paper, you will automatically be opted out of early publication. We ask that you notify us now if you or your institution is planning to press release the article. All press must be co-ordinated with PLOS.

Thank you again for supporting Open Access publishing; we are looking forward to publishing your work in PLOS Pathogens.

Best regards,

Debra E Bessen

Section Editor

PLOS Pathogens

Debra Bessen

Section Editor

PLOS Pathogens

Sumita Bhaduri-McIntosh

Editor-in-Chief

PLOS Pathogens

orcid.org/0000-0003-2946-9497

Michael Malim

Editor-in-Chief

PLOS Pathogens

orcid.org/0000-0002-7699-2064

***********************************************************

Reviewer Comments (if any, and for reference):

Reviewer's Responses to Questions

**Part I - Summary**

Please use this section to discuss strengths/weaknesses of study, novelty/significance, general execution and scholarship.

Reviewer #3: I thank the authors for their detailed explanations and the revisions made in response to the second round of comments. I am satisfied with the changes and support publication of the manuscript in its current form.

**********

**Part II – Major Issues: Key Experiments Required for Acceptance**

Please use this section to detail the key new experiments or modifications of existing experiments that should be absolutely required to validate study conclusions.

Generally, there should be no more than 3 such required experiments or major modifications for a "Major Revision" recommendation. If more than 3 experiments are necessary to validate the study conclusions, then you are encouraged to recommend "Reject".

Reviewer #3: (No Response)

**********

**Part III – Minor Issues: Editorial and Data Presentation Modifications**

Please use this section for editorial suggestions as well as relatively minor modifications of existing data that would enhance clarity.

Reviewer #3: (No Response)

**********

PLOS authors have the option to publish the peer review history of their article (what does this mean?). If published, this will include your full peer review and any attached files.

If you choose “no”, your identity will remain anonymous but your review may still be made public.

**Do you want your identity to be public for this peer review?**  For information about this choice, including consent withdrawal, please see our Privacy Policy.

Reviewer #3: No

# Editor report: Acceptance letter

**DOI:** 10.1371/journal.ppat.1014349.r006

**Authors:** Debra Bessen, Bersissa Kumsa, Debra Bessen, Bersissa Kumsa, Debra Bessen

Dear Dr Stuart,

We are delighted to inform you that your manuscript, "Longitudinal study of a rodent invasion reveals shifts in host pathogen interactions.," has been formally accepted for publication in PLOS Pathogens.

We have now passed your article onto the PLOS Production Department who will complete the rest of the pre-publication process. All authors will receive a confirmation email upon publication.

The corresponding author will soon be receiving a typeset proof for review, to ensure errors have not been introduced during production. Please review the PDF proof of your manuscript carefully, as this is the last chance to correct any scientific or type-setting errors. Please note that major changes, or those which affect the scientific understanding of the work, will likely cause delays to the publication date of your manuscript. Note: Proofs for Front Matter articles (Pearls, Reviews, Opinions, etc...) are generated on a different schedule and may not be made available as quickly.

Soon after your final files are uploaded, the early version of your manuscript, if you opted to have an early version of your article, will be published online. The date of the early version will be your article's publication date. The final article will be published to the same URL, and all versions of the paper will be accessible to readers.

For Research Articles, you will receive an invoice from PLOS for your publication fee after your manuscript has reached the completed accept phase. If you receive an email requesting payment before acceptance or for any other service, this may be a phishing scheme. Learn how to identify phishing emails and protect your accounts at https://explore.plos.org/phishing.

Thank you again for supporting open-access publishing; we are looking forward to publishing your work in PLOS Pathogens.

Best regards,

Sumita Bhaduri-McIntosh

Editor-in-Chief

PLOS Pathogens

orcid.org/0000-0003-2946-9497

Michael Malim

Editor-in-Chief

PLOS Pathogens

orcid.org/0000-0002-7699-2064
